# The Role of Membrane Fluidization in the Gel-Assisted Formation of Giant Polymersomes

**DOI:** 10.1371/journal.pone.0158729

**Published:** 2016-07-13

**Authors:** Adrienne C. Greene, Ian M. Henderson, Andrew Gomez, Walter F. Paxton, Virginia VanDelinder, George D. Bachand

**Affiliations:** 1 Center for Integrated Nanotechnologies, Sandia National Laboratories, Albuquerque, NM, United States of America; 2 Center for Materials Science and Engineering, Sandia National Laboratories, Albuquerque, NM, United States of America; Martin-Luther-Universität Halle-Wittenberg, GERMANY

## Abstract

Polymersomes are being widely explored as synthetic analogs of lipid vesicles based on their enhanced stability and potential uses in a wide variety of applications in (e.g., drug delivery, cell analogs, etc.). Controlled formation of giant polymersomes for use in membrane studies and cell mimetic systems, however, is currently limited by low-yield production methodologies. Here, we describe for the first time, how the size distribution of giant poly(ethylene glycol)-poly(butadiene) (PEO-PBD) polymersomes formed by gel-assisted rehydration may be controlled based on membrane fluidization. We first show that the average diameter and size distribution of PEO-PBD polymersomes may be readily increased by increasing the temperature of the rehydration solution. Further, we describe a correlative relationship between polymersome size and membrane fluidization through the addition of sucrose during rehydration, enabling the formation of PEO-PBD polymersomes with a range of diameters, including giant-sized vesicles (>100 μm). This correlative relationship suggests that sucrose may function as a small molecule fluidizer during rehydration, enhancing polymer diffusivity during formation and increasing polymersome size. Overall the ability to easily regulate the size of PEO-PBD polymersomes based on membrane fluidity, either through temperature or fluidizers, has broadly applicability in areas including targeted therapeutic delivery and synthetic biology.

## Introduction

Giant unilamellar vesicles (GUVs) are biological membrane models created through the self-assembly of amphiphilic molecules[1]. While lipid GUVs are excellent mimics of bio-membrane systems, they have inherent limitations, including short shelf life and degradation from various environmental perturbations. To overcome these restrictions, the use of polymer vesicles, or polymersomes, is being widely explored as synthetic analogs of lipid vesicles. Polymersomes are created through the self-assembly of amphiphilic block copolymers[2–4], and are renowned for their stability[5], robustness[2], chemical versatility[6,7], barrier properties[2,8], and tunable physical attributes[9–11]. Engineering versatile polymer combinations with additional alterations through surface modification, changes of pH[12], and/or heat[12] allows polymersomes to be used in a wide range of applications.

As polymersomes continue to both supplement and supplant lipid vesicles in many applications, the production and characterization of cell-sized, giant (>4 μm) vesicles becomes increasingly important[13]. Such giant polymer vesicles (polymersomes) are important for, among other things, characterizing the properties of the polymer bilayer[2,5], forming and studying polymer nanotubes by manipulation of the polymer bilayer[3,14], and investigating protein stabilization[15]. In addition to understanding these valuable physical characteristics, polymersomes are excellent candidates for use in developing stable, synthetic biomimetic cells (e.g., protocells)[14] and for controlled biological drug targeting and release[16–18]. Unfortunately, the production of giant polymersomes, necessary for probing properties of polymer membranes[19,20], is currently limited to a few labor-intensive and/or low-yield techniques such as electroformation[2] and templated rehydration[21]. Further, giant polymersomes have recently been used to encaspsulate enzymes capable of triggering release of the vesicle contents[22], and for mimicking human red blood cells for the treatment of malaria[23]. In such applications, the ability to control the size of polymersomes enables loading variable concentrations of biomolecules and tuning vesicles to mimic different endogenous cell sizes.

Recently, a new technique has been developed to create lipid GUVs via gel-assisted rehydration[24]. This method involves depositing an organic solution of lipids onto a dehydrated agarose gel, which, when rehydrated, forms lamellar structures that eventually coalesce into GUVs. Aside from being very convenient and simple, gel-assisted rehydration also enables giant vesicles to be created in physiological salt conditions, an important distinction from other techniques[24,25] and allows for easy reconstitution of membrane proteins in lipid vesicles[26]. Herein we investigated (*i*) the formation of giant polymersomes from amphiphile block copolymers using gel-assisted rehydration, and (*ii*) characterized the role of membrane fluidization on the ability to tune the size of the resulting polymersomes. We further demonstrated polymersomes that may be formed using block copolymers with different physical and chemical properties, as well as with a variety of rehydration solutions including physiological buffers.

## Results and Discussion

### Formation of Polymersomes Using Gel-Assisted Rehydration

**Fig 1:**
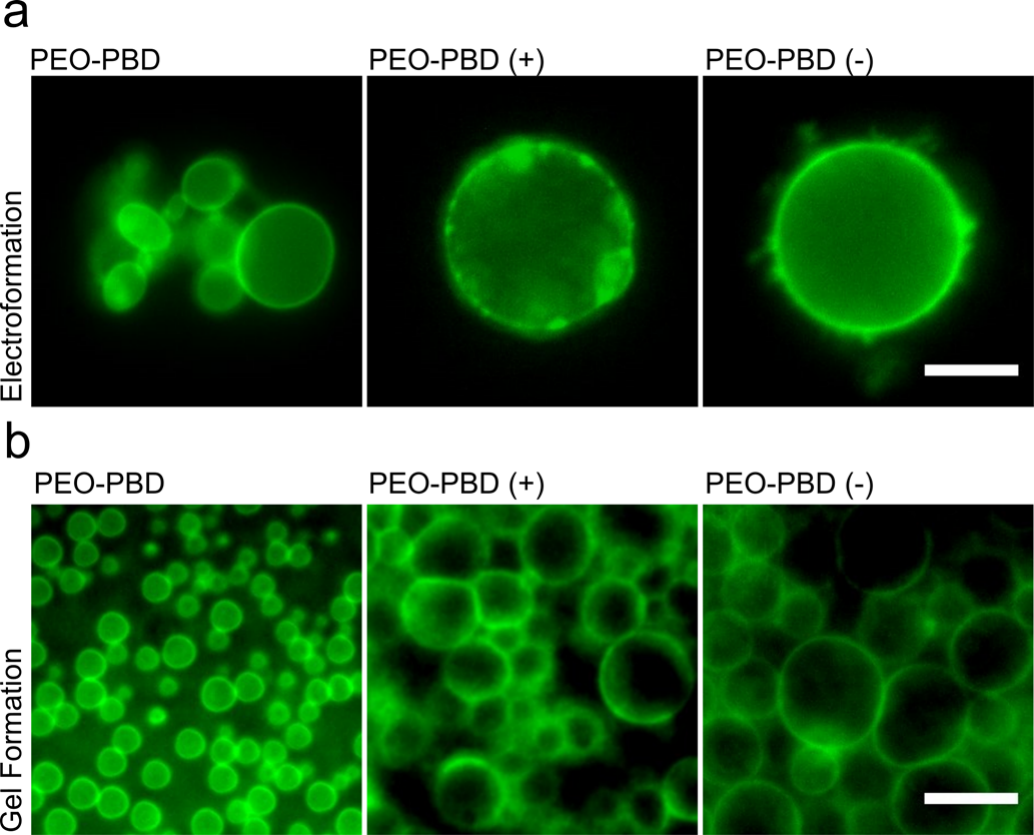
Comparison of polymersomes formed by electroformation and gel-assisted rehydration. (a) Representative epifluorescence photomicrographs depicting PEO-PBD, positively-charged PEO-PBD (NH^3+^) and negatively-charged PEO-PBD (COO^-^) polymersomes formation using platinum wire electroformation. (b) Epifluorescence photomicrographs depicting polymersome formation using gel-assisted rehydration after 1 h on an agarose gel at 40°C. Scale bar = 10 μm.

Polymersome formation using the gel-assisted rehydration method outlined in Fig A in S1 File was directly compared to traditional electroformation. The first polymer tested was a poly(ethylene glycol)-poly(butadiene) diblock copolymer or PEO-PBD, with a total molecular weight of 2,950 Daltons and the following block composition: EO_22_-BD_37_ (Table 1 and Table A in S1 File). Positively and negatively charged PEO-PBD polymers (functionalized with an amine and carboxyl group, respectively) were also evaluated to determine if the charged functional groups affected polymersome formation. Electroformation of polymersomes using the neutral or charged PEO-PBD copolymers did not reliably result in unilamellar vesicles (Fig 1A). Rather, the vesicles that did form had processes protruding from the membrane surface or were asymmetric; reproducibility was likewise problematic. In contrast, formation of polymersomes using gel-assisted rehydration significantly increased the yield, reproducibility and symmetry of polymersome formation with neutral and both charged polymers (Fig 1B). Large populations consisting of several hundred intact neutral PEO-PBD polymersomes formed with an average diameter of 5.8 μm ± 2.5 (Fig 1). Time-lapse photomicrographs showed that polymersome formation begins as early as 25 min post-rehydration (Fig B in S1 File), starkly contrasting the lengthy periods for electroformation and templated rehydration (i.e. several hours).

**Table 1:** Summary of the different polymers tested, their abbreviations used in the text and the molecular weight (M_w_).

**Polymer**	**Abbreviation**	M_w_ (Da)*
**Poly(ethylene glycol)-poly(butadiene)**	PEO-PBD	2,950
**Poly(ethylene glycol)-poly(butadiene)-NH3**^**+**^	PEO-PBD (+)	2,950
**Poly(ethylene glycol)-poly(butadiene)-COO**^**-**^	PEO-PBD (-)	2,950
**Poly(ethylene glycol)-poly(ethylethylene)**	PEO-PEE	3,050
**Poly(ethylene glycol)-poly(propylene oxide)- poly(ethylene glycol)**	PEO-PPO-PEO	8,350

*For further details on the polymer characteristics, see Table A in S1 File. Note that the standard diblock copolymer used throughout this work is abbreviated as PEO-PBD (first entry in the table).

**Fig 2:**
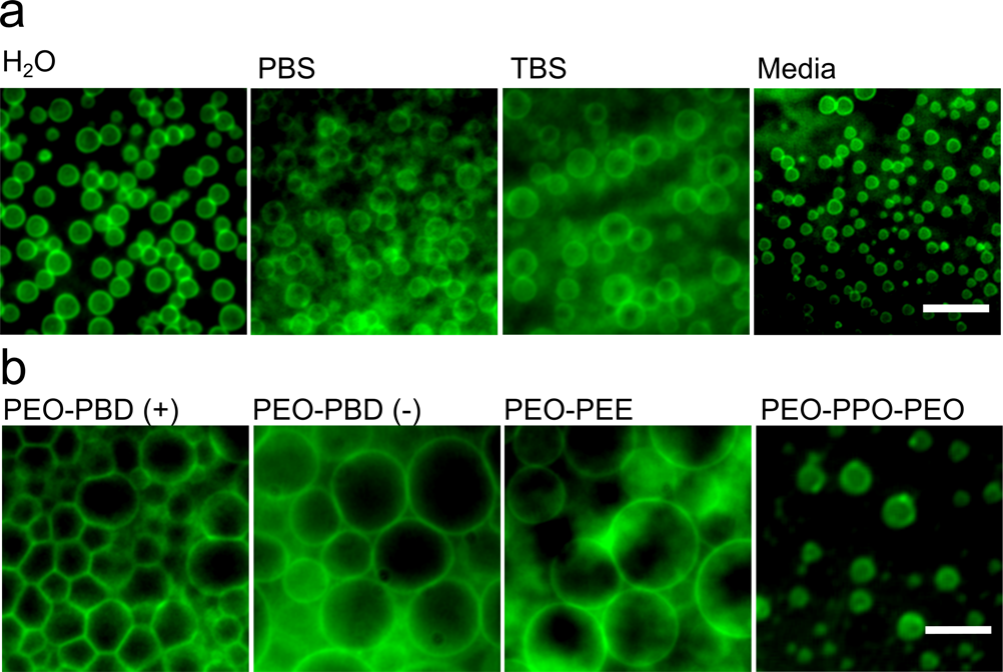
Polymersomes were formed in different buffers and from a variety of polymers following rehydrated with water at 40°C for 1 h on an agarose gel. (a) Epifluorescence photomicrographs of PEO-PBD polymersomes formed using various different rehydration solutions, or (b) with different polymer compositions (See Table 1 and Table A in S1 File for more details on the different polymers). Scale bars = 10 μm.

Traditional methods of polymersome formation are typically limited to rehydration in sucrose solutions such as in electroformation protocols[2]. In contrast, PEO-PBD polymersomes were successfully formed in a variety of physiologically-compatible buffer solutions (Fig 2A) using gel-assisted rehydration. Polymersomes were even successfully formed in a mammalian cell culture medium, collectively rendering this technique useful for studies investigating the creation of artificial cell-like systems, which require biologically-compatible solutions [14,27]. Additionally, polymersomes were stable upon transfer to different media post-formation, though they did respond to alterations in osmotic pressure as expected. Specifically, polymersomes swelled when transferred to a hypotonic solution (S1 Movie), and shrank when transferred to a hypertonic solution (S2 Movie), demonstrating the plasticity of the polymersomes.

Polymersomes were also successfully formed from several different polymer compositions including different diblock copolymers such as poly(ethylene glycol)-poly(ethylethylene) (PEO-PEE) (Fig 2B, Table 1 and Table A in S1 File). As described above, charged PEO-PBD polymers also robustly formed polymersomes (Figs 1B and 2B), and are important synthetic analogs to naturally-occurring charged lipids. Polymersome formation was also attempted with a commercially available triblock co-polymer, poly(ethylene oxide)-*b*-poly(propylene oxide)-*b-*poly(ethylene oxide) (PEO-PPO-PEO), with partially formed vesicle-like structures resulting (Fig 2B and Table A in S1 File). Together these data demonstrate that gel-assisted rehydration is an improved alternative for producing polymersomes with polymer compositions that are more challenging to the formation of polymersomes by other methods.

### Characterization of Polymersomes

**Fig 3:**
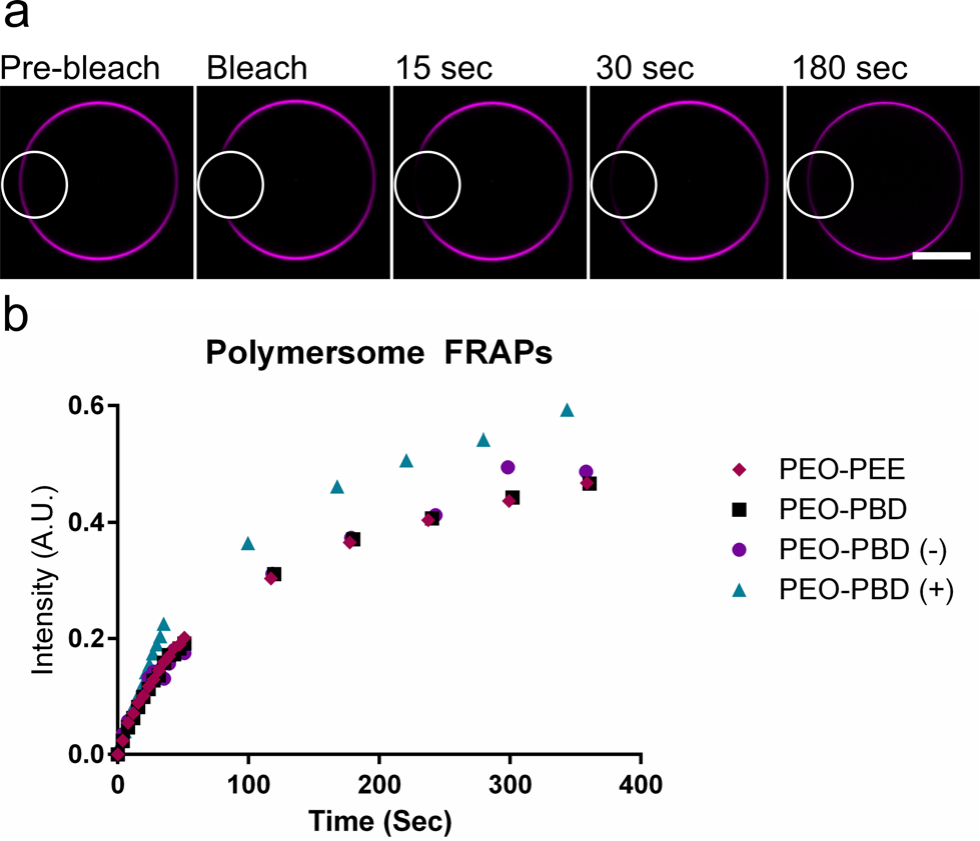
Fluorescence recovery after photobleaching (FRAP) analysis shows that polymersome membranes are fluid. (a) Epifluorescence imaging of a representative PEO-PBD polymersome pre-, during and post-fluorescence bleaching. The region of the membrane that was bleached is circled. Scale bar = 10 μm. (b) Time-dependent fluorescence recovery profiles for different polymers.

To characterize the integrity of the polymersomes, the fluidity of the polymer membranes was measured using fluorescence recovery after photobleaching (FRAP). Representative photomicrographs of FRAP on a neutral PEO-PBD polymersome are shown in Fig 3A. A region of a polymersome membrane containing a small percentage of Liss-rhodamine-labeled lipid was bleached with a laser and the fluorescence recovery was monitored over several minutes (see circled region in the images). The fluorescence in the different polymer compositions recovered over time, as shown in Fig 3B, and plateaued after ~5 min (Fig C in S1 File), indicating that the polymer membranes are fluid. All of the polymersomes tested were fluid across expected timescales, and indeed were similar in their individual recovery rates. FRAP curves were fit to a single component exponential decay according to published methods[28,29] and diffusion coefficients were calculated (see Methods for further details). Diffusion coefficients of the different polymer membranes fell within expected ranges of polymer membrane diffusion[3,30] (Table 2 and Fig D in S1 File), suggesting that the polymersomes formed through gel-assisted rehydration possess properties similar to those formed by traditional methods.

**Table 2:** Diffusion coefficients (mean ± standard error) estimated from FRAP data for polymersomes formed from different polymers.

**Polymer**	Diffusion Coefficient (μm^2^/s)
**PEO-PEE**	0.0287 ± 0.009
**PEO-PBD**	0.0144 ± 0.006
**PEO-PBD (-)**	0.0244 ± 0.003
**PEO-PBD (+)**	0.0142 ± 0.007

### Controlling Polymersome Size with Temperature

**Fig 4:**
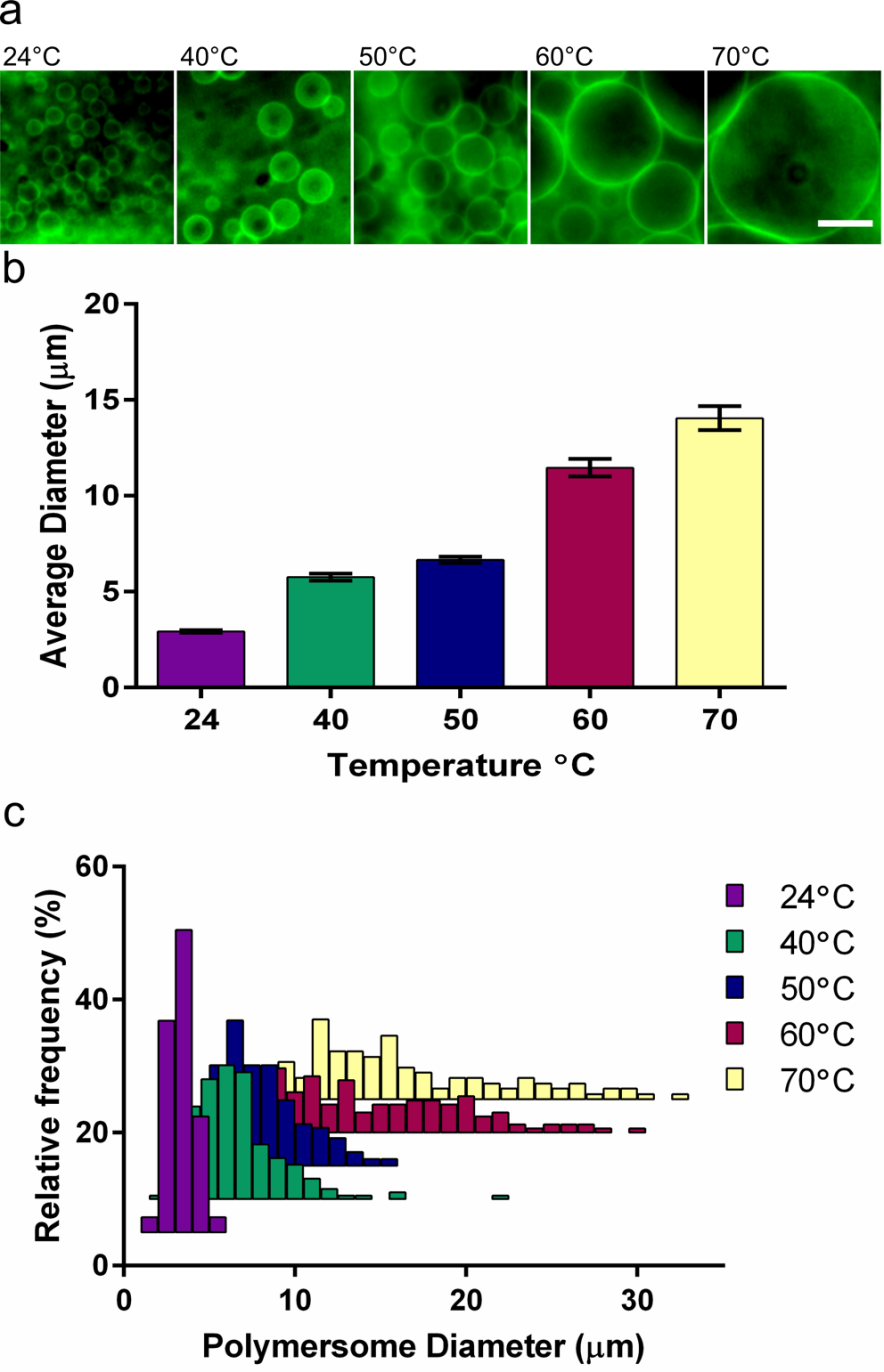
Dependency of vesicle size on different rehydration temperatures. PEO-PBD polymersomes were generated in water on 1% agarose gels for 30 min at varying temperatures on a hot plate. (a) Epifluorescence photomicrographs (scale bar = 10 μm), (b) average diameters (± standard error of the mean), and (c) frequency distribution plots for polymersomes formed at different temperatures.

While methods such as extrusion can mediate size distribution of small polymer vesicles (<0.5 μm), it is generally challenging to control the size of giant polymer vesicles. Using gel-assisted rehydration, size control of the PEO-PBD polymersomes was easily attained by altering the temperature during the rehydration step. As the temperature increased, the average size of the polymersomes concomitantly increased (Fig 4A and 4B). ANOVA analysis of the average diameter of the polymersomes confirmed that temperature significantly affects polymersome size (P < 0.001; Table B in S1 File). Furthermore, pairwise comparisons of these data reveal significant differences in polymersome size among all of the temperatures (P < 0.03), except between the 60°C and 70°C samples in which the average sizes were not significantly different (P = 0.069). Frequency distributions for the polymersome populations also indicated an increase in the dispersity as temperature increased (Fig 4C). Furthermore, increasing the temperature consequently increased the rate of formation, with large intact vesicles being formed in less than 10 min at the higher temperatures. The increased rate of formation and size upon heating is likely due to an increase in PEO-PBD diffusivity, resulting in greater membrane fluidity (and thus acting as a physical membrane fluidizer). Similar effects have been reported with hybrid polymer-lipid membranes[31]. Temperature-dependence of polymersome size provides a predictable, simple and more rapid approach for regulating PEO-PBD polymersome size as compared to other techniques[21,32].

We examined the phase transition of agarose in water in order to evaluate whether its solubilization during hydration may have affected polymersome formation. A melting point apparatus equipped with an optical density filter indicated that the gel undergoes a phase transition and begins to solubilize at 62°C. Thus, the presence of soluble agarose in solution and potential intercalation in polymersome membranes likely has a minimal effect at temperatures lower than the melting point, but cannot be entirely ruled out.

### Controlling Polymersome Size with a Small Molecule Fluidizer

Previous reports using gel-assisted rehydration to form lipid GUVs suggest that formation consists of vesicle swelling upon rehydration, followed by their fusion due to mechanical crowding[24]. Specific conditions (e.g. addition of salts and agitation[11]), however, are necessary to facilitate fusion of polymer vesicles, suggesting that formation of giant polymersomes by gel-assisted rehydration likely relies on different interactions. The relationship between temperature and polymersome size, shown in Fig 4, was attributed to the increased membrane fluidity during rehydration. To further explore the role of membrane fluidization, we tested the effect of adding a small molecule membrane fluidizing agent, sucrose, on the formation of PEO-PBD polymersomes and diffusivity of the polymersome components.

**Fig 5:**
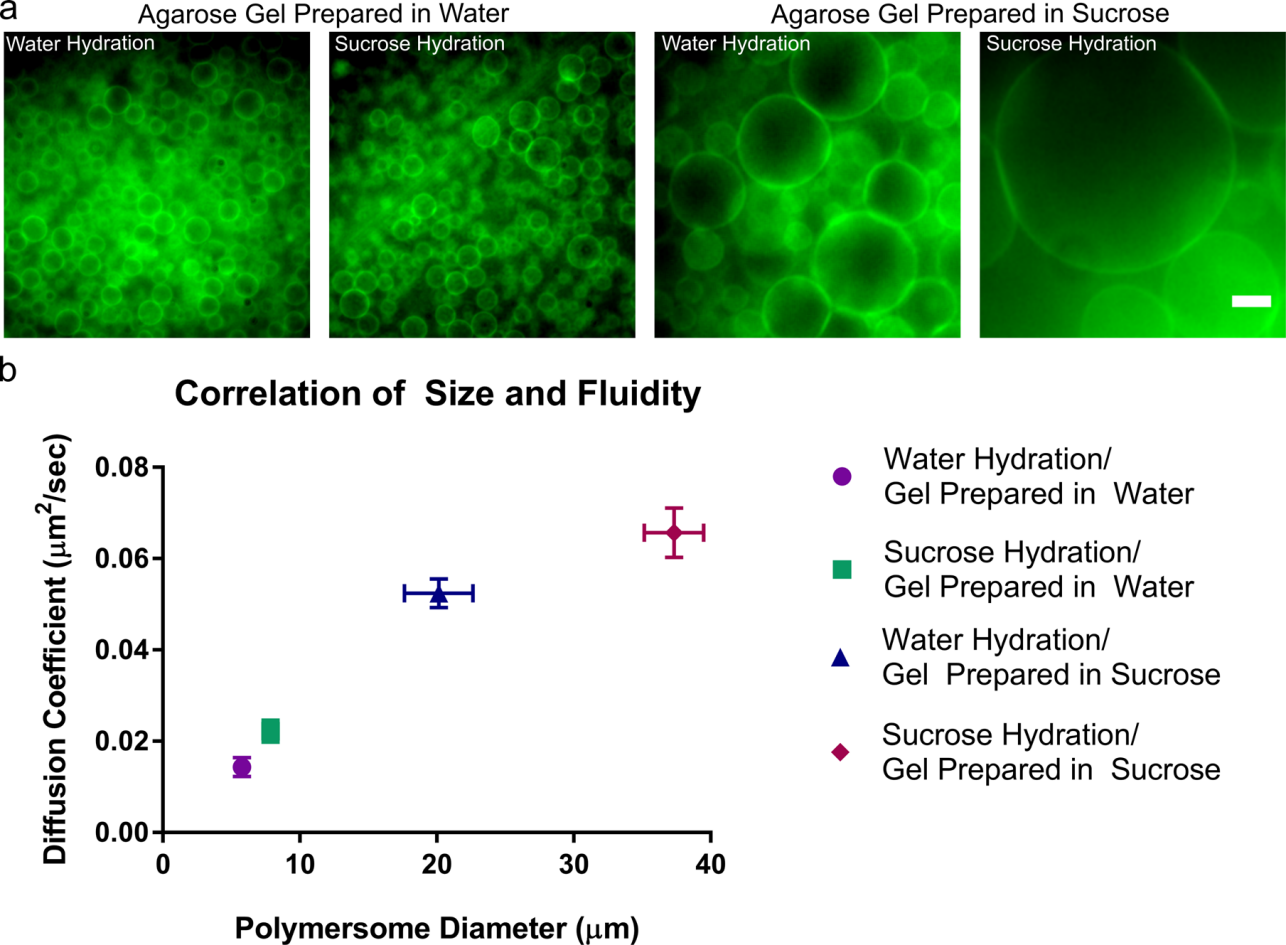
Effects of membrane fluidization on the formation of polymersomes. (a) Epifluorescence photomicrographs of PEO-PBD polymersomes formed on either agarose gels in water (left two images) or agarose gels in sucrose (right two images) and rehydrated in either water or sucrose as indicated at the top of the image. Scale bar = 10 μm. (b) Correlation of polymersome size (measured for over 80 polymersomes) and diffusion coefficients (calculated for at least five different polymersomes). X-error bars are standard error of the mean for the polymersome diameters. Y-error bars are standard error of the mean for the diffusion coefficients.

Sucrose solutions were added at various steps during the gel-assisted rehydration method: (*i*) agarose gels were prepared in a sucrose solution, (*ii*) sucrose was present in the rehydration solution and (*iii*) sucrose was used in both the agarose gel preparation and in the rehydration solution. The addition of sucrose in the gel, with either water or sucrose rehydration resulted in increased vesicle size from vesicles formed on a gel made in water (Fig 5 and Fig E in S1 File). Diffusion coefficients of PEO-PBD polymersomes were measured as described above for each of the different conditions in which sucrose was added (Fig E in S1 File). Plotting the measured diffusion coefficients against the average vesicle size for each condition revealed a strong correlation (Fig 5B), strongly supporting that membrane fluidization aids in the formation of giant polymersomes.

The largest diameter and the broadest size distribution of PEO-PBD polymersomes were formed by hydrating a gel prepared with sucrose and using a sucrose rehydration solution; the fastest rate of formation (<5 min) was also observed under this condition. Here, populations of giant-sized polymersomes measuring 100 μm or greater in diameter were formed (Fig 5 and Fig E in S1 File) supporting the idea that fluidization greatly aids in PEO-PBD polymersome formation. Furthermore, this trend was consistent with the formation of charged PEO-PBD polymersomes at either high temperatures or on an agarose gel made in sucrose and rehydrated in sucrose in which polymersomes were significantly larger than in the control condition (Fig G in S1 File). Finally, sucrose was added in the gel and rehydration solution coupled with heating during rehydration (70°C), but did not result in larger vesicles compared with those formed at 40°C.

The addition of sucrose can also exert an osmotic pressure during vesicle formation, as reported in the formation and detachment of lipid vesicles from the surface[33,34]. While the role of osmotics on the polymersome formation cannot be discounted, our data suggest that membrane fluidization is sufficient to aid in the formation of giant PEO-PBD polymersomes based the similar effects of increasing temperature and adding sucrose. Furthermore, the direct correlation of polymersome size and diffusivity (Fig 5B) suggests that sucrose is indeed fluidizing the polymer membrane, which in turn enhanced the rate of formation and size of polymer vesicles.

To further test the fluidizing effect of sucrose, sucrose was added to high molecular weight polymers that did not initially form polymersomes using gel-assisted rehydration (i.e., using a gel prepared in water, and rehydrated with water; Table A in S1 File). Here the lack of formation may be attributed to potential entanglement of the polymers, which has been reported to increase with increased molecular weight and result in membranes with lower fluidity and permeability[3,35]. The addition of sucrose to the rehydration solution successfully enabled the formation of polymersomes from PEO-PBD polymers with varying block compositions (Table A and Fig F in S1 File). In all cases, polymersome diameter was limited to around 1–5 μm upon addition of sucrose, which further support the importance of membrane fluidization in the formation of polymersomes.

## Conclusion

In summary, we have shown that gel-assisted rehydration is a convenient method for producing cell-sized and considerably larger polymersomes from PEO-PBD block copolymers. In addition to the simple and robust nature of this technique, we describe the ability to modulate membrane fluidity of low molecular weight PEO-PBD polymersomes using temperature and small-molecule fluidizers during rehydration, resulting in an easy means of tuning vesicle size. Based on these observations, we propose that membrane fluidization may be critical to the formation of polymersomes by gel-assisted rehydration, and should be considered particularly when using high molecular block copolymers to form polymersomes. Lastly, we demonstrate the application of gel-assisted rehydration for the formation of polymersomes from charged PEO-PBD, enabling increasingly complex synthetic polymer membranes that better mimic biological plasma membranes[36].

## Methods

### Formation of Agarose Films on Glass Slides by Deposition

The original protocol detailing the formation of agarose films for giant lipid vesicle formation[24] was adapted to the formation of polymersomes. Briefly, 1% (w/v) molecular biology agarose (Sigma-Aldrich, St. Louis, MO; product number A9539) was dissolved in deionized water by boiling. The agarose solution (300 μL) was deposited onto a 25 mm square #1 glass coverslip (VWR, Radnor, PA). The long edge of another pipette tip was used to spread the agarose solution evenly on the coverslip surface. Agarose films were dried by incubating at 40°C for >1 hour and stored at room temperature until use.

### Formation of Polymer Films on the Prepared Agarose Films

All polymers were prepared in chloroform at a 5 mg/mL concentration with 0.5 mol% of either Lissamine Rhodamine B PE lipid (Invitrogen, Inc., Carlsbad, CA) or 0.5 mol% NBD-PC lipid (Avanti Polar Lipids, Inc., Alabaster, AL) for epifluorescence imaging purposes. 30 μL of polymer solution was deposited onto the agarose films and spread evenly across the dried agarose using the long edge of a needle. Polymer films were placed under vacuum at room temperature overnight to fully remove any solvent residues.

### Formation of Polymersomes

Unless otherwise stated, all polymersomes were generated using the following method (Fig A in S1 File): PDMS wells were adhered to the agarose/polymer films and 500 μL deionized water was deposited into the well. Films were incubated for 60 min on a 40°C hotplate prior to imaging directly on the surface. For the buffer compatibility experiments, polymer films were rehydrated in 500 μL of 1x PBS (137 mM NaCl, 2.7 mM KCl, 10 mM Na_2_HPO_4_, 1.8 mM KH_2_PO_4,_ pH 7.4), 1x tris buffered saline (50 mM Tris-Cl, pH 7.5, 150 mM NaCl), 100 mM sucrose in water, or full cell culture media (Dulbecco’s Modified Eagle Medium [Life Technologies, Grand Island, NY], supplemented with 10% fetal bovine serum and 10 mM L-glutamine). Polymersome diameter size was measured using Fiji imaging software[37] (>100 polymersomes/condition) and size distributions were plotted using GraphPad Prism statistics software (La Jolla, CA) (see Table B in S1 File). ANOVA analysis was performed using SigmaPlot (San Jose, CA).

### Optical Characterization of Polymersomes

Polymersomes were imaged using an inverted microscope (Olympus IX81) in epifluorescence with either a 40× or 100× objective (as noted in the text). Images were captured using an Orca-Flash 4.0 cMOS camera (Hamamatsu Photonics, San Diego, CA) and processed using Fiji imaging software[37]. Polymersomes were imaged either directly on the agarose film surface or removed from the agarose surface and adhered to a clean glass substrate.

### Fluorescent Recovery after Photobleaching (FRAP) Analysis of Polymersomes

To characterize fluidity of the polymer membranes, FRAP imaging was performed on a FV-1000 Olympus IX-81 Confocal Laser Scanning Microscope, with FV10-ASW software. A 60× oil objective or 40× air objective was used depending on polymersome size. A multi-line argon laser was used for excitation at 488 nm and 543 nm for NBD and Lissamine Rhodamine dyes respectively. Fluorescence data processing was performed using standard protocols of single component exponential decay as shown below[28,29]. Briefly, a small circular region of the membrane was bleached for approximately 3–5 sec for the NBD dye and ~10–30 seconds for the Lissamine Rhodamine dye at 100% laser power. Fluorescence recovery was imaged over the course of 5–10 min. FRAP data were fit to a single component decay model. The equation used was:

$$F(t)=A(1-exp^{-t\tau })$$ (1)

A is the recovery intensity of the mobile fraction as t→∞, generally bounded with a lower limit of last recorded intensity, τ is the characteristic diffusion time and t is the time at which intensity was recorded. The diffusion constant was then calculated using a previously published equation[28].

$$D=\frac{0.88*\omega^{2}}{4\tau_{1/2}}$$ (2)

ω is the radius of the circular bleach region and the half-life, τ_1/2_, was calculated using Eq 3 for single component exponential decay[28].

$$\tau_{1/2}=\frac{LN(0.5)}{-\tau }$$ (3)

## Supporting Information

S1 File Supporting Information File. Supporting figures mentioned in the text and detailed materials and methods (including polymer synthesis) may be found in the material supplied as Supporting Information.
(DOCX)

S2 File Excel Data Spreadsheets. Zip file archive containing Microsoft Excel spreadsheets with raw data collected from photomicrographs.
(ZIP)

S3 File Raw FRAP Images. Zip file archive containing original photomicrographs obtained in Fluorescence Recovery After Photobleaching (FRAP) experiments.
(ZIP)

S4 File Polymersome Formation by Sucrose Rehydration on Gels Prepared with Sucrose. Zip file archive containing original photomicrographs of polymersomes formed following rehydration with sucrose and on gels prepared with sucrose.
(ZIP)

S5 File Polymersome Formation by Sucrose Rehydration on Gels Prepared with Water. Zip file archive containing original photomicrographs of polymersomes formed following rehydration with sucrose and on gels prepared with water.
(ZIP)

S6 File Polymersome Formation by Sucrose Rehydration on Gels Prepared with Water. Zip file archive containing original photomicrographs of polymersomes formed following rehydration with sucrose and on gels prepared with water.
(ZIP)

S7 File Polymersome Formation by Sucrose Rehydration on Gels Prepared with Water. Zip archive containing original photomicrographs of polymersomes formed following rehydration with water and on gels prepared with water.
(ZIP)

S8 File Polymersome Formation at 24°C. Zip file archive containing original photomicrographs of polymersomes formed following rehydration at 24°C.
(ZIP)

S9 File Polymersome Formation at 40°C. Zip file archive containing original photomicrographs of polymersomes formed following rehydration at 40°C.
(ZIP)

S10 File Polymersome Formation at 50°C. Zip file archive containing original photomicrographs of polymersomes formed following rehydration at 50°C.
(ZIP)

S11 File Polymersome Formation at 60°C. Zip file archive containing original photomicrographs of polymersomes formed following rehydration at 60°C.
(ZIP)

S12 File Polymersome Formation at 70°C. Zip file archive containing original photomicrographs of polymersomes formed following rehydration at 70°C.
(ZIP)

S1 Movie Exposure of PEO-PBD-COO^-^ polymersomes to a hypotonic solution. Time-lapse epifluorescence photomicrographs depicting PEO-PBD-COO^-^ polymersomes formed on a gel prepared in sucrose and rehydrated with sucrose for 1 h at 40°C. The solution was switched to water and images were acquired every 3 seconds. Movie is 5x faster than real time. Scale bar = 10 μm.
(AVI)

S2 Movie Exposure of PEO-PBD-COO^-^ polymersomes to a hypertonic solution. Time-lapse epifluorescence photomicrographs depicting PEO-PBD-COO^-^ polymersomes formed on a gel prepared in sucrose and rehydrated with water for 1 h at 40°C. The solution was switched to PBS and images were acquired every 3 seconds. Movie is 5x faster than real time. Scale bar = 10 μm.
(AVI)

## Abbreviations

Polymer vesicle: polymersome
