# Supplementary material for: The Role of Membrane Fluidization in the Gel-Assisted Formation of Giant Polymersomes
Source: PLoS One. 2016 Jul 13;11(7):e0158729. doi: 10.1371/journal.pone.0158729 (PMC4943728; doi:10.1371/journal.pone.0158729)
Supplement: S1 File — Supporting figures mentioned in the text and detailed materials and methods (including polymer synthesis) may be found in the material supplied as Supporting Information. (DOCX) [file pone.0158729.s001.docx]

Supporting Information

**The Role of Membrane Fluidization in the Gel-Assisted Formation of Giant Polymersomes**

Adrienne C. Greene^1^, Ian M. Henderson^1^, Andrew Gomez^2^, Walter F. Paxton^1^, Virginia VanDelinder^1^, George D. Bachand^1^

^1^Center for Integrated Nanotechnologies, Sandia National Laboratories, Albuquerque, NM, United States

^2^Center for Materials Science & Engineering, Sandia National Laboratories, Albuquerque, NM, United States

**Table A. Summary of the characteristics of different polymers tested and their formation of polymersomes.** Composition lists the chain length of the indicated polymer blocks. Partial formation indicates polymersomes did not fully detach from the surface and/or formed polymersome-like structures, but did not form a fully intact polymersome. Note that the standard polymer used throughout this work is abbreviated as PEO-PBD (first entry in the table).

| Polymer | Abbreviation | M_w_(total) | Block Composition | Charge | Vesicle Formation |
| --- | --- | --- | --- | --- | --- |
| Poly(ethylene glycol)-poly(butadiene)-P2904 | PEO-PBD | 2950 | EO_22_-Bd_37_ | Neutral | Yes |
| Poly(ethylene glycol)-poly(butadiene)-P9757 | PEO-PBD (MW = 4000) | 4000 | EO_34-_Bd_46_ | Neutral | Partial |
| Poly(ethylene glycol)-poly(butadiene)-P3404^§^ | PEO-PBD (MW = 7300) | 7300 | EO_52_-Bd_93_ | Neutral | Partial |
| Poly(ethylene glycol)-poly(butadiene)-P4753 | PEO-PBD (MW = 10400) | 10400 | EO_89_-Bd_120_ | Neutral | Very small |
| Poly(ethylene glycol)-poly(butadiene)-NH^3+^ | PEO-PBD (+) | 2950 | EO_22_-Bd_37_ | Positive | Yes |
| Poly(ethylene glycol)-poly(butadiene)-COO^-^ | PEO-PBD (-) | 2950 | EO_22_-Bd_37_ | Negative | Yes |
| Poly(ethylene glycol)-poly(ethylethylene) | PEO-PEE | 3050 | EO_22_-EE_37_ | Neutral | Yes |
| Poly(ethylene glycol)-poly(propylene oxide)- poly(ethylene glycol) | PEO-PPO-PEO | 8350 | EO_80_-PO_27_-EO_80_ | Neutral | Partial |
| Polystyrene-poly(ethylene glycol) | PS-PEO | 5000 | EO_34_-PS_34_ | Neutral | No |

^§^1,4 addition.

**Table B.** **Descriptive statistics from ANOVA analysis on the size distribution of PEO-PBD polymersomes made at different temperatures.**

| Temperature | Mean diameter (µm) | Min | Max | Range | Standard Deviation |
| --- | --- | --- | --- | --- | --- |
| 24 ˚C | 2.928 | 1.431 | 4.839 | 3.408 | 0.741 |
| 40 ˚C | 5.760 | 1.446 | 20.571 | 19.125 | 2.532 |
| 50 ˚C | 6.654 | 2.017 | 14.126 | 12.109 | 2.361 |
| 60 ˚C | 11.463 | 2.284 | 25.420 | 25.420 | 5.826 |
| 70 ˚C | 14.043 | 2.863 | 32.635 | 32.635 | 6.984 |


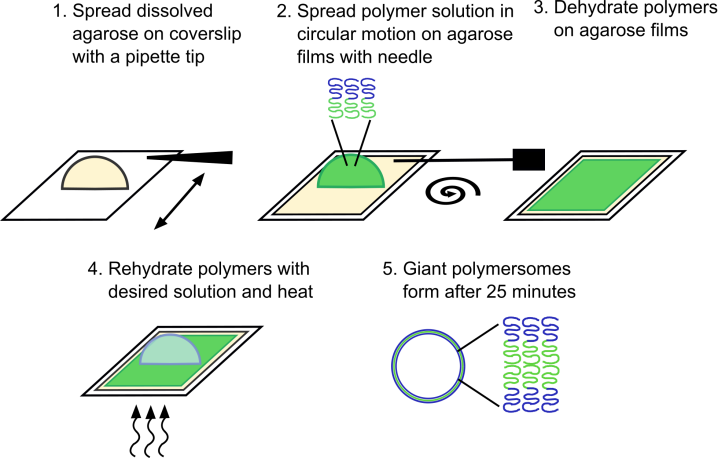


**Fig. A. Schematic depicting the approach for forming polymersomes through gel-assisted rehydration.**

**
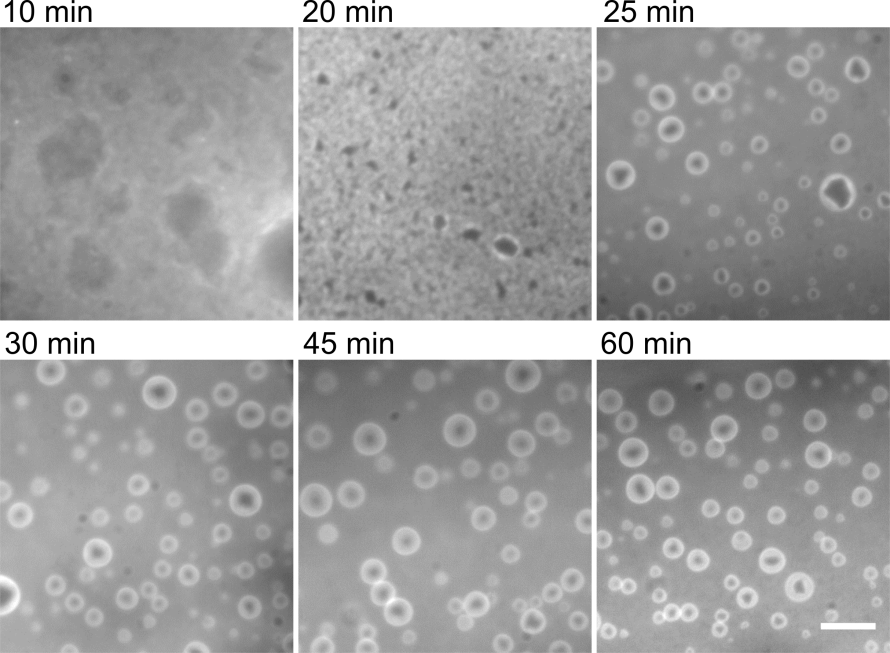
**

**Fig. B. Time-lapse of polymersome giant vesicle formation.** Epifluorescence images depict PEO-PBD polymersome formation over the course of 1 h on an agarose gel at 40 ˚C. Scale bar = 10 µm.





**Fig. C. Fluorescence recovery after photobleaching (FRAP) analysis shows that polymersomes are fluid.** Time-dependent fluorescence recovery profiles for different polymers over 5 minutes. These polymersome FRAPs are from a different data set than those listed in Figure 3 in which these images were collected less frequently and over more time to prevent photobleaching.

***

***

**Fig. D.** **Fluorescence recovery after photobleaching raw data and fits for one representative data set on each polymer type.** Each FRAP was fit to a single exponential equation (see methods for further explanation). The lines represent the fit of the real data points of the corresponding color.

**
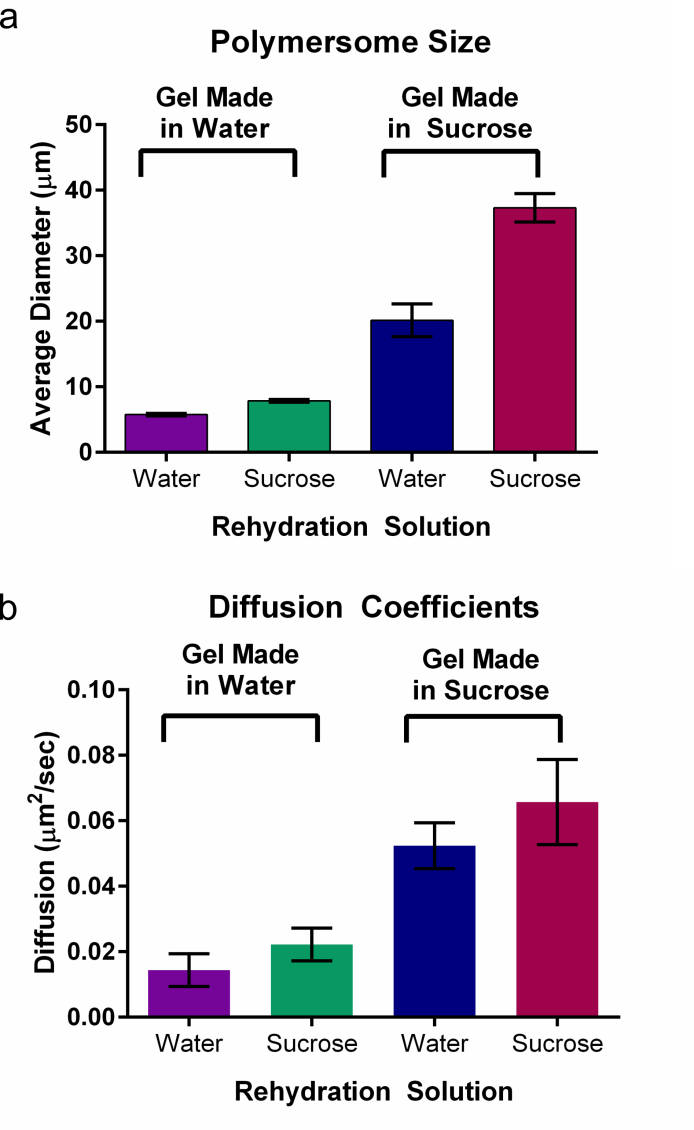
**

**Fig. E. Size and membrane diffusion coefficients of polymersomes made under different conditions.** (a) Average diameter (± standard of the mean) of polymersomes formed in the indicated conditions. (b) Diffusion coefficients (mean ± standard deviation) of the different polymersomes.


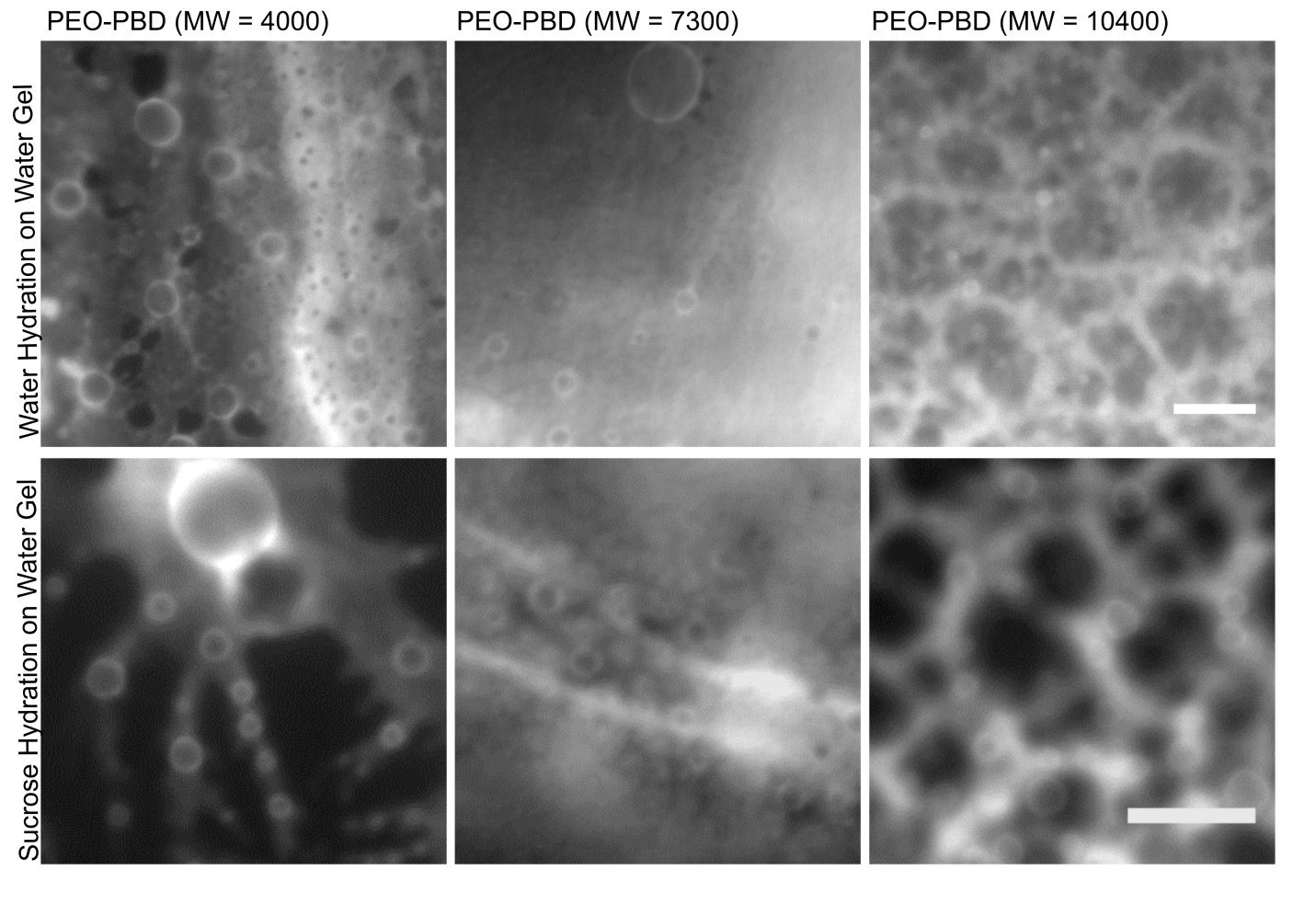


**Fig. F. PEO-PBD polymersome formation with different molecular weight polymers and different sucrose conditions.** Epifluorescence images show that addition of sucrose successfully forms polymersomes with polymers that typically do not form vesicles or do not robustly form vesicles. Scale bar = 10 µm.


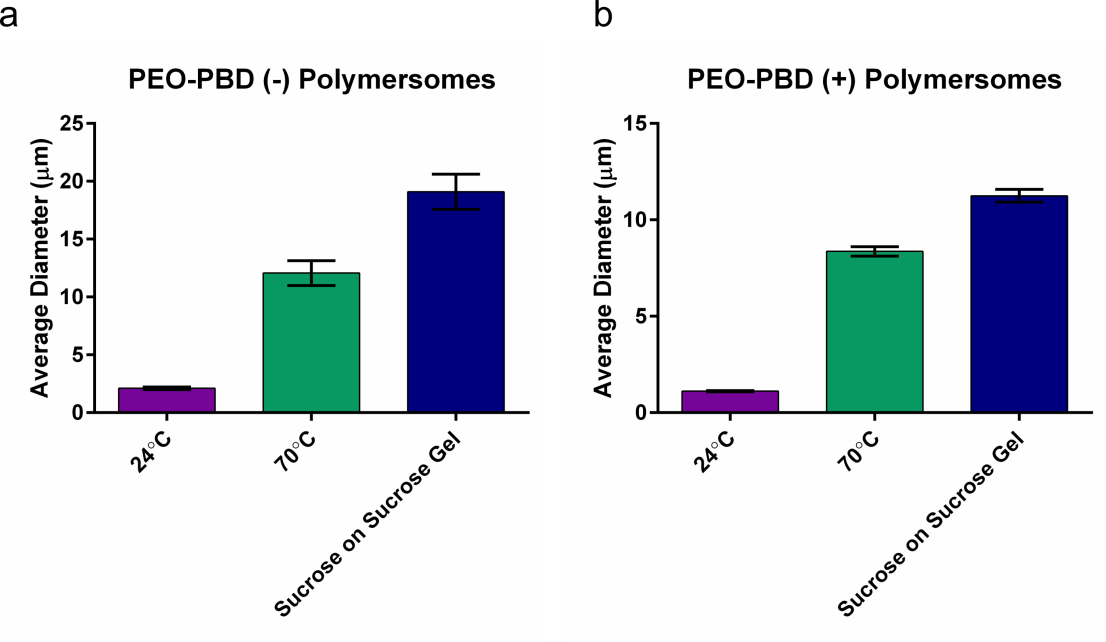


**Fig. G. Charged PEO-PBD polymersomes formed under different conditions.** PEO-PBD polymersomes functionalized with either an NH^2+^ or a COO^-^ were formed at different temperatures and rehydration conditions. Average diameters (± standard error of the mean) of polymersomes functionalized with (a) COO^-^ and (b) NH^2+^.

**Materials and Methods**

**Polymer preparation and characterization**

**Preparation and characterization of mesyl-PEO-PBD**

A 100 mL round bottom flask (RBF) was charged with 1.03 g poly(ethylene glycol)-poly(butadiene) or PEO-PBD with a molecular weight of 2,950 (Polymer # P2904, Polymer Source, Inc.) and 30 mL of methylene chloride (dried over CaH_2_) and fitted with a stir bar and septum. The polymer was dissolved and the solution purged with dry nitrogen for 15 min after which the flask was cooled to 0 ˚C and purged with N_2_ for another 10 min. Methanesulfonyl chloride (0.5 mL) was added via a syringe and the mixture was continuously stirred at 0 ˚C for 5 min. Trimethylamine (TEA) (0.85 mL) was then added slowly to the flask via a syringe. The mixture was allowed to come to room temperature with stirring for a total of 15 h.

The contents of the flask were then diluted with 300 mL of methylene chloride, and washed with aqueous 1 N HCl (3 × 300 mL) and then washed with aqueous saturated sodium bicarbonate (3 × 300 mL). Finally, the organic fraction was dried over sodium sulfate, filtered, and the solvent removed under *vacuo* to recover 0.60 g of the mesylated polymer (58% yield). ^1^H NMR (90 MHz, CDCl_3_): δ 5.5-4.5 (br, 108H, PBD alkenes), 4.28 (t, 2H, -CH_2_-C*H_2_*-O-SO_2_CH_3_) 3.6 (br s, 86H, PEO ether protons), 3.01 (s, 3H, -O-SO_2_C*H*_3_) 2.3-0.5 (br, 108H, PBD aliphatic protons).

**Preparation and characterization of phthalimidyl-PEO-PBD**

Mesyl-PEO-PBD (0.45 g), 0.30 g potassium phthlamide, and 10 mL DMF were added to a 50 mL round bottom flask fitted with a stirbar and septum, and purged with N_2_. The mixture was brought to 50 ˚C for 30 min, then cooled to room temperature and let stirred under N_2_ overnight. The DMF was then removed under *vacuo* and replaced with 15 mL of tetrahydrofuran. The excess potassium phthalimide was removed via filtration, and the residuals purified via preparatory gel permeation chromatography (GPC) to yield 0.135 g of the polymer product. ^1^H NMR (90 MHz, CDCl_3_): δ 7.71 (br m, 4H, phthalyl group), 5.5-4.5 (br m, 108H, PBD alkenes), 3.6 (br s, 86 H, PEO ether backbone protons), 2.3-0.5 (br, 108H, PBD aliphatic backbone protons).

**Preparation and characterization of amine functionalized PEO-PBD polymers**

The phthalimidyl polymer (0.125 g) was placed in a 25 mL round bottom flask along with 10 mL of ethanol and a stir bar. Once the polymer was suspended in solution then 0.5 mL of hydrazine hydrate was added and the mixture brought to reflux under N_2_ for 1 h. Solvent was then removed under vacuum and the polymer re-dissolved in THF and purified via preparatory GPC to yield 42 mg of the product. Formation of the amine was denoted by the disappearance of the phthalimide peaks in NMR.

**Preparation and characterization PEO-PBD ethyl ester**

A 50 mL round bottom flask was charged with 0.43 g of PEO-PBD and 20 mg of a 60% NaH dispersion in mineral oil. The flask was fitted with a septum and purged for 10 min. with dry N_2_. Anhydrous THF (10 mL) was then added via a syringe through the septum and the resulting solution was further purged with N_2_ for 20 min. The solution was stirred for another 20 min under N_2_ prior to the addition of 0.1 mL of ethyl bromoacetate via syringe. The resulting mixture was stirred under nitrogen for another 3 h. The suspension was filtered through a 0.2 μm syringe filter before being purified by preparatory GPC to yield 0.32 g of the product. ^1^H NMR (90 MHz CDCl_3_): δ 5.5-4.5 (br m, 108H, PBD alkenes), 4.1, (t, (coupling constants? J = x Hz) 2H, C(O)C*H*_2_CH_2_), 3.6 (br s, 86 H, PEO ether backbone), 2.87 2.3-0.5 (br s, 108H, PBD aliphatic backbone).

**Preparation and characterization carboxyl functionalized PEO-PBD polymers**

To a 50 mL round bottom flask was added 44 mg of PEO-PBD ethyl ester dissolved in 1 mL of THF along with 10 mL of aqueous 5 % KOH and stirred over night at 50 ˚C under N_2_. The mixture was then acidified with HCl until the pH was approximately 2. The mixture was then extracted into methylene chloride (3 × 10 mL), the organic phase isolated, dried over anhydrous NaSO_4_, filtered, solvent stripped and the residuals purified by GPC.

**Formation of polymersomes using gel-assisted rehydration**

**Formation of agarose films on glass slides by deposition**

The original protocol detailing the formation of agarose films for giant lipid vesicle formation[1] was adapted to the formation of polymersomes. Molecular biology agarose (Sigma-Aldrich, St. Louis, MO; product number A9539) was dissolved at 1% (w/v) in deionized water by boiling. The agarose solution (300 µL) was deposited onto a 25 mm square #1 glass coverslip (VWR, Radnor, PA). The long edge of another pipette tip was used to spread the agarose solution evenly on the coverslip surface. Agarose films were dried by incubating at 40 ˚C for >1 hour and stored at room temperature until use.

**Formation of polymer films on the prepared agarose films**

All polymers were prepared in chloroform at a 5 mg/mL concentration with 0.5 mol% of either Lissamine Rhodamine B PE lipid (Invitrogen, Inc., Carlsbad, CA) or 0.5 mol% NBD-PC lipid (Avanti Polar Lipids, Inc., Alabaster, AL) for epifluorescence imaging purposes. 30 µL of polymer solution was deposited onto the agarose films and spread evenly across the dried agarose using the long edge of a needle. Polymer films were placed under vacuum at room temperature overnight to fully remove any solvent residues.

**Formation of polymersomes on different substrates**

Different gel substrates were prepared and tested for the formation of polymersomes. 1% (w/v) ultra-pure low melting point agarose gels (Life Technologies, Grand Island, NY) and 1% (w/v) 2-Hydroxyethylagarose (Sigma-Aldrich, St. Louis, MO) were prepared using the same method as for the regular (high melting point) agarose gels described above. The following different agarose gel conditions were also evaluated: 1% (w/v) agarose dissolved in water and 1% (w/v) agarose dissolved in 100 mM sucrose in water. Poly(ethylene glycol)-poly(butadiene) or PEO-PBD (Polymer Source, Inc., Dorval, Canada) neutral polymer films were formed on the different gel substrates and different gel conditions as well as a plain glass substrate.

For the acrylamide gel preparation, cover slips (25 mm × 25 mm, VWR, Radnor, PA) were cleaned with a piranha solution (3:1 sulfuric acid to 30% (w/v) hydrogen peroxide), rinsed with deionized water and dried in a 50 °C oven for 1 h. The slides were then incubated in 200 mL of anhydrous toluene and 5 mL of (3-aminopropyl)triethoxysilane (APTES, Sigma-Aldrich, St. Louis, MO) at 50 ˚C under N_2_ for 2 h. Slides were washed with acetone, deionized water and then methanol and dried in a 50 ˚C oven for 30 min. The slides were then placed in an oven-dried 300 mL beaker containing a stir bar and 150 mL of anhydrous benzene. The beaker was placed in an ice bath and allowed to cool for 20 min. Under a stream of N_2_, 5 mL of methacyloyl chloride was added and stirred under N_2_ for 5 min followed by the addition of 5 mL of tripropyl amine and incubated overnight at room temperature. The slides were washed with acetone, methanol and water and 0.95 g of acrylamide and 0.056 g of methylene bis-acrylamide dissolved in 10 mL of deionized water was then added. 50 μL of a 10% (w/v) solution of ammonium persulfate followed by 10 μL of TEMED were added to the mixture and agitated. 1 mL of the final solution was transferred to the coverslip via a syringe. After the gel formed (>20 min), each cover slip was rinsed in deionized water for at least 4 h prior to use.

**Formation of polymersomes**

Unless otherwise stated, all polymersomes were generated using the following method (Scheme 1): PDMS wells were adhered to the agarose/polymer films and 500 µL deionized water was deposited into the well. Films were incubated for 60 min on a 40 ˚C hotplate prior to imaging directly on the surface. For the buffer compatibility experiments, polymer films were rehydrated in 500 µL of 1x PBS (137 mM NaCl, 2.7 mM KCl, 10 mM Na_2_HPO_4_, 1.8 mM KH_2_PO_4,_ pH 7.4), 1x tris buffered saline (50 mM Tris-Cl, pH 7.5, 150 mM NaCl), 100 mM sucrose in water, or full cell culture media (Dulbecco’s Modified Eagle Medium [Life Technologies, Grand Island, NY], supplemented with 10% fetal bovine serum and 10 mM L-glutamine).

**Formation of polymersomes at different temperatures**

The effect of temperature on the formation of polymersomes was tested using the procedure stated above, but incubated on a hotplate with the following temperatures: 24 ˚C, 40 ˚C, 50 ˚C, 60 ˚C and 70 ˚C. Polymersome diameter size was measured using Fiji imaging software[2] (>100 polymersomes/condition) and size distributions were plotted using GraphPad Prism statistics software (La Jolla, CA) (see S2 Table). ANOVA analysis was performed using SigmaPlot (San Jose, CA).

**Sucrose-assisted rehydration**

Formation of polymersomes was compared across the following four conditions: 1) 1% (w/v) agarose gels formed in water and PEO-PBD polymers rehydrated using deionized water, 2) 1% (w/v) agarose gels formed in water and PEO-PBD polymers rehydrated using 100mM sucrose, 3) 1% (w/v) agarose gels formed in a 100 mM sucrose solution and PEO-PBD polymers rehydrated using deionized water and 4) 1% (w/v) agarose gels formed in a 100 mM sucrose solution and PEO-PBD polymers rehydrated using a 100 mM sucrose solution. Polymersome diameter was measured using Fiji imaging software and size distributions were plotted using GraphPad Prism statistics software (La Jolla, CA). Two-way analysis of variance (ANOVA) analysis was performed using SigmaPlot (San Jose, CA). Polymers of different molecular weights were deposited onto 1% (w/w) agarose gels and rehydrated with either deionized water or 100 mM sucrose (see S1 Table).

**Electroformation of polymersomes**

Formation of polymersomes using gel-assisted rehydration was directly compared to traditional platinum wire electroformation. The electroformation settings were conducted as follows: ~30 µL of a 5 mg/mL polymer solution in chloroform was deposited onto two platinum wires spaced 1 mm apart. The wire apparatus was placed under vacuum overnight followed by placing in a 1.5 mL cuvette containing 100 mM sucrose. Electroformation was conducted using an attack phase of 15 min at 10 hz and 0-2 V, an envelope phase of 90 min at 10 hz and 2 V, and a decay phase of 15-20 min at 4 hz and 4 V. Fig 1 shows two representative images of polymer structures formed using this electroformation method with three different types of polymers: neutral PEO-PBD and positively or negatively charged PEO-PBD (functionalized with either an amine or carboxyl group, respectively).

**Optical characterization of polymersomes**

Polymersomes were imaged using an inverted microscope (Olympus IX81) in epifluorescence with either a 40× or 100× objective (as noted in the text). Images were captured using an Orca-Flash 4.0 cMOS camera (Hamamatsu Photonics, San Diego, CA) and processed using Fiji imaging software[2]. Polymersomes were imaged either directly on the agarose film surface or removed from the agarose surface and adhered to a clean glass substrate. To remove the polymersomes from the surface, coverslips were allowed to incubate overnight at room temperature and gently pipetted off of the surface using a 200 µL pipette with the end of the tip cut off and gently repeated up and down pipetting. Surface modified coverslips were used to minimize floating and movement of detached polymersomes. Circular silicone isolator wells (Electron Microscopy Sciences, Hatfield, PA) of diameter 9 mm and depth 0.5 mm were added to modified coverslips. Polymersomes were added to the well enclosure area and sealed with another coverslip for incubation. Minimum incubation time was 15 min-1 h. For incubations longer than 1 h, coverslips were placed in a humidity chamber to prevent evaporation. Coverslips treated with ozone for 15 min were used to create hydrophilic surfaces and worked well for imaging neutral PEO-PBD polymersomes. Negatively-charged polymersomes (PEO-PBD polymers functionalized with COO^-^, or PEO-PBD[-]) were imaged on piranha cleaned coverslips treated with APTES. APTES functionalization was done following standard APTES-coating protocols. Briefly, 2% APTES, 5% deionized water, and 93% of a 95% ethanol solution were mixed and hydrolyzed for 5 min before adding to the coverslips. Coverslips were functionalized with APTES for 10 min followed by 4-5 rinses with 95% ethanol. The coverslips were then cured for 15 min at ~100 ˚C. Polymersomes were added to gasket enclosure and incubated for imaging as previously described. Positively charged polymersomes (PEO-PBD polymers functionalized with NH^3+^, or PEO-PBD[+]) were difficult to recover from the agarose surface using pipette removal. Previous in house experiments studies using positively charged polymersomes formed by electroformation methods showed that casein passivated glass assists in bringing PEO-PBD (+) polymersomes down to the surface, even though the production yield was low. Coverslips were then treated with ~18 mg/mL of casein in Tris buffer for 5 min. The casein solution was then wicked off followed by the additional of the PEO-PBD (+) polymersomes. Minimum incubation time was 2-4 h. Additionally, polymersomes formed in sucrose can also be diluted in a glucose solution of the same osmolarity to bring polymersomes down to the surface.

**Fluorescent Recovery after Photobleaching (FRAP) analysis of polymersomes**

To characterize fluidity of the polymer membranes, FRAP imaging was performed on a FV-1000 Olympus IX-81 Confocal Laser Scanning Microscope, with FV10-ASW software. A 60× oil objective or 40× air objective was used depending on polymersome size. A multi-line argon laser was used for excitation at 488 nm and 543 nm for NBD and Lissamine Rhodamine dyes respectively. Fluorescence data processing was performed using standard protocols of single component exponential decay as shown below[3,4]. Briefly, a small circular region of the membrane was bleached for approximately 3-5 sec for the NBD dye and ~10-30 seconds for the Lissamine Rhodamine dye at 100% laser power. Fluorescence recovery was imaged over the course of 5-10 min. FRAP data were fit to a single component decay model. The equation used was:

$F\left( t \right)=A(1-{exp}^{-t\tau}$) (1)

A is the recovery intensity of the mobile fraction as t→∞, generally bounded with a lower limit of last recorded intensity, τ is the characteristic diffusion time and t is the time at which intensity was recorded. The diffusion constant was then calculated using a previously published equation[3].

$D=\frac{0.88*\omega^{2}}{4\tau_{1/2}}$ (2)

ω is the radius of the circular bleach region and the half-life, τ_1/2_, was calculated using equation 3 for single component exponential decay[3].

$\tau_{1/2}=\frac{LN\left( 0.5 \right)}{-\tau}$ (3)

**References**

1. Horger KS, Estes DJ, Capone R, Mayer M. Films of Agarose Enable Rapid Formation of Giant Liposomes in Solutions of Physiologic Ionic Strength. J Am Chem Soc. 2009;131: 1810–1819. doi:10.1021/ja805625u

2. Schindelin J, Arganda-Carreras I, Frise E, Kaynig V, Longair M, Pietzsch T, et al. Fiji: an open-source platform for biological-image analysis. Nat Methods. 2012;9: 676–82. doi:10.1038/nmeth.2019

3. Axelrod D, Koppel DE, Schlessinger J, Elson E, Webb WW. Mobility measurement by analysis of fluorescence photobleaching recovery kinetics. Biophys J. Elsevier; 1976;16: 1055–1069. doi:10.1016/0002-9610(76)90409-8

4. Jönsson P, Jonsson MP, Tegenfeldt JO, Höök F. A method improving the accuracy of fluorescence recovery after photobleaching analysis. Biophys J. 2008;95: 5334–5348. doi:10.1529/biophysj.108.134874
