# Supplementary figures and images for: The Role of Membrane Fluidization in the Gel-Assisted Formation of Giant Polymersomes (part 2 of 3)
Source: PLoS One. 2016 Jul 13;11(7):e0158729. doi: 10.1371/journal.pone.0158729 (PMC4943728; doi:10.1371/journal.pone.0158729)

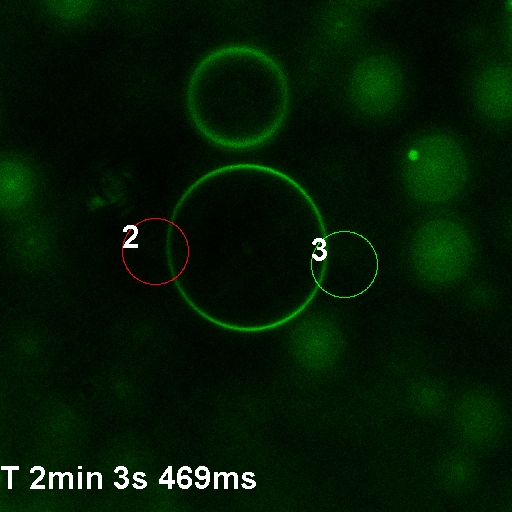

Supplement: S3 File — Zip file archive containing original photomicrographs obtained in Fluorescence Recovery After Photobleaching (FRAP) experiments. (ZIP) [file pone.0158729.s003.zip › PEO-PBD-NH2+ FRAP Fast/NH2 On Surface Free Run 15 activation 10frame 20us_pix_C001T016.jpg]

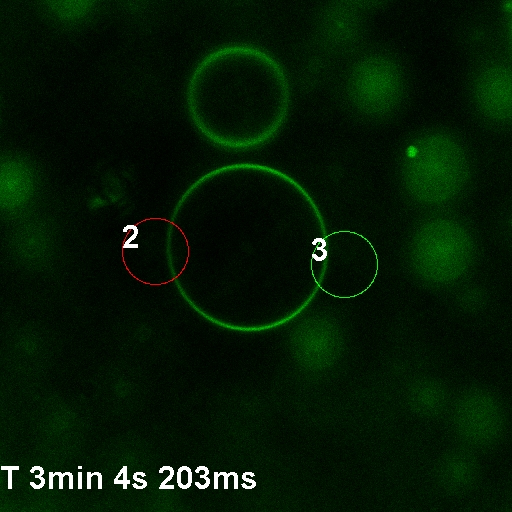

Supplement: S3 File — Zip file archive containing original photomicrographs obtained in Fluorescence Recovery After Photobleaching (FRAP) experiments. (ZIP) [file pone.0158729.s003.zip › PEO-PBD-NH2+ FRAP Fast/NH2 On Surface Free Run 15 activation 10frame 20us_pix_C001T017.jpg]

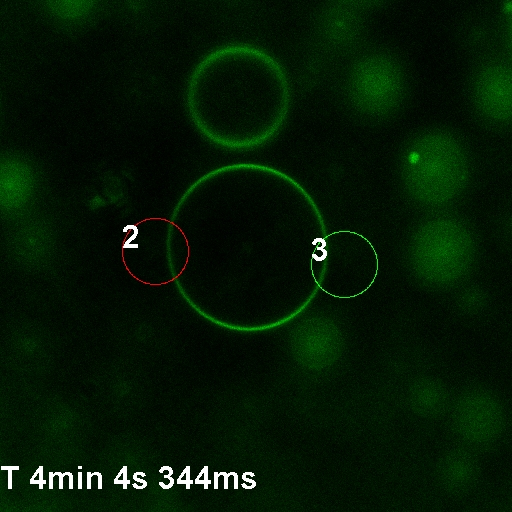

Supplement: S3 File — Zip file archive containing original photomicrographs obtained in Fluorescence Recovery After Photobleaching (FRAP) experiments. (ZIP) [file pone.0158729.s003.zip › PEO-PBD-NH2+ FRAP Fast/NH2 On Surface Free Run 15 activation 10frame 20us_pix_C001T018.jpg]

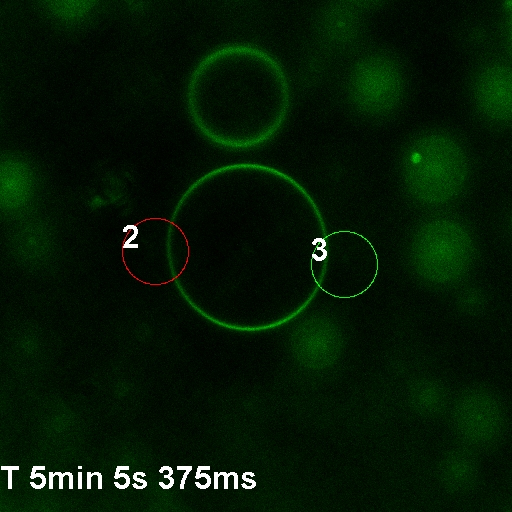

Supplement: S3 File — Zip file archive containing original photomicrographs obtained in Fluorescence Recovery After Photobleaching (FRAP) experiments. (ZIP) [file pone.0158729.s003.zip › PEO-PBD-NH2+ FRAP Fast/NH2 On Surface Free Run 15 activation 10frame 20us_pix_C001T019.jpg]

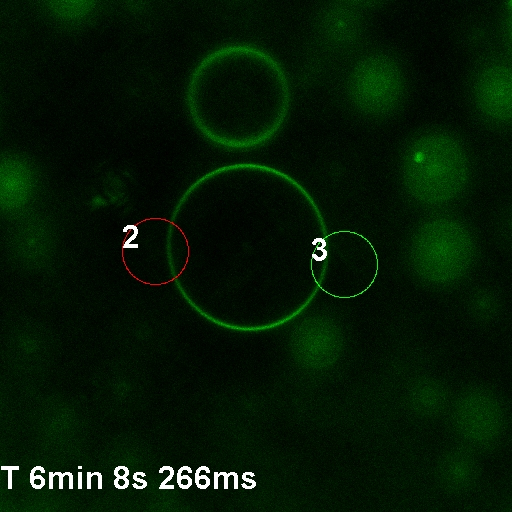

Supplement: S3 File — Zip file archive containing original photomicrographs obtained in Fluorescence Recovery After Photobleaching (FRAP) experiments. (ZIP) [file pone.0158729.s003.zip › PEO-PBD-NH2+ FRAP Fast/NH2 On Surface Free Run 15 activation 10frame 20us_pix_C001T020.jpg]

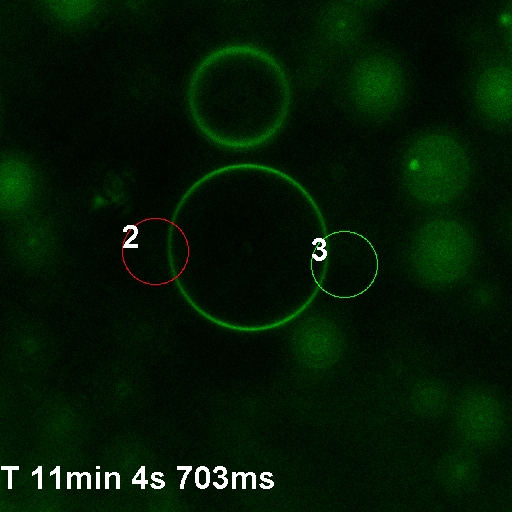

Supplement: S3 File — Zip file archive containing original photomicrographs obtained in Fluorescence Recovery After Photobleaching (FRAP) experiments. (ZIP) [file pone.0158729.s003.zip › PEO-PBD-NH2+ FRAP Fast/NH2 On Surface Free Run 15 activation 10frame 20us_pix_C001T021.jpg]

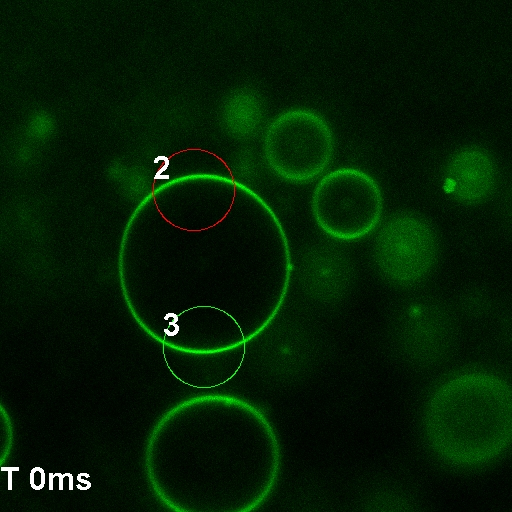

Supplement: S3 File — Zip file archive containing original photomicrographs obtained in Fluorescence Recovery After Photobleaching (FRAP) experiments. (ZIP) [file pone.0158729.s003.zip › PEO-PBD-NH2+ FRAP Slow/NH2 On Surface Reg Activatin 20us_pix 10 frame_C001T001.jpg]

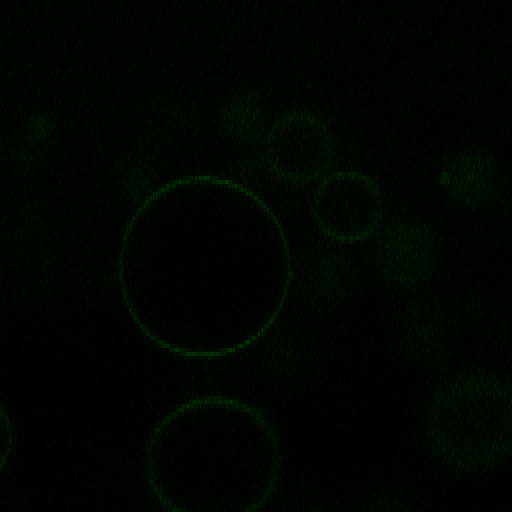

Supplement: S3 File — Zip file archive containing original photomicrographs obtained in Fluorescence Recovery After Photobleaching (FRAP) experiments. (ZIP) [file pone.0158729.s003.zip › PEO-PBD-NH2+ FRAP Slow/NH2 On Surface Reg Activatin 20us_pix 10 frame_C001T001-R001.jpg]

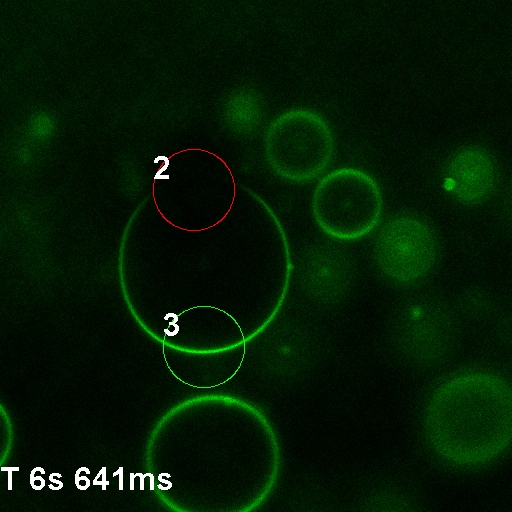

Supplement: S3 File — Zip file archive containing original photomicrographs obtained in Fluorescence Recovery After Photobleaching (FRAP) experiments. (ZIP) [file pone.0158729.s003.zip › PEO-PBD-NH2+ FRAP Slow/NH2 On Surface Reg Activatin 20us_pix 10 frame_C001T002.jpg]

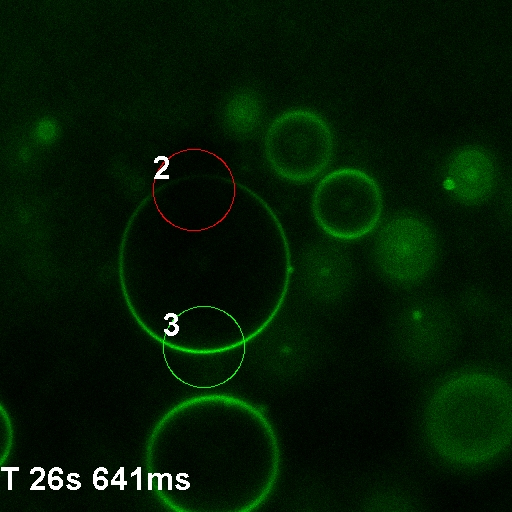

Supplement: S3 File — Zip file archive containing original photomicrographs obtained in Fluorescence Recovery After Photobleaching (FRAP) experiments. (ZIP) [file pone.0158729.s003.zip › PEO-PBD-NH2+ FRAP Slow/NH2 On Surface Reg Activatin 20us_pix 10 frame_C001T003.jpg]

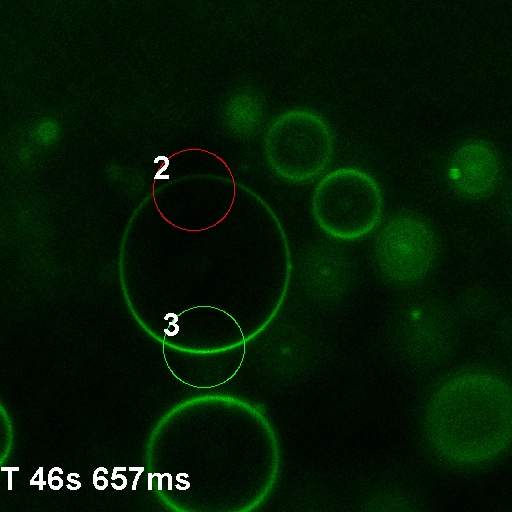

Supplement: S3 File — Zip file archive containing original photomicrographs obtained in Fluorescence Recovery After Photobleaching (FRAP) experiments. (ZIP) [file pone.0158729.s003.zip › PEO-PBD-NH2+ FRAP Slow/NH2 On Surface Reg Activatin 20us_pix 10 frame_C001T004.jpg]

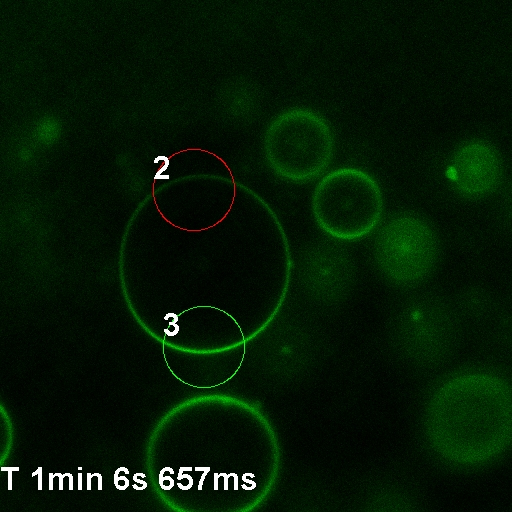

Supplement: S3 File — Zip file archive containing original photomicrographs obtained in Fluorescence Recovery After Photobleaching (FRAP) experiments. (ZIP) [file pone.0158729.s003.zip › PEO-PBD-NH2+ FRAP Slow/NH2 On Surface Reg Activatin 20us_pix 10 frame_C001T005.jpg]

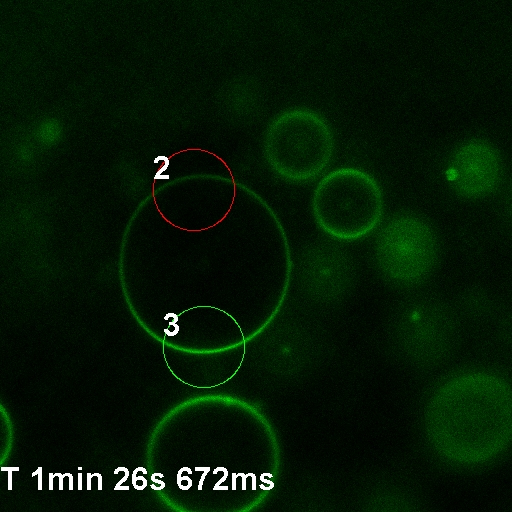

Supplement: S3 File — Zip file archive containing original photomicrographs obtained in Fluorescence Recovery After Photobleaching (FRAP) experiments. (ZIP) [file pone.0158729.s003.zip › PEO-PBD-NH2+ FRAP Slow/NH2 On Surface Reg Activatin 20us_pix 10 frame_C001T006.jpg]

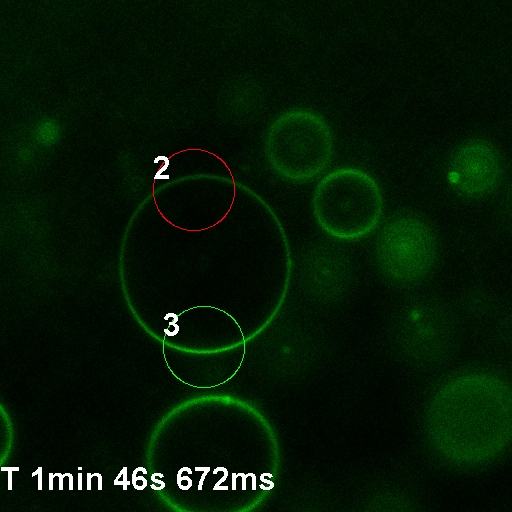

Supplement: S3 File — Zip file archive containing original photomicrographs obtained in Fluorescence Recovery After Photobleaching (FRAP) experiments. (ZIP) [file pone.0158729.s003.zip › PEO-PBD-NH2+ FRAP Slow/NH2 On Surface Reg Activatin 20us_pix 10 frame_C001T007.jpg]

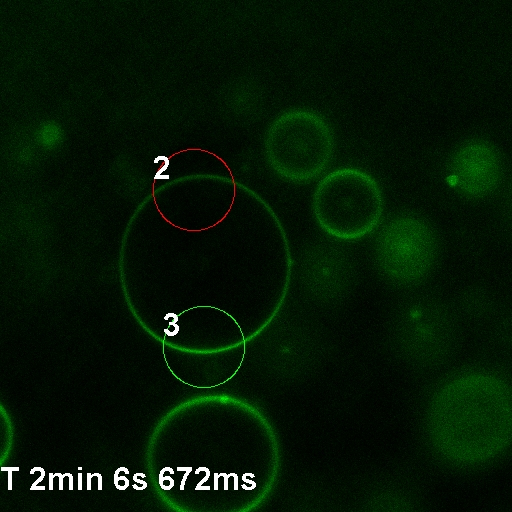

Supplement: S3 File — Zip file archive containing original photomicrographs obtained in Fluorescence Recovery After Photobleaching (FRAP) experiments. (ZIP) [file pone.0158729.s003.zip › PEO-PBD-NH2+ FRAP Slow/NH2 On Surface Reg Activatin 20us_pix 10 frame_C001T008.jpg]

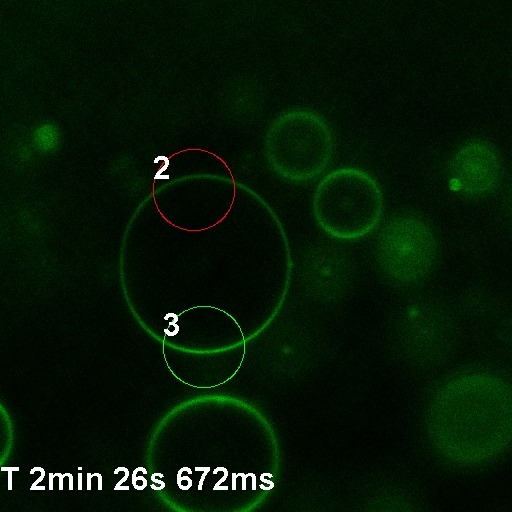

Supplement: S3 File — Zip file archive containing original photomicrographs obtained in Fluorescence Recovery After Photobleaching (FRAP) experiments. (ZIP) [file pone.0158729.s003.zip › PEO-PBD-NH2+ FRAP Slow/NH2 On Surface Reg Activatin 20us_pix 10 frame_C001T009.jpg]

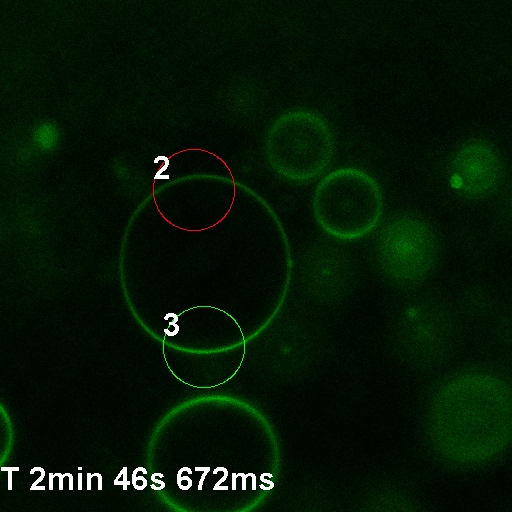

Supplement: S3 File — Zip file archive containing original photomicrographs obtained in Fluorescence Recovery After Photobleaching (FRAP) experiments. (ZIP) [file pone.0158729.s003.zip › PEO-PBD-NH2+ FRAP Slow/NH2 On Surface Reg Activatin 20us_pix 10 frame_C001T010.jpg]

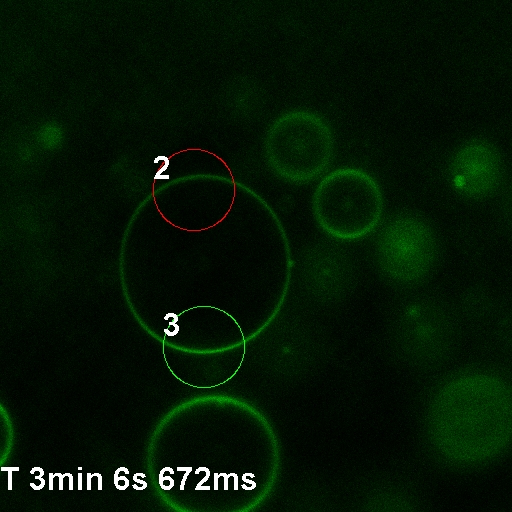

Supplement: S3 File — Zip file archive containing original photomicrographs obtained in Fluorescence Recovery After Photobleaching (FRAP) experiments. (ZIP) [file pone.0158729.s003.zip › PEO-PBD-NH2+ FRAP Slow/NH2 On Surface Reg Activatin 20us_pix 10 frame_C001T011.jpg]

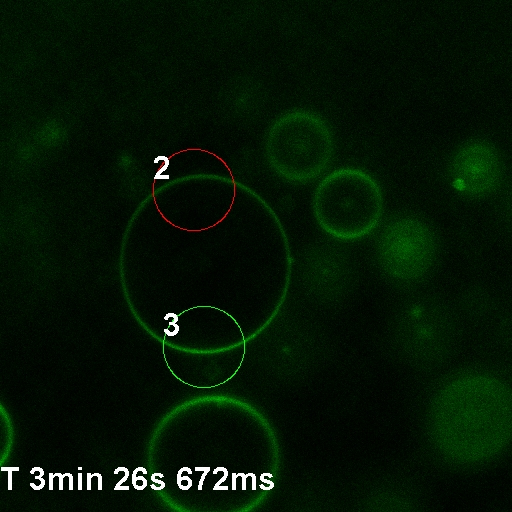

Supplement: S3 File — Zip file archive containing original photomicrographs obtained in Fluorescence Recovery After Photobleaching (FRAP) experiments. (ZIP) [file pone.0158729.s003.zip › PEO-PBD-NH2+ FRAP Slow/NH2 On Surface Reg Activatin 20us_pix 10 frame_C001T012.jpg]

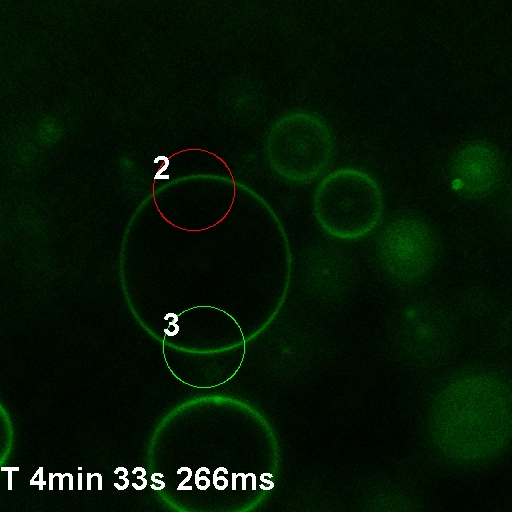

Supplement: S3 File — Zip file archive containing original photomicrographs obtained in Fluorescence Recovery After Photobleaching (FRAP) experiments. (ZIP) [file pone.0158729.s003.zip › PEO-PBD-NH2+ FRAP Slow/NH2 On Surface Reg Activatin 20us_pix 10 frame_C001T013.jpg]

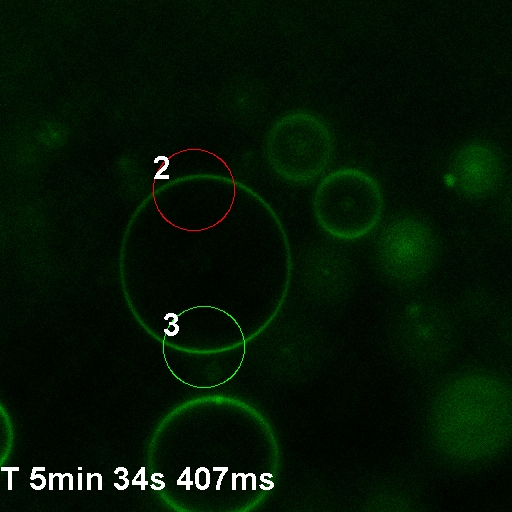

Supplement: S3 File — Zip file archive containing original photomicrographs obtained in Fluorescence Recovery After Photobleaching (FRAP) experiments. (ZIP) [file pone.0158729.s003.zip › PEO-PBD-NH2+ FRAP Slow/NH2 On Surface Reg Activatin 20us_pix 10 frame_C001T014.jpg]

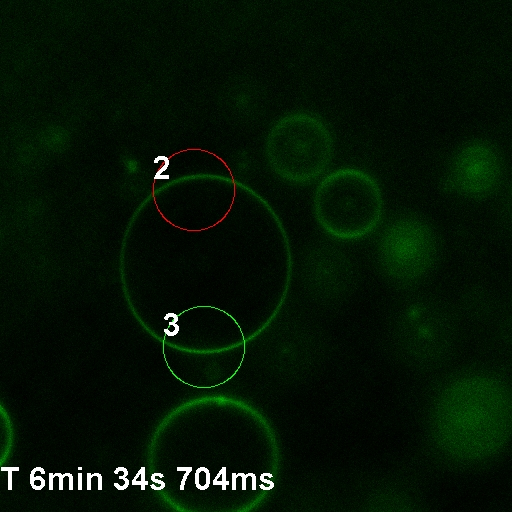

Supplement: S3 File — Zip file archive containing original photomicrographs obtained in Fluorescence Recovery After Photobleaching (FRAP) experiments. (ZIP) [file pone.0158729.s003.zip › PEO-PBD-NH2+ FRAP Slow/NH2 On Surface Reg Activatin 20us_pix 10 frame_C001T015.jpg]

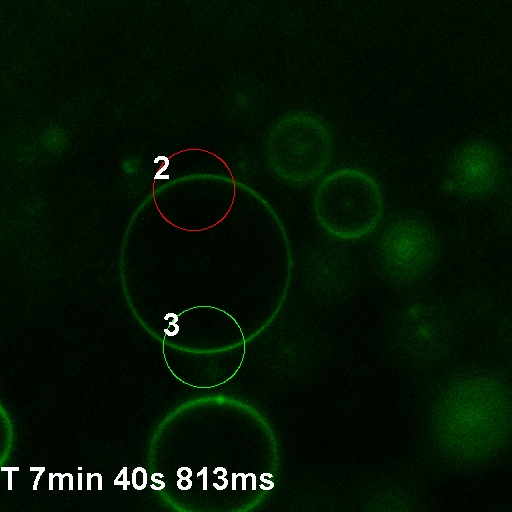

Supplement: S3 File — Zip file archive containing original photomicrographs obtained in Fluorescence Recovery After Photobleaching (FRAP) experiments. (ZIP) [file pone.0158729.s003.zip › PEO-PBD-NH2+ FRAP Slow/NH2 On Surface Reg Activatin 20us_pix 10 frame_C001T016.jpg]

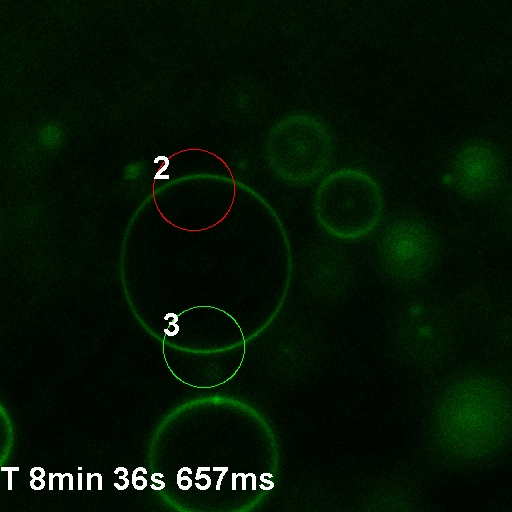

Supplement: S3 File — Zip file archive containing original photomicrographs obtained in Fluorescence Recovery After Photobleaching (FRAP) experiments. (ZIP) [file pone.0158729.s003.zip › PEO-PBD-NH2+ FRAP Slow/NH2 On Surface Reg Activatin 20us_pix 10 frame_C001T017.jpg]

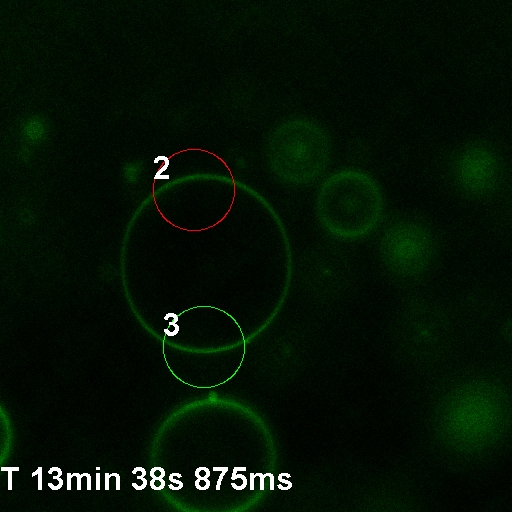

Supplement: S3 File — Zip file archive containing original photomicrographs obtained in Fluorescence Recovery After Photobleaching (FRAP) experiments. (ZIP) [file pone.0158729.s003.zip › PEO-PBD-NH2+ FRAP Slow/NH2 On Surface Reg Activatin 20us_pix 10 frame_C001T018.jpg]

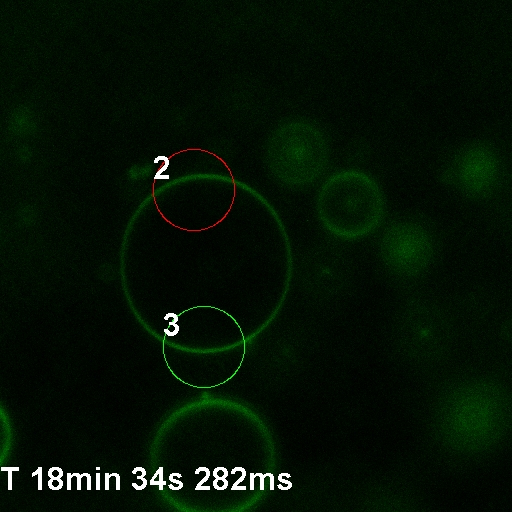

Supplement: S3 File — Zip file archive containing original photomicrographs obtained in Fluorescence Recovery After Photobleaching (FRAP) experiments. (ZIP) [file pone.0158729.s003.zip › PEO-PBD-NH2+ FRAP Slow/NH2 On Surface Reg Activatin 20us_pix 10 frame_C001T019.jpg]

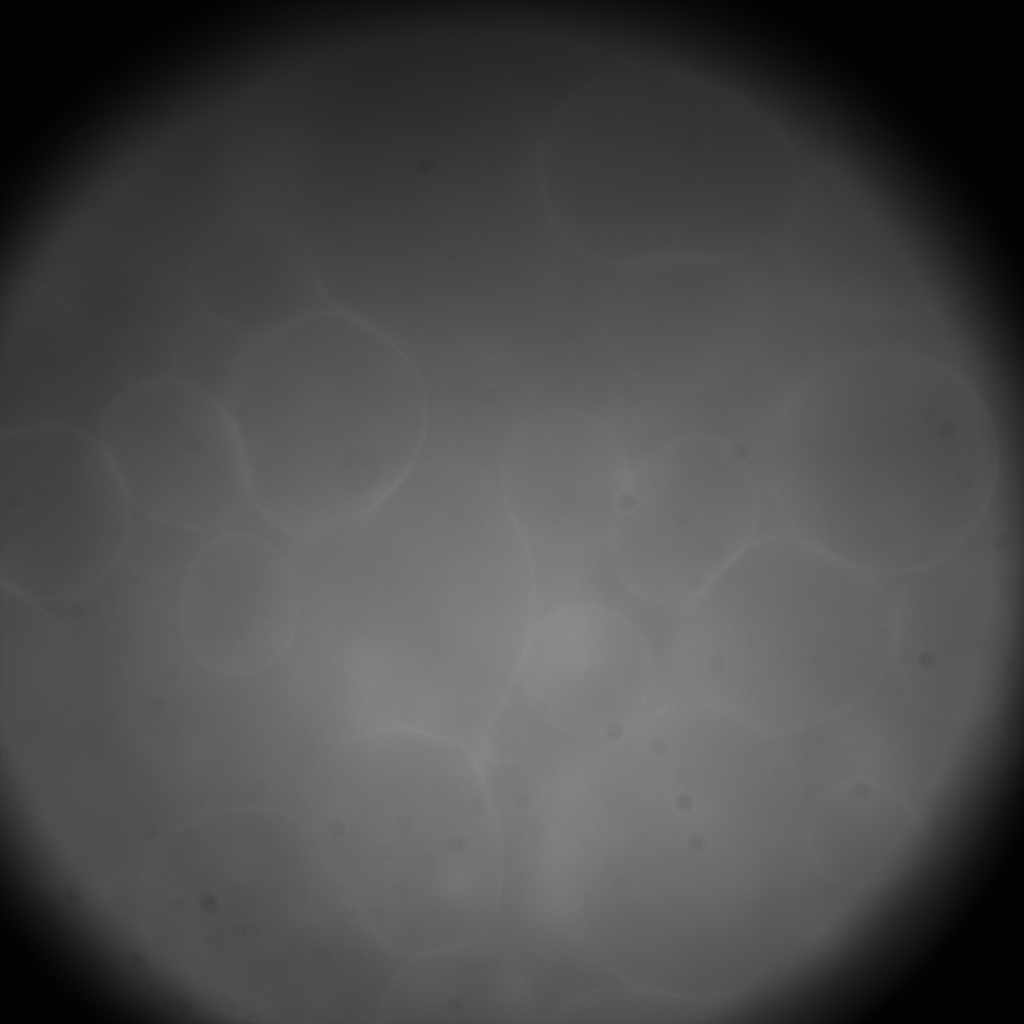

Supplement: S4 File — Zip file archive containing original photomicrographs of polymersomes formed following rehydration with sucrose and on gels prepared with sucrose. (ZIP) [file pone.0158729.s004.zip › Sucrose Rehydration on Gel Prepared in Sucrose/Image_5028_20150330_161613.tif]

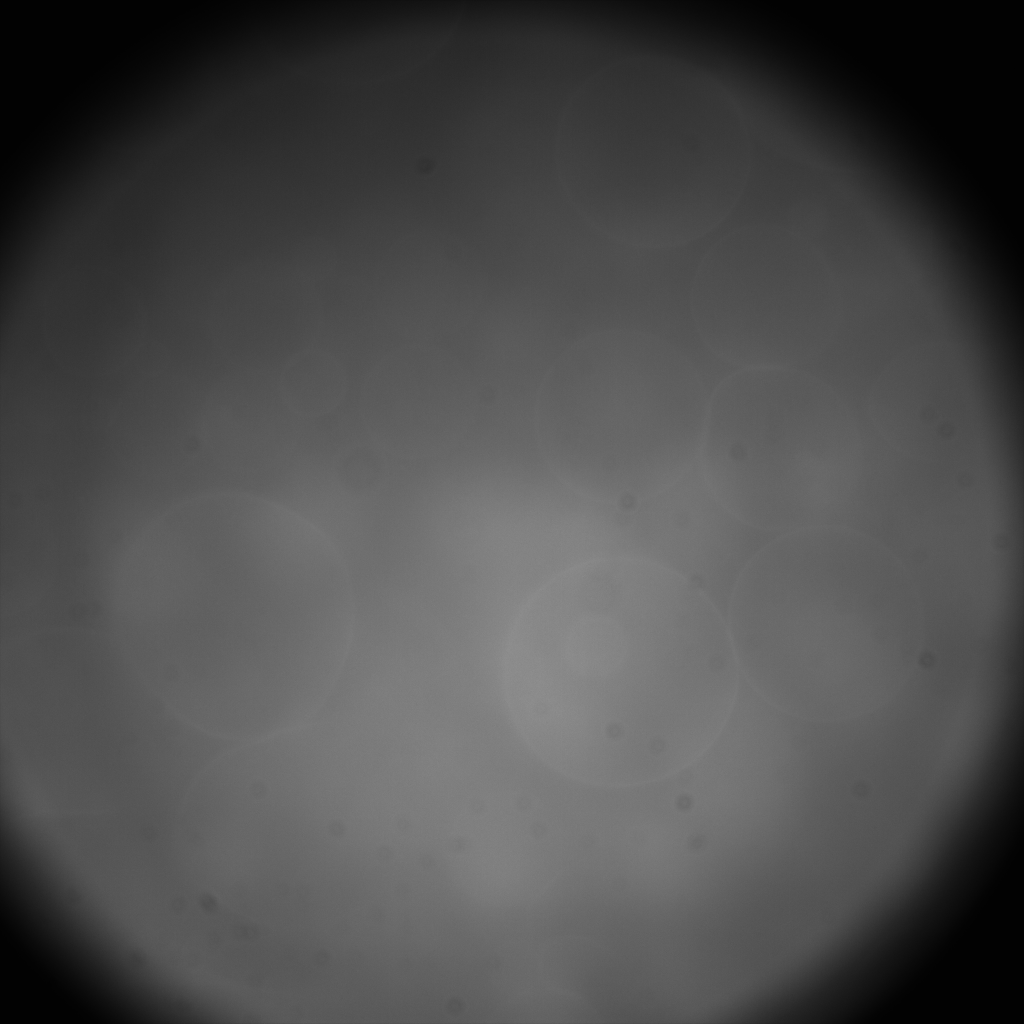

Supplement: S4 File — Zip file archive containing original photomicrographs of polymersomes formed following rehydration with sucrose and on gels prepared with sucrose. (ZIP) [file pone.0158729.s004.zip › Sucrose Rehydration on Gel Prepared in Sucrose/Image_5029_20150330_161620.tif]

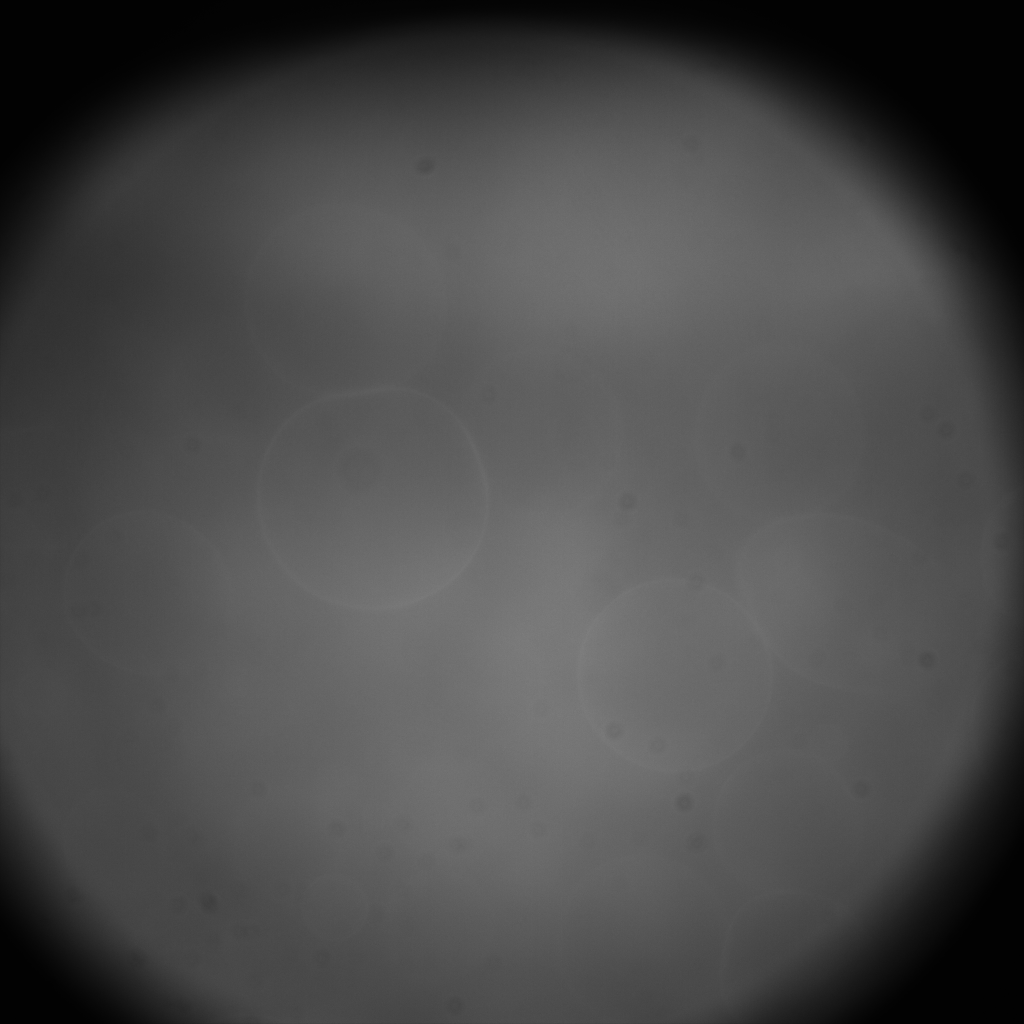

Supplement: S4 File — Zip file archive containing original photomicrographs of polymersomes formed following rehydration with sucrose and on gels prepared with sucrose. (ZIP) [file pone.0158729.s004.zip › Sucrose Rehydration on Gel Prepared in Sucrose/Image_5030_20150330_161627.tif]

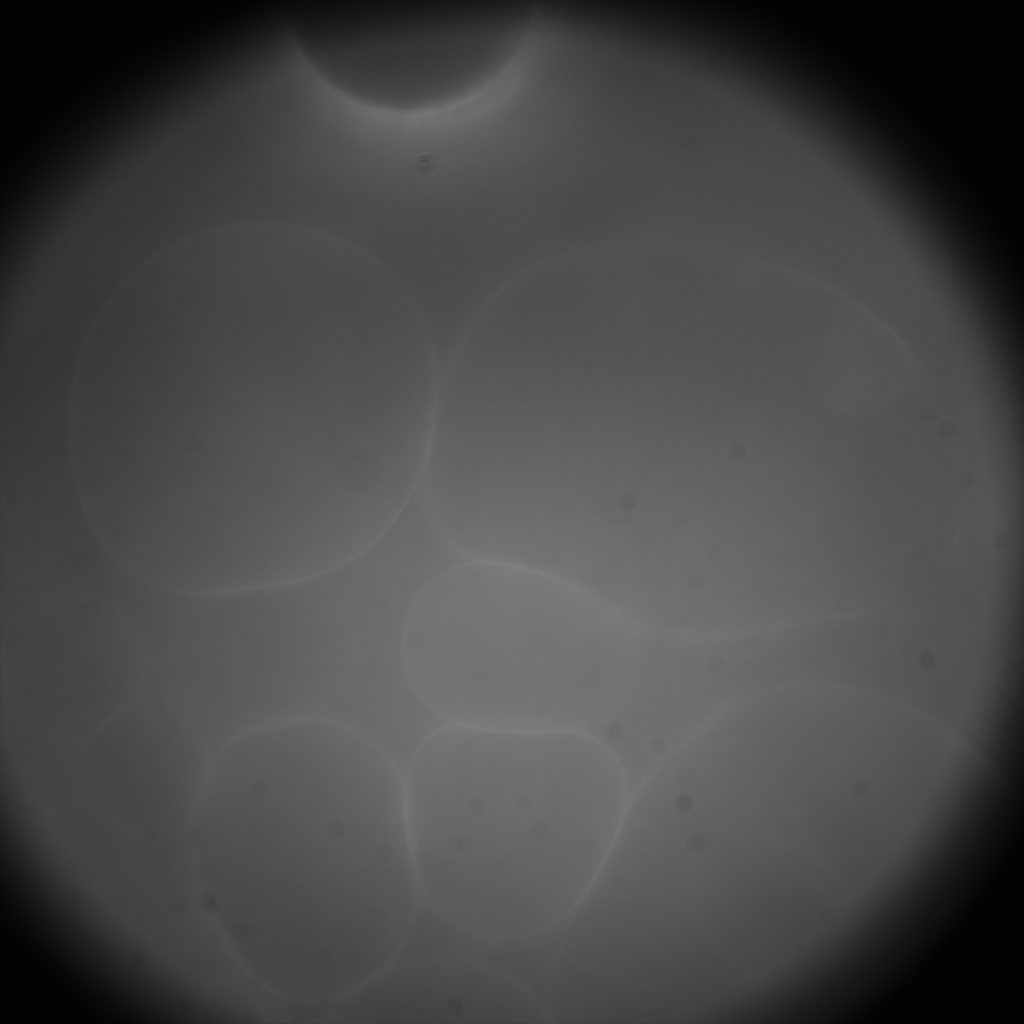

Supplement: S4 File — Zip file archive containing original photomicrographs of polymersomes formed following rehydration with sucrose and on gels prepared with sucrose. (ZIP) [file pone.0158729.s004.zip › Sucrose Rehydration on Gel Prepared in Sucrose/Image_5031_20150330_161638.tif]

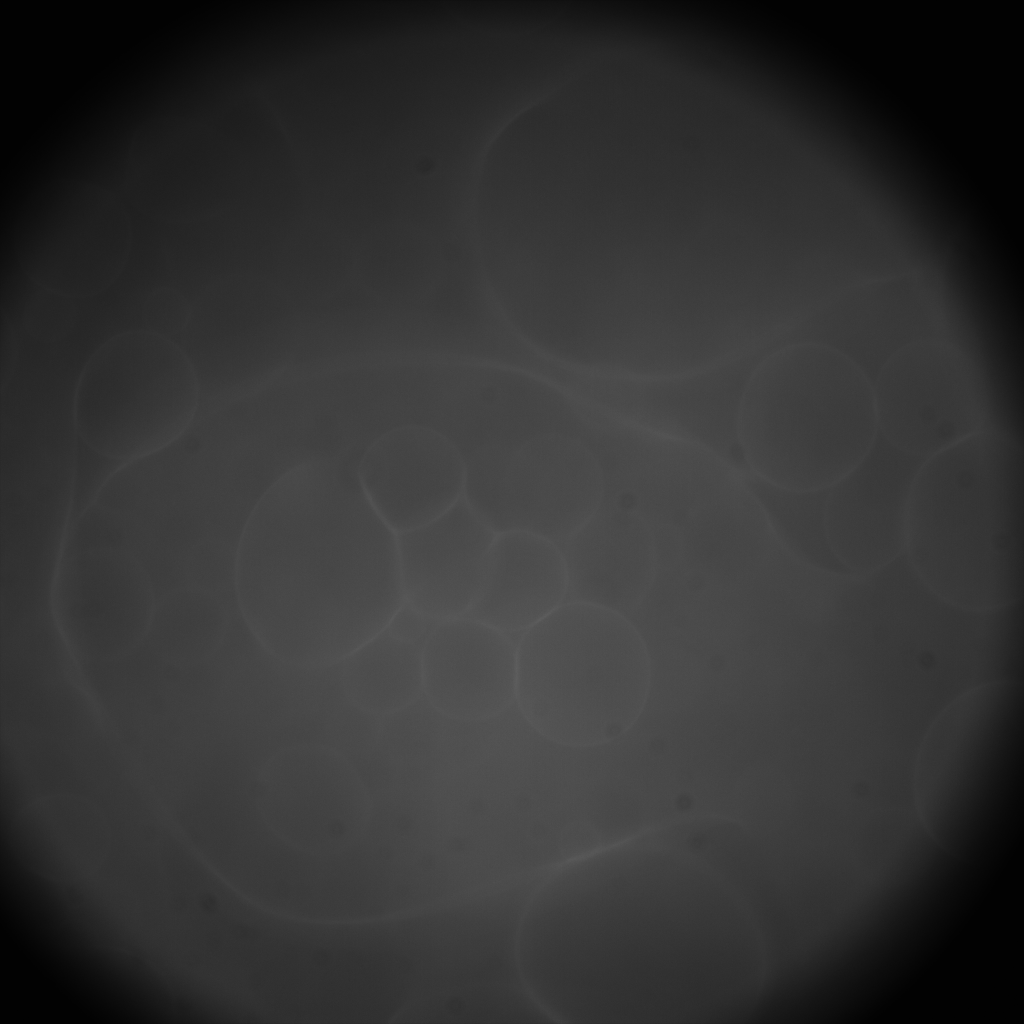

Supplement: S4 File — Zip file archive containing original photomicrographs of polymersomes formed following rehydration with sucrose and on gels prepared with sucrose. (ZIP) [file pone.0158729.s004.zip › Sucrose Rehydration on Gel Prepared in Sucrose/Image_5032_20150330_161651.tif]

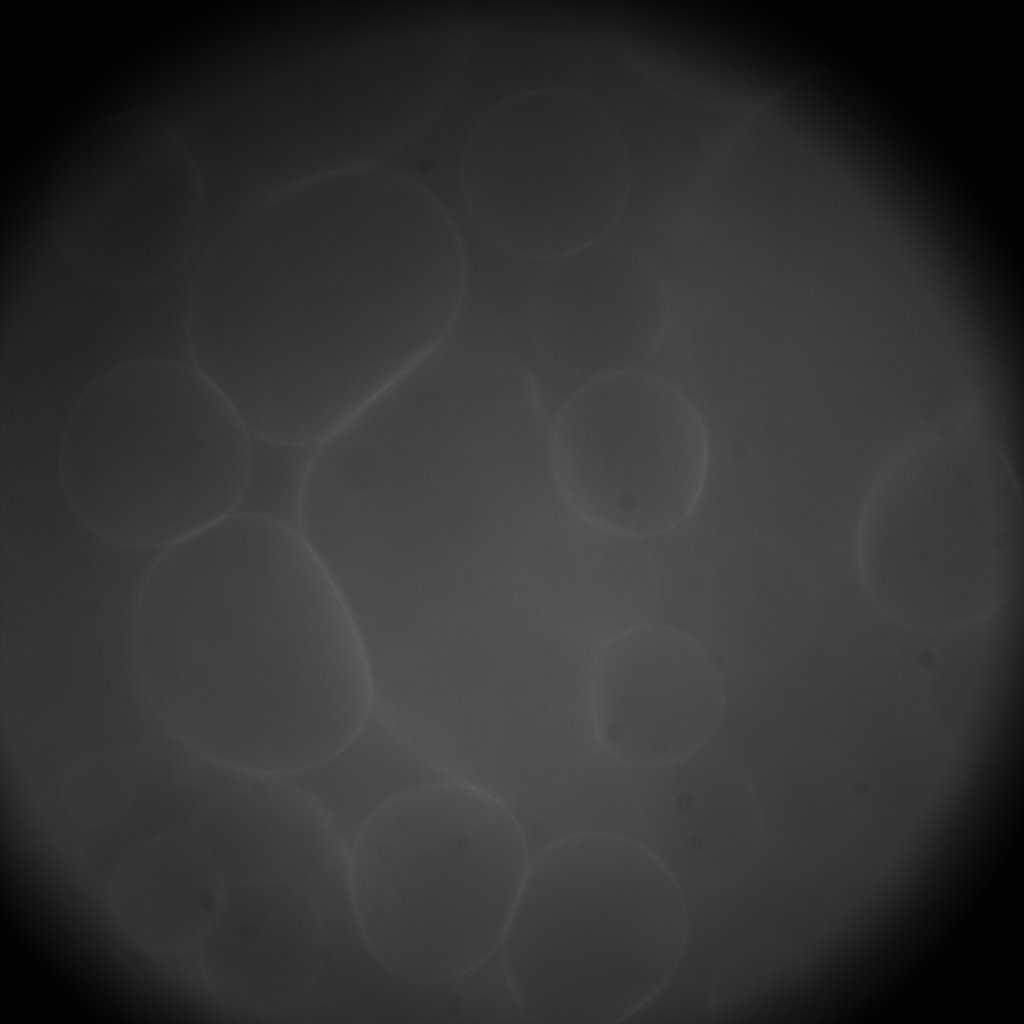

Supplement: S4 File — Zip file archive containing original photomicrographs of polymersomes formed following rehydration with sucrose and on gels prepared with sucrose. (ZIP) [file pone.0158729.s004.zip › Sucrose Rehydration on Gel Prepared in Sucrose/Image_5033_20150330_161705.tif]

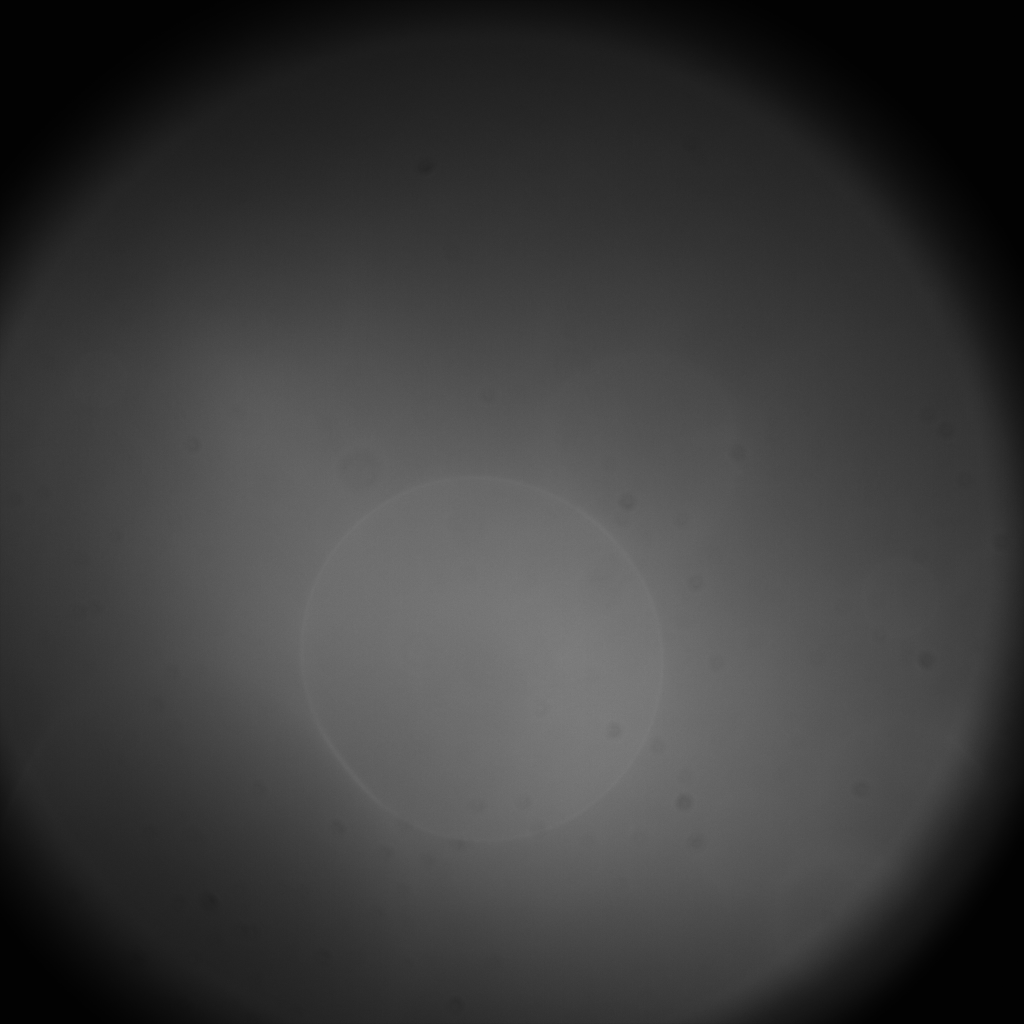

Supplement: S4 File — Zip file archive containing original photomicrographs of polymersomes formed following rehydration with sucrose and on gels prepared with sucrose. (ZIP) [file pone.0158729.s004.zip › Sucrose Rehydration on Gel Prepared in Sucrose/Image_5034_20150330_161728.tif]

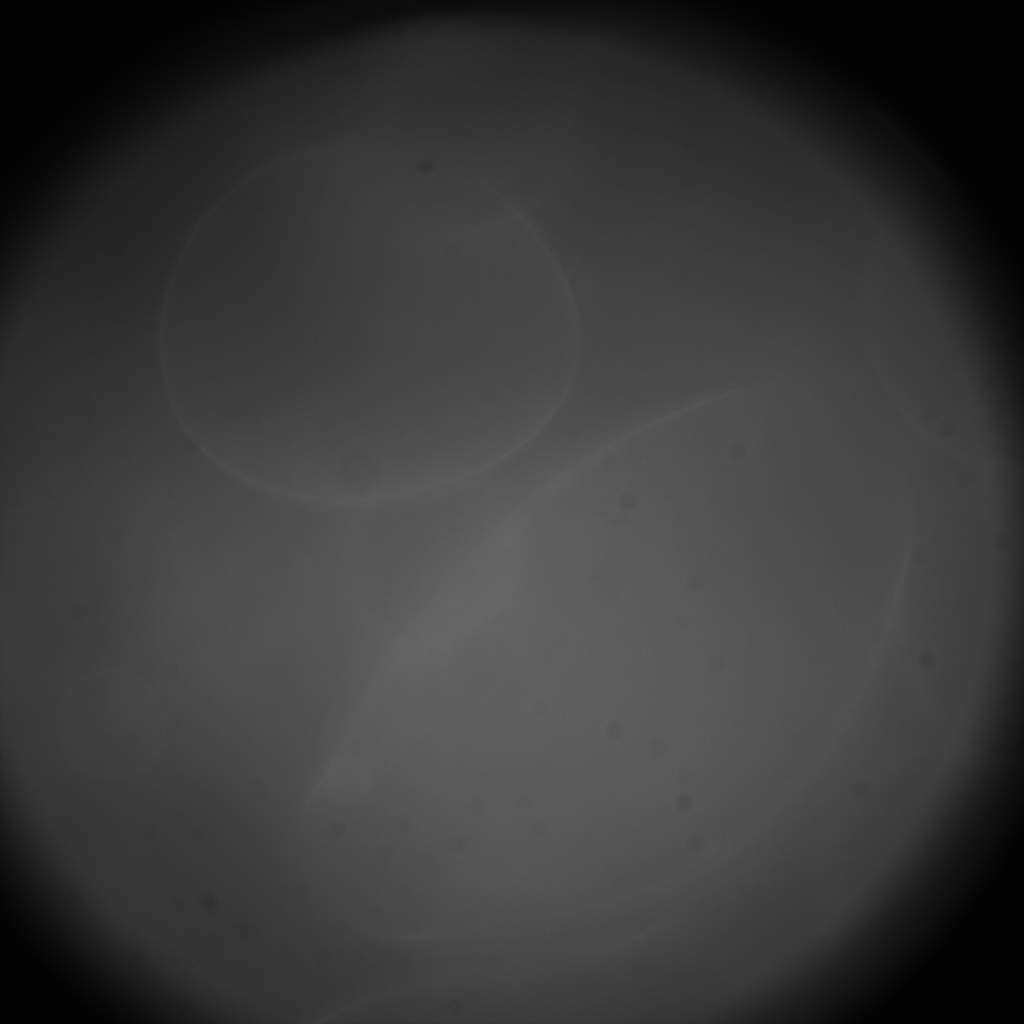

Supplement: S4 File — Zip file archive containing original photomicrographs of polymersomes formed following rehydration with sucrose and on gels prepared with sucrose. (ZIP) [file pone.0158729.s004.zip › Sucrose Rehydration on Gel Prepared in Sucrose/Image_5035_20150330_161747.tif]

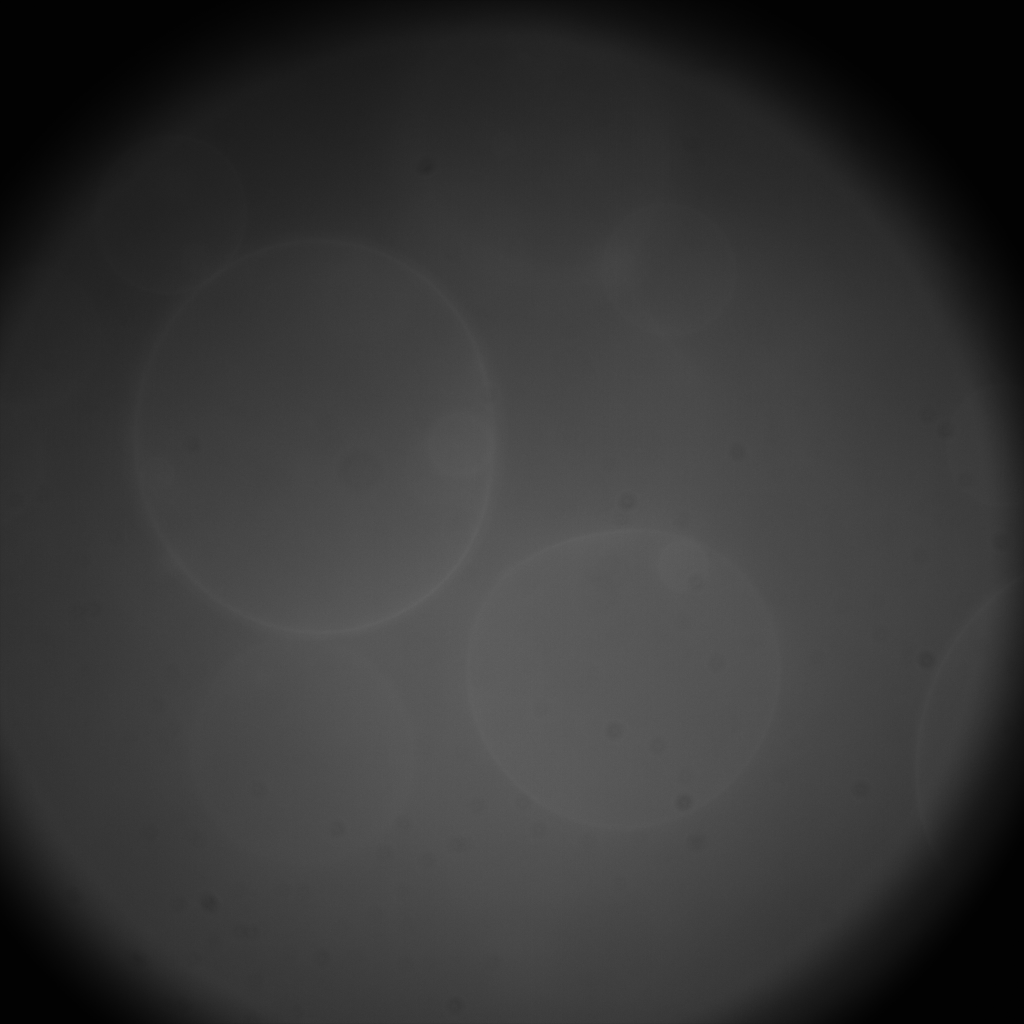

Supplement: S4 File — Zip file archive containing original photomicrographs of polymersomes formed following rehydration with sucrose and on gels prepared with sucrose. (ZIP) [file pone.0158729.s004.zip › Sucrose Rehydration on Gel Prepared in Sucrose/Image_5036_20150330_161853.tif]

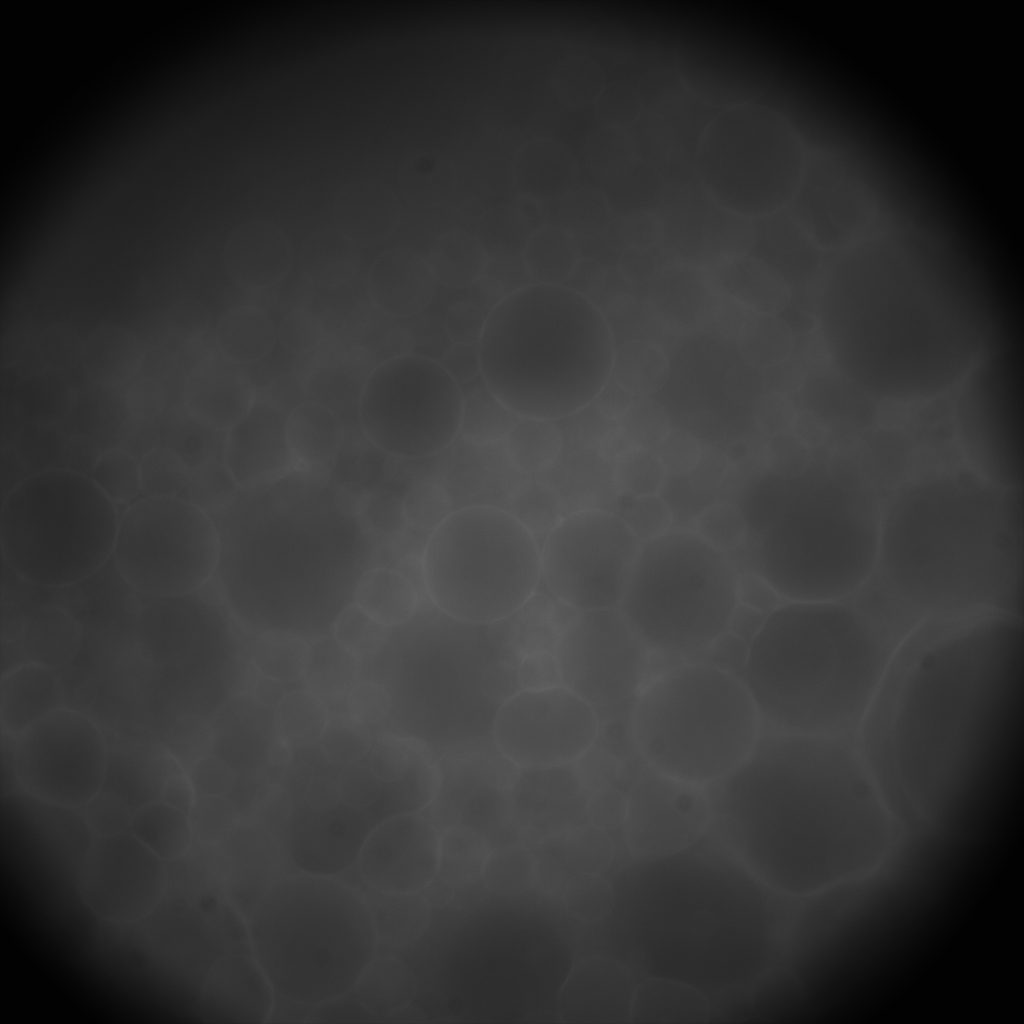

Supplement: S4 File — Zip file archive containing original photomicrographs of polymersomes formed following rehydration with sucrose and on gels prepared with sucrose. (ZIP) [file pone.0158729.s004.zip › Sucrose Rehydration on Gel Prepared in Sucrose/Image_5049_20150330_163434.tif]

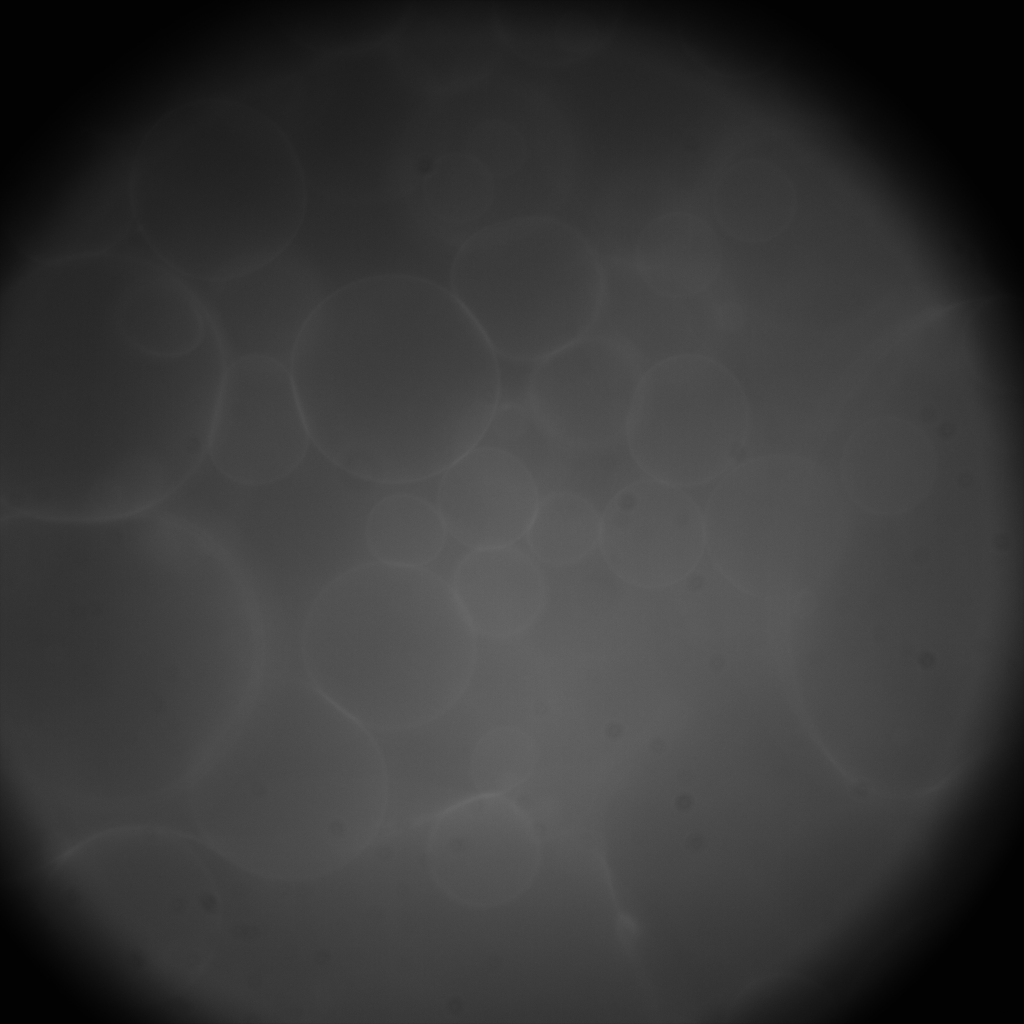

Supplement: S4 File — Zip file archive containing original photomicrographs of polymersomes formed following rehydration with sucrose and on gels prepared with sucrose. (ZIP) [file pone.0158729.s004.zip › Sucrose Rehydration on Gel Prepared in Sucrose/Image_5050_20150330_163448.tif]

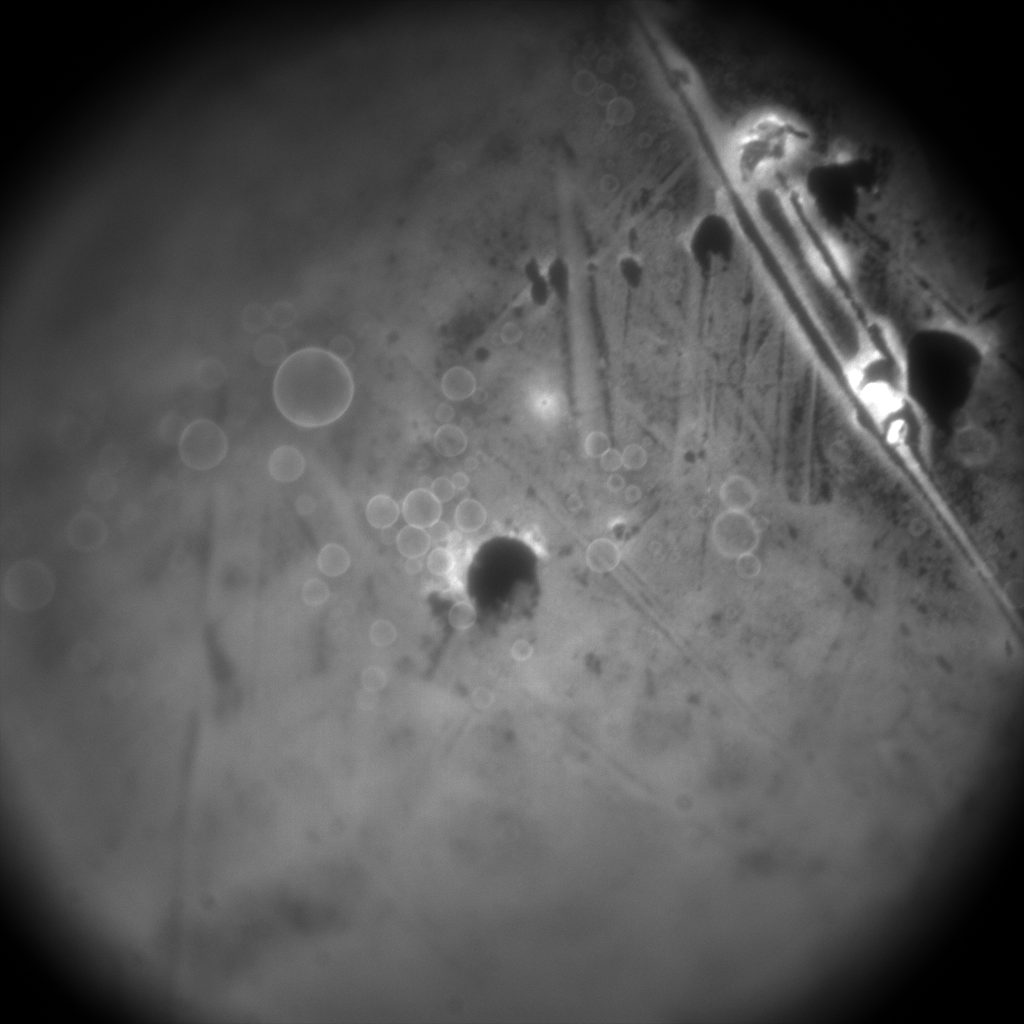

Supplement: S4 File — Zip file archive containing original photomicrographs of polymersomes formed following rehydration with sucrose and on gels prepared with sucrose. (ZIP) [file pone.0158729.s004.zip › Sucrose Rehydration on Gel Prepared in Sucrose/Image_5051_20150330_163602.tif]

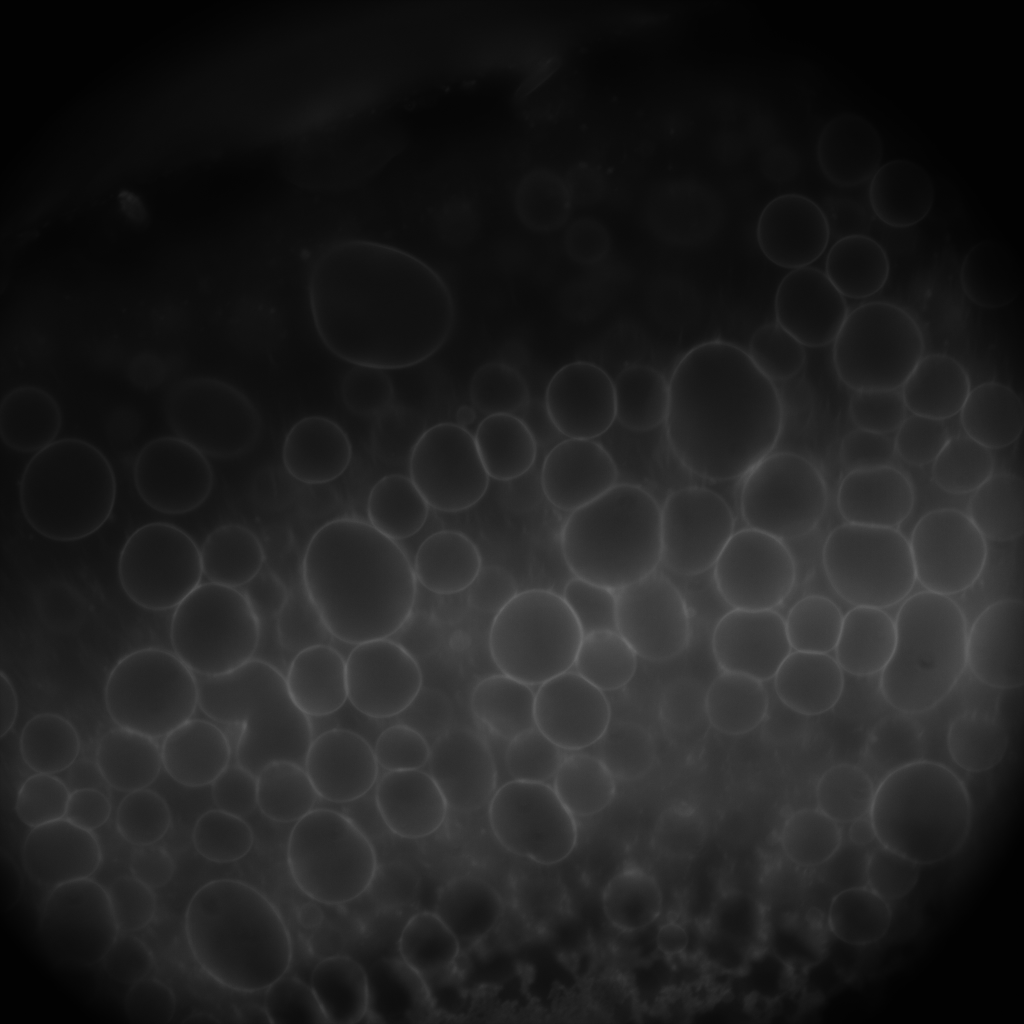

Supplement: S4 File — Zip file archive containing original photomicrographs of polymersomes formed following rehydration with sucrose and on gels prepared with sucrose. (ZIP) [file pone.0158729.s004.zip › Sucrose Rehydration on Gel Prepared in Sucrose/Image_5052_20150330_163653.tif]

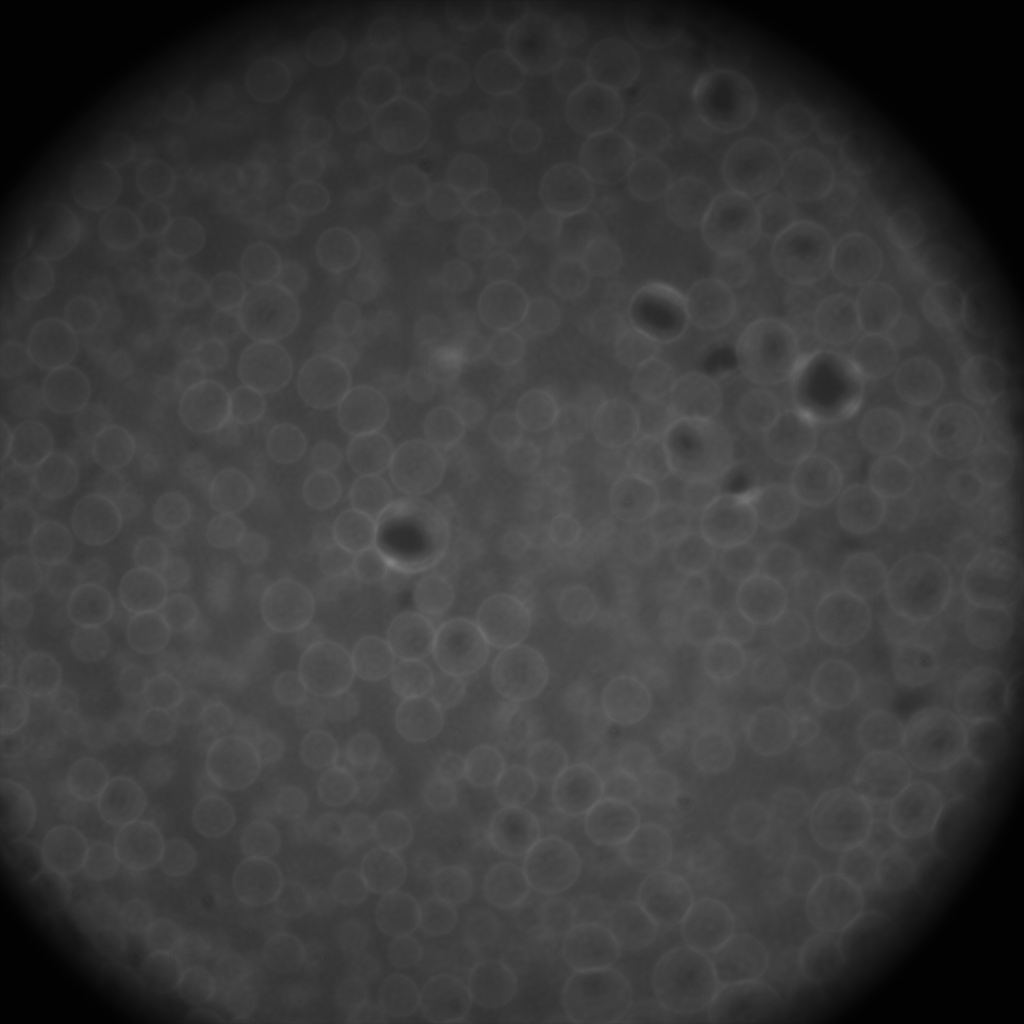

Supplement: S5 File — Zip file archive containing original photomicrographs of polymersomes formed following rehydration with sucrose and on gels prepared with water. (ZIP) [file pone.0158729.s005.zip › Sucrose Rehydration on Gel Prepared in Water/Image_4404_20150316_154231.tif]

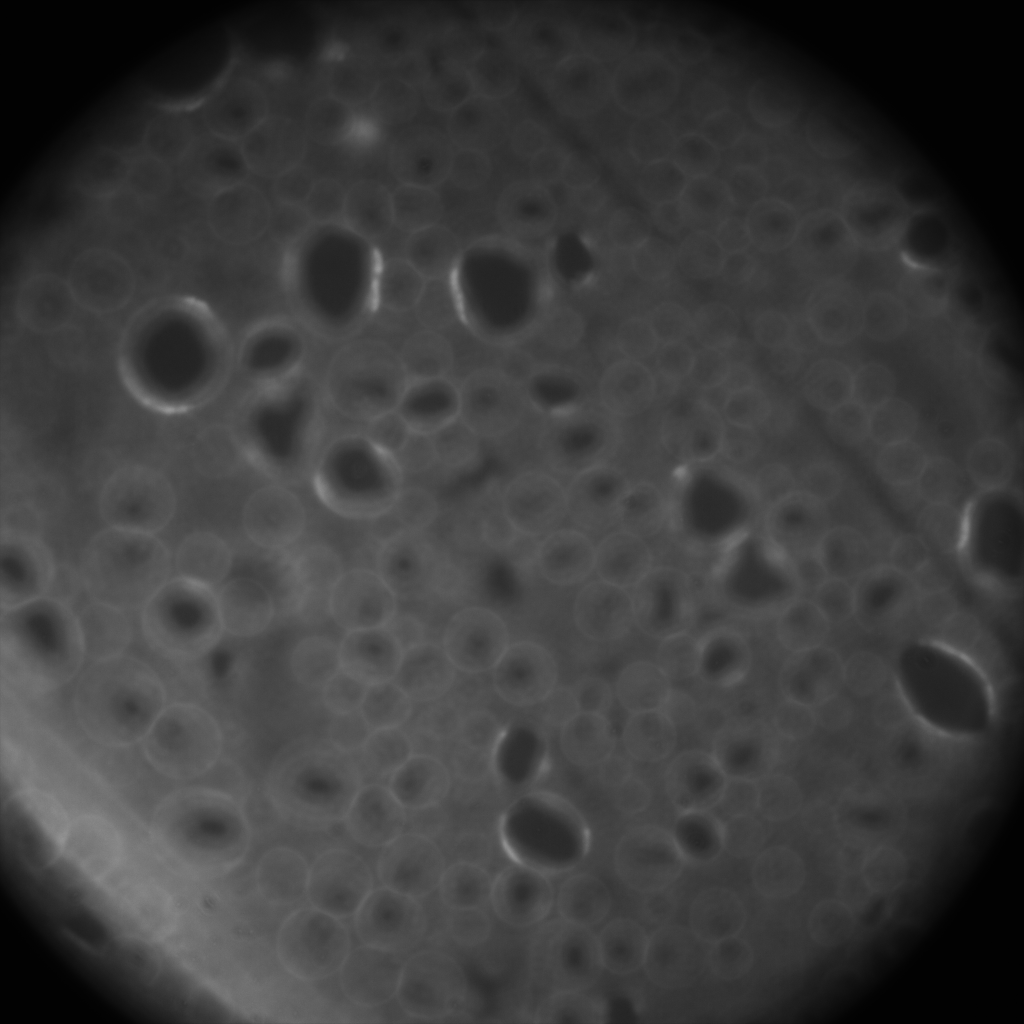

Supplement: S5 File — Zip file archive containing original photomicrographs of polymersomes formed following rehydration with sucrose and on gels prepared with water. (ZIP) [file pone.0158729.s005.zip › Sucrose Rehydration on Gel Prepared in Water/Image_4405_20150316_154246.tif]

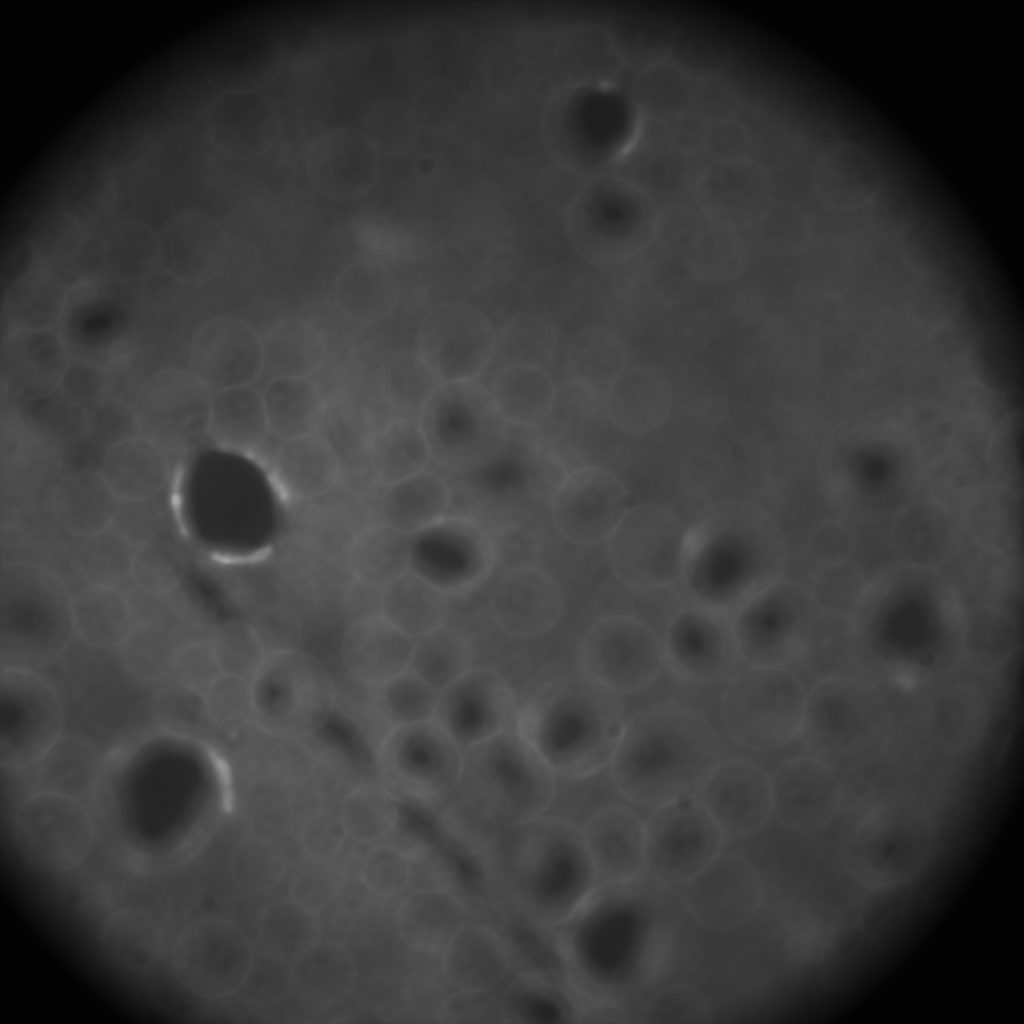

Supplement: S5 File — Zip file archive containing original photomicrographs of polymersomes formed following rehydration with sucrose and on gels prepared with water. (ZIP) [file pone.0158729.s005.zip › Sucrose Rehydration on Gel Prepared in Water/Image_4406_20150316_154312.tif]

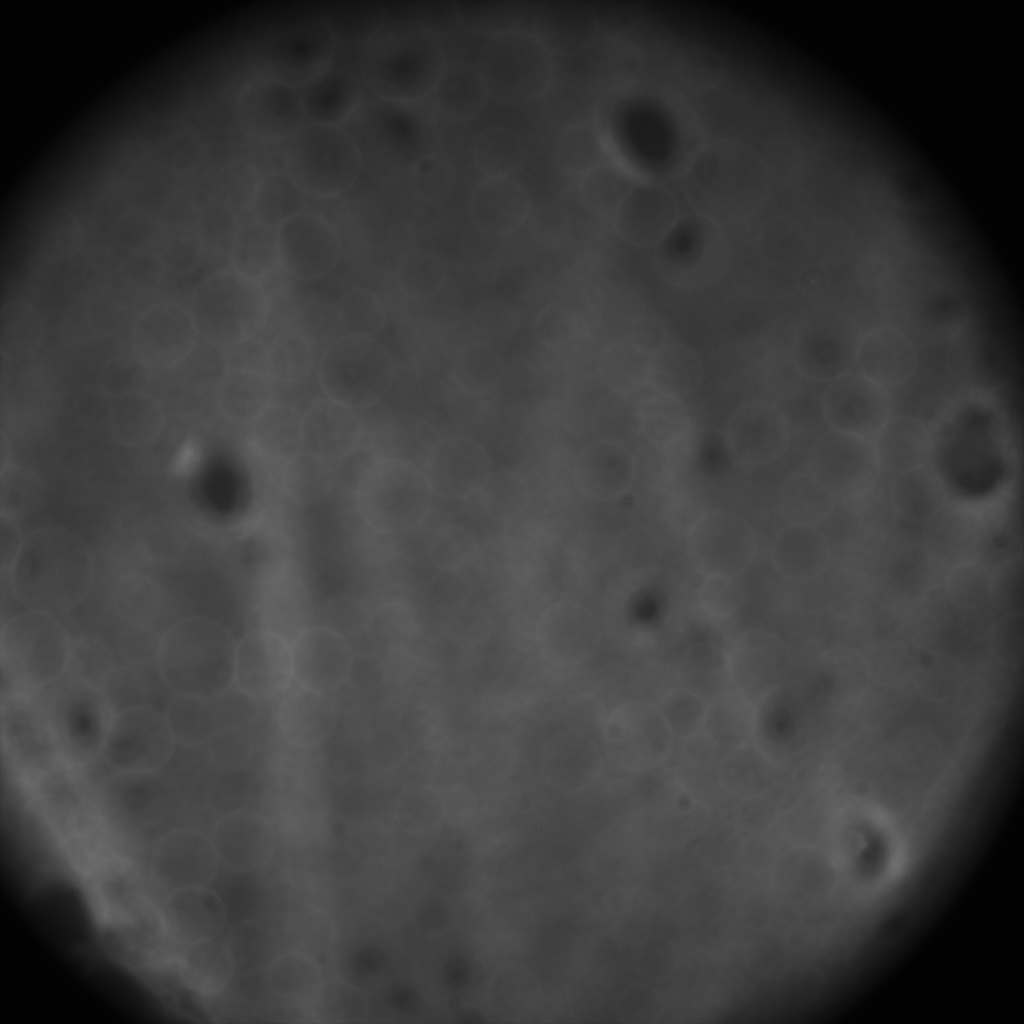

Supplement: S5 File — Zip file archive containing original photomicrographs of polymersomes formed following rehydration with sucrose and on gels prepared with water. (ZIP) [file pone.0158729.s005.zip › Sucrose Rehydration on Gel Prepared in Water/Image_4407_20150316_154320.tif]

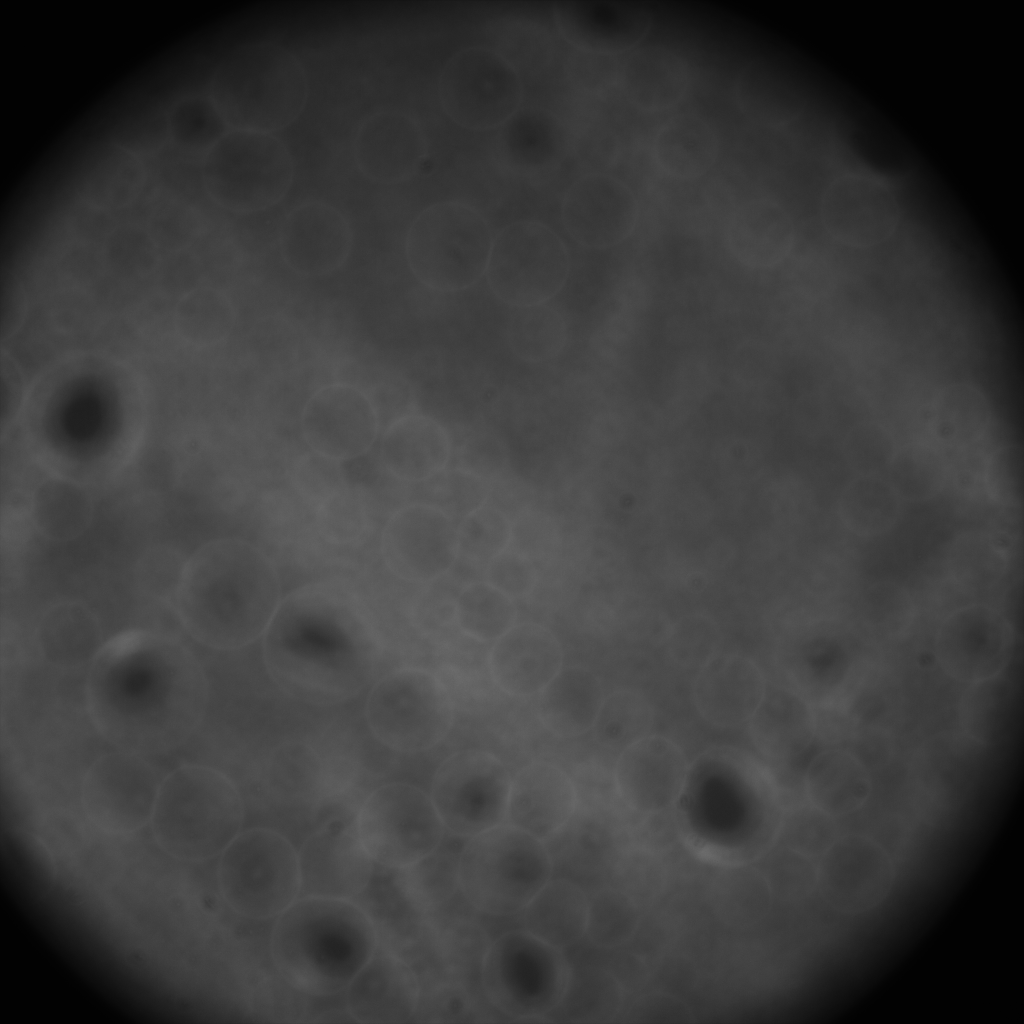

Supplement: S5 File — Zip file archive containing original photomicrographs of polymersomes formed following rehydration with sucrose and on gels prepared with water. (ZIP) [file pone.0158729.s005.zip › Sucrose Rehydration on Gel Prepared in Water/Image_4408_20150316_154333.tif]

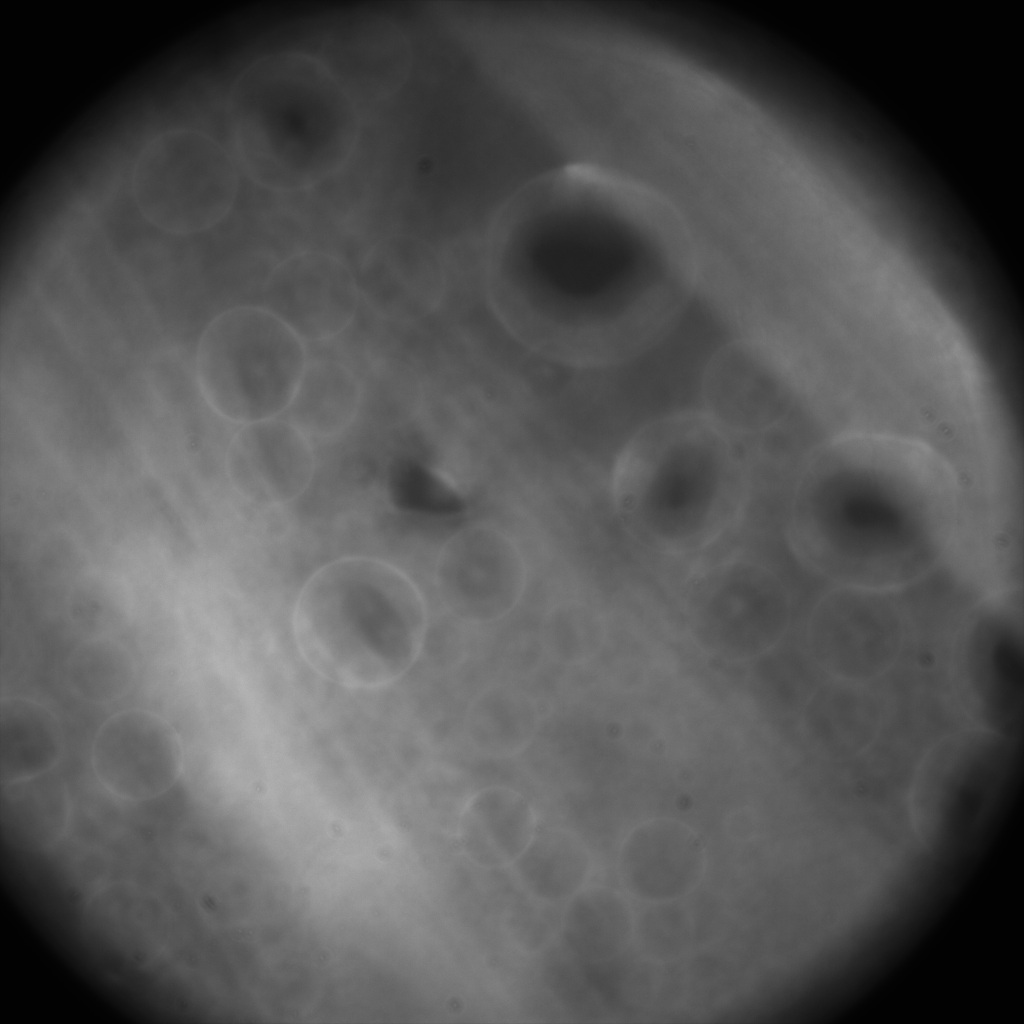

Supplement: S5 File — Zip file archive containing original photomicrographs of polymersomes formed following rehydration with sucrose and on gels prepared with water. (ZIP) [file pone.0158729.s005.zip › Sucrose Rehydration on Gel Prepared in Water/Image_4409_20150316_154342.tif]

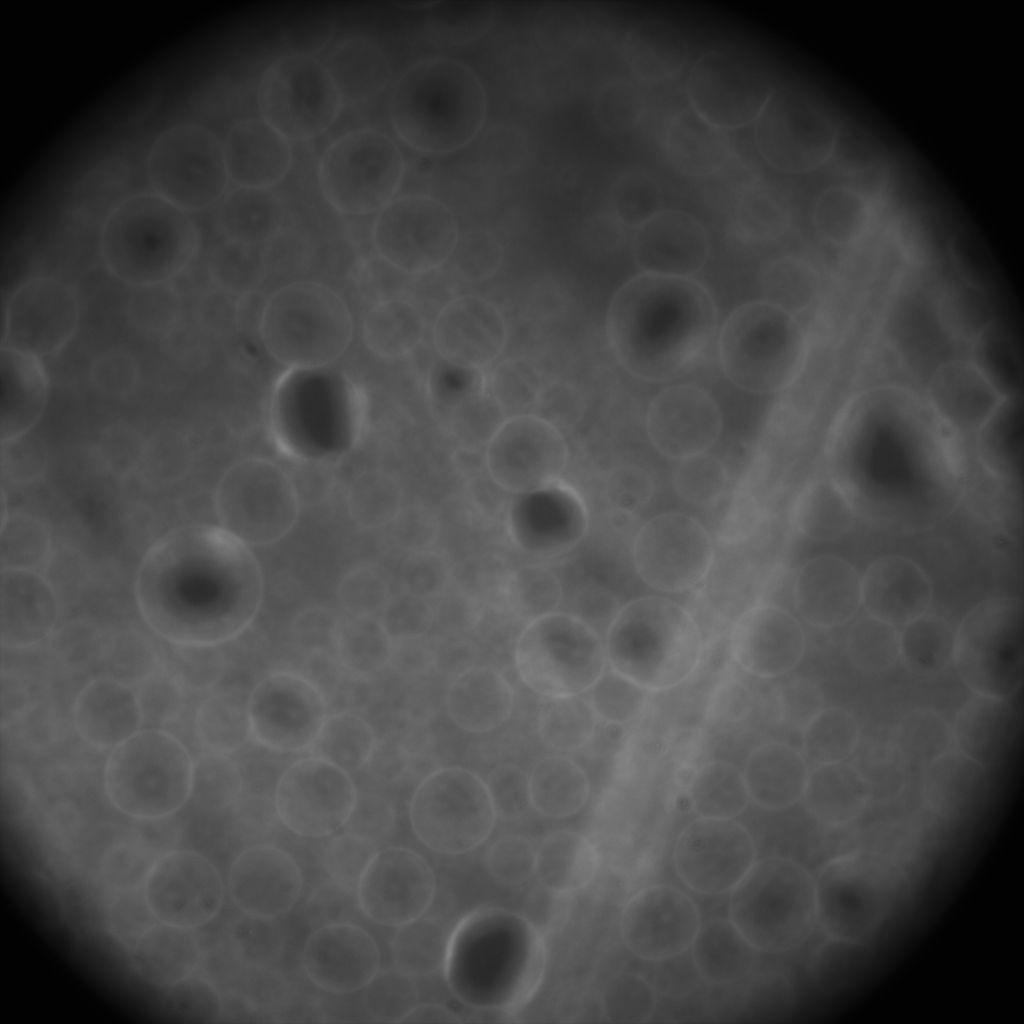

Supplement: S5 File — Zip file archive containing original photomicrographs of polymersomes formed following rehydration with sucrose and on gels prepared with water. (ZIP) [file pone.0158729.s005.zip › Sucrose Rehydration on Gel Prepared in Water/Image_4410_20150316_154349.tif]

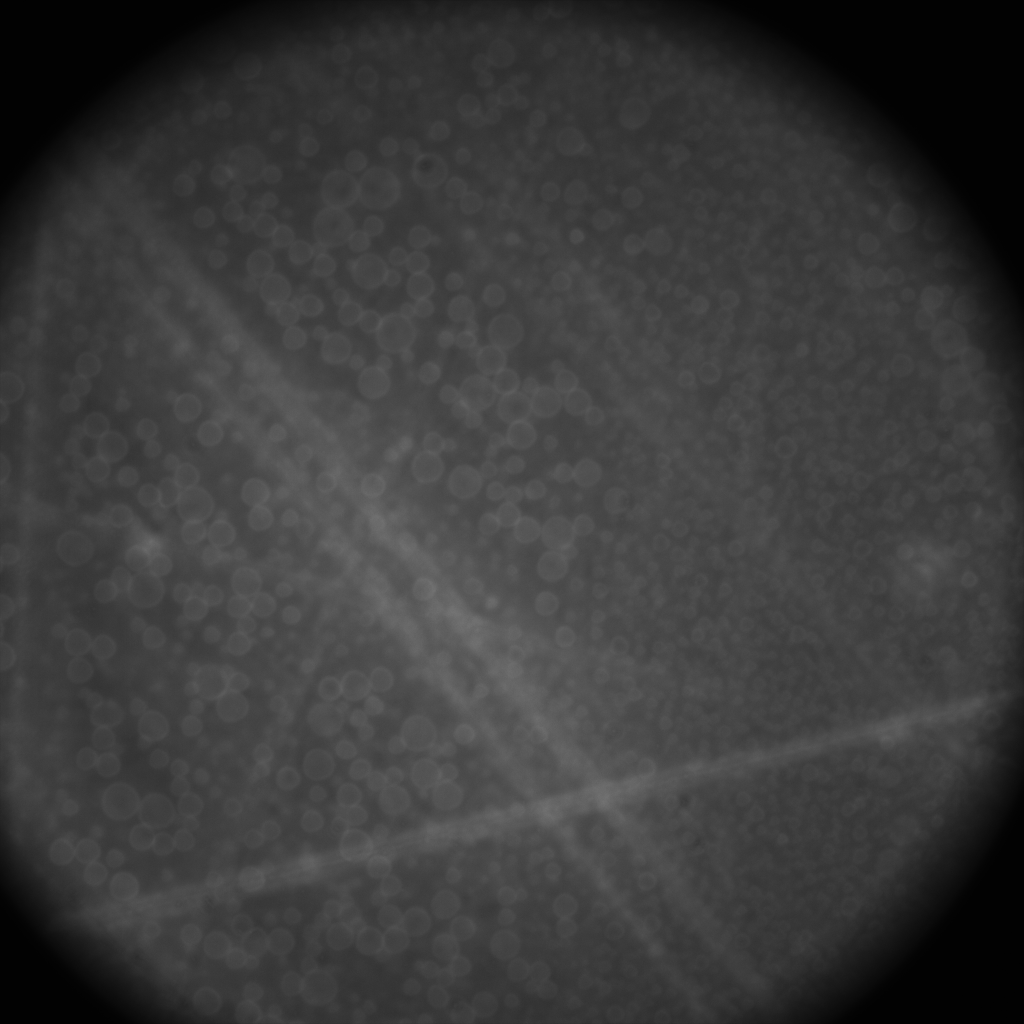

Supplement: S5 File — Zip file archive containing original photomicrographs of polymersomes formed following rehydration with sucrose and on gels prepared with water. (ZIP) [file pone.0158729.s005.zip › Sucrose Rehydration on Gel Prepared in Water/Image_4411_20150316_154405.tif]

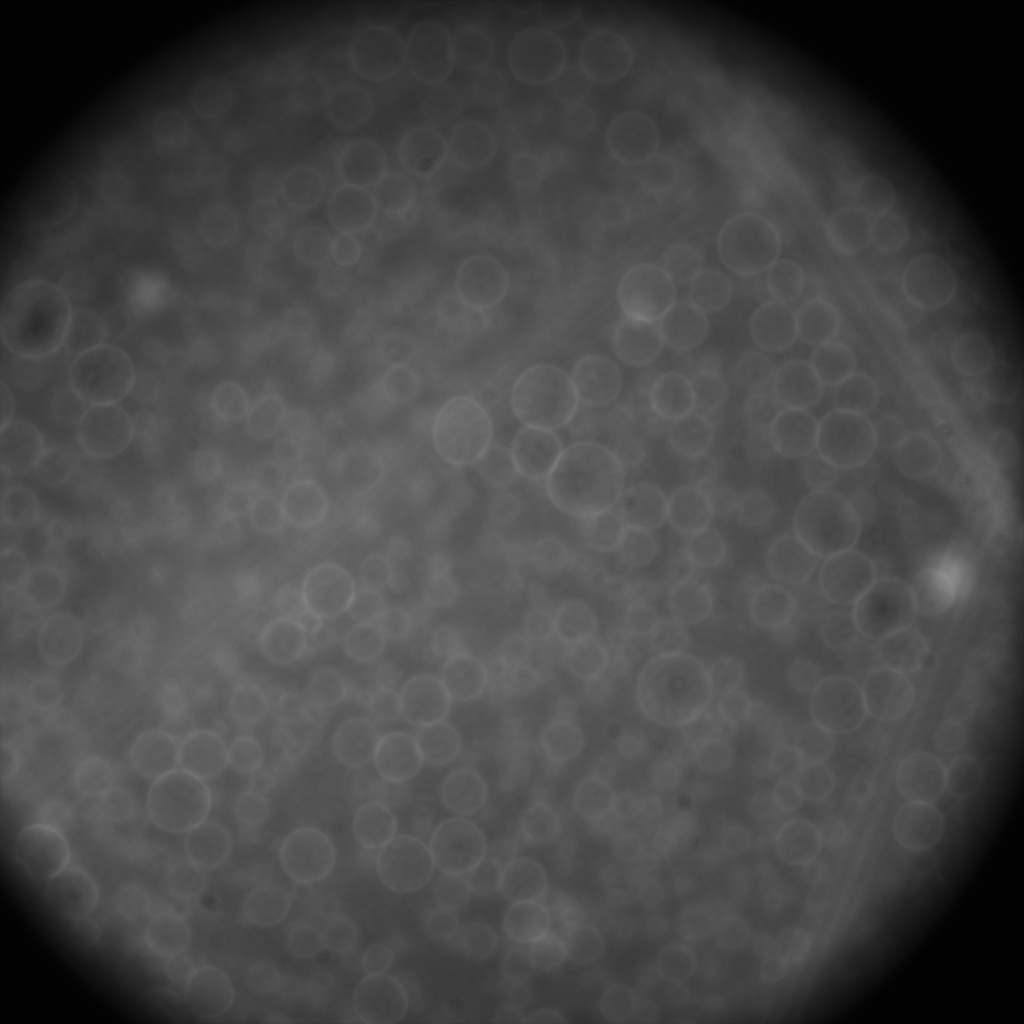

Supplement: S5 File — Zip file archive containing original photomicrographs of polymersomes formed following rehydration with sucrose and on gels prepared with water. (ZIP) [file pone.0158729.s005.zip › Sucrose Rehydration on Gel Prepared in Water/Image_4412_20150316_154417.tif]

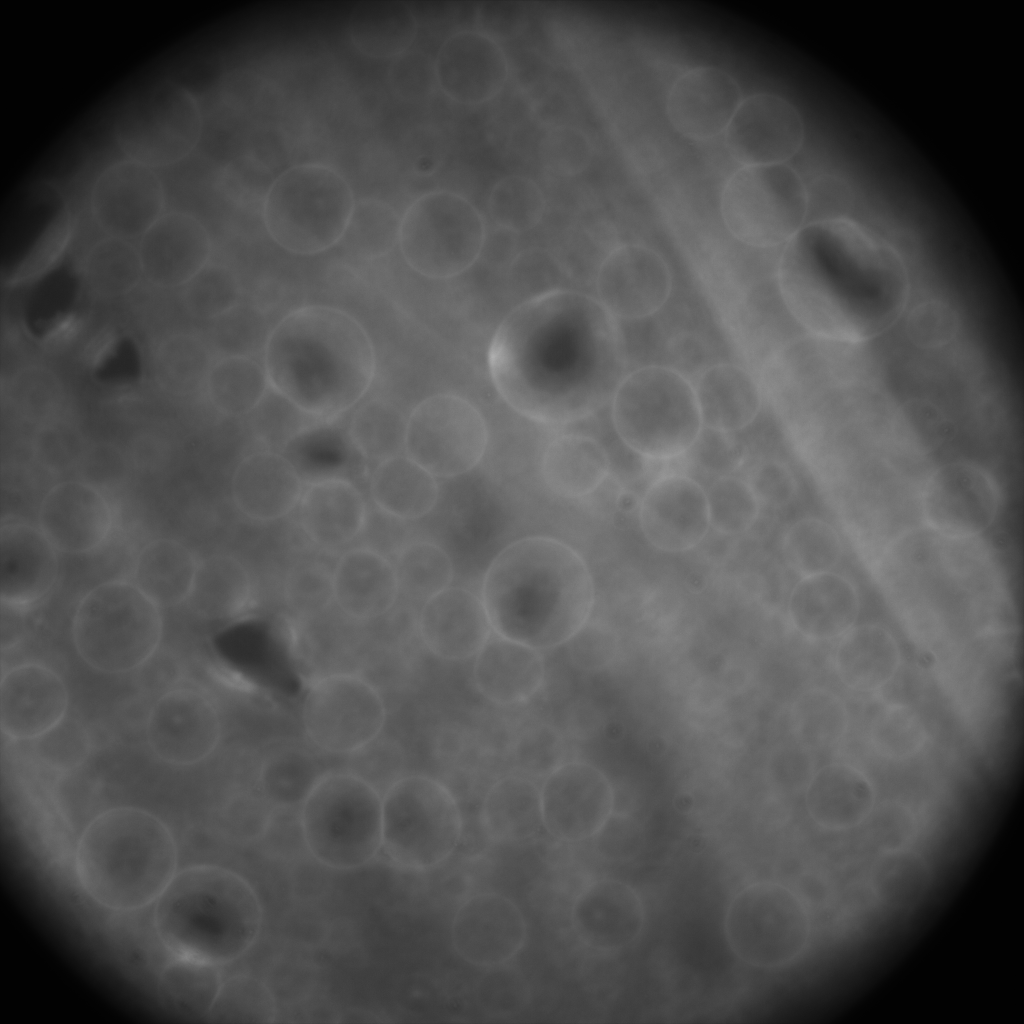

Supplement: S5 File — Zip file archive containing original photomicrographs of polymersomes formed following rehydration with sucrose and on gels prepared with water. (ZIP) [file pone.0158729.s005.zip › Sucrose Rehydration on Gel Prepared in Water/Image_4413_20150316_154429.tif]

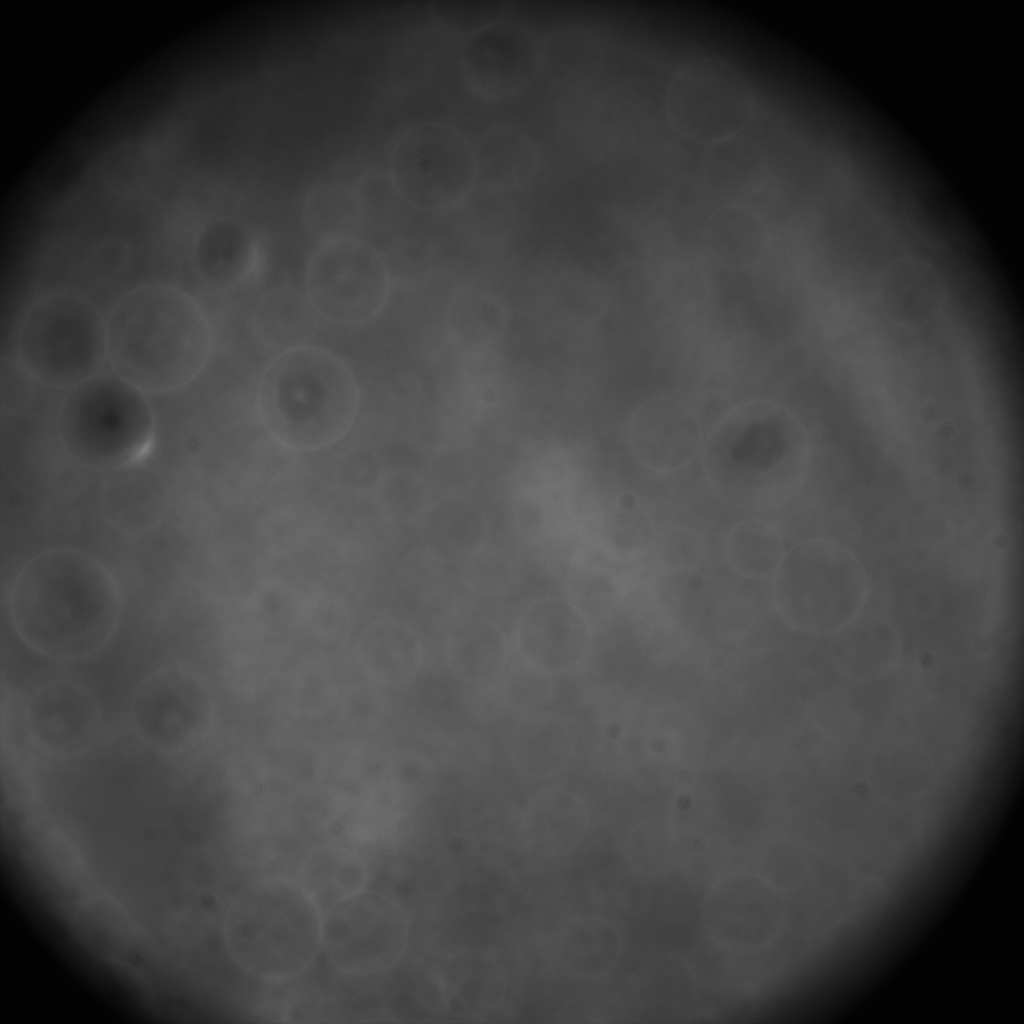

Supplement: S5 File — Zip file archive containing original photomicrographs of polymersomes formed following rehydration with sucrose and on gels prepared with water. (ZIP) [file pone.0158729.s005.zip › Sucrose Rehydration on Gel Prepared in Water/Image_4414_20150316_154444.tif]

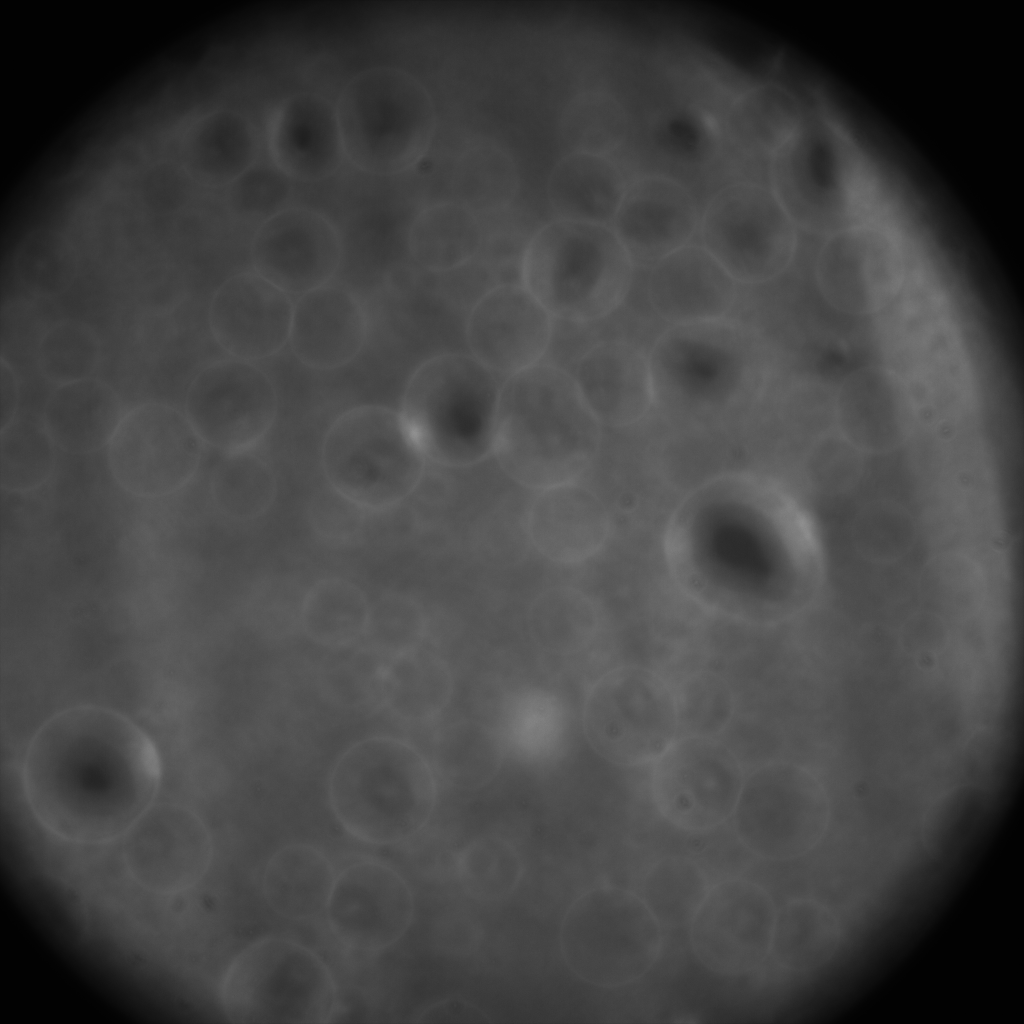

Supplement: S5 File — Zip file archive containing original photomicrographs of polymersomes formed following rehydration with sucrose and on gels prepared with water. (ZIP) [file pone.0158729.s005.zip › Sucrose Rehydration on Gel Prepared in Water/Image_4415_20150316_154455.tif]

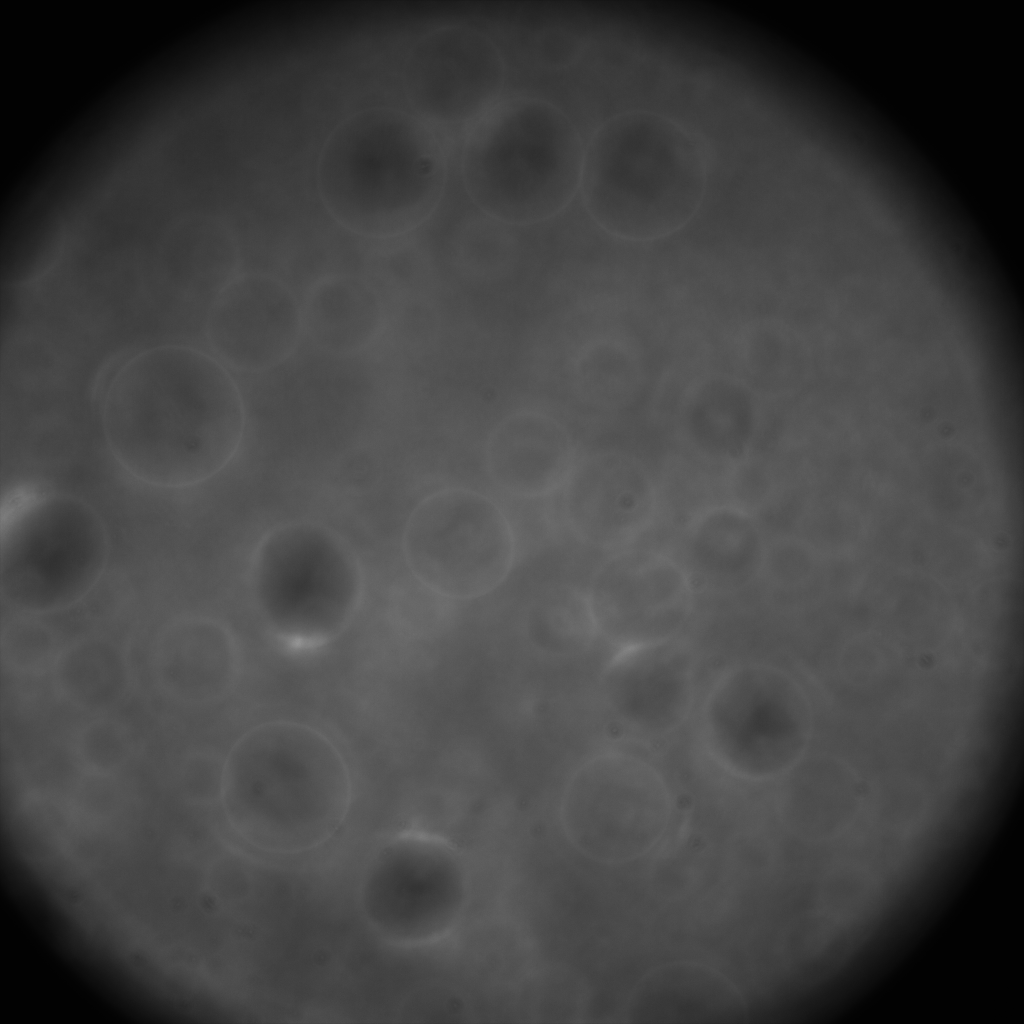

Supplement: S5 File — Zip file archive containing original photomicrographs of polymersomes formed following rehydration with sucrose and on gels prepared with water. (ZIP) [file pone.0158729.s005.zip › Sucrose Rehydration on Gel Prepared in Water/Image_4416_20150316_154517.tif]

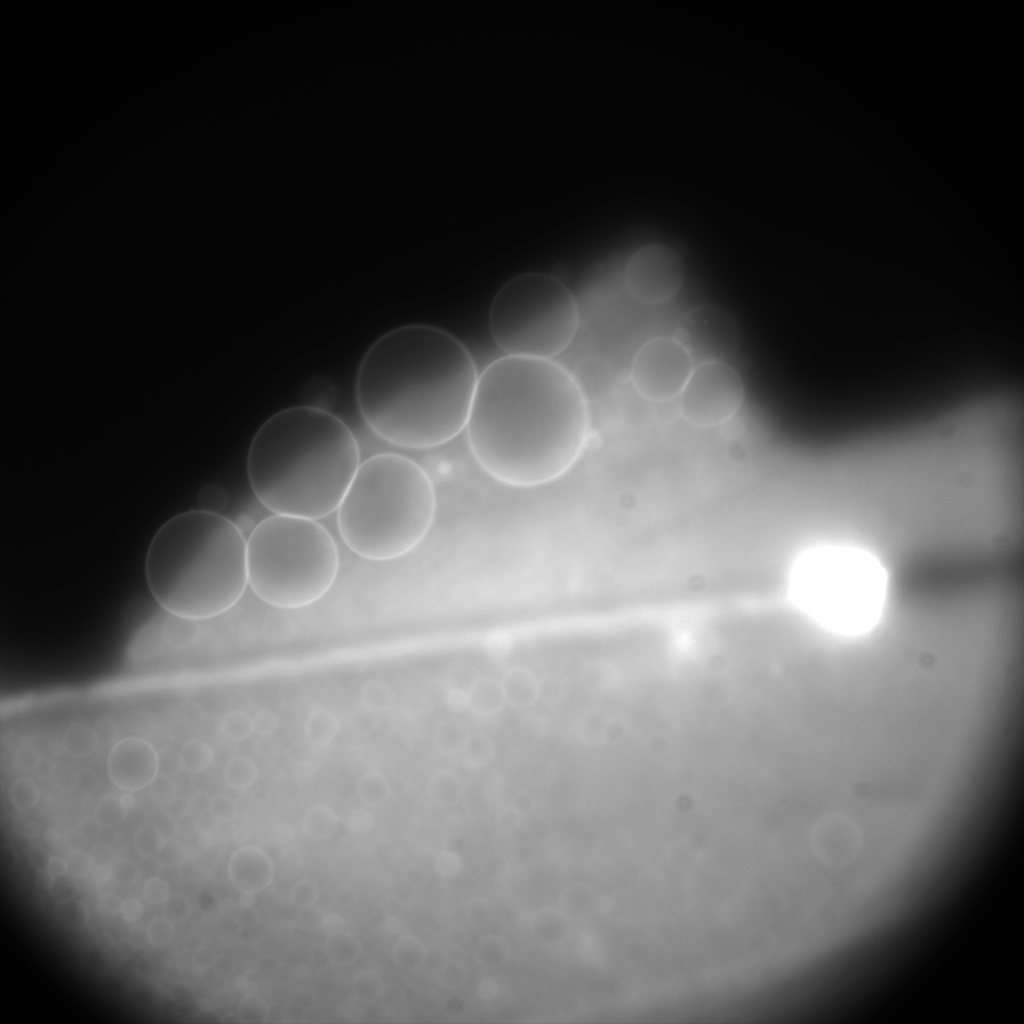

Supplement: S6 File — Zip file archive containing original photomicrographs of polymersomes formed following rehydration with sucrose and on gels prepared with water. (ZIP) [file pone.0158729.s006.zip › Water Rehydration on Gel Prepared in Sucrose/Image_4958_20150330_123232.tif]

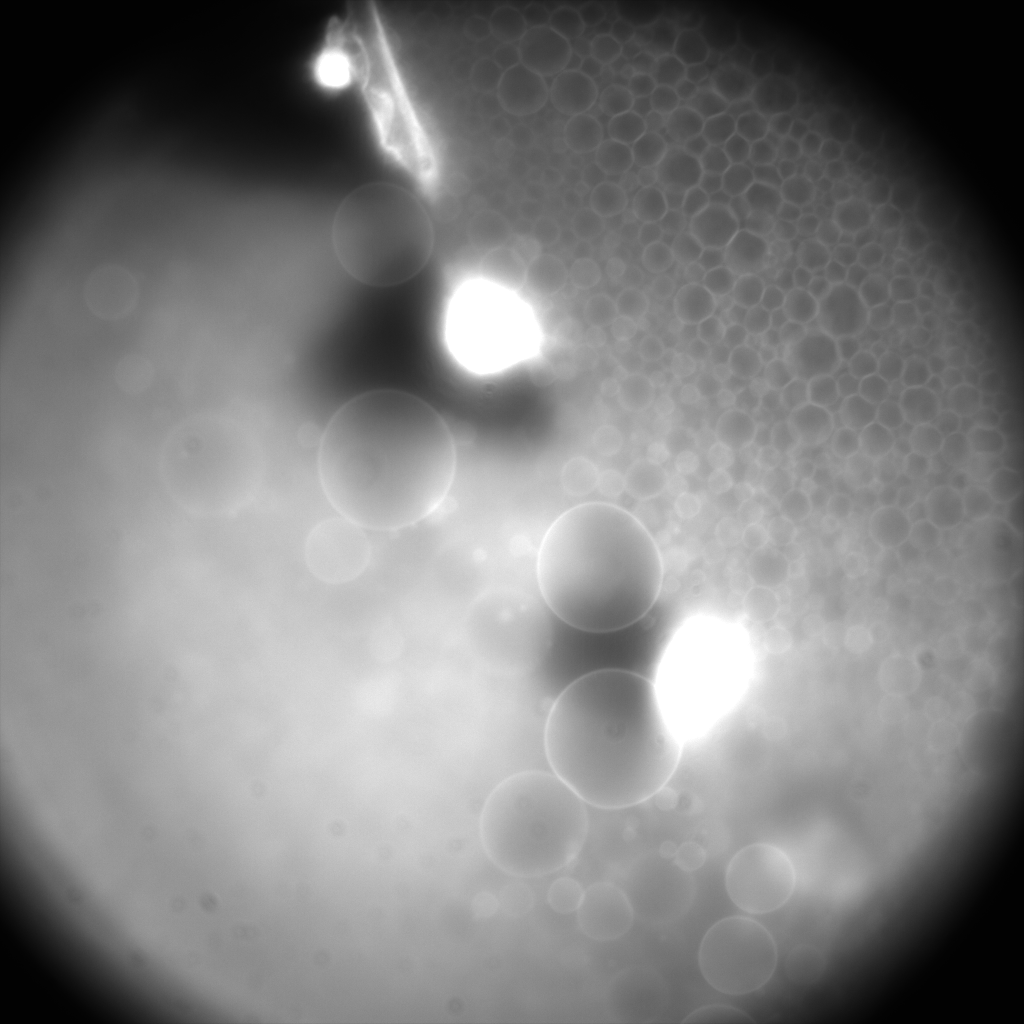

Supplement: S6 File — Zip file archive containing original photomicrographs of polymersomes formed following rehydration with sucrose and on gels prepared with water. (ZIP) [file pone.0158729.s006.zip › Water Rehydration on Gel Prepared in Sucrose/Image_4959_20150330_123245.tif]

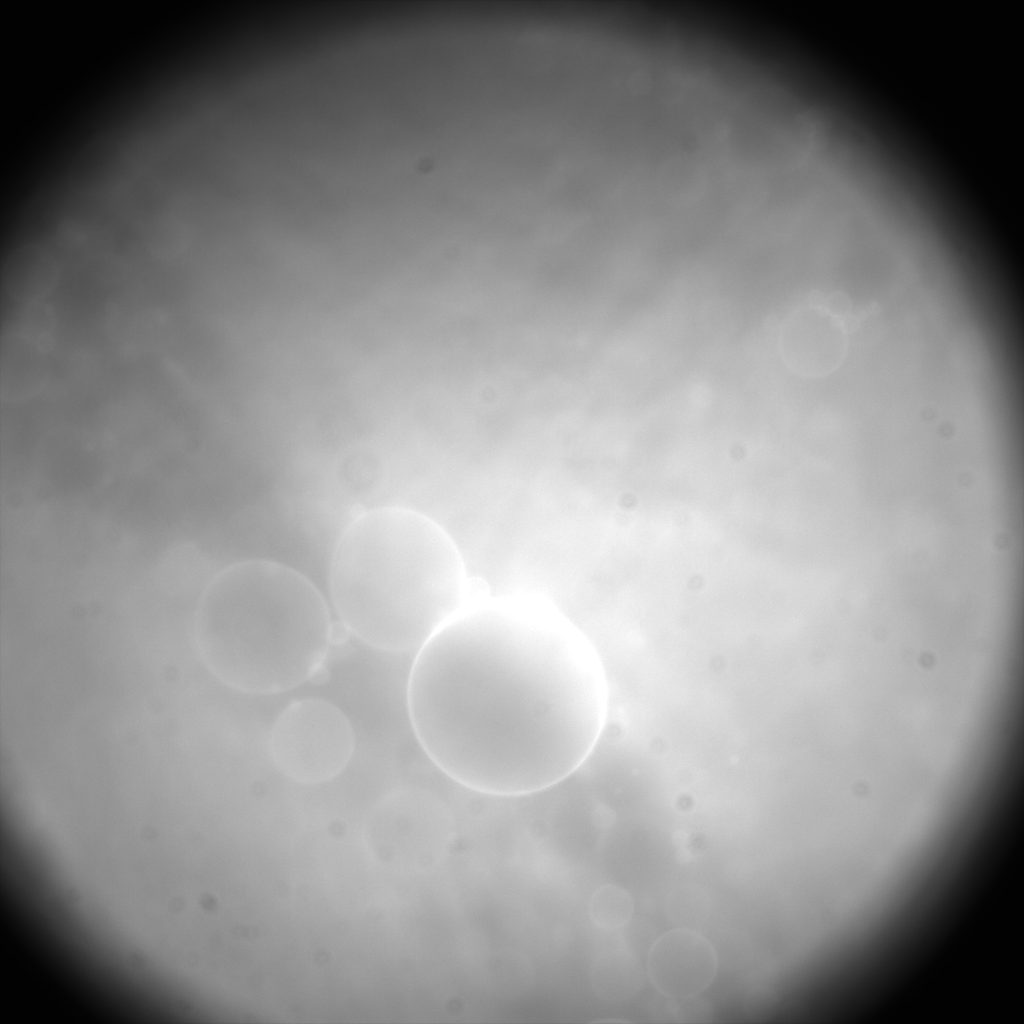

Supplement: S6 File — Zip file archive containing original photomicrographs of polymersomes formed following rehydration with sucrose and on gels prepared with water. (ZIP) [file pone.0158729.s006.zip › Water Rehydration on Gel Prepared in Sucrose/Image_4960_20150330_123255.tif]

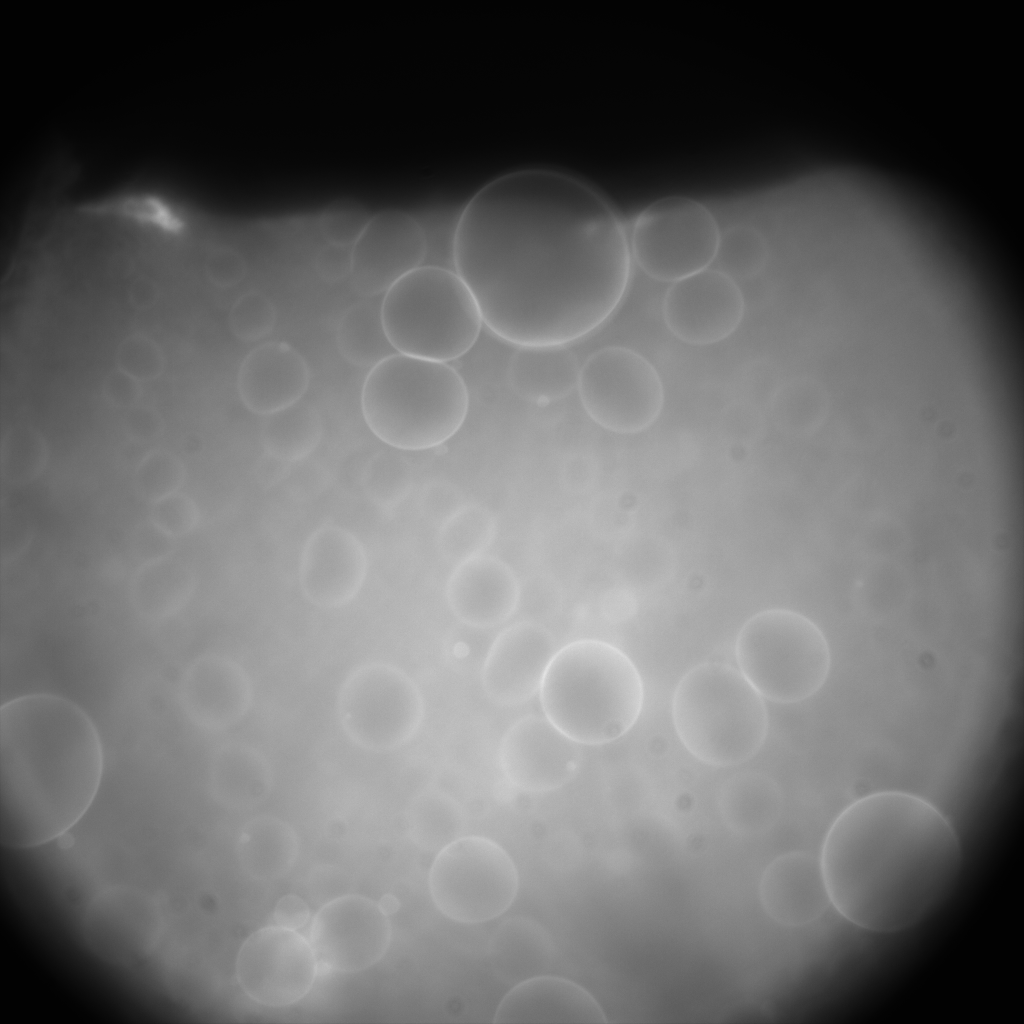

Supplement: S6 File — Zip file archive containing original photomicrographs of polymersomes formed following rehydration with sucrose and on gels prepared with water. (ZIP) [file pone.0158729.s006.zip › Water Rehydration on Gel Prepared in Sucrose/Image_4961_20150330_123308.tif]

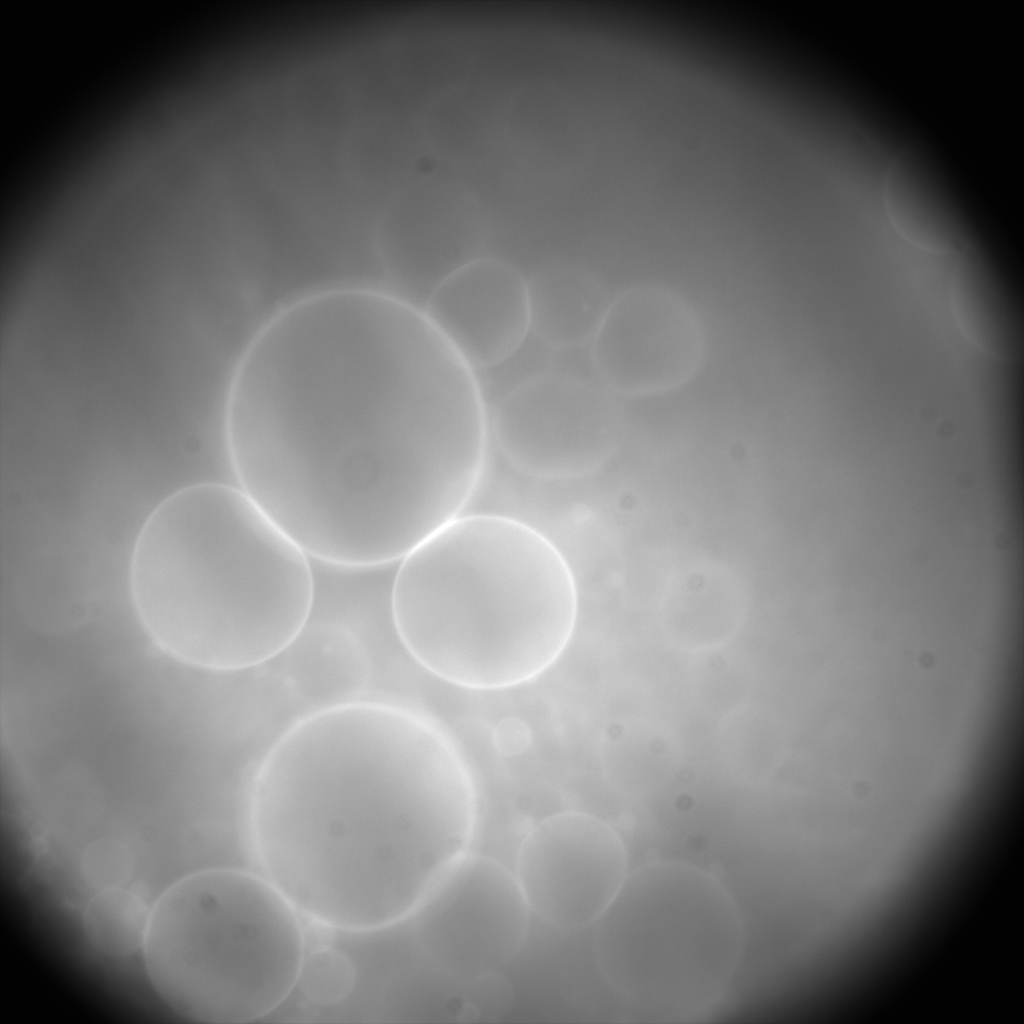

Supplement: S6 File — Zip file archive containing original photomicrographs of polymersomes formed following rehydration with sucrose and on gels prepared with water. (ZIP) [file pone.0158729.s006.zip › Water Rehydration on Gel Prepared in Sucrose/Image_4962_20150330_123322.tif]

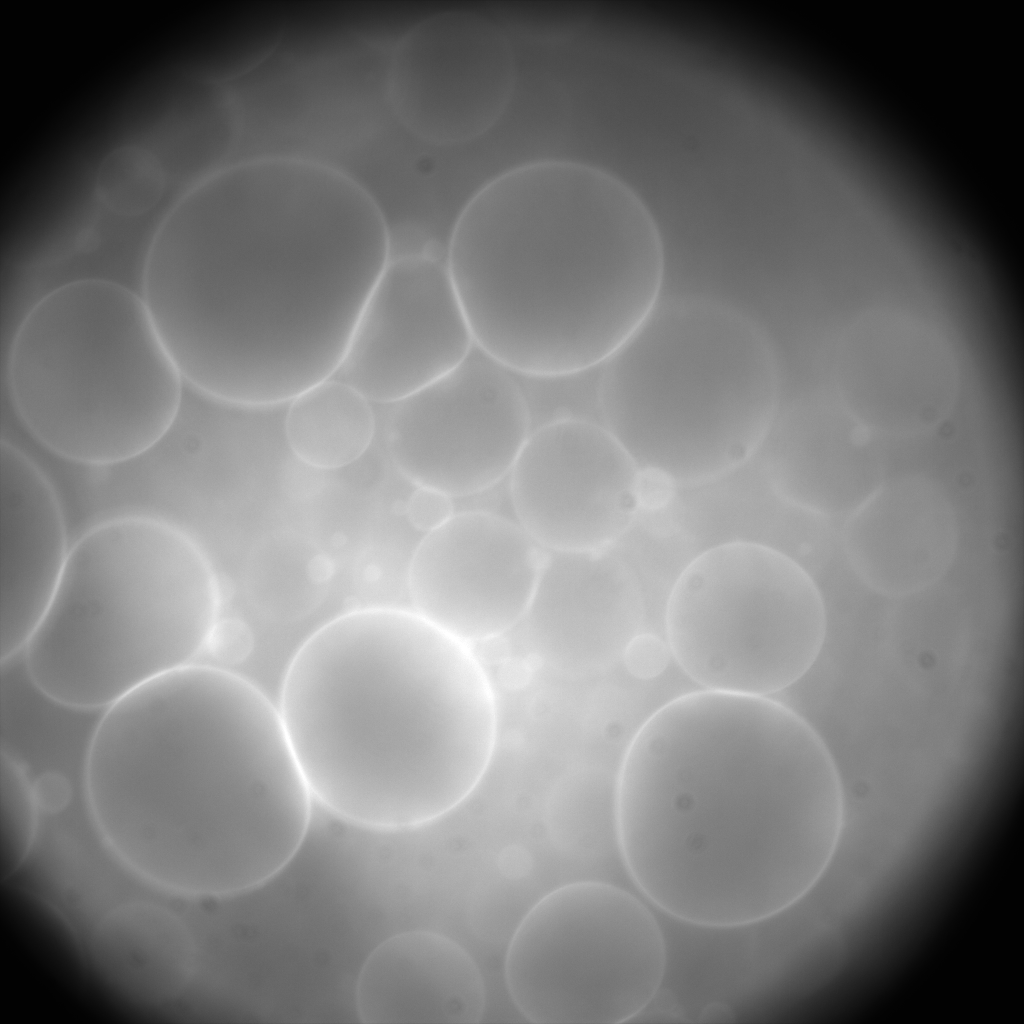

Supplement: S6 File — Zip file archive containing original photomicrographs of polymersomes formed following rehydration with sucrose and on gels prepared with water. (ZIP) [file pone.0158729.s006.zip › Water Rehydration on Gel Prepared in Sucrose/Image_4963_20150330_123335.tif]

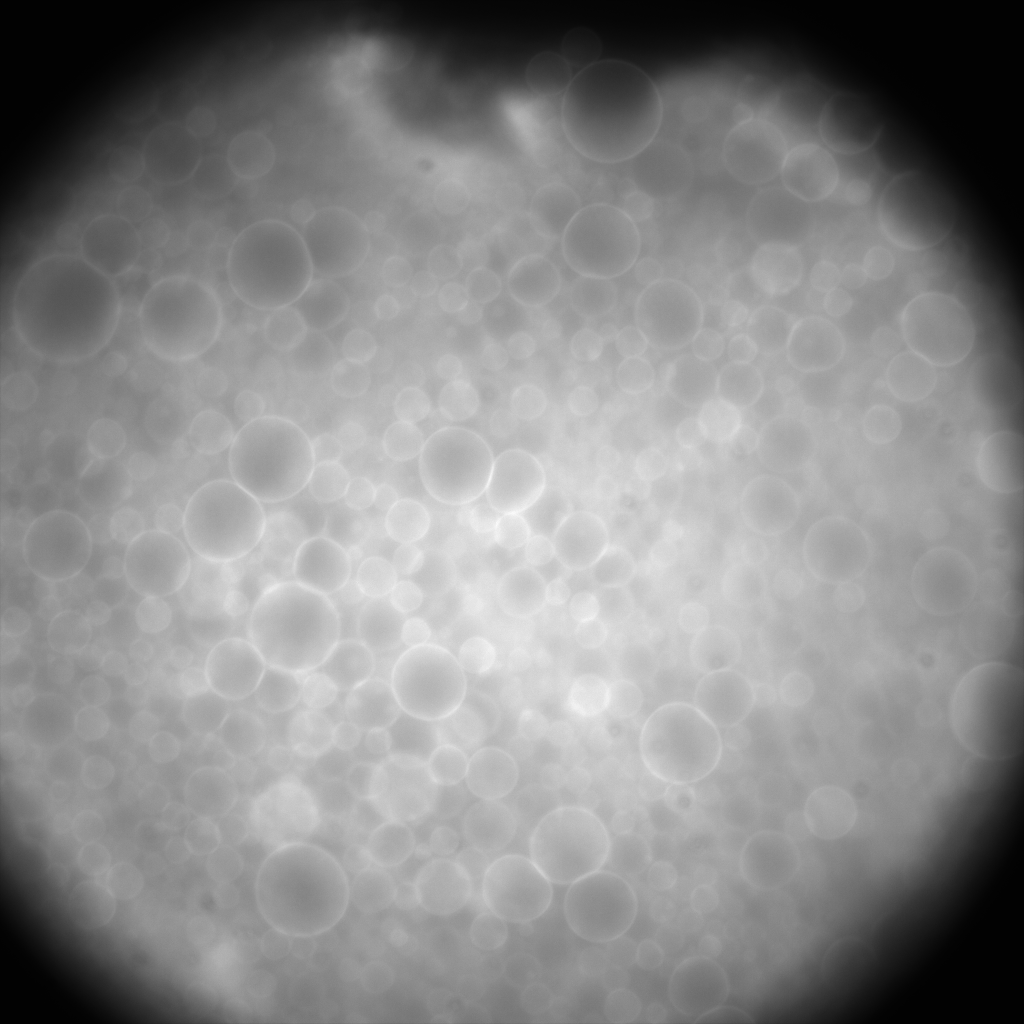

Supplement: S6 File — Zip file archive containing original photomicrographs of polymersomes formed following rehydration with sucrose and on gels prepared with water. (ZIP) [file pone.0158729.s006.zip › Water Rehydration on Gel Prepared in Sucrose/Image_4995_20150330_130013.tif]

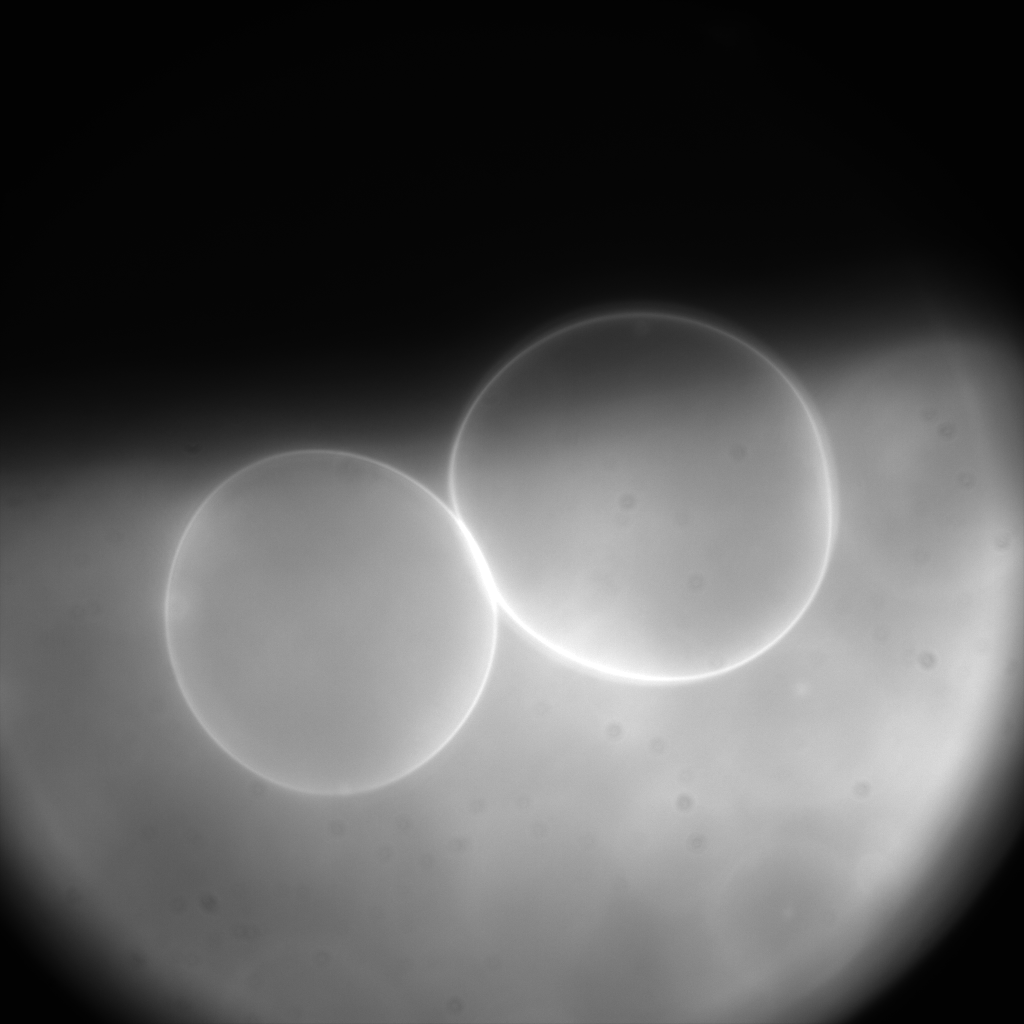

Supplement: S6 File — Zip file archive containing original photomicrographs of polymersomes formed following rehydration with sucrose and on gels prepared with water. (ZIP) [file pone.0158729.s006.zip › Water Rehydration on Gel Prepared in Sucrose/Image_4996_20150330_130035.tif]

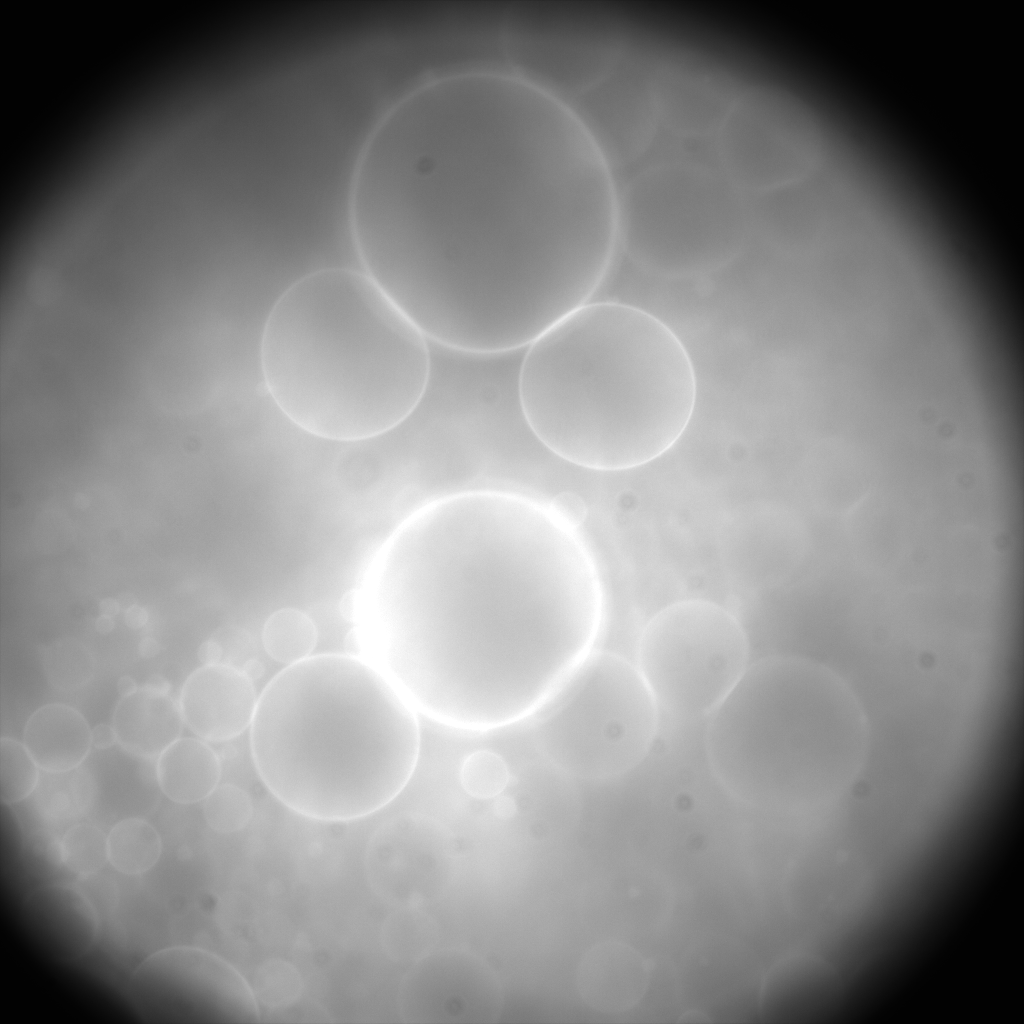

Supplement: S6 File — Zip file archive containing original photomicrographs of polymersomes formed following rehydration with sucrose and on gels prepared with water. (ZIP) [file pone.0158729.s006.zip › Water Rehydration on Gel Prepared in Sucrose/Image_4997_20150330_130326.tif]

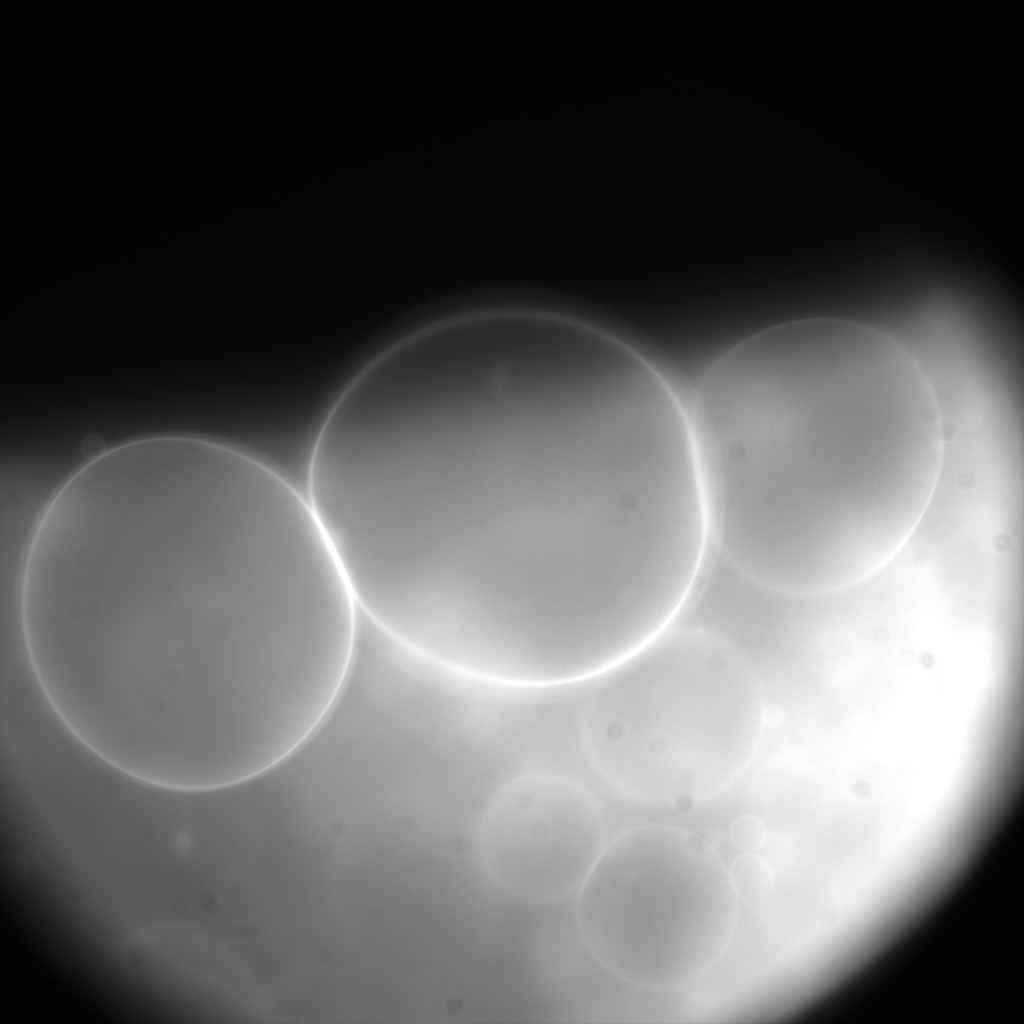

Supplement: S6 File — Zip file archive containing original photomicrographs of polymersomes formed following rehydration with sucrose and on gels prepared with water. (ZIP) [file pone.0158729.s006.zip › Water Rehydration on Gel Prepared in Sucrose/Image_4998_20150330_130830.tif]

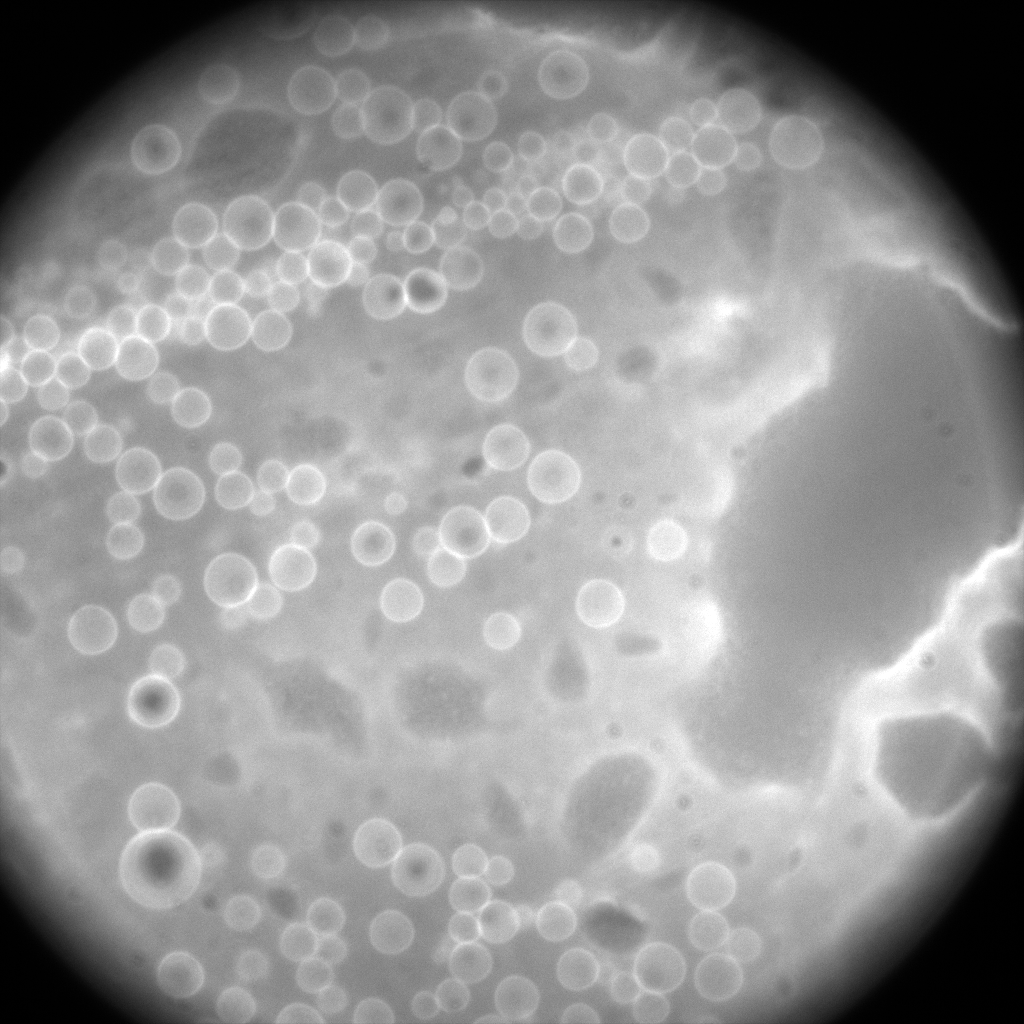

Supplement: S7 File — Zip archive containing original photomicrographs of polymersomes formed following rehydration with water and on gels prepared with water. (ZIP) [file pone.0158729.s007.zip › Water Rehydration on Gel Prepared in Water/Image_4318_20150316_134659.tif]

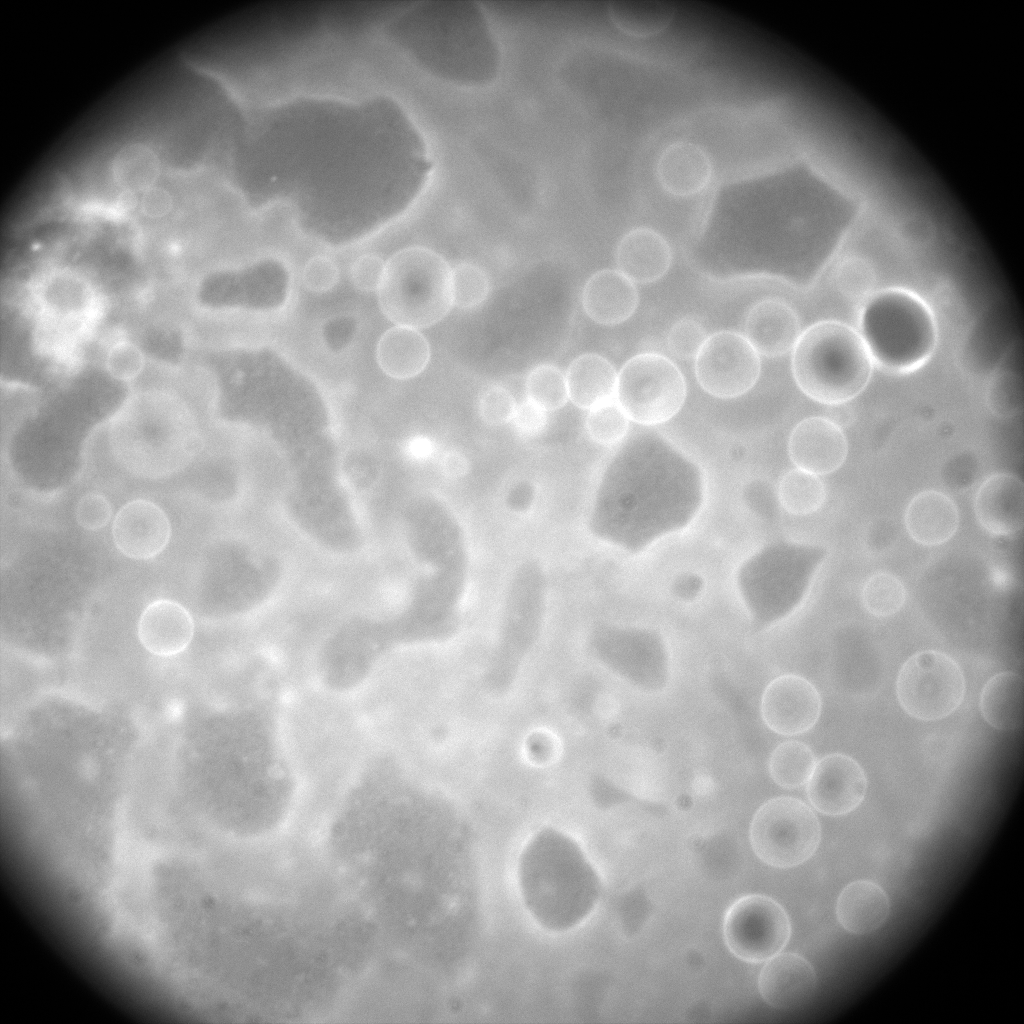

Supplement: S7 File — Zip archive containing original photomicrographs of polymersomes formed following rehydration with water and on gels prepared with water. (ZIP) [file pone.0158729.s007.zip › Water Rehydration on Gel Prepared in Water/Image_4319_20150316_134714.tif]

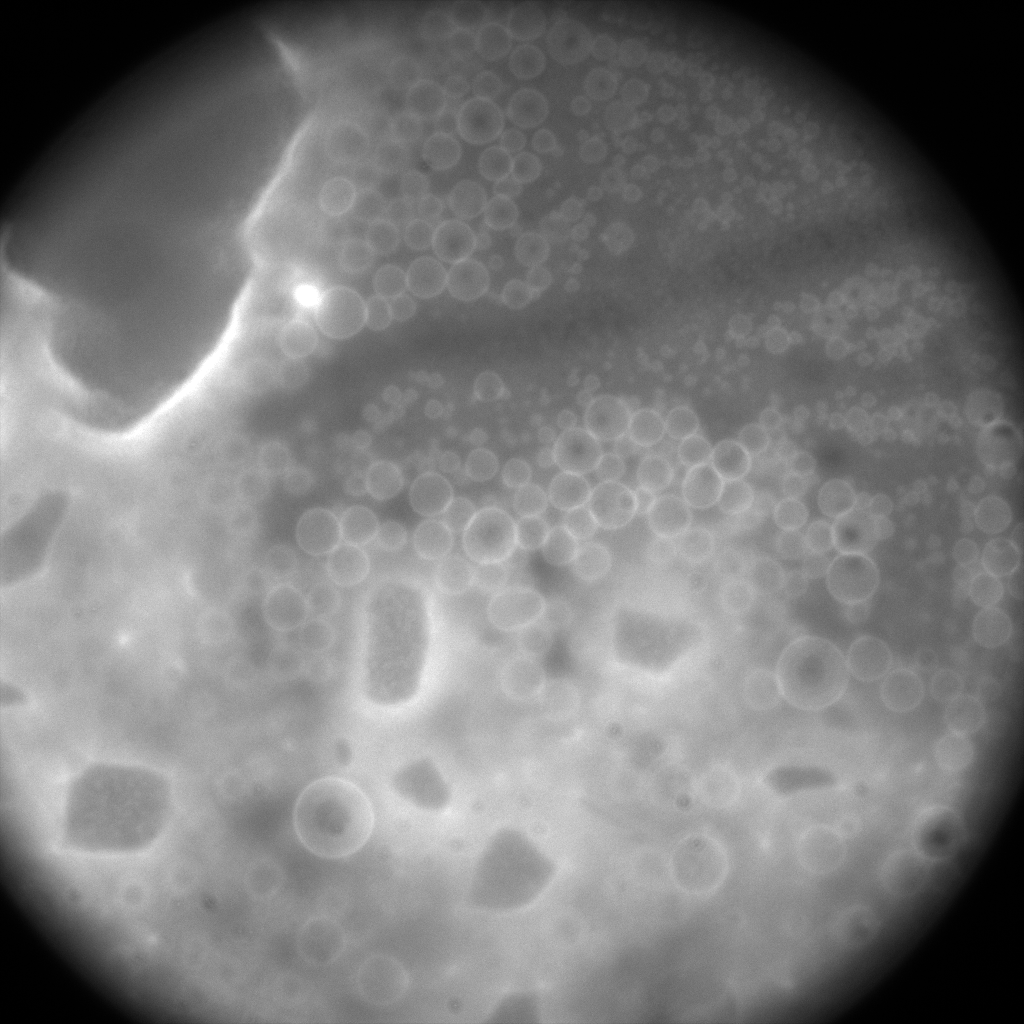

Supplement: S7 File — Zip archive containing original photomicrographs of polymersomes formed following rehydration with water and on gels prepared with water. (ZIP) [file pone.0158729.s007.zip › Water Rehydration on Gel Prepared in Water/Image_4320_20150316_134736.tif]

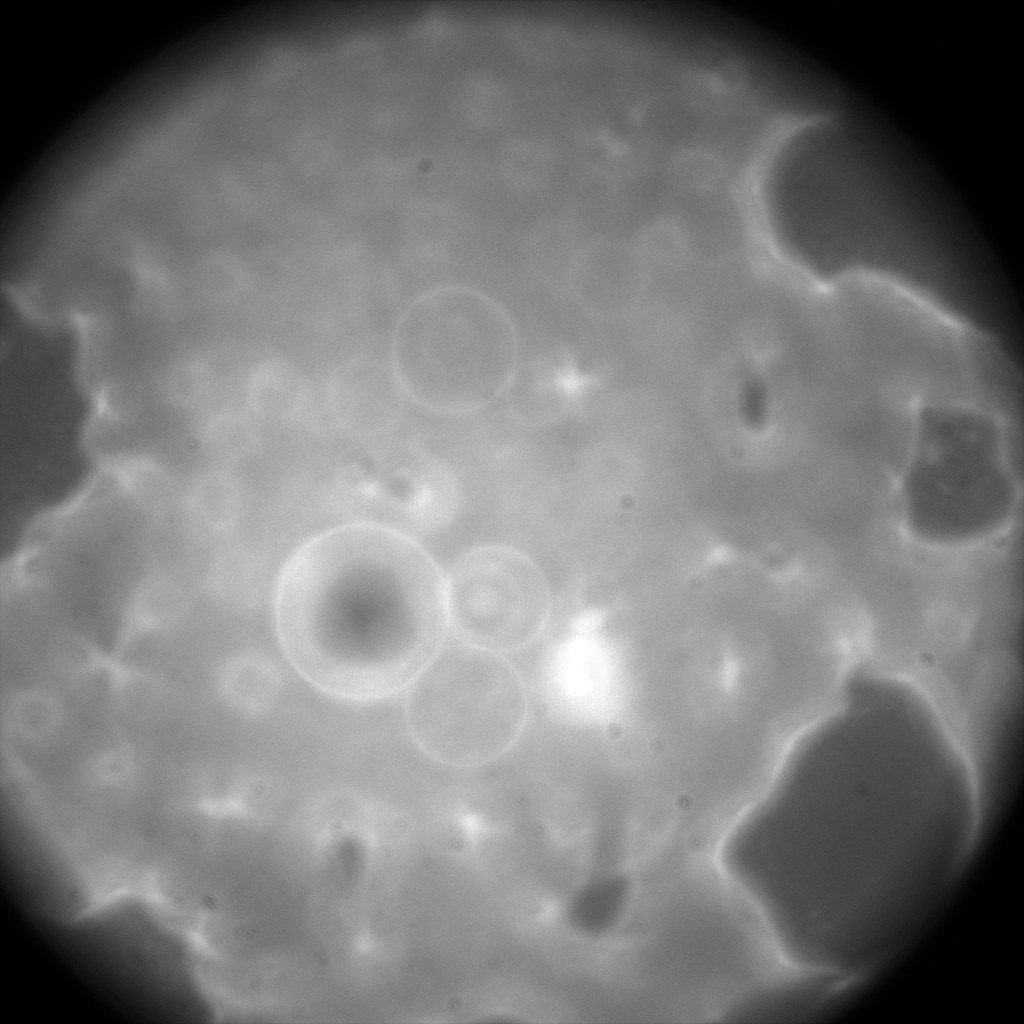

Supplement: S7 File — Zip archive containing original photomicrographs of polymersomes formed following rehydration with water and on gels prepared with water. (ZIP) [file pone.0158729.s007.zip › Water Rehydration on Gel Prepared in Water/Image_4321_20150316_134746.tif]

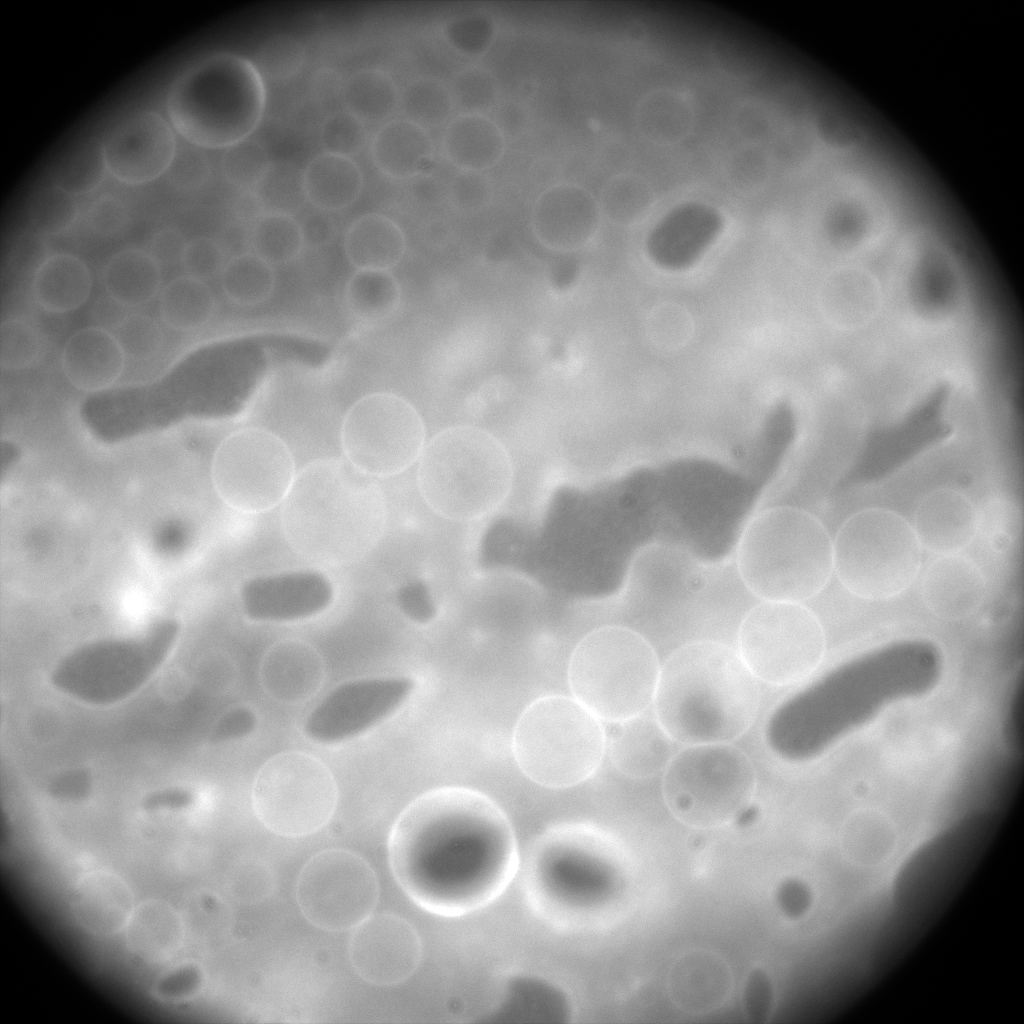

Supplement: S7 File — Zip archive containing original photomicrographs of polymersomes formed following rehydration with water and on gels prepared with water. (ZIP) [file pone.0158729.s007.zip › Water Rehydration on Gel Prepared in Water/Image_4322_20150316_134808.tif]

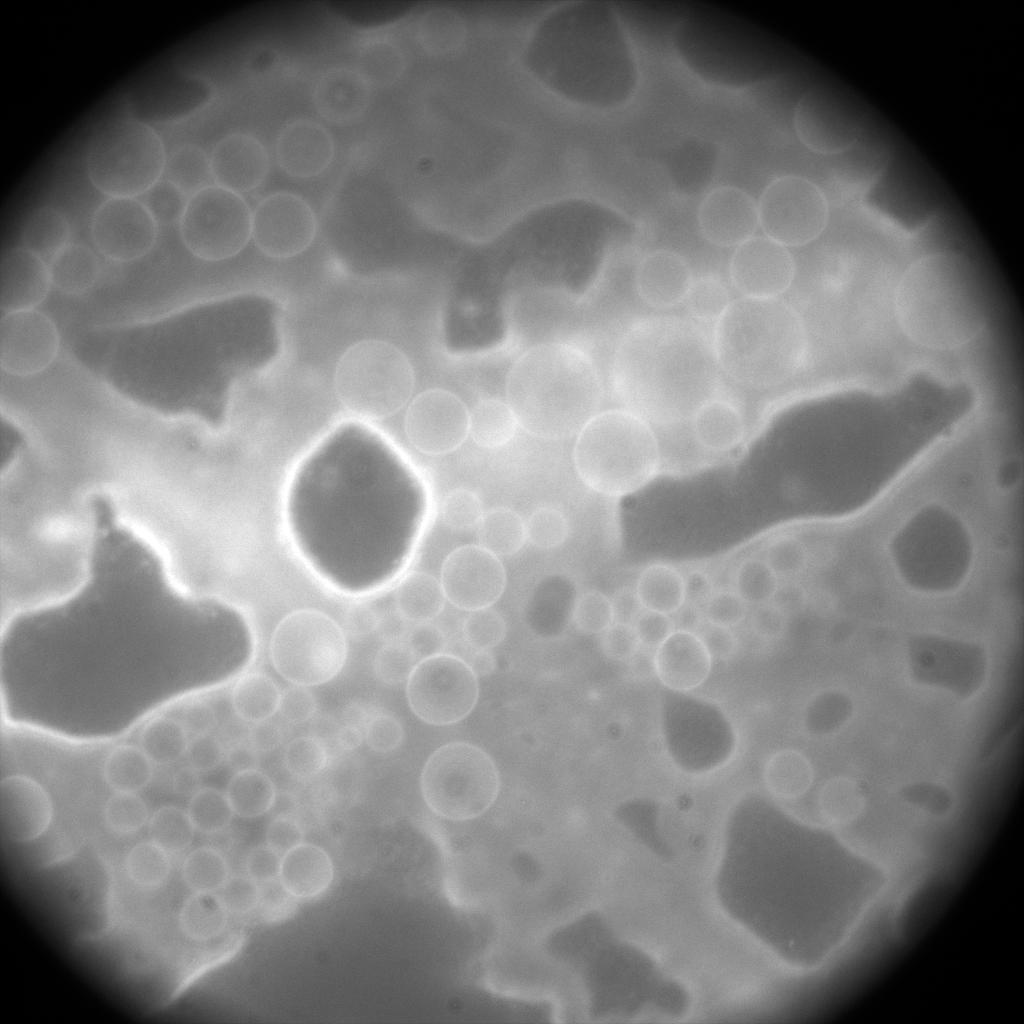

Supplement: S7 File — Zip archive containing original photomicrographs of polymersomes formed following rehydration with water and on gels prepared with water. (ZIP) [file pone.0158729.s007.zip › Water Rehydration on Gel Prepared in Water/Image_4328_20150316_134917.tif]

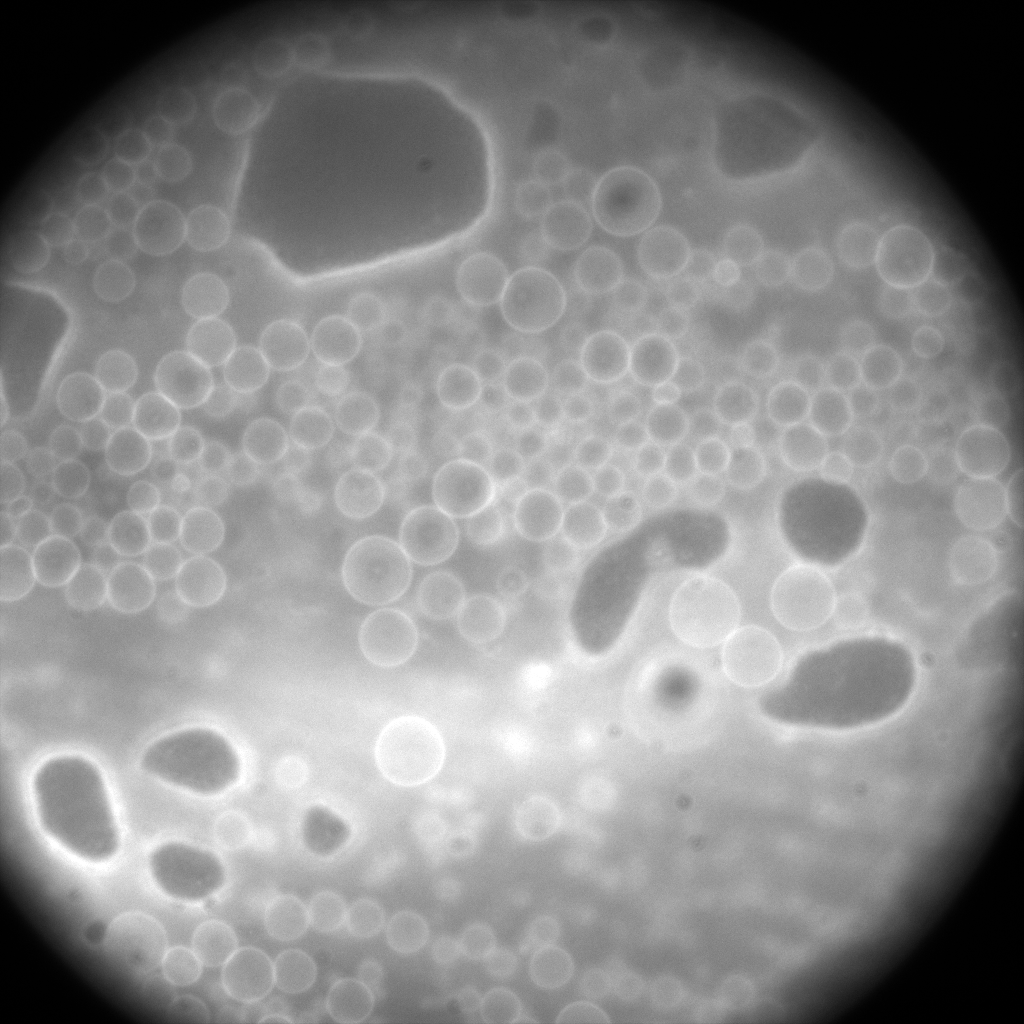

Supplement: S7 File — Zip archive containing original photomicrographs of polymersomes formed following rehydration with water and on gels prepared with water. (ZIP) [file pone.0158729.s007.zip › Water Rehydration on Gel Prepared in Water/Image_4329_20150316_134942.tif]

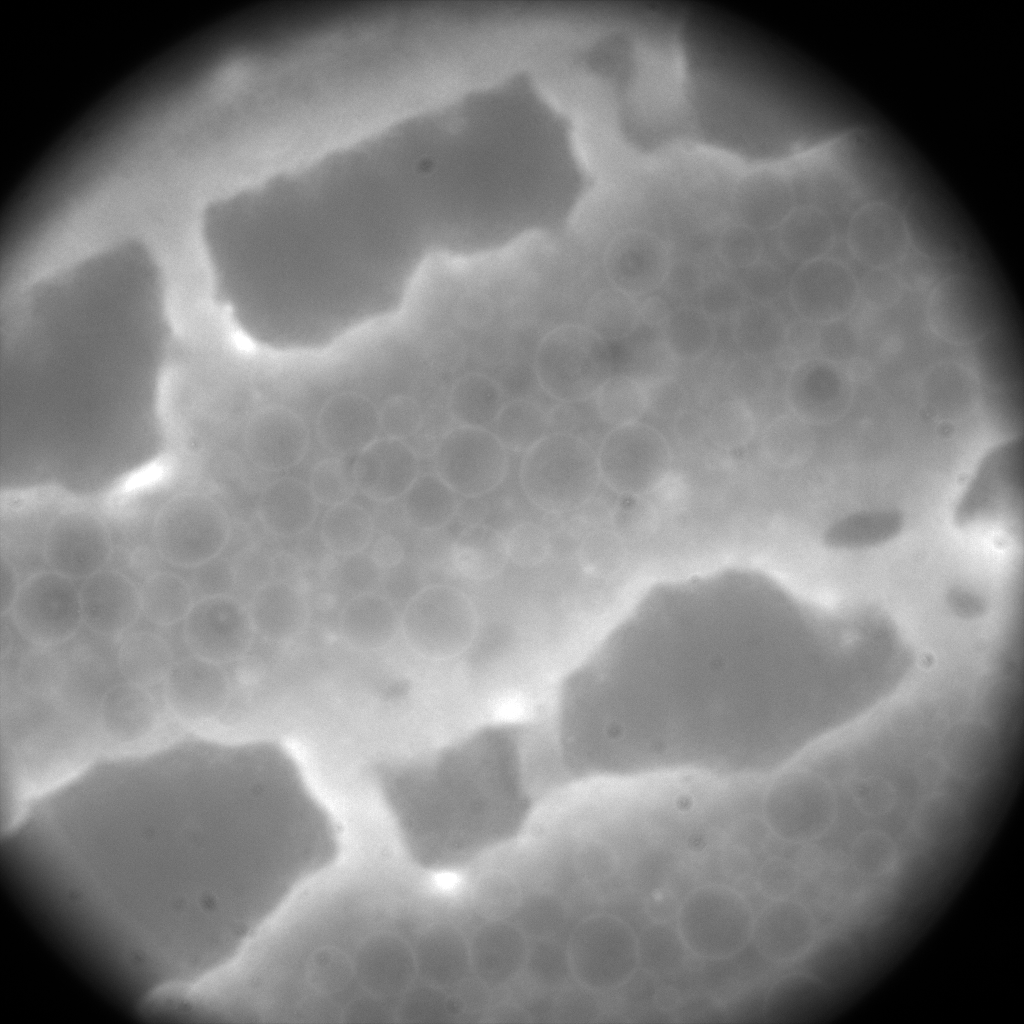

Supplement: S7 File — Zip archive containing original photomicrographs of polymersomes formed following rehydration with water and on gels prepared with water. (ZIP) [file pone.0158729.s007.zip › Water Rehydration on Gel Prepared in Water/Image_4330_20150316_135039.tif]

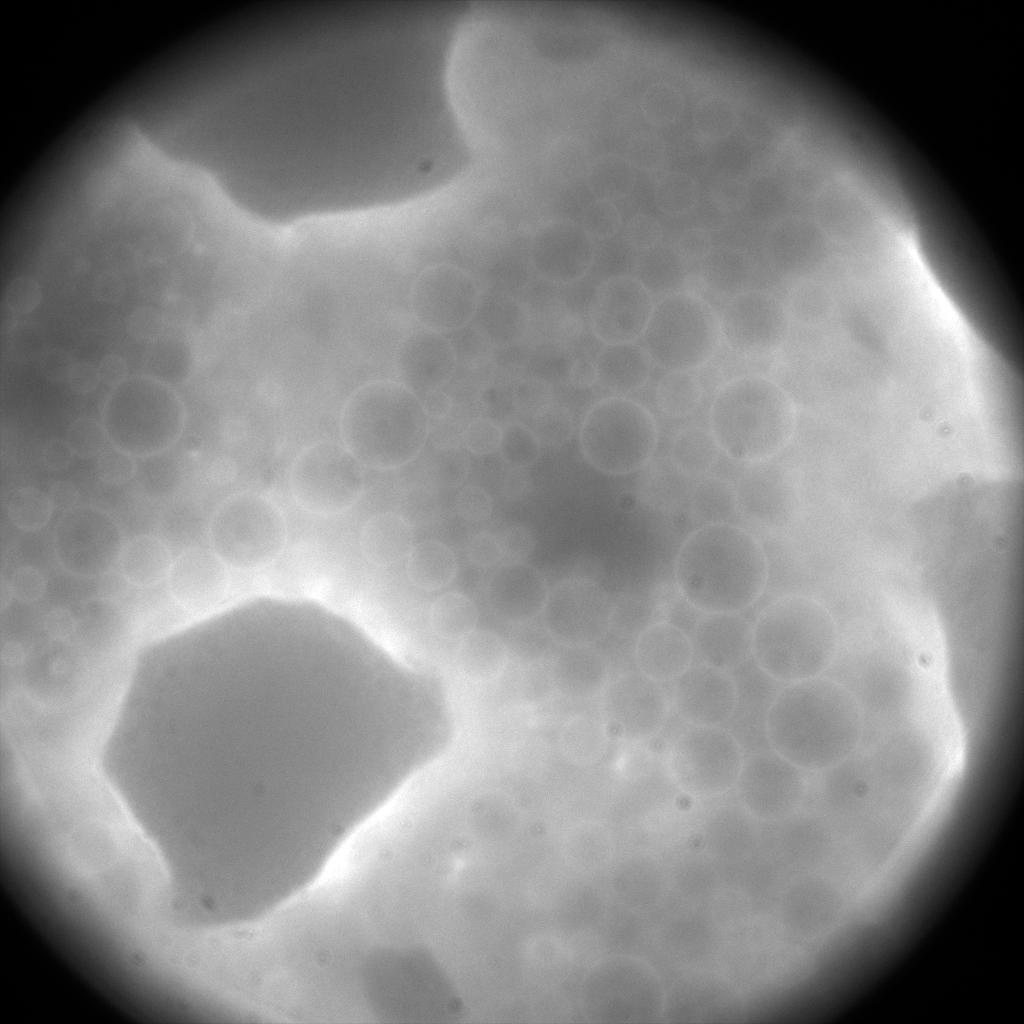

Supplement: S7 File — Zip archive containing original photomicrographs of polymersomes formed following rehydration with water and on gels prepared with water. (ZIP) [file pone.0158729.s007.zip › Water Rehydration on Gel Prepared in Water/Image_4331_20150316_135101.tif]

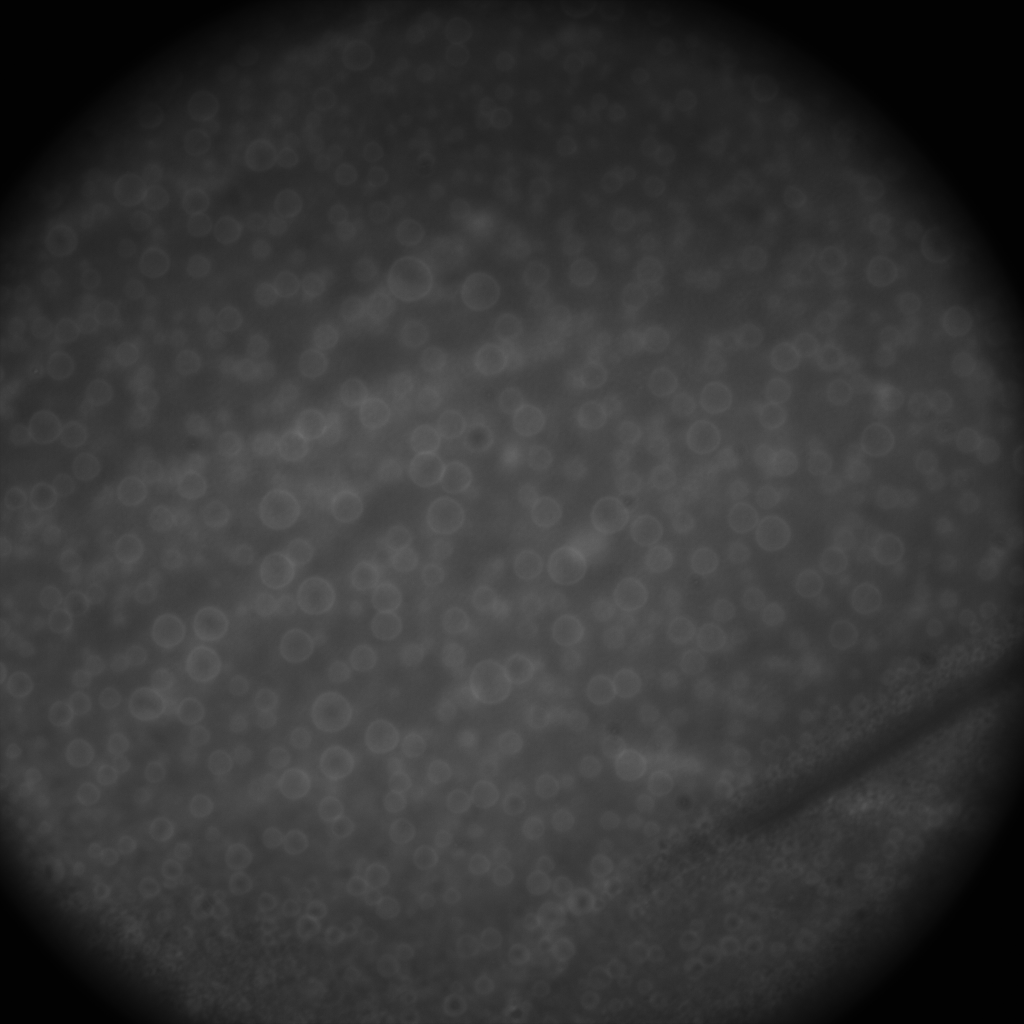

Supplement: S7 File — Zip archive containing original photomicrographs of polymersomes formed following rehydration with water and on gels prepared with water. (ZIP) [file pone.0158729.s007.zip › Water Rehydration on Gel Prepared in Water/Image_4402_20150316_153957.tif]

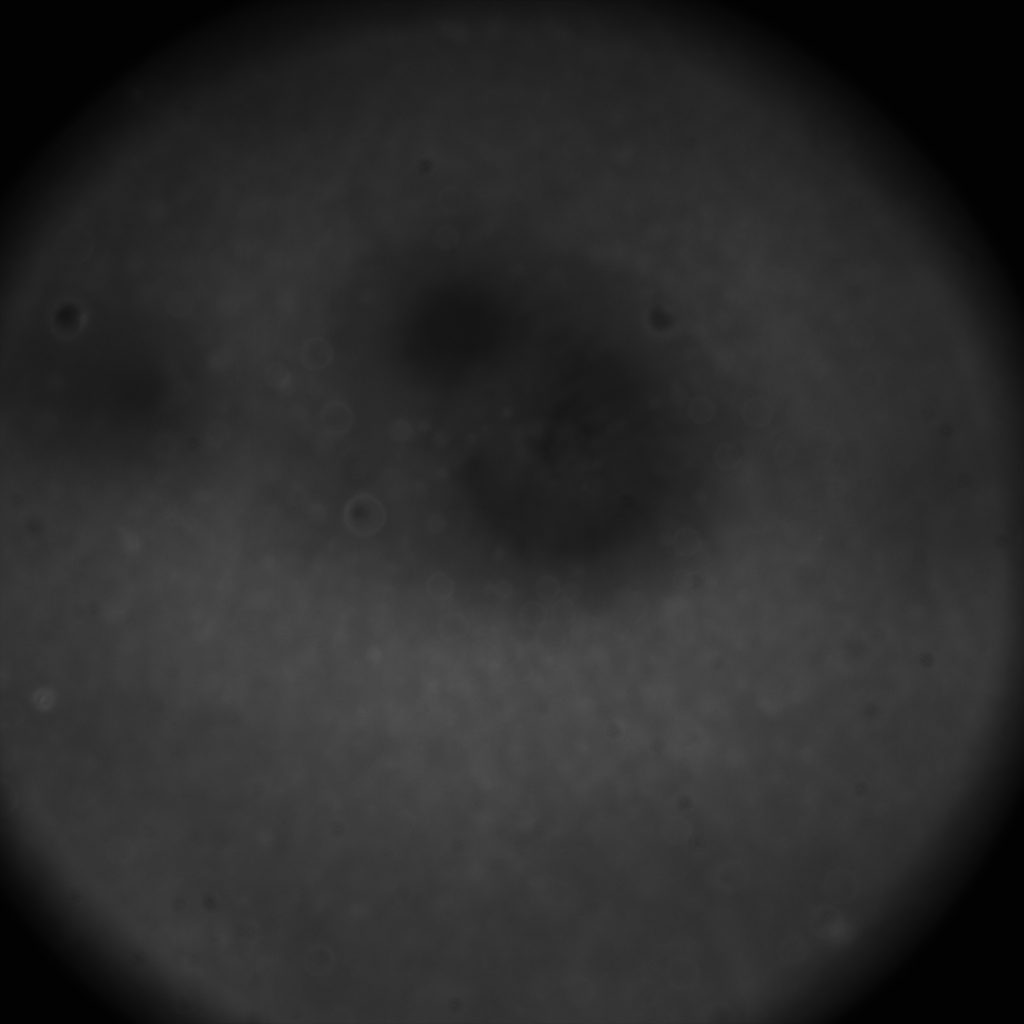

Supplement: S8 File — Zip file archive containing original photomicrographs of polymersomes formed following rehydration at 24°C. (ZIP) [file pone.0158729.s008.zip › 24C/Image_4307_20150316_133645.tif]

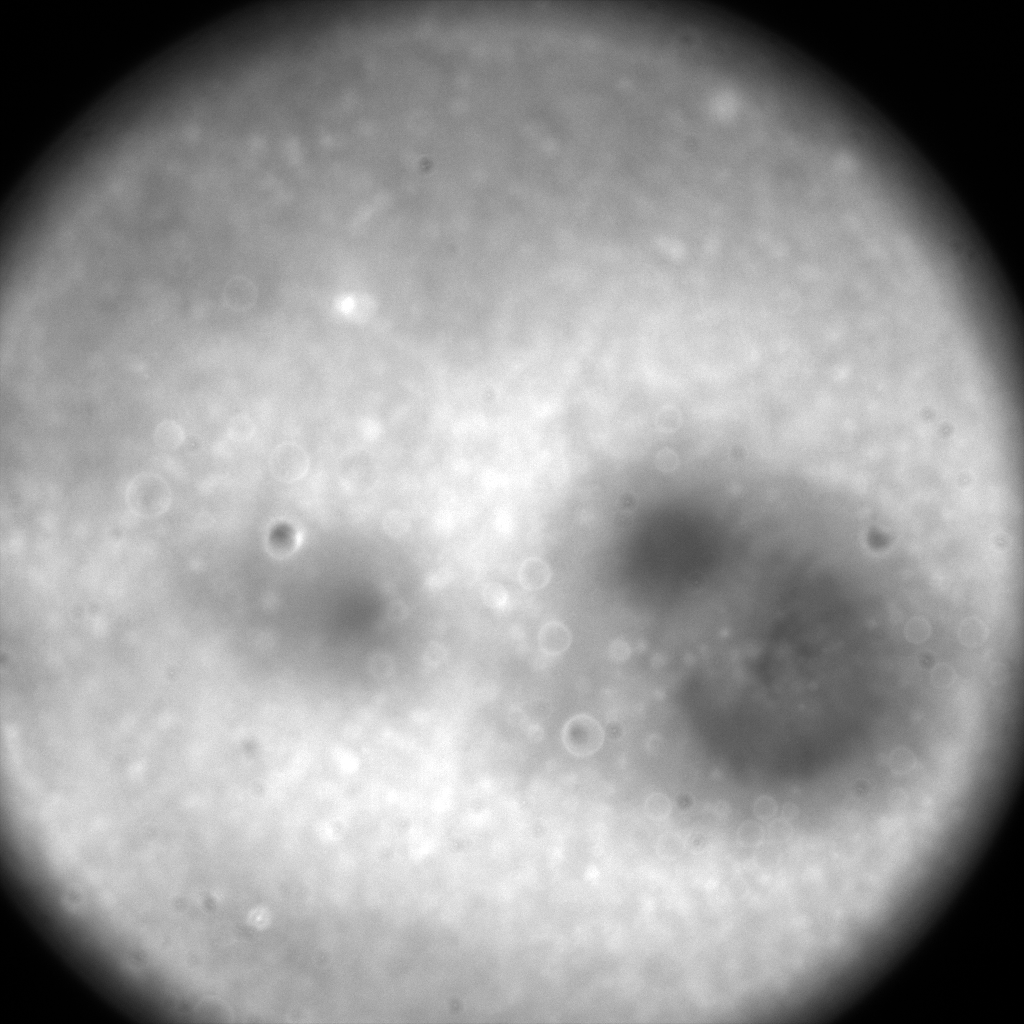

Supplement: S8 File — Zip file archive containing original photomicrographs of polymersomes formed following rehydration at 24°C. (ZIP) [file pone.0158729.s008.zip › 24C/Image_4309_20150316_133731.tif]

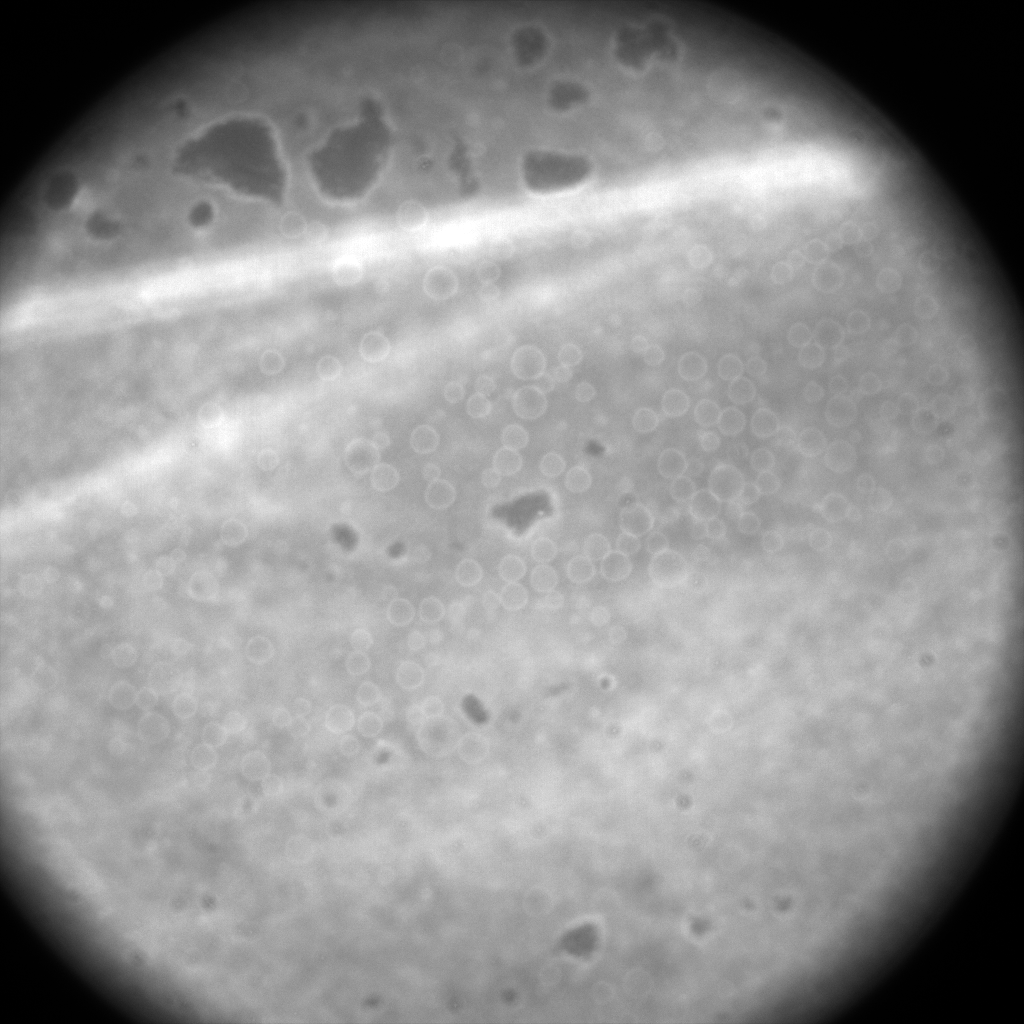

Supplement: S8 File — Zip file archive containing original photomicrographs of polymersomes formed following rehydration at 24°C. (ZIP) [file pone.0158729.s008.zip › 24C/Image_4310_20150316_133829.tif]

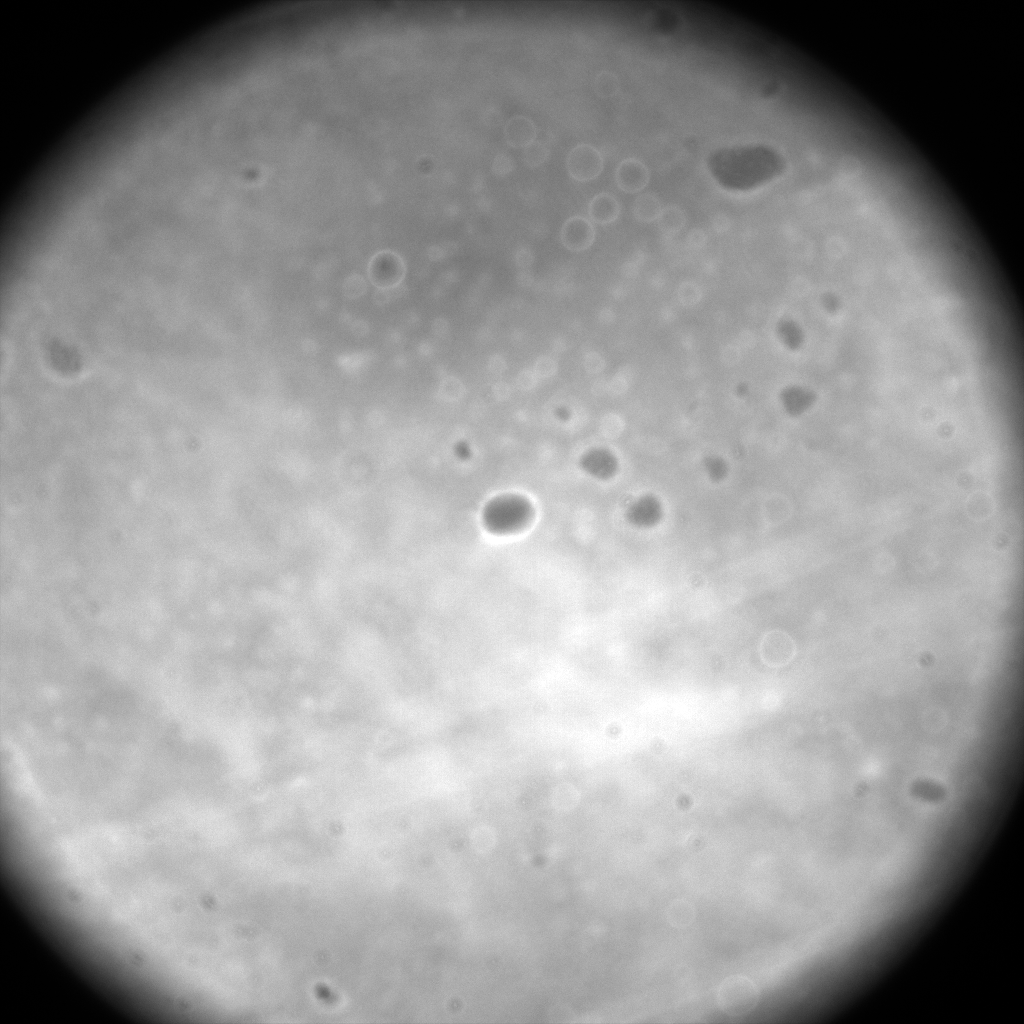

Supplement: S8 File — Zip file archive containing original photomicrographs of polymersomes formed following rehydration at 24°C. (ZIP) [file pone.0158729.s008.zip › 24C/Image_4311_20150316_133840.tif]

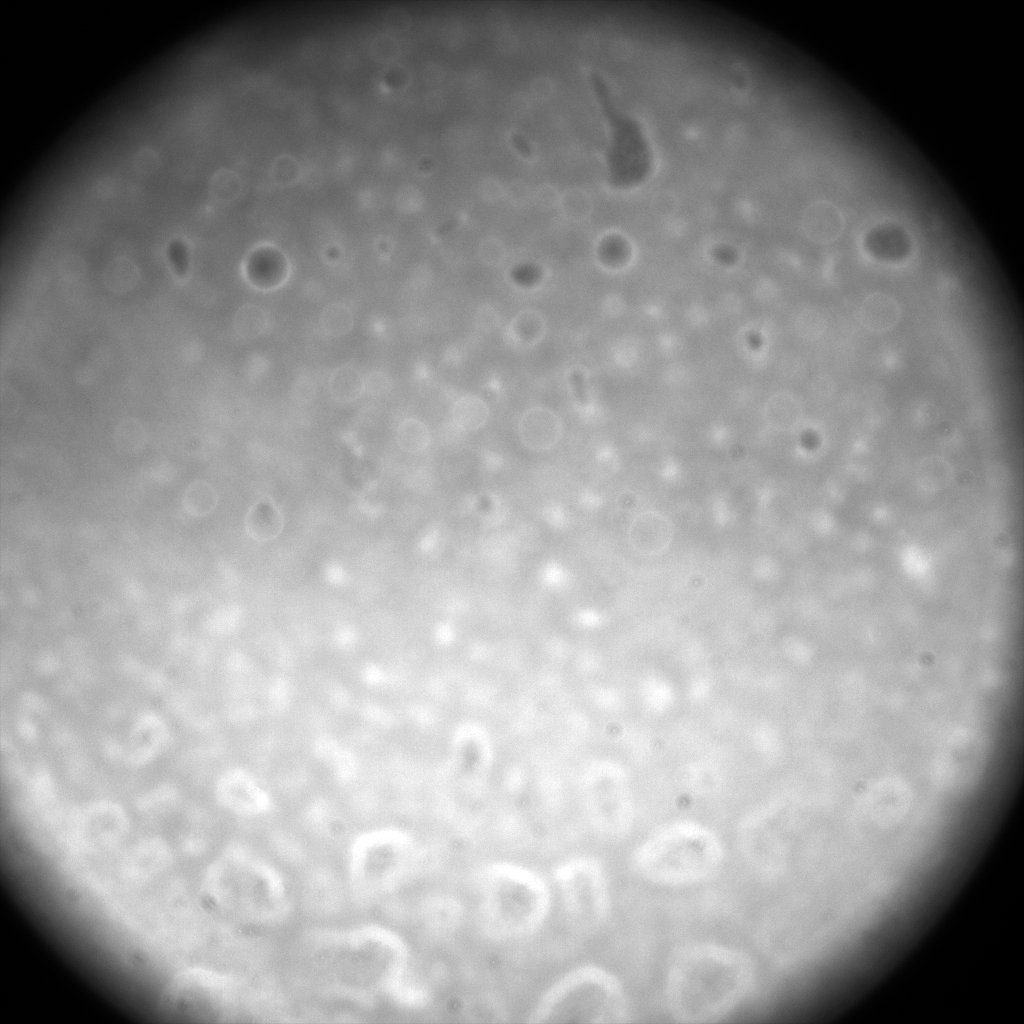

Supplement: S8 File — Zip file archive containing original photomicrographs of polymersomes formed following rehydration at 24°C. (ZIP) [file pone.0158729.s008.zip › 24C/Image_4312_20150316_133857.tif]

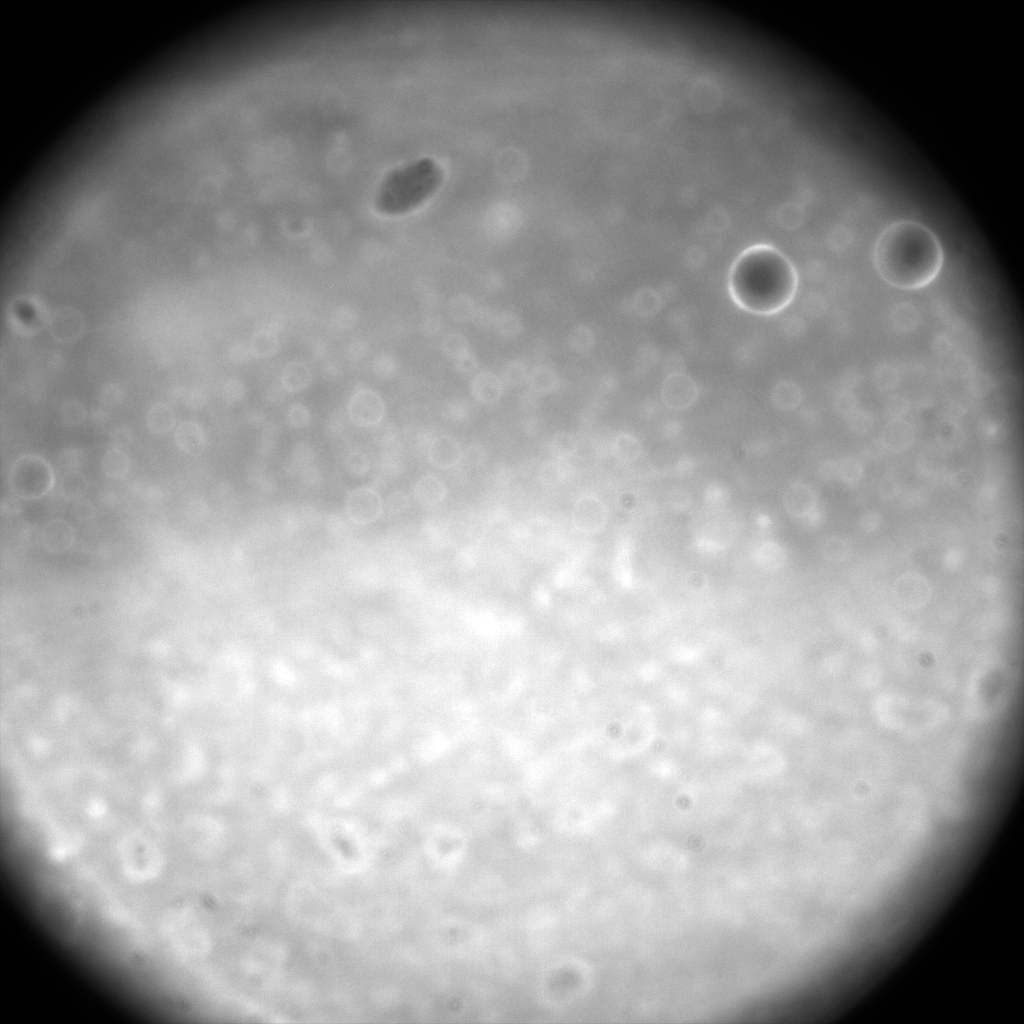

Supplement: S8 File — Zip file archive containing original photomicrographs of polymersomes formed following rehydration at 24°C. (ZIP) [file pone.0158729.s008.zip › 24C/Image_4313_20150316_133906.tif]

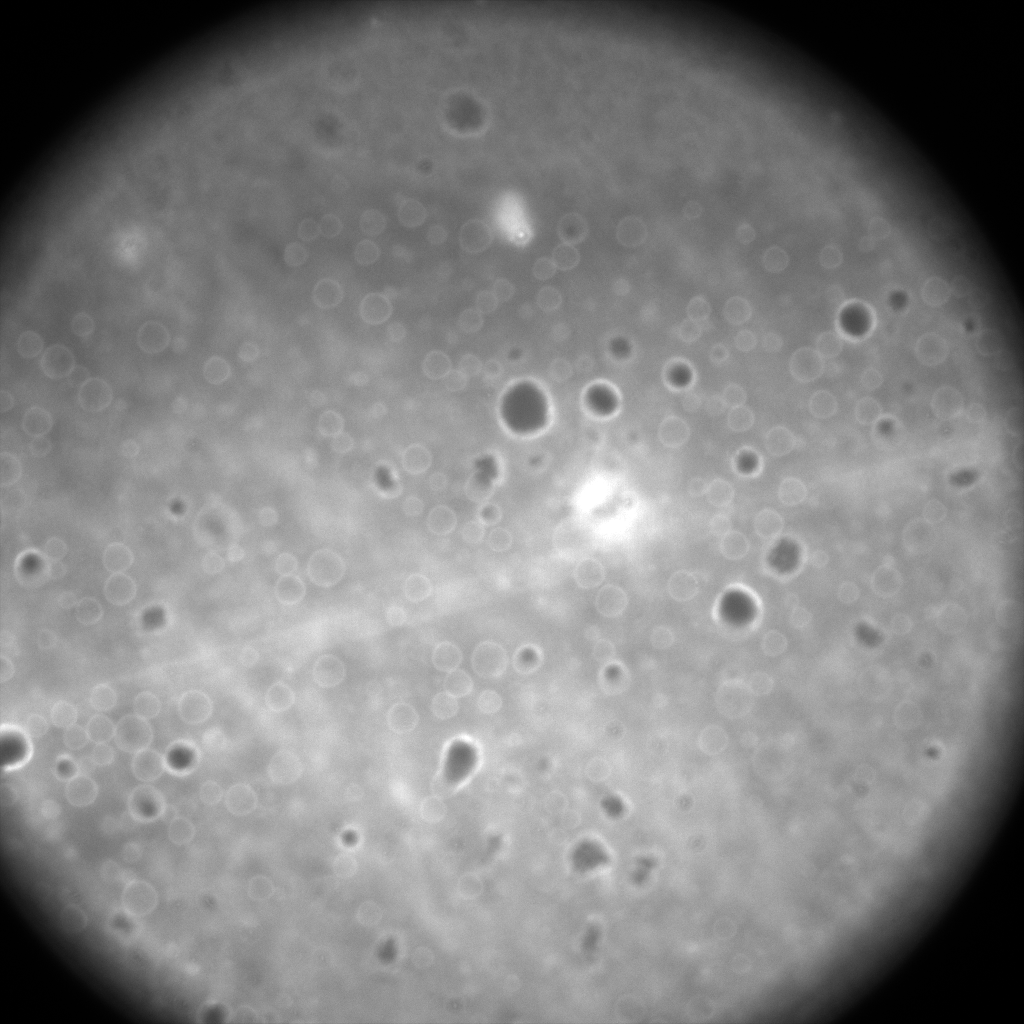

Supplement: S8 File — Zip file archive containing original photomicrographs of polymersomes formed following rehydration at 24°C. (ZIP) [file pone.0158729.s008.zip › 24C/Image_4314_20150316_134001.tif]

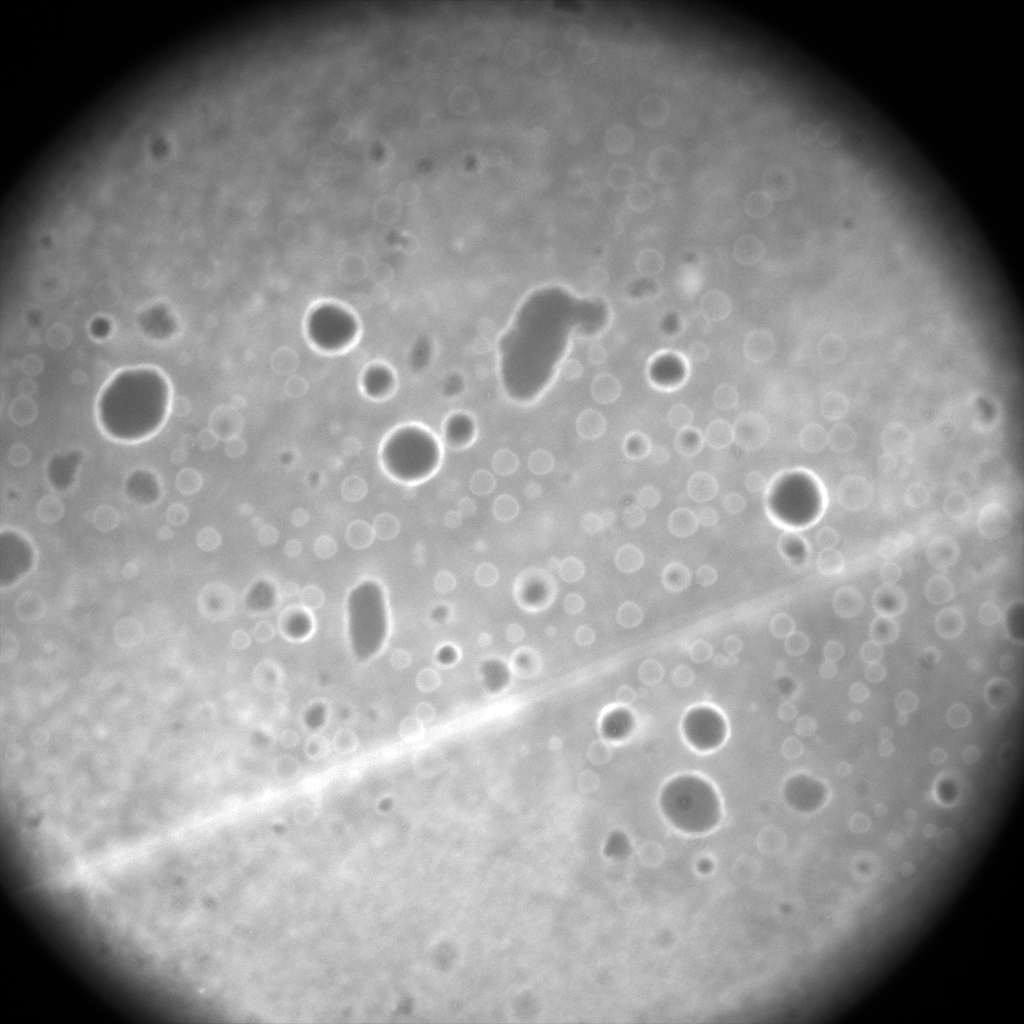

Supplement: S8 File — Zip file archive containing original photomicrographs of polymersomes formed following rehydration at 24°C. (ZIP) [file pone.0158729.s008.zip › 24C/Image_4315_20150316_134015.tif]

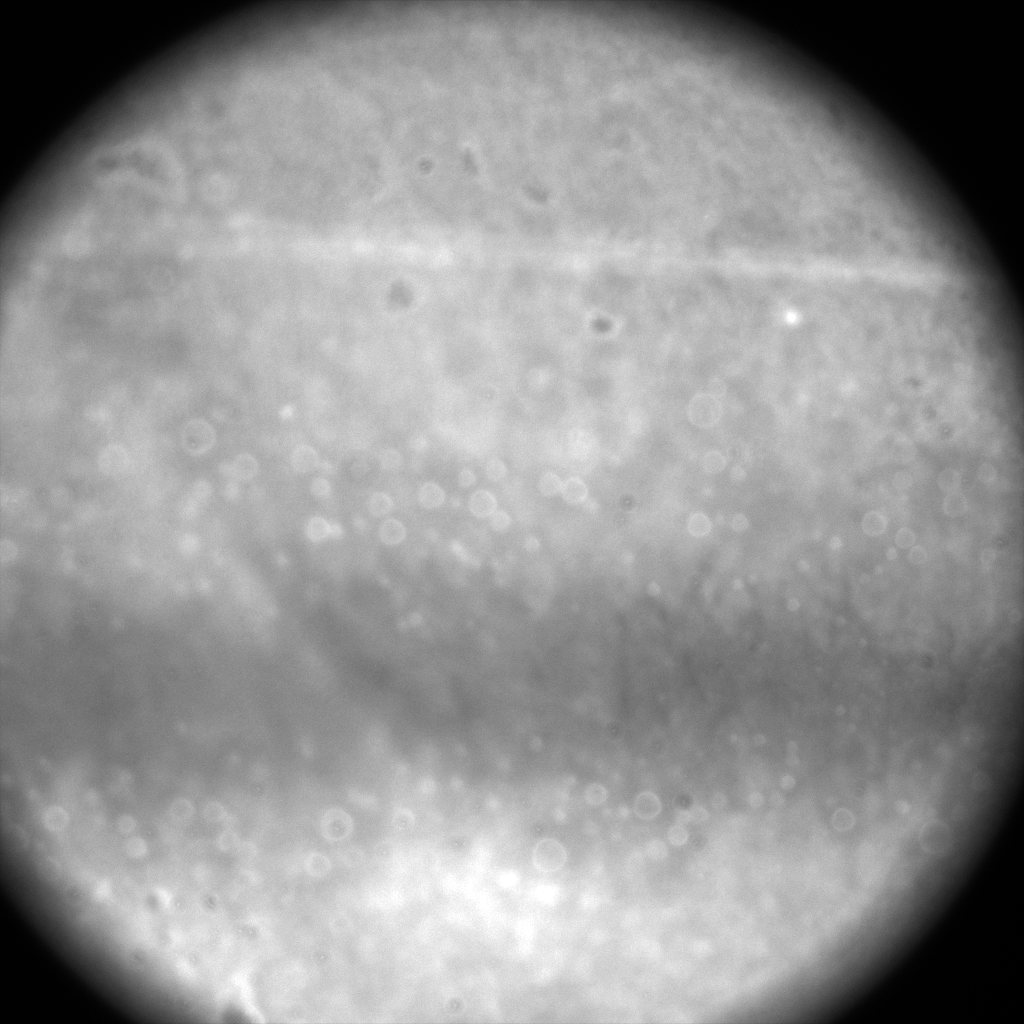

Supplement: S8 File — Zip file archive containing original photomicrographs of polymersomes formed following rehydration at 24°C. (ZIP) [file pone.0158729.s008.zip › 24C/Image_4316_20150316_134032.tif]

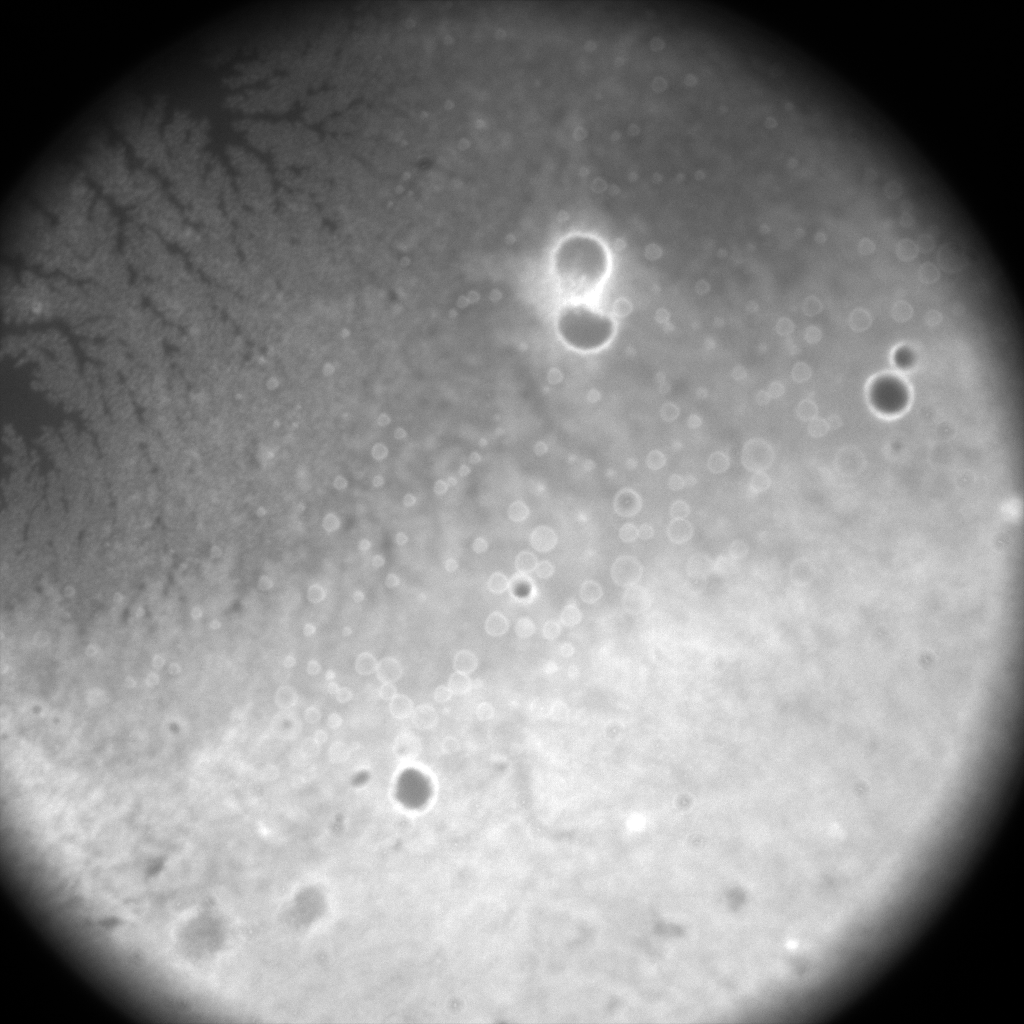

Supplement: S8 File — Zip file archive containing original photomicrographs of polymersomes formed following rehydration at 24°C. (ZIP) [file pone.0158729.s008.zip › 24C/Image_4317_20150316_134204.tif]

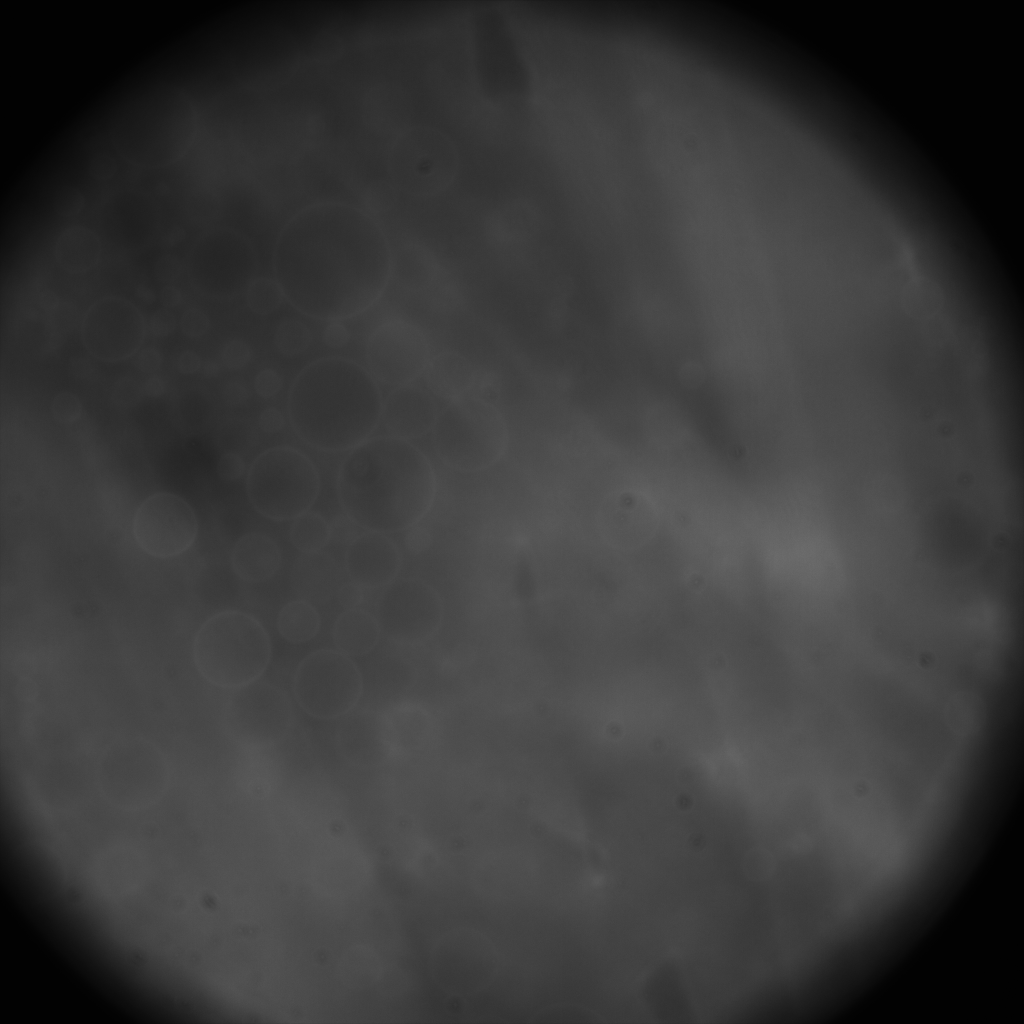

Supplement: S10 File — Zip file archive containing original photomicrographs of polymersomes formed following rehydration at 50°C. (ZIP) [file pone.0158729.s010.zip › 50C/Image_4071_20150311_172804.tif]

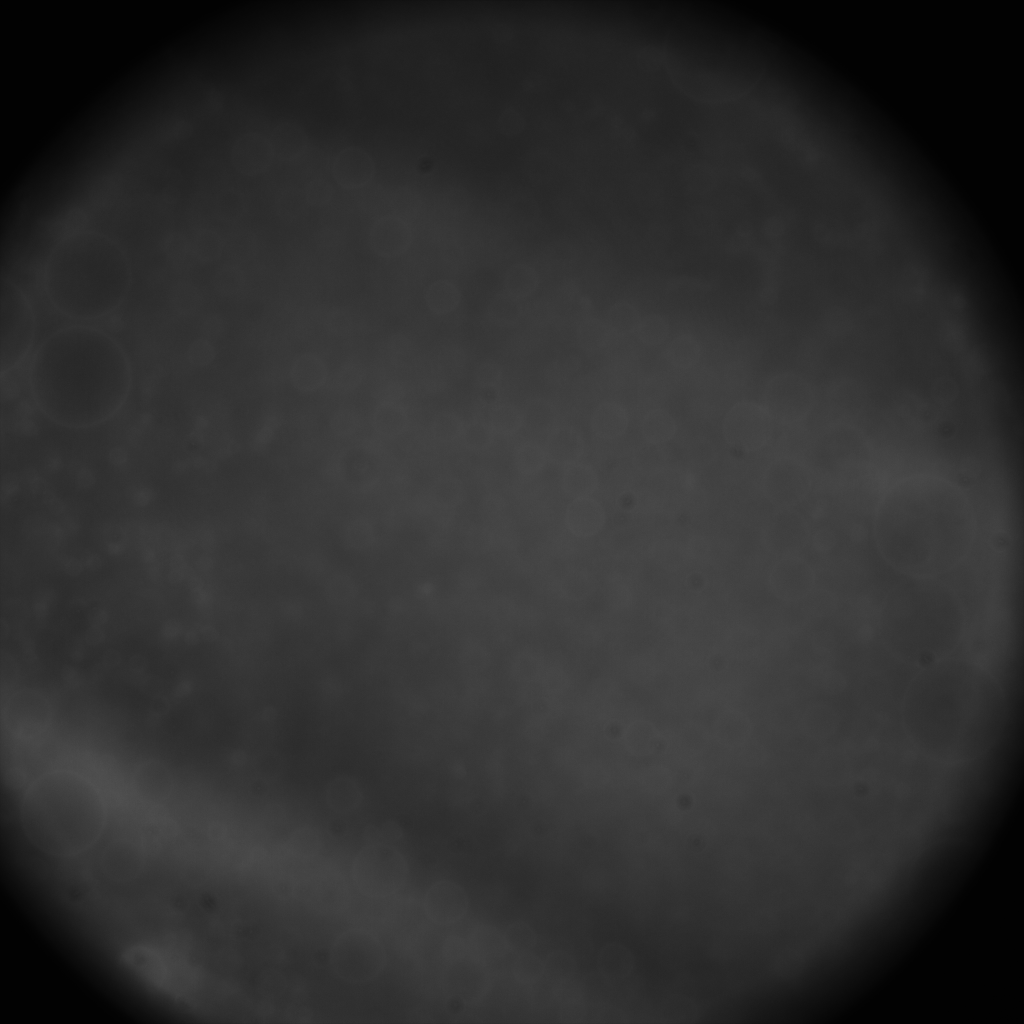

Supplement: S10 File — Zip file archive containing original photomicrographs of polymersomes formed following rehydration at 50°C. (ZIP) [file pone.0158729.s010.zip › 50C/Image_4073_20150311_172824.tif]

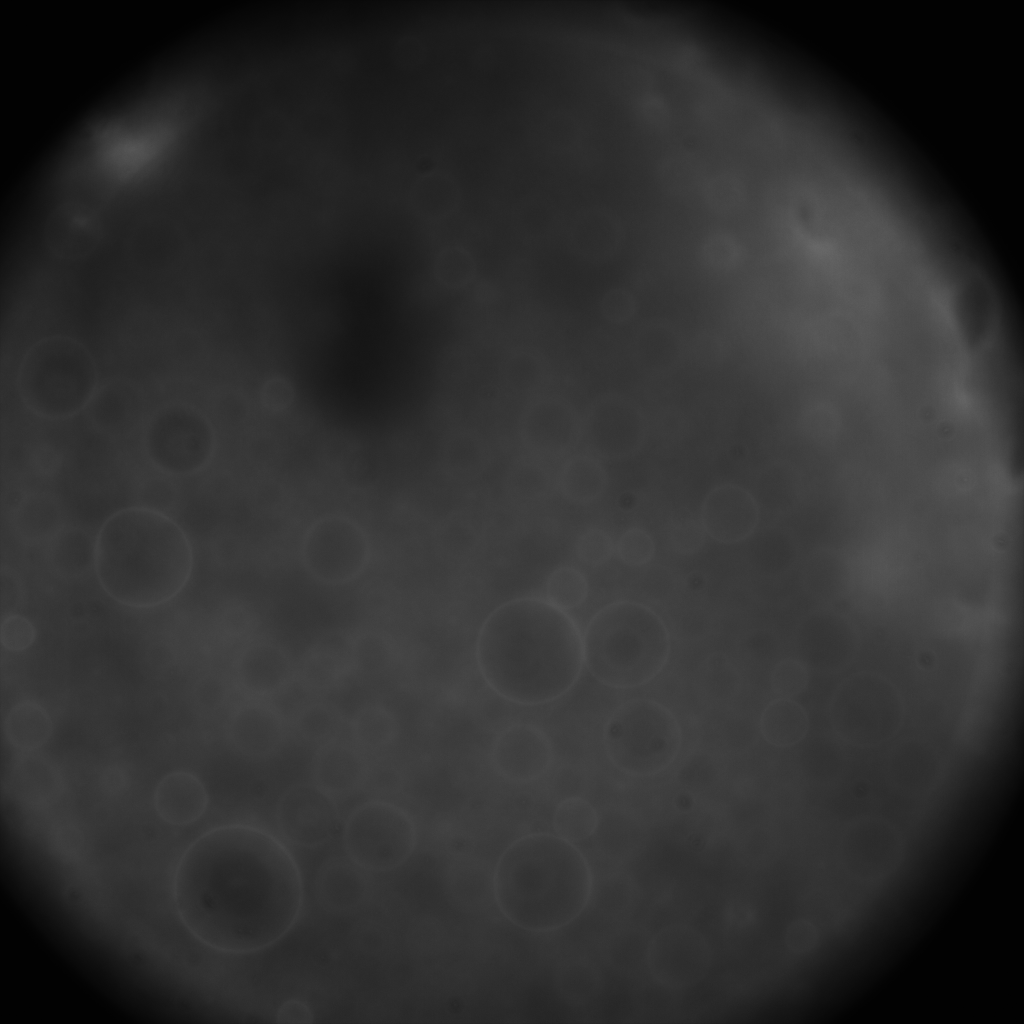

Supplement: S10 File — Zip file archive containing original photomicrographs of polymersomes formed following rehydration at 50°C. (ZIP) [file pone.0158729.s010.zip › 50C/Image_4075_20150311_172845.tif]

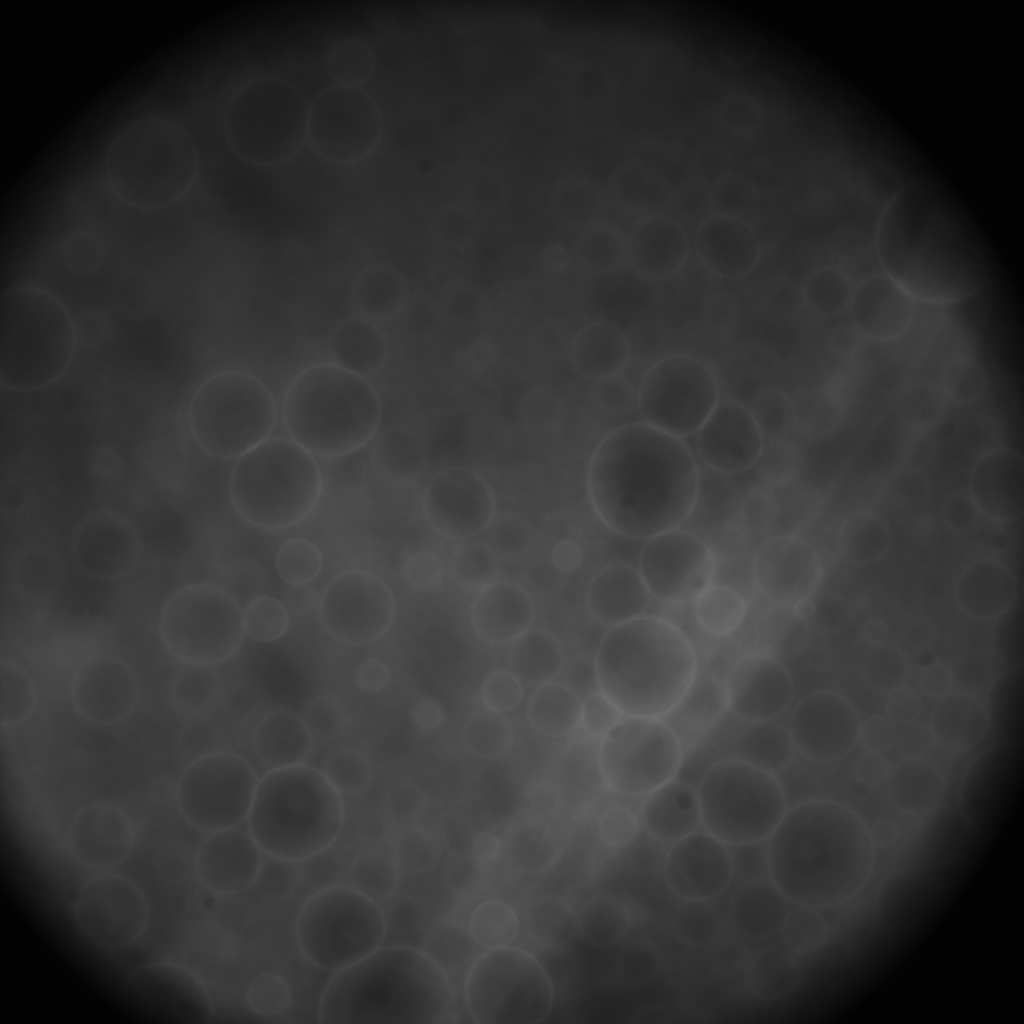

Supplement: S10 File — Zip file archive containing original photomicrographs of polymersomes formed following rehydration at 50°C. (ZIP) [file pone.0158729.s010.zip › 50C/Image_4077_20150311_172916.tif]

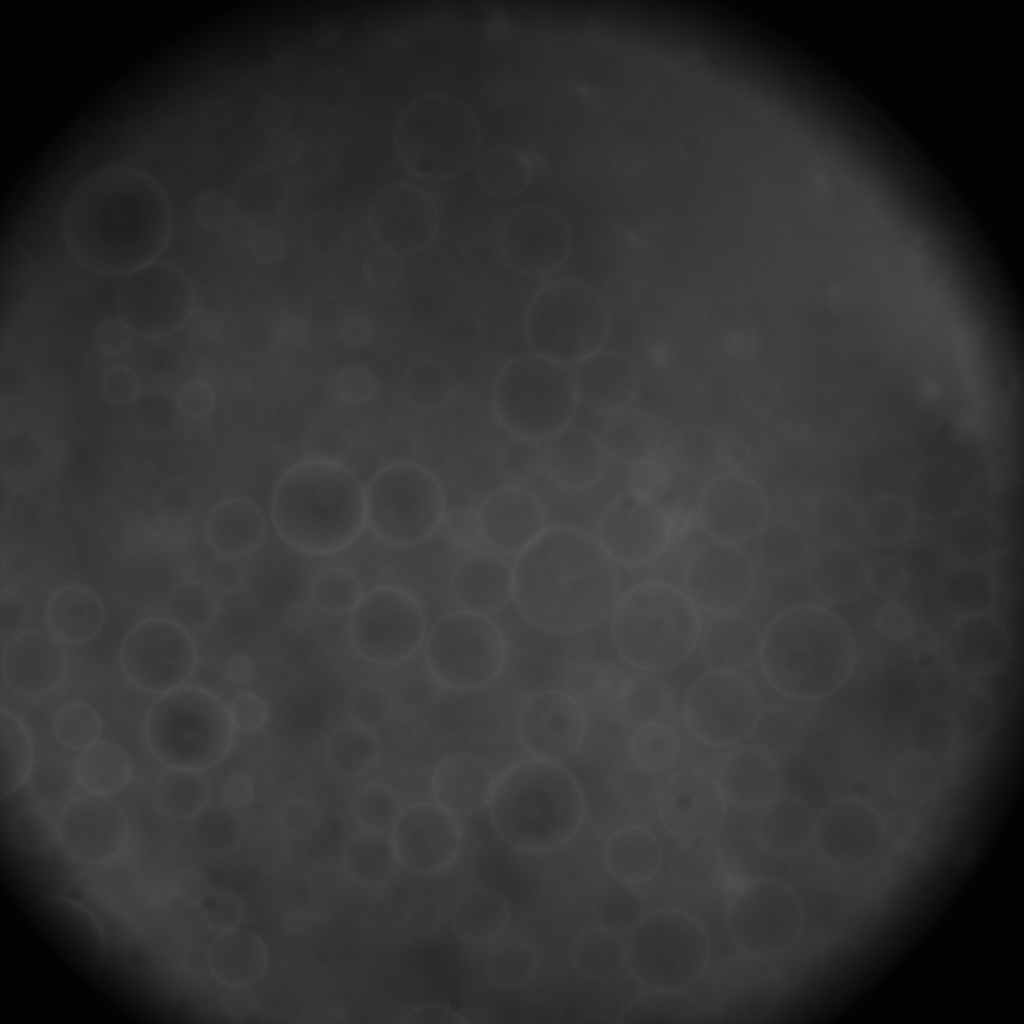

Supplement: S10 File — Zip file archive containing original photomicrographs of polymersomes formed following rehydration at 50°C. (ZIP) [file pone.0158729.s010.zip › 50C/Image_4079_20150311_172931.tif]

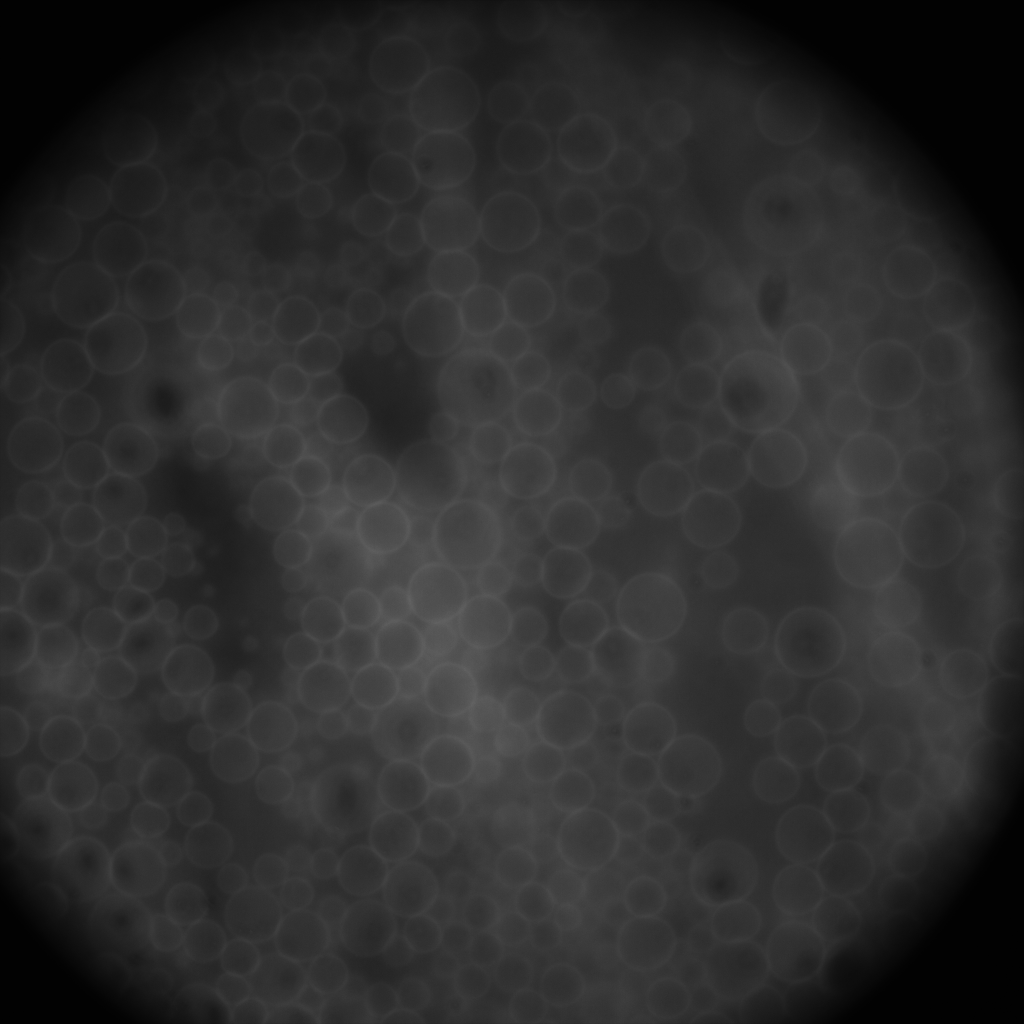

Supplement: S10 File — Zip file archive containing original photomicrographs of polymersomes formed following rehydration at 50°C. (ZIP) [file pone.0158729.s010.zip › 50C/Image_4081_20150311_172958.tif]

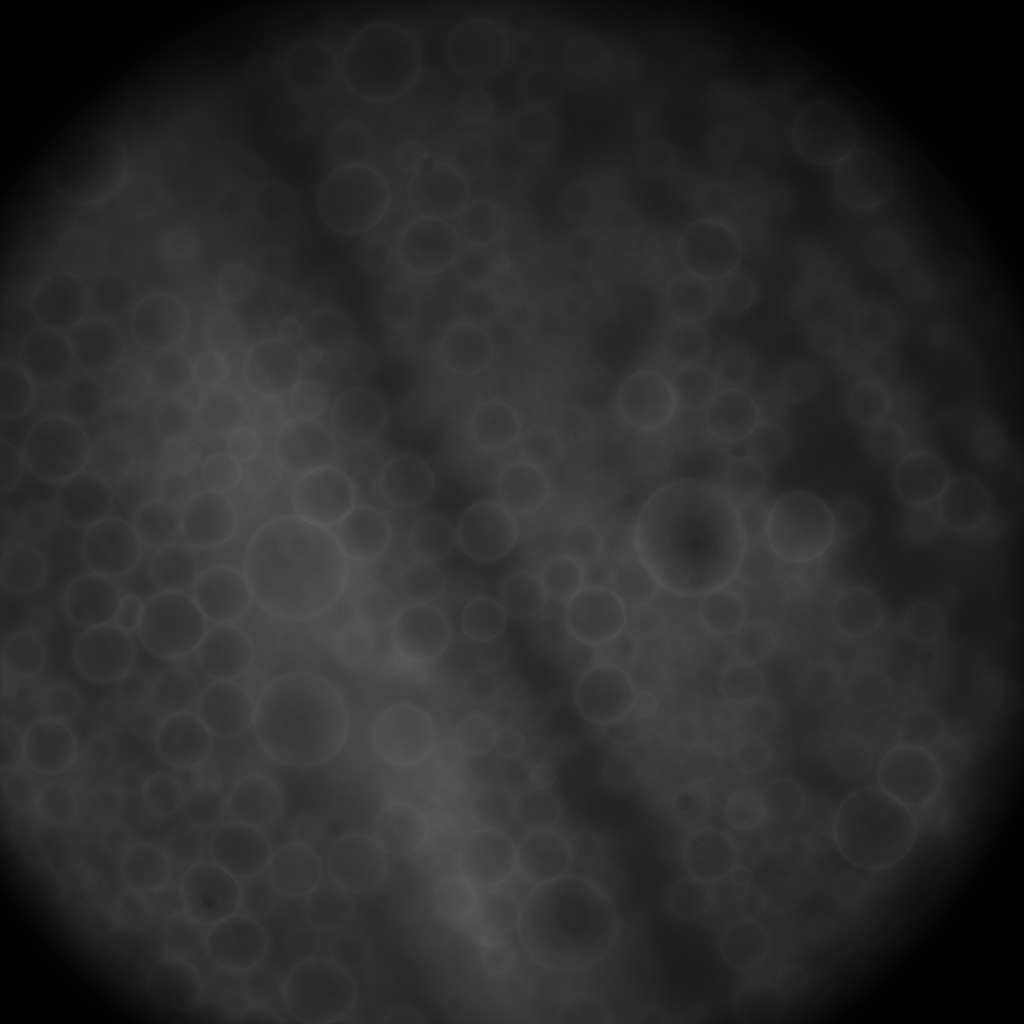

Supplement: S10 File — Zip file archive containing original photomicrographs of polymersomes formed following rehydration at 50°C. (ZIP) [file pone.0158729.s010.zip › 50C/Image_4082_20150311_173036.tif]

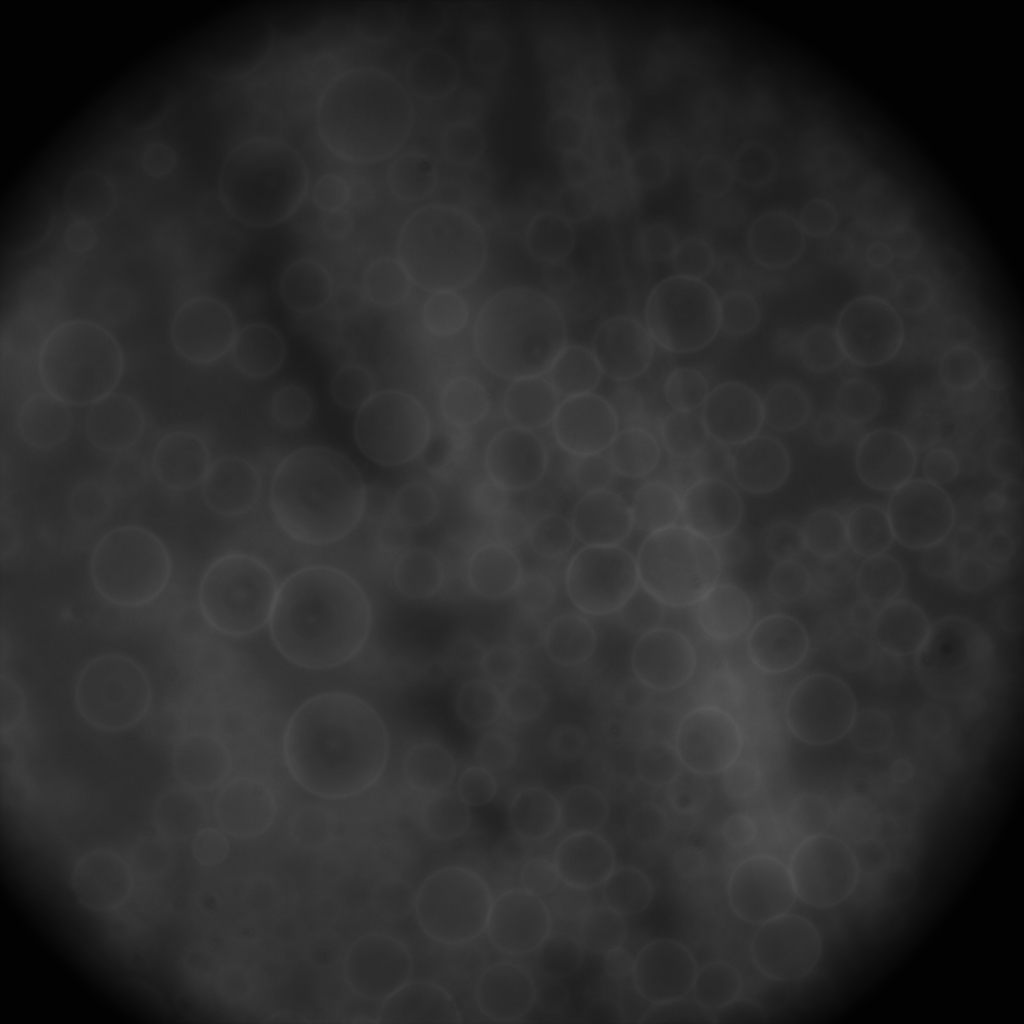

Supplement: S10 File — Zip file archive containing original photomicrographs of polymersomes formed following rehydration at 50°C. (ZIP) [file pone.0158729.s010.zip › 50C/Image_4083_20150311_173050.tif]

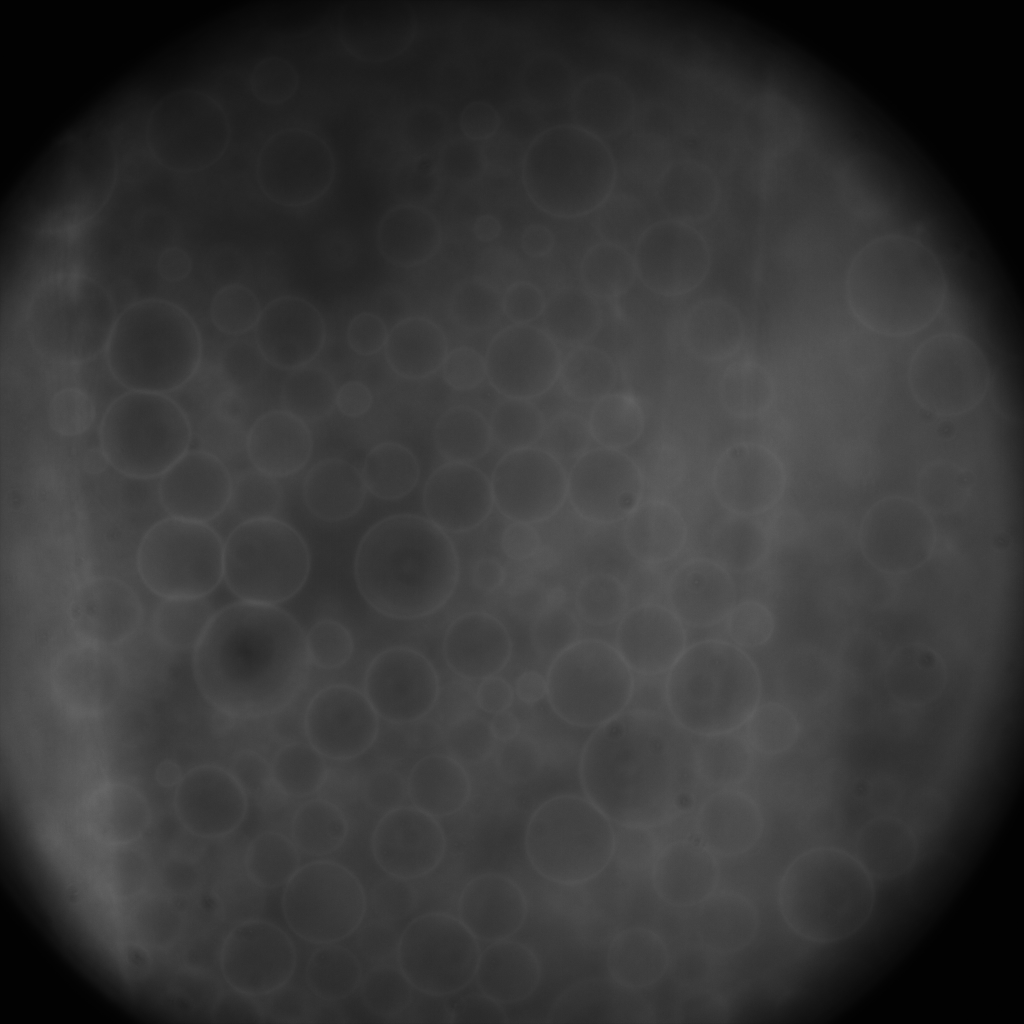

Supplement: S10 File — Zip file archive containing original photomicrographs of polymersomes formed following rehydration at 50°C. (ZIP) [file pone.0158729.s010.zip › 50C/Image_4084_20150311_173104.tif]

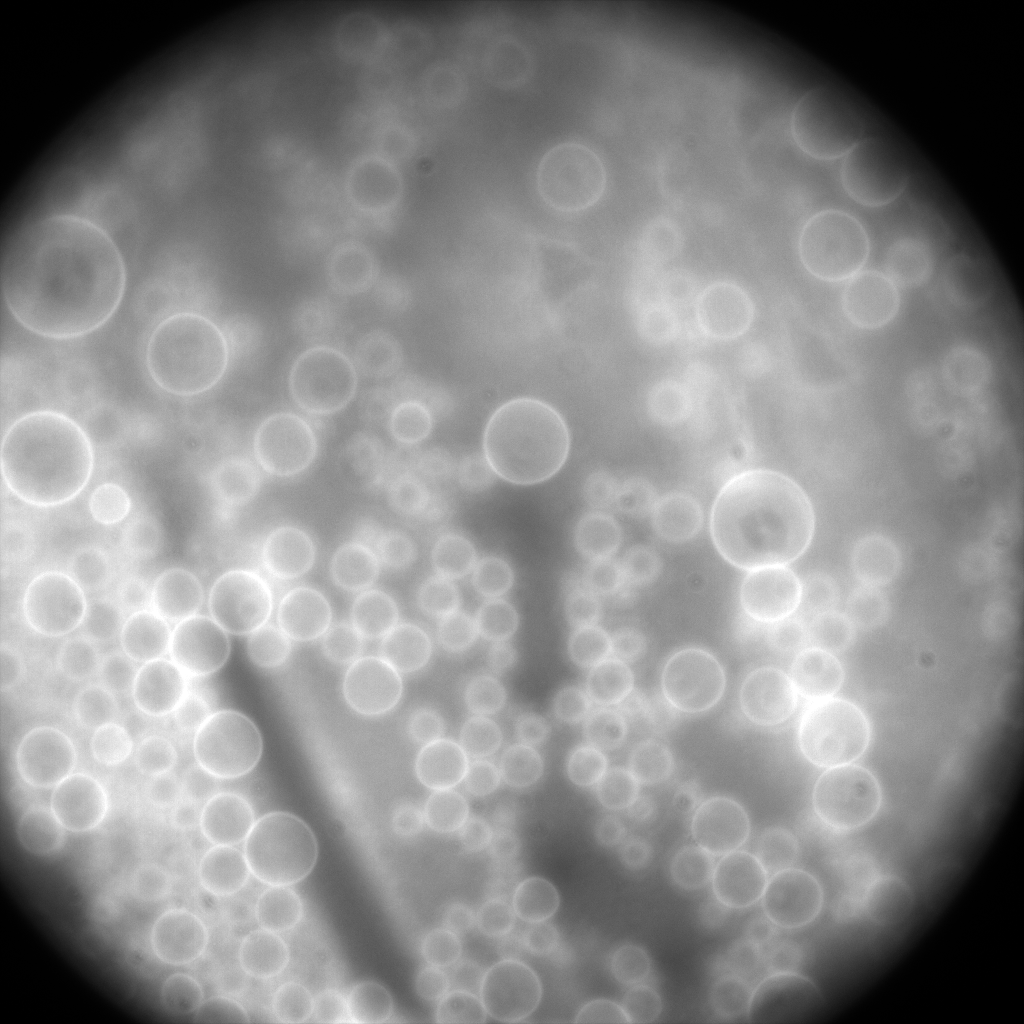

Supplement: S10 File — Zip file archive containing original photomicrographs of polymersomes formed following rehydration at 50°C. (ZIP) [file pone.0158729.s010.zip › 50C/Image_4100_20150311_174555.tif]

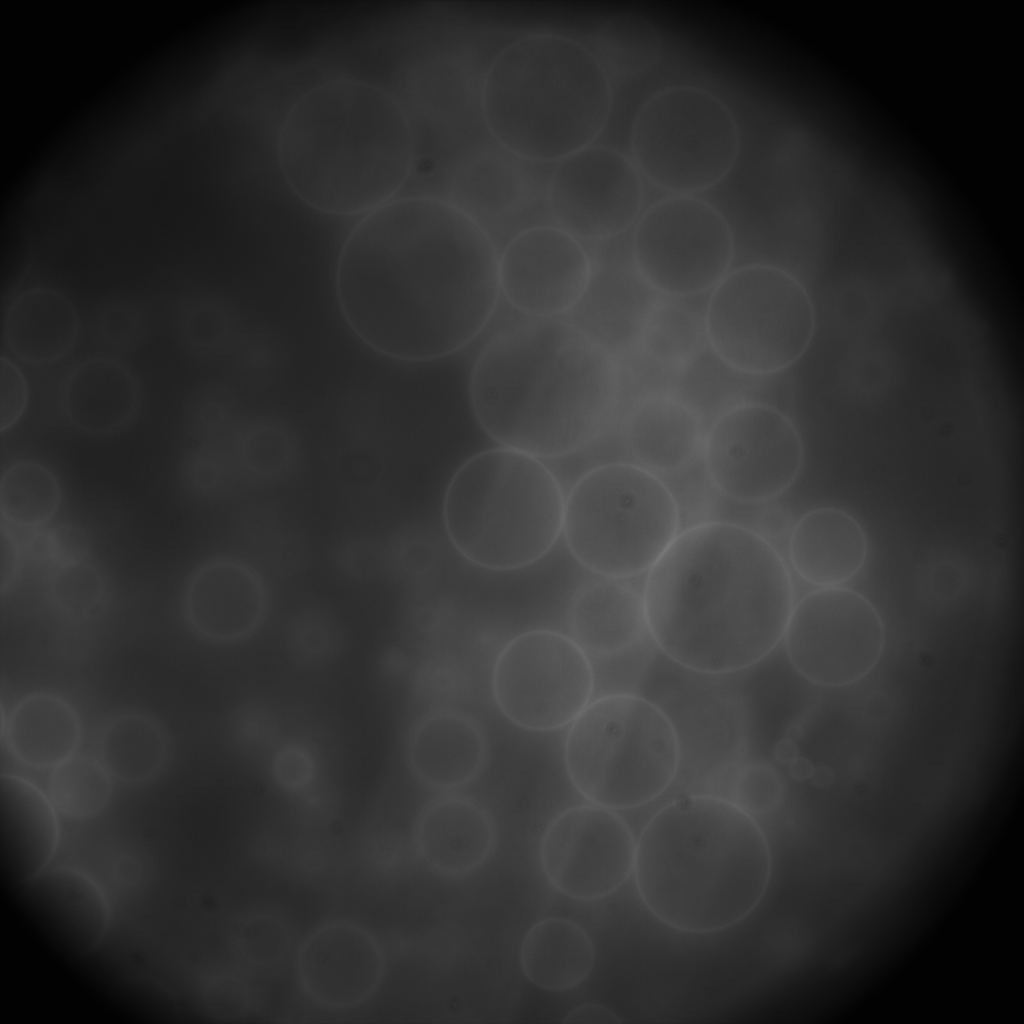

Supplement: S11 File — Zip file archive containing original photomicrographs of polymersomes formed following rehydration at 60°C. (ZIP) [file pone.0158729.s011.zip › 60C/Image_4085_20150311_173204.tif]

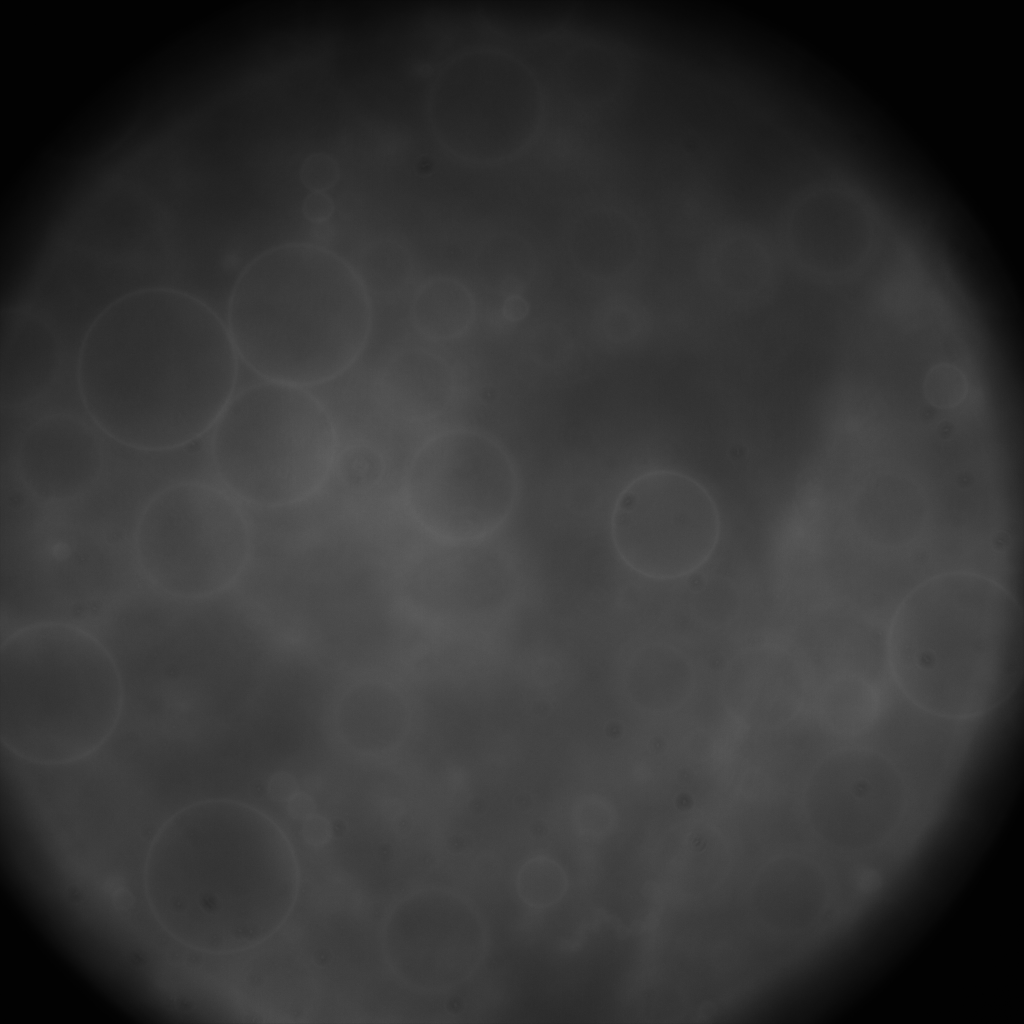

Supplement: S11 File — Zip file archive containing original photomicrographs of polymersomes formed following rehydration at 60°C. (ZIP) [file pone.0158729.s011.zip › 60C/Image_4087_20150311_173432.tif]

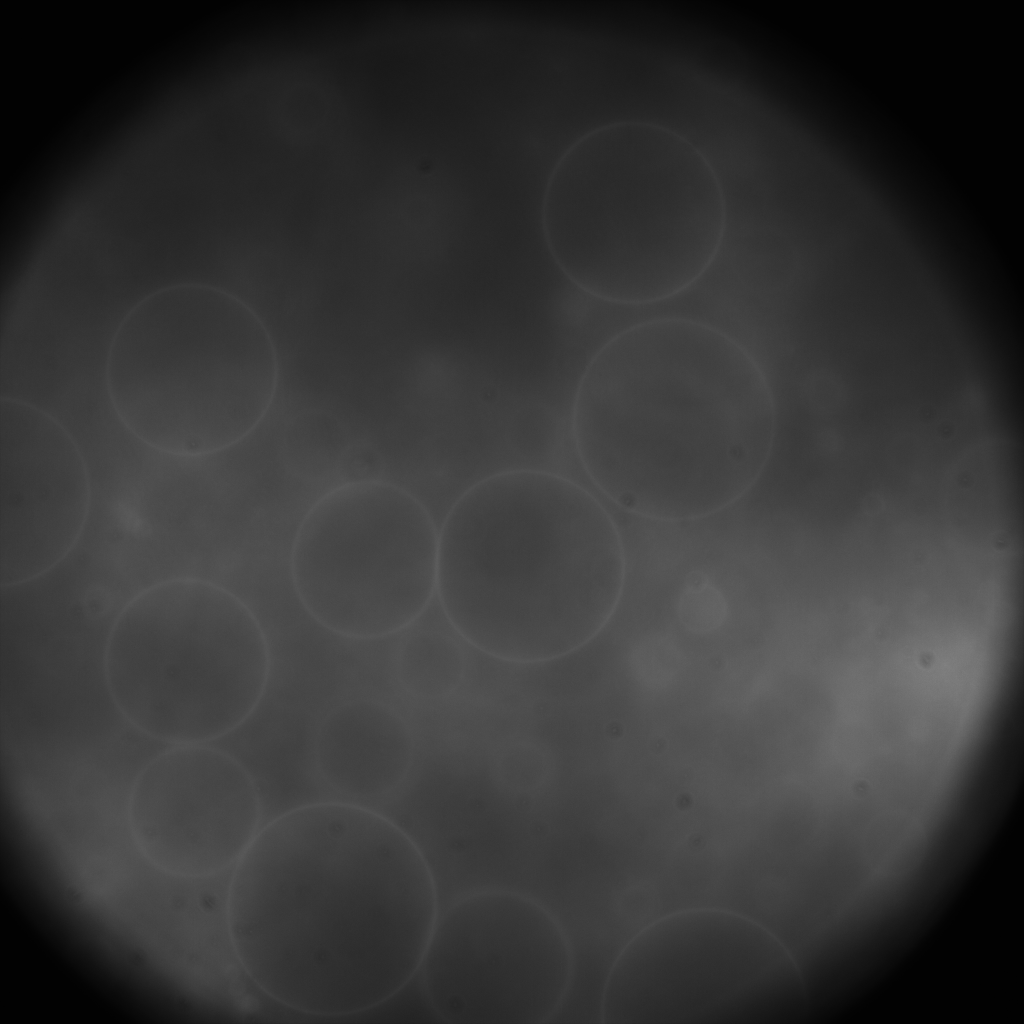

Supplement: S11 File — Zip file archive containing original photomicrographs of polymersomes formed following rehydration at 60°C. (ZIP) [file pone.0158729.s011.zip › 60C/Image_4089_20150311_173517.tif]

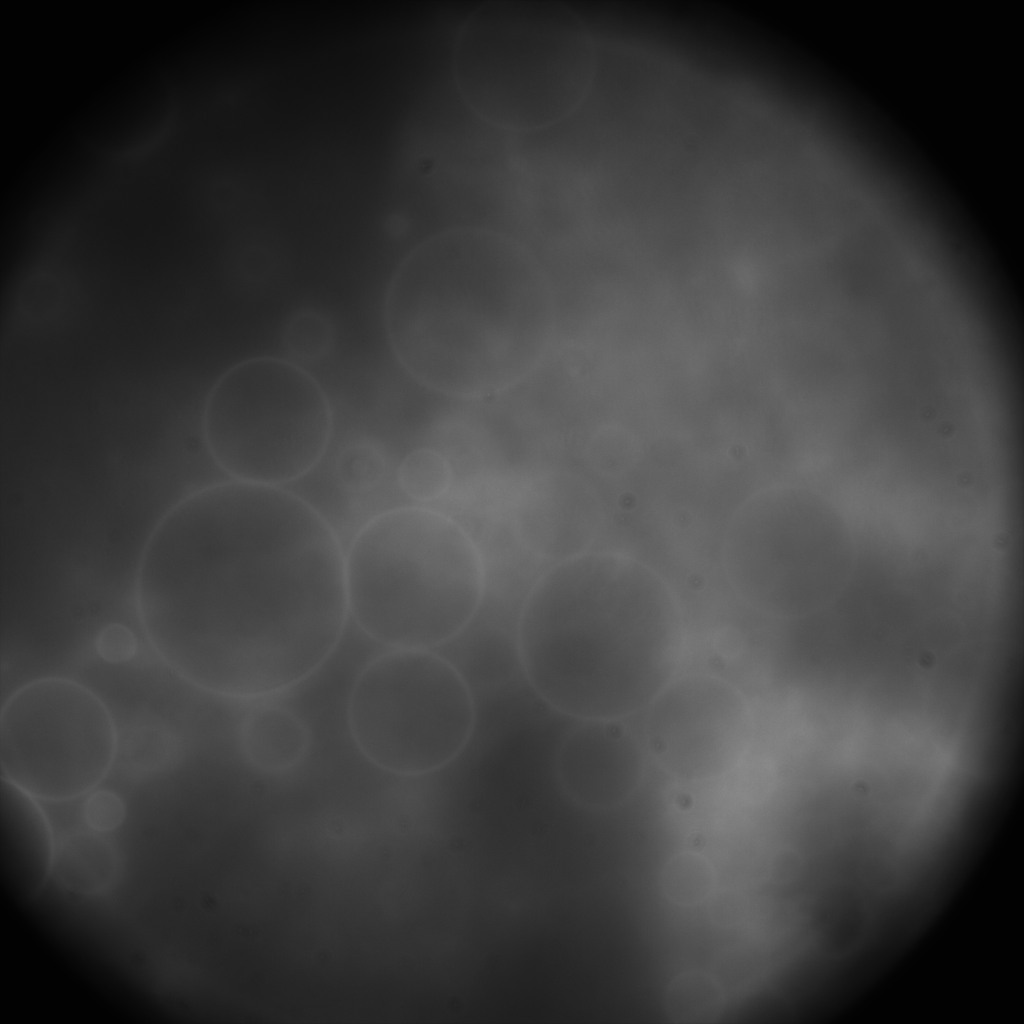

Supplement: S11 File — Zip file archive containing original photomicrographs of polymersomes formed following rehydration at 60°C. (ZIP) [file pone.0158729.s011.zip › 60C/Image_4091_20150311_173641.tif]

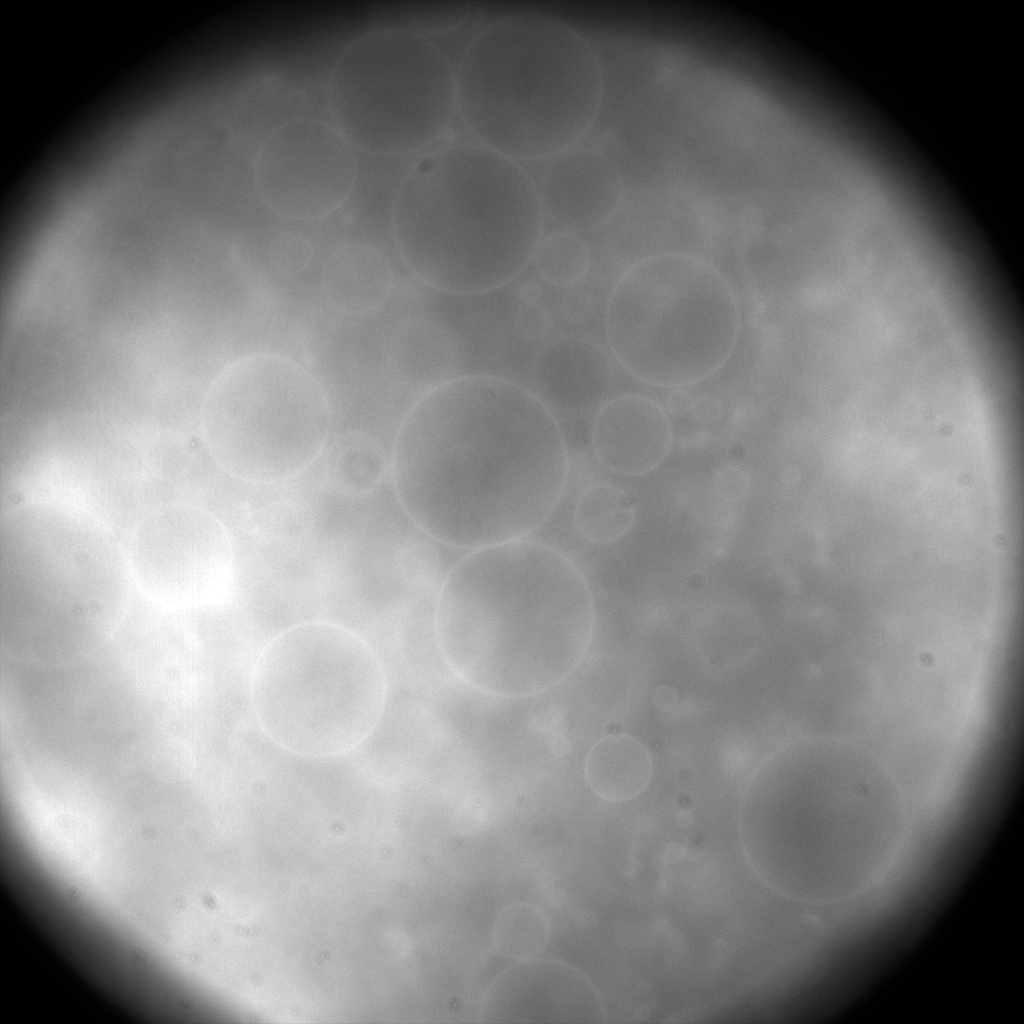

Supplement: S11 File — Zip file archive containing original photomicrographs of polymersomes formed following rehydration at 60°C. (ZIP) [file pone.0158729.s011.zip › 60C/Image_4093_20150311_173723.tif]

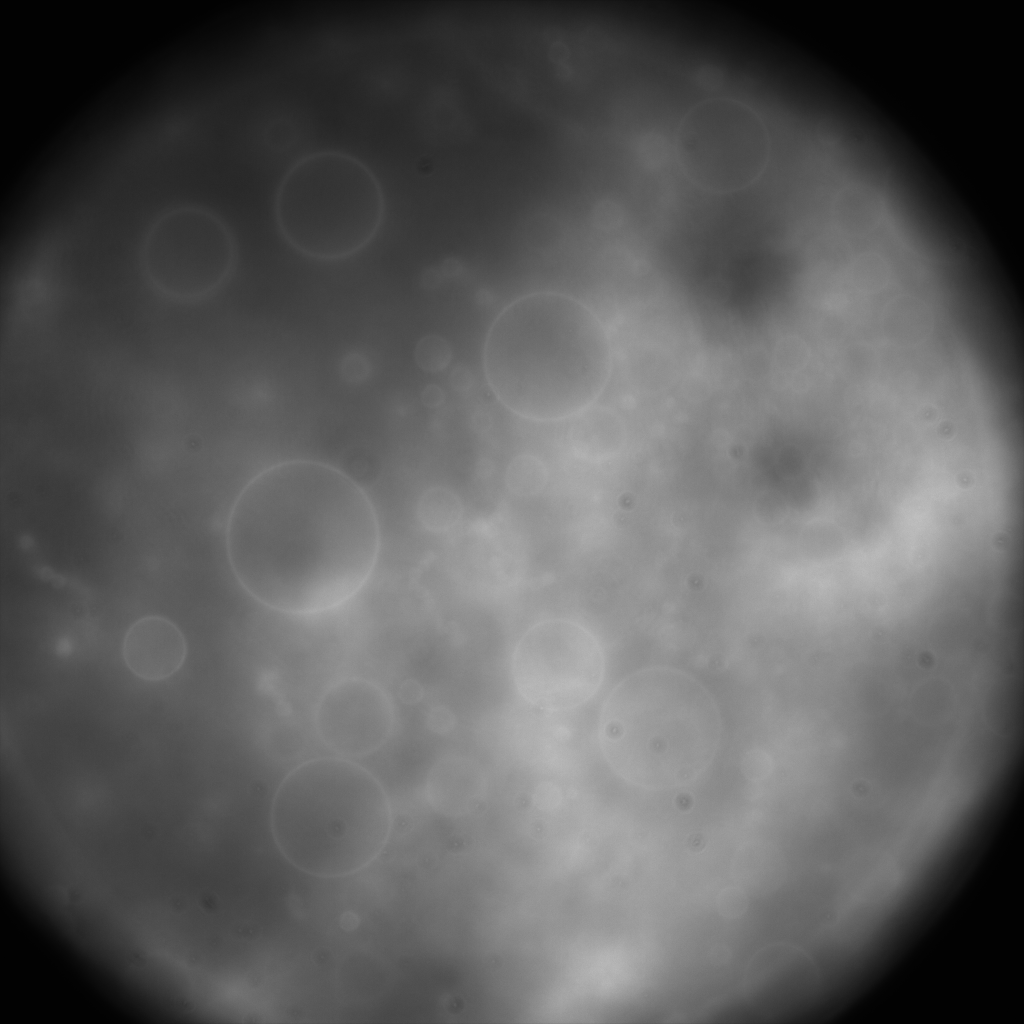

Supplement: S11 File — Zip file archive containing original photomicrographs of polymersomes formed following rehydration at 60°C. (ZIP) [file pone.0158729.s011.zip › 60C/Image_4095_20150311_173756.tif]

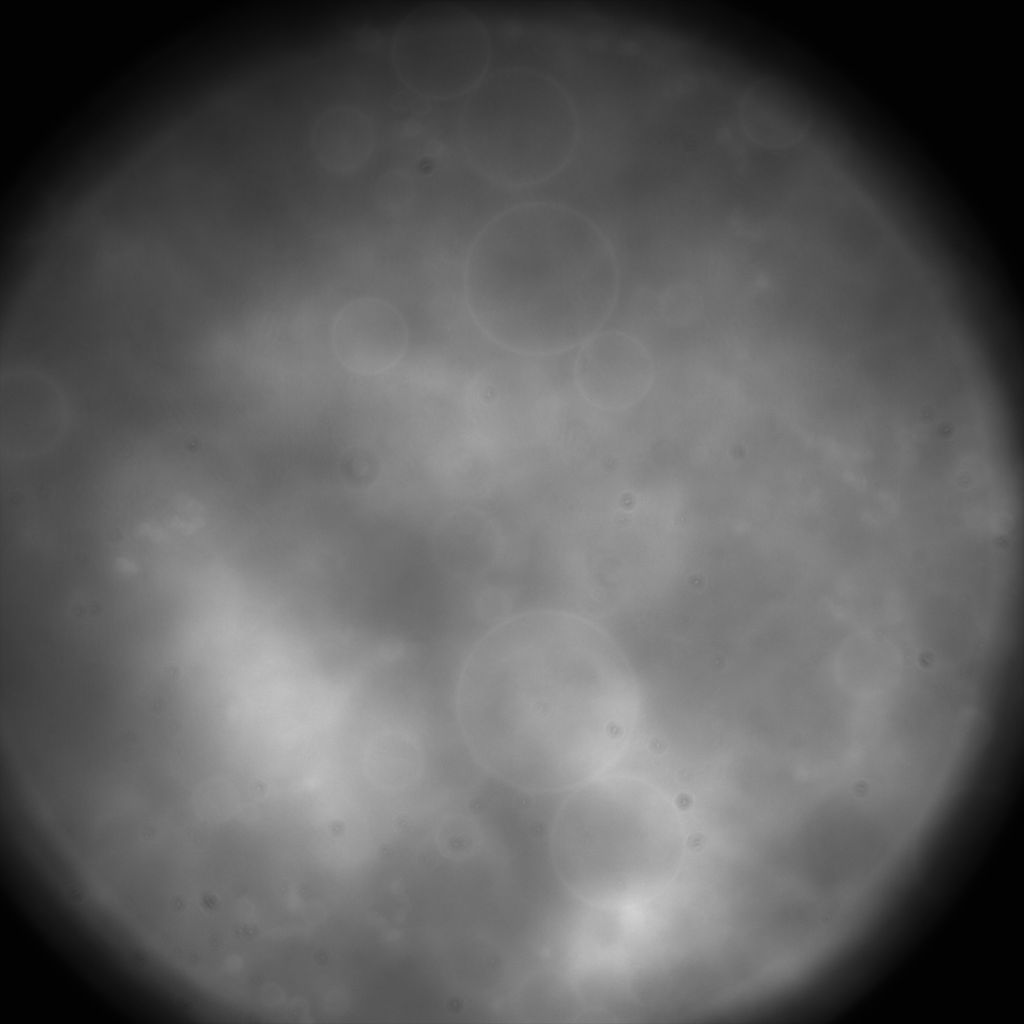

Supplement: S11 File — Zip file archive containing original photomicrographs of polymersomes formed following rehydration at 60°C. (ZIP) [file pone.0158729.s011.zip › 60C/Image_4096_20150311_173810.tif]

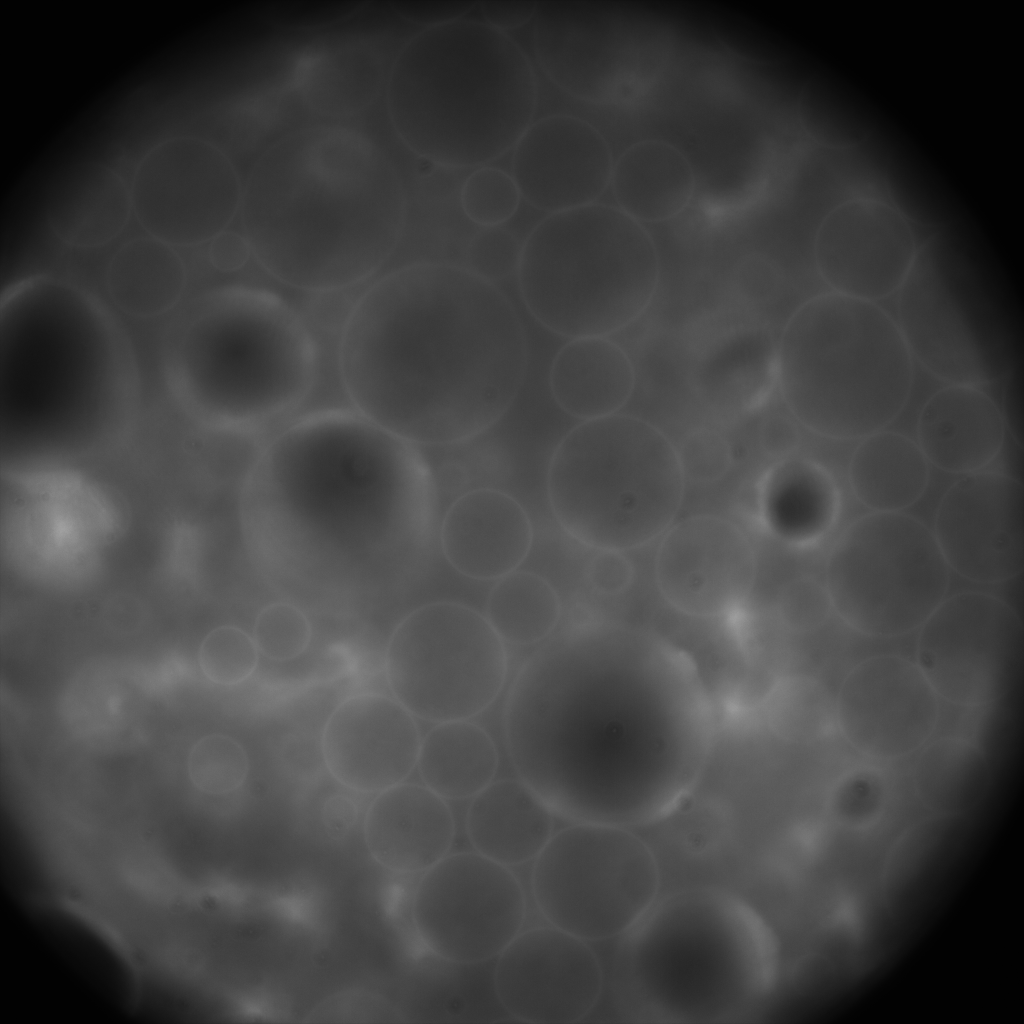

Supplement: S11 File — Zip file archive containing original photomicrographs of polymersomes formed following rehydration at 60°C. (ZIP) [file pone.0158729.s011.zip › 60C/Image_4097_20150311_173829.tif]
