# Supplementary figures and images for: The Role of Membrane Fluidization in the Gel-Assisted Formation of Giant Polymersomes (part 1 of 3)
Source: PLoS One. 2016 Jul 13;11(7):e0158729. doi: 10.1371/journal.pone.0158729 (PMC4943728; doi:10.1371/journal.pone.0158729)

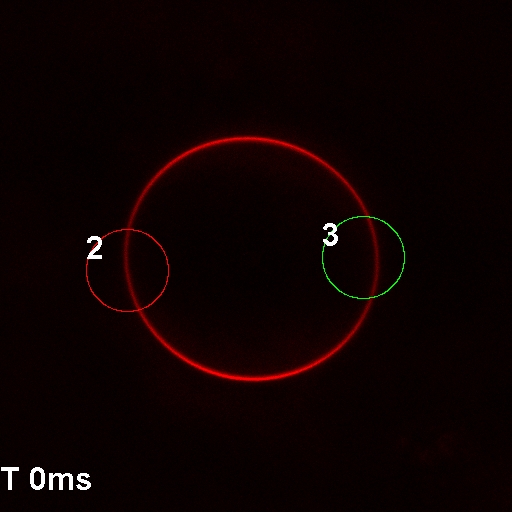

Supplement: S3 File — Zip file archive containing original photomicrographs obtained in Fluorescence Recovery After Photobleaching (FRAP) experiments. (ZIP) [file pone.0158729.s003.zip › PEO-PBD FRAP Fast/P2904 on surface Free Run 1_C001T001.jpg]

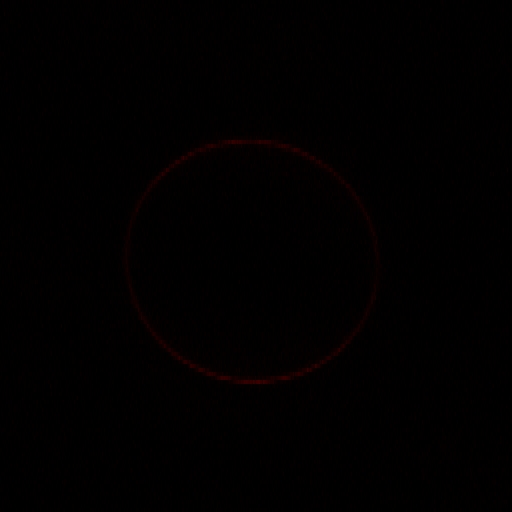

Supplement: S3 File — Zip file archive containing original photomicrographs obtained in Fluorescence Recovery After Photobleaching (FRAP) experiments. (ZIP) [file pone.0158729.s003.zip › PEO-PBD FRAP Fast/P2904 on surface Free Run 1_C001T001-R001.jpg]

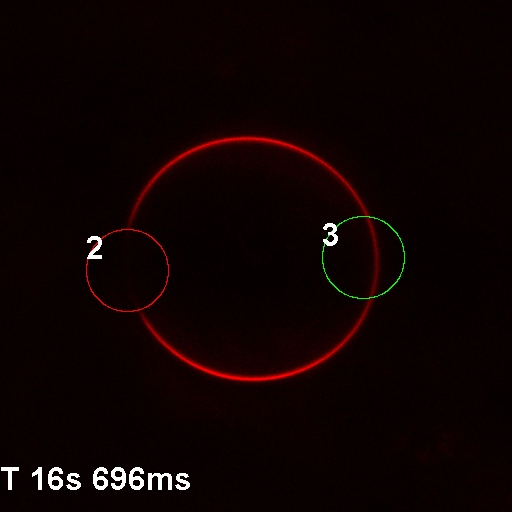

Supplement: S3 File — Zip file archive containing original photomicrographs obtained in Fluorescence Recovery After Photobleaching (FRAP) experiments. (ZIP) [file pone.0158729.s003.zip › PEO-PBD FRAP Fast/P2904 on surface Free Run 1_C001T002.jpg]

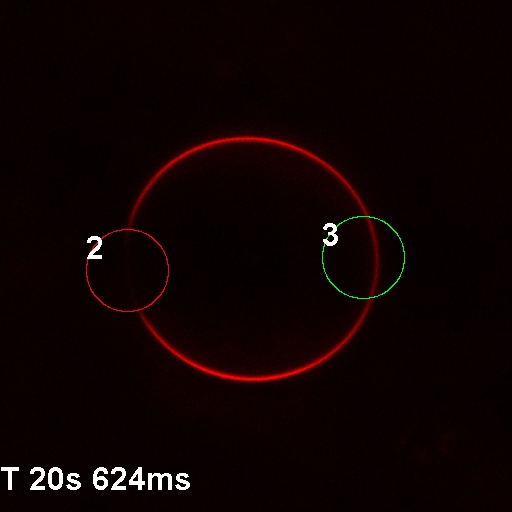

Supplement: S3 File — Zip file archive containing original photomicrographs obtained in Fluorescence Recovery After Photobleaching (FRAP) experiments. (ZIP) [file pone.0158729.s003.zip › PEO-PBD FRAP Fast/P2904 on surface Free Run 1_C001T003.jpg]

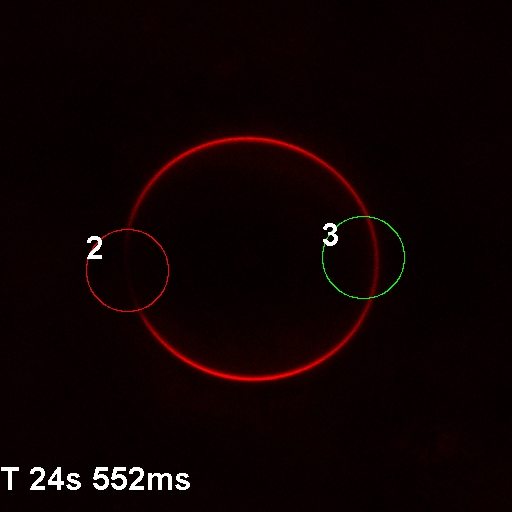

Supplement: S3 File — Zip file archive containing original photomicrographs obtained in Fluorescence Recovery After Photobleaching (FRAP) experiments. (ZIP) [file pone.0158729.s003.zip › PEO-PBD FRAP Fast/P2904 on surface Free Run 1_C001T004.jpg]

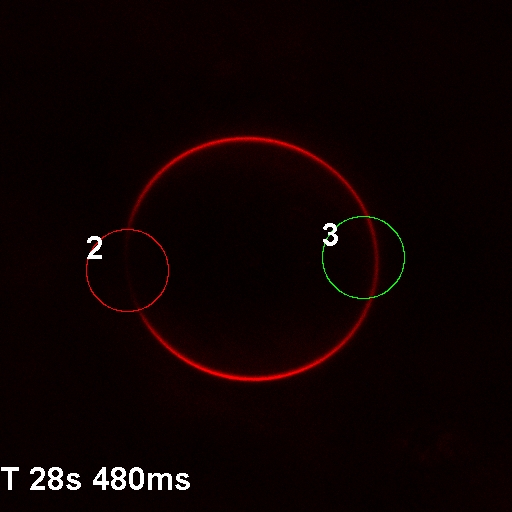

Supplement: S3 File — Zip file archive containing original photomicrographs obtained in Fluorescence Recovery After Photobleaching (FRAP) experiments. (ZIP) [file pone.0158729.s003.zip › PEO-PBD FRAP Fast/P2904 on surface Free Run 1_C001T005.jpg]

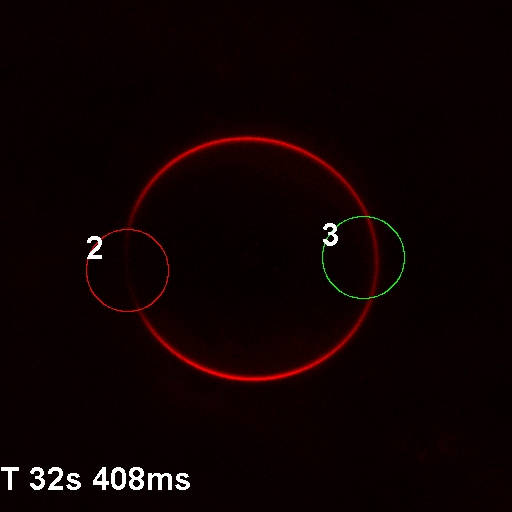

Supplement: S3 File — Zip file archive containing original photomicrographs obtained in Fluorescence Recovery After Photobleaching (FRAP) experiments. (ZIP) [file pone.0158729.s003.zip › PEO-PBD FRAP Fast/P2904 on surface Free Run 1_C001T006.jpg]

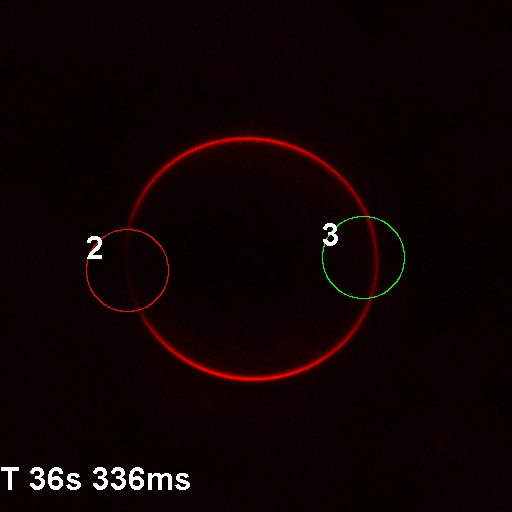

Supplement: S3 File — Zip file archive containing original photomicrographs obtained in Fluorescence Recovery After Photobleaching (FRAP) experiments. (ZIP) [file pone.0158729.s003.zip › PEO-PBD FRAP Fast/P2904 on surface Free Run 1_C001T007.jpg]

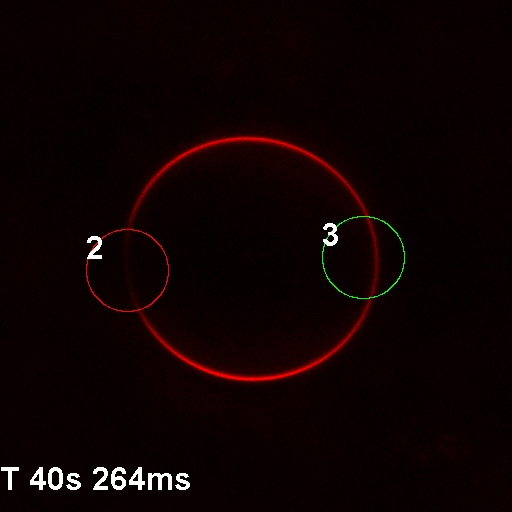

Supplement: S3 File — Zip file archive containing original photomicrographs obtained in Fluorescence Recovery After Photobleaching (FRAP) experiments. (ZIP) [file pone.0158729.s003.zip › PEO-PBD FRAP Fast/P2904 on surface Free Run 1_C001T008.jpg]

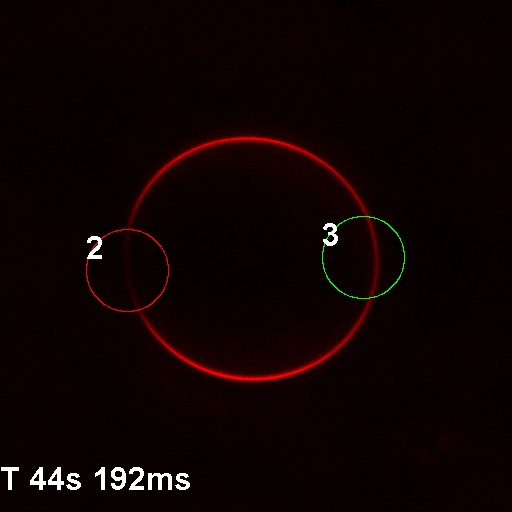

Supplement: S3 File — Zip file archive containing original photomicrographs obtained in Fluorescence Recovery After Photobleaching (FRAP) experiments. (ZIP) [file pone.0158729.s003.zip › PEO-PBD FRAP Fast/P2904 on surface Free Run 1_C001T009.jpg]

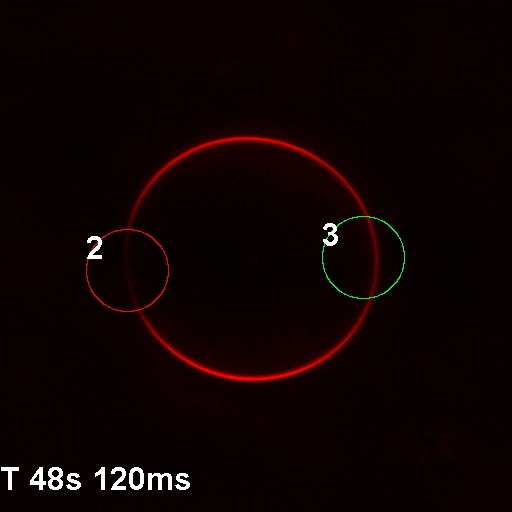

Supplement: S3 File — Zip file archive containing original photomicrographs obtained in Fluorescence Recovery After Photobleaching (FRAP) experiments. (ZIP) [file pone.0158729.s003.zip › PEO-PBD FRAP Fast/P2904 on surface Free Run 1_C001T010.jpg]

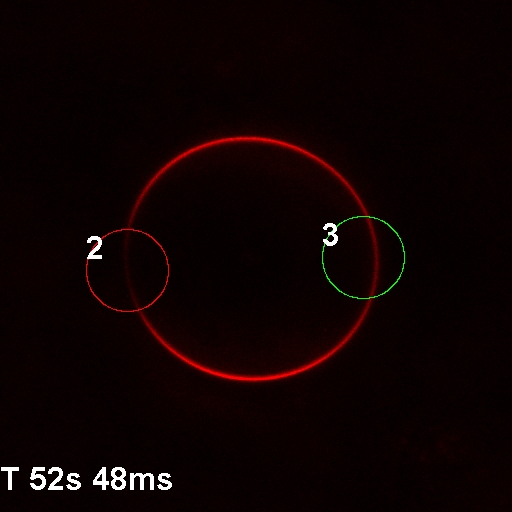

Supplement: S3 File — Zip file archive containing original photomicrographs obtained in Fluorescence Recovery After Photobleaching (FRAP) experiments. (ZIP) [file pone.0158729.s003.zip › PEO-PBD FRAP Fast/P2904 on surface Free Run 1_C001T011.jpg]

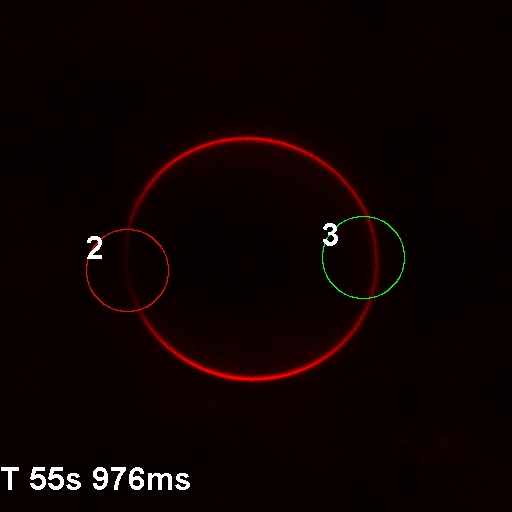

Supplement: S3 File — Zip file archive containing original photomicrographs obtained in Fluorescence Recovery After Photobleaching (FRAP) experiments. (ZIP) [file pone.0158729.s003.zip › PEO-PBD FRAP Fast/P2904 on surface Free Run 1_C001T012.jpg]

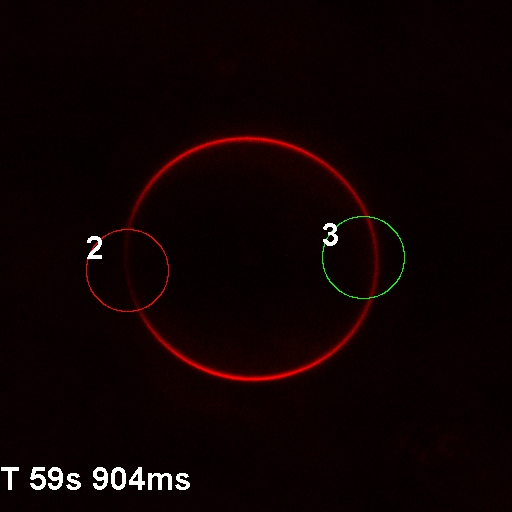

Supplement: S3 File — Zip file archive containing original photomicrographs obtained in Fluorescence Recovery After Photobleaching (FRAP) experiments. (ZIP) [file pone.0158729.s003.zip › PEO-PBD FRAP Fast/P2904 on surface Free Run 1_C001T013.jpg]

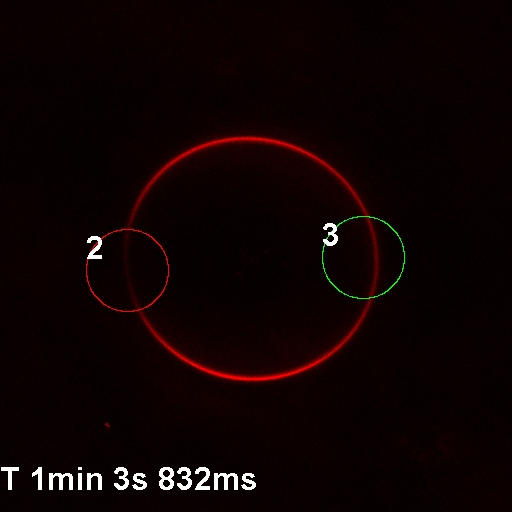

Supplement: S3 File — Zip file archive containing original photomicrographs obtained in Fluorescence Recovery After Photobleaching (FRAP) experiments. (ZIP) [file pone.0158729.s003.zip › PEO-PBD FRAP Fast/P2904 on surface Free Run 1_C001T014.jpg]

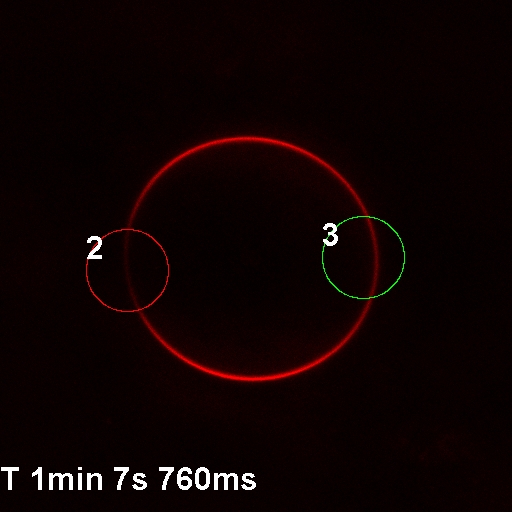

Supplement: S3 File — Zip file archive containing original photomicrographs obtained in Fluorescence Recovery After Photobleaching (FRAP) experiments. (ZIP) [file pone.0158729.s003.zip › PEO-PBD FRAP Fast/P2904 on surface Free Run 1_C001T015.jpg]

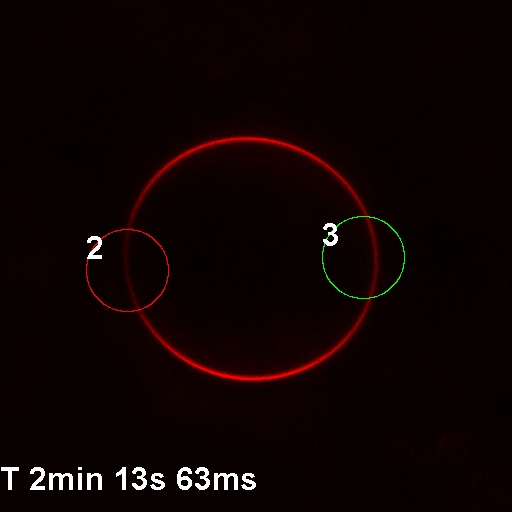

Supplement: S3 File — Zip file archive containing original photomicrographs obtained in Fluorescence Recovery After Photobleaching (FRAP) experiments. (ZIP) [file pone.0158729.s003.zip › PEO-PBD FRAP Fast/P2904 on surface Free Run 1_C001T016.jpg]

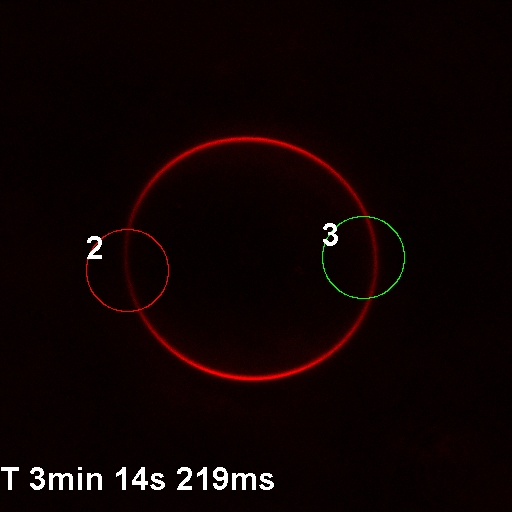

Supplement: S3 File — Zip file archive containing original photomicrographs obtained in Fluorescence Recovery After Photobleaching (FRAP) experiments. (ZIP) [file pone.0158729.s003.zip › PEO-PBD FRAP Fast/P2904 on surface Free Run 1_C001T017.jpg]

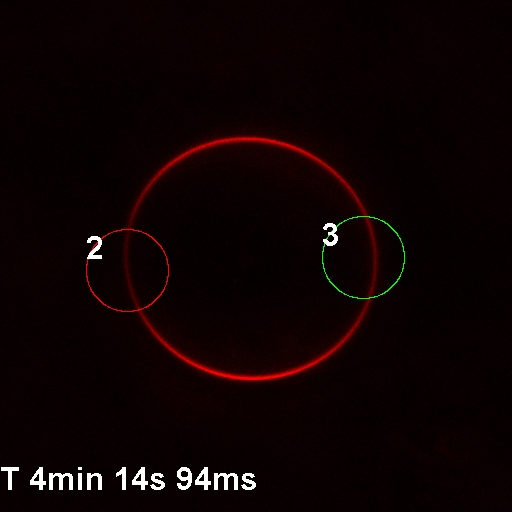

Supplement: S3 File — Zip file archive containing original photomicrographs obtained in Fluorescence Recovery After Photobleaching (FRAP) experiments. (ZIP) [file pone.0158729.s003.zip › PEO-PBD FRAP Fast/P2904 on surface Free Run 1_C001T018.jpg]

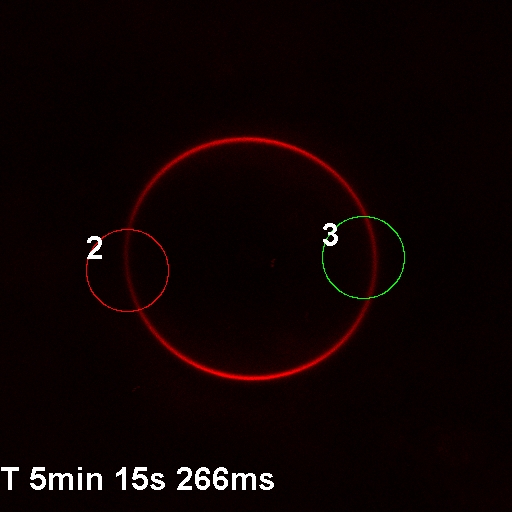

Supplement: S3 File — Zip file archive containing original photomicrographs obtained in Fluorescence Recovery After Photobleaching (FRAP) experiments. (ZIP) [file pone.0158729.s003.zip › PEO-PBD FRAP Fast/P2904 on surface Free Run 1_C001T019.jpg]

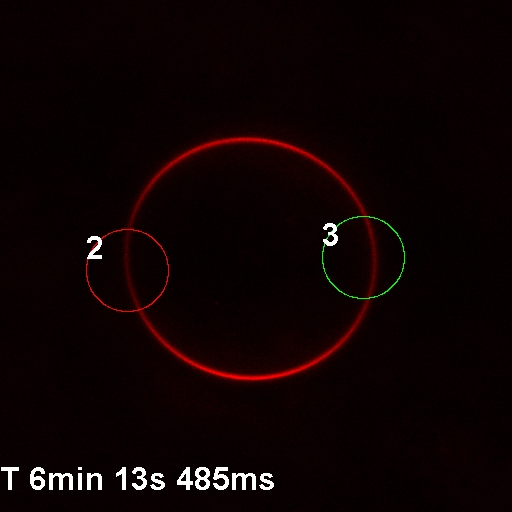

Supplement: S3 File — Zip file archive containing original photomicrographs obtained in Fluorescence Recovery After Photobleaching (FRAP) experiments. (ZIP) [file pone.0158729.s003.zip › PEO-PBD FRAP Fast/P2904 on surface Free Run 1_C001T020.jpg]

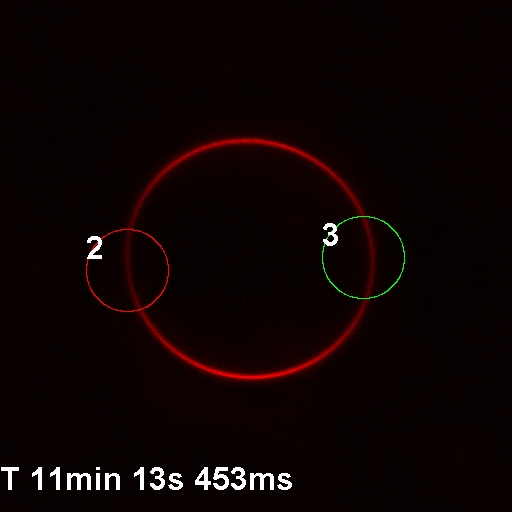

Supplement: S3 File — Zip file archive containing original photomicrographs obtained in Fluorescence Recovery After Photobleaching (FRAP) experiments. (ZIP) [file pone.0158729.s003.zip › PEO-PBD FRAP Fast/P2904 on surface Free Run 1_C001T021.jpg]

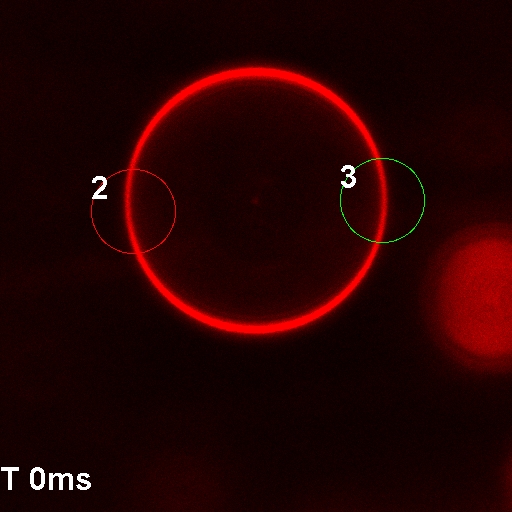

Supplement: S3 File — Zip file archive containing original photomicrographs obtained in Fluorescence Recovery After Photobleaching (FRAP) experiments. (ZIP) [file pone.0158729.s003.zip › PEO-PBD FRAP Slow/P2904 on surface_C001T001.jpg]

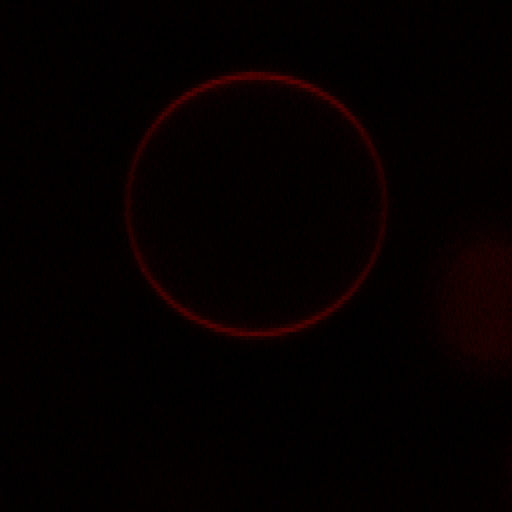

Supplement: S3 File — Zip file archive containing original photomicrographs obtained in Fluorescence Recovery After Photobleaching (FRAP) experiments. (ZIP) [file pone.0158729.s003.zip › PEO-PBD FRAP Slow/P2904 on surface_C001T001-R001.jpg]

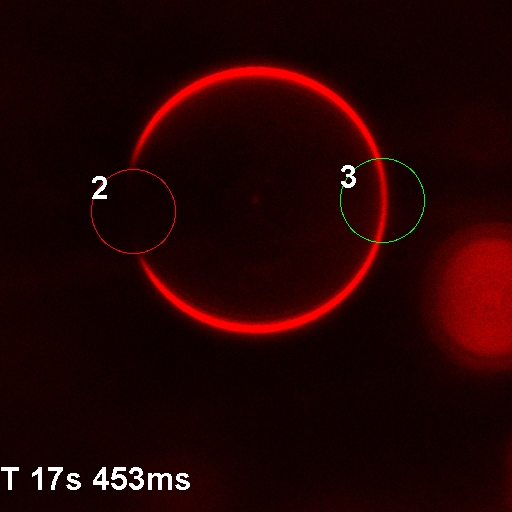

Supplement: S3 File — Zip file archive containing original photomicrographs obtained in Fluorescence Recovery After Photobleaching (FRAP) experiments. (ZIP) [file pone.0158729.s003.zip › PEO-PBD FRAP Slow/P2904 on surface_C001T002.jpg]

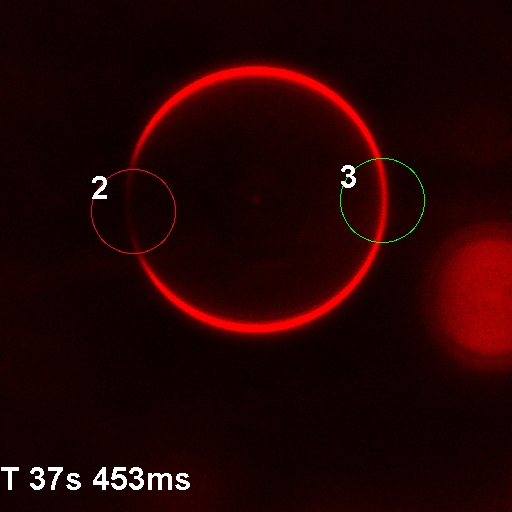

Supplement: S3 File — Zip file archive containing original photomicrographs obtained in Fluorescence Recovery After Photobleaching (FRAP) experiments. (ZIP) [file pone.0158729.s003.zip › PEO-PBD FRAP Slow/P2904 on surface_C001T003.jpg]

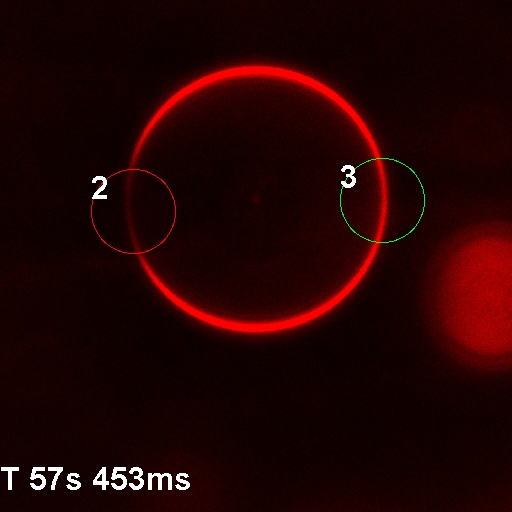

Supplement: S3 File — Zip file archive containing original photomicrographs obtained in Fluorescence Recovery After Photobleaching (FRAP) experiments. (ZIP) [file pone.0158729.s003.zip › PEO-PBD FRAP Slow/P2904 on surface_C001T004.jpg]

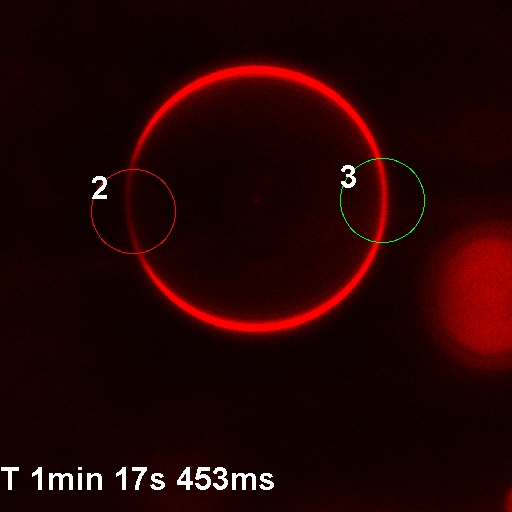

Supplement: S3 File — Zip file archive containing original photomicrographs obtained in Fluorescence Recovery After Photobleaching (FRAP) experiments. (ZIP) [file pone.0158729.s003.zip › PEO-PBD FRAP Slow/P2904 on surface_C001T005.jpg]

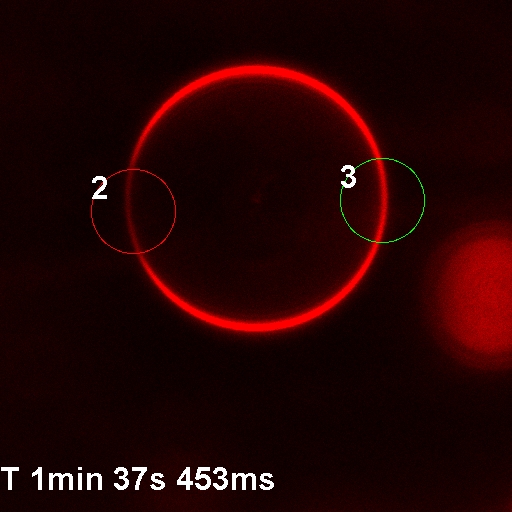

Supplement: S3 File — Zip file archive containing original photomicrographs obtained in Fluorescence Recovery After Photobleaching (FRAP) experiments. (ZIP) [file pone.0158729.s003.zip › PEO-PBD FRAP Slow/P2904 on surface_C001T006.jpg]

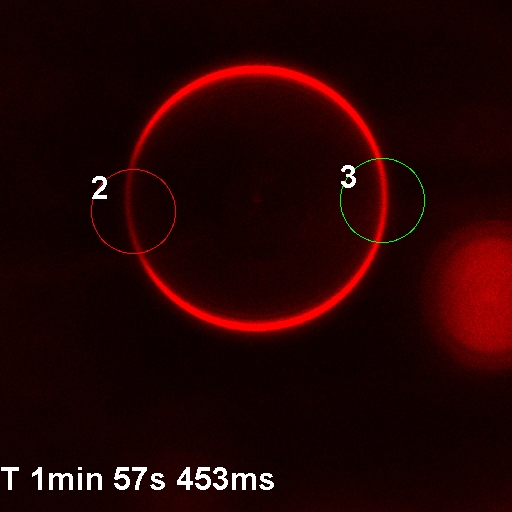

Supplement: S3 File — Zip file archive containing original photomicrographs obtained in Fluorescence Recovery After Photobleaching (FRAP) experiments. (ZIP) [file pone.0158729.s003.zip › PEO-PBD FRAP Slow/P2904 on surface_C001T007.jpg]

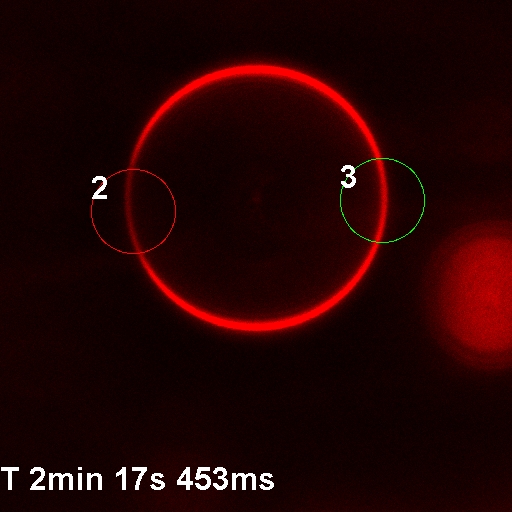

Supplement: S3 File — Zip file archive containing original photomicrographs obtained in Fluorescence Recovery After Photobleaching (FRAP) experiments. (ZIP) [file pone.0158729.s003.zip › PEO-PBD FRAP Slow/P2904 on surface_C001T008.jpg]

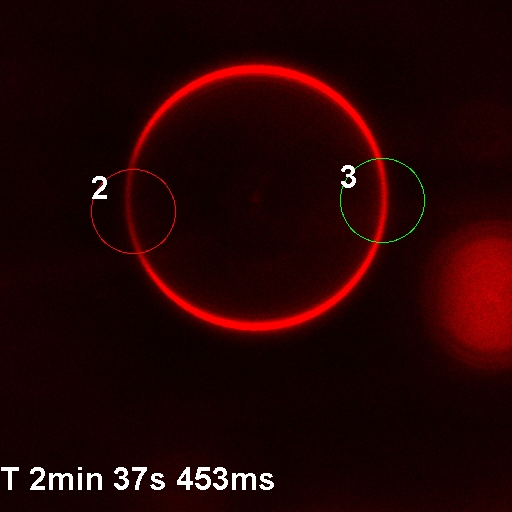

Supplement: S3 File — Zip file archive containing original photomicrographs obtained in Fluorescence Recovery After Photobleaching (FRAP) experiments. (ZIP) [file pone.0158729.s003.zip › PEO-PBD FRAP Slow/P2904 on surface_C001T009.jpg]

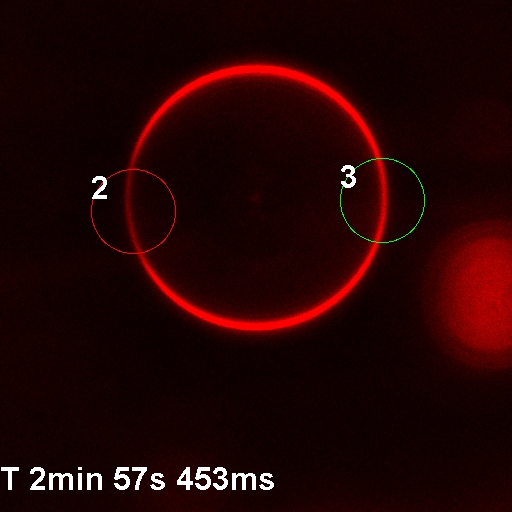

Supplement: S3 File — Zip file archive containing original photomicrographs obtained in Fluorescence Recovery After Photobleaching (FRAP) experiments. (ZIP) [file pone.0158729.s003.zip › PEO-PBD FRAP Slow/P2904 on surface_C001T010.jpg]

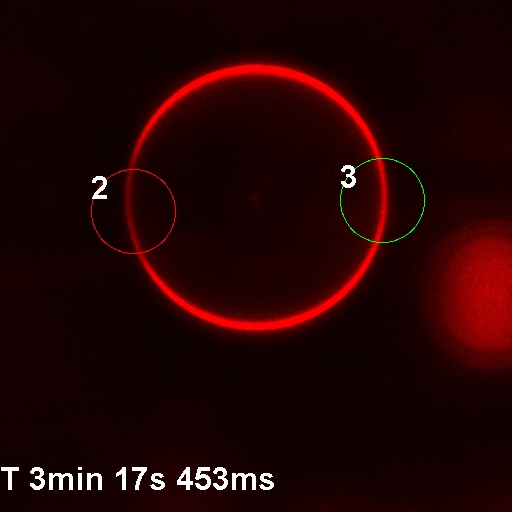

Supplement: S3 File — Zip file archive containing original photomicrographs obtained in Fluorescence Recovery After Photobleaching (FRAP) experiments. (ZIP) [file pone.0158729.s003.zip › PEO-PBD FRAP Slow/P2904 on surface_C001T011.jpg]

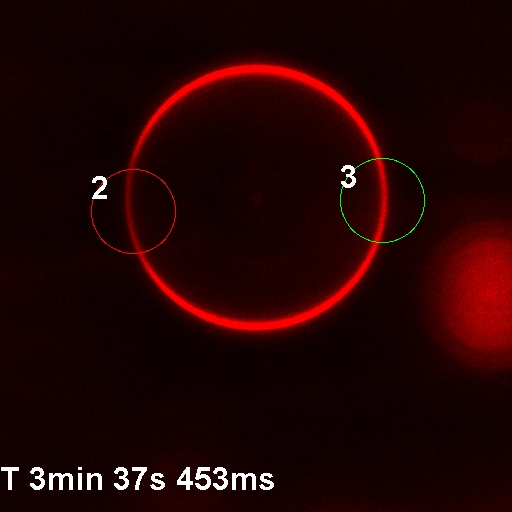

Supplement: S3 File — Zip file archive containing original photomicrographs obtained in Fluorescence Recovery After Photobleaching (FRAP) experiments. (ZIP) [file pone.0158729.s003.zip › PEO-PBD FRAP Slow/P2904 on surface_C001T012.jpg]

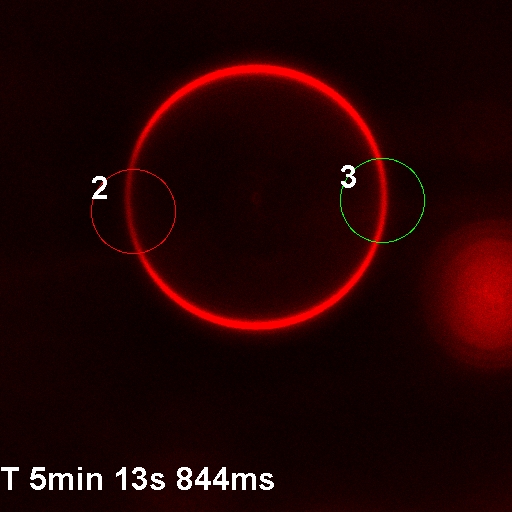

Supplement: S3 File — Zip file archive containing original photomicrographs obtained in Fluorescence Recovery After Photobleaching (FRAP) experiments. (ZIP) [file pone.0158729.s003.zip › PEO-PBD FRAP Slow/P2904 on surface_C001T013.jpg]

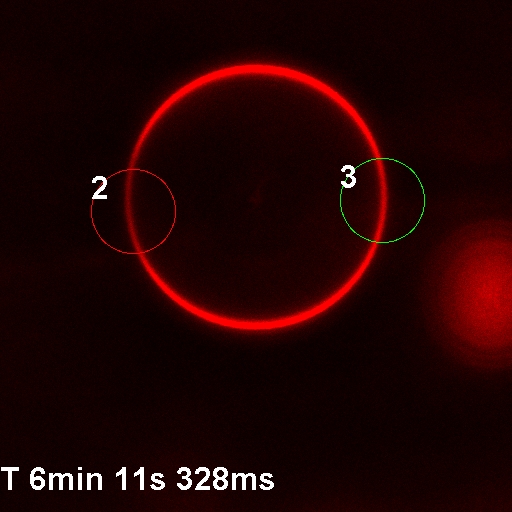

Supplement: S3 File — Zip file archive containing original photomicrographs obtained in Fluorescence Recovery After Photobleaching (FRAP) experiments. (ZIP) [file pone.0158729.s003.zip › PEO-PBD FRAP Slow/P2904 on surface_C001T014.jpg]

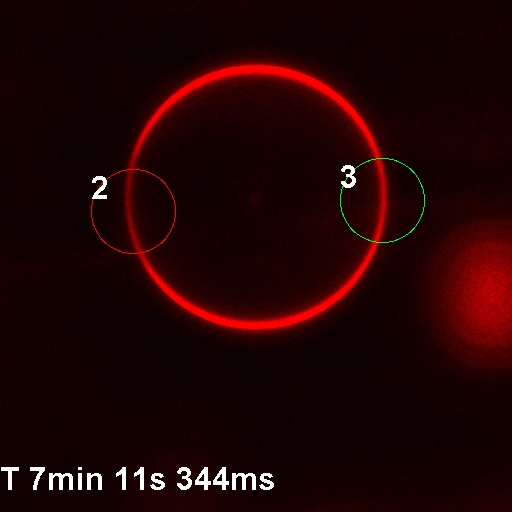

Supplement: S3 File — Zip file archive containing original photomicrographs obtained in Fluorescence Recovery After Photobleaching (FRAP) experiments. (ZIP) [file pone.0158729.s003.zip › PEO-PBD FRAP Slow/P2904 on surface_C001T015.jpg]

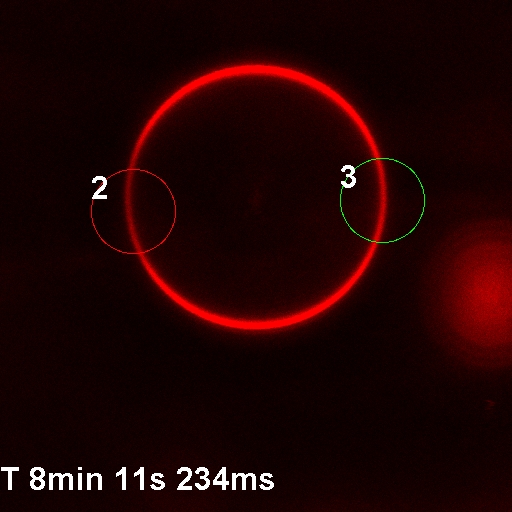

Supplement: S3 File — Zip file archive containing original photomicrographs obtained in Fluorescence Recovery After Photobleaching (FRAP) experiments. (ZIP) [file pone.0158729.s003.zip › PEO-PBD FRAP Slow/P2904 on surface_C001T016.jpg]

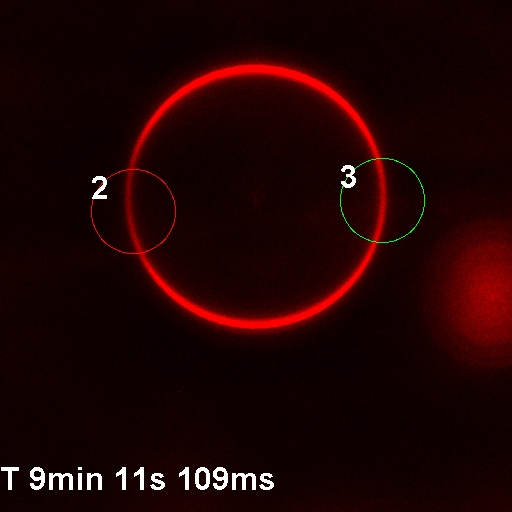

Supplement: S3 File — Zip file archive containing original photomicrographs obtained in Fluorescence Recovery After Photobleaching (FRAP) experiments. (ZIP) [file pone.0158729.s003.zip › PEO-PBD FRAP Slow/P2904 on surface_C001T017.jpg]

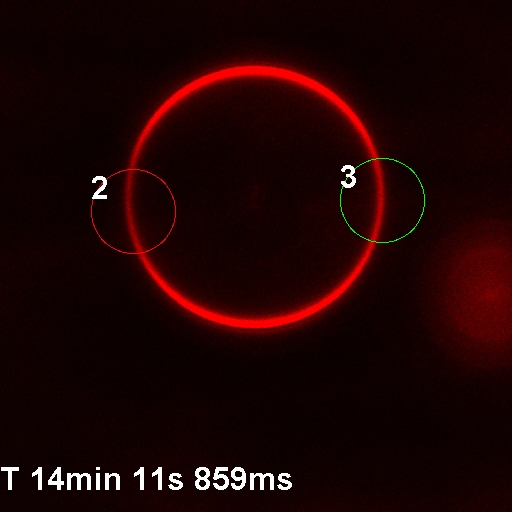

Supplement: S3 File — Zip file archive containing original photomicrographs obtained in Fluorescence Recovery After Photobleaching (FRAP) experiments. (ZIP) [file pone.0158729.s003.zip › PEO-PBD FRAP Slow/P2904 on surface_C001T018.jpg]

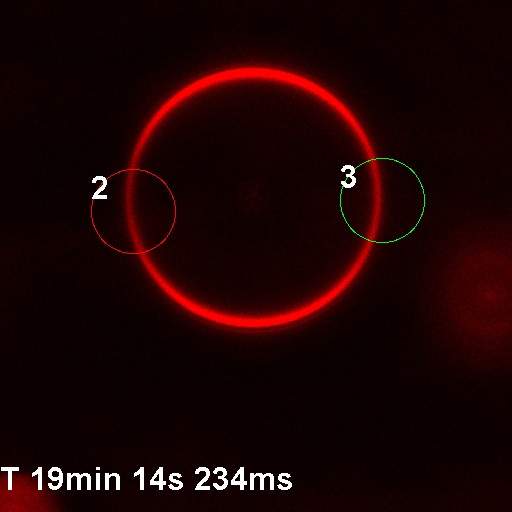

Supplement: S3 File — Zip file archive containing original photomicrographs obtained in Fluorescence Recovery After Photobleaching (FRAP) experiments. (ZIP) [file pone.0158729.s003.zip › PEO-PBD FRAP Slow/P2904 on surface_C001T019.jpg]

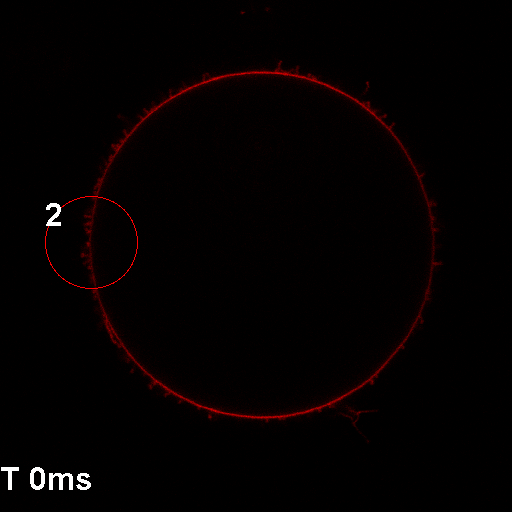

Supplement: S3 File — Zip file archive containing original photomicrographs obtained in Fluorescence Recovery After Photobleaching (FRAP) experiments. (ZIP) [file pone.0158729.s003.zip › PEO-PBD-COO- FRAP Fast/COO- frap with Freerun 15 times_C001T001.tif]

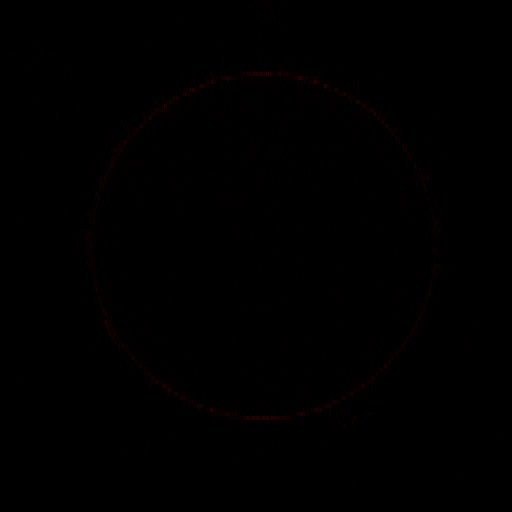

Supplement: S3 File — Zip file archive containing original photomicrographs obtained in Fluorescence Recovery After Photobleaching (FRAP) experiments. (ZIP) [file pone.0158729.s003.zip › PEO-PBD-COO- FRAP Fast/COO- frap with Freerun 15 times_C001T001-R001.tif]

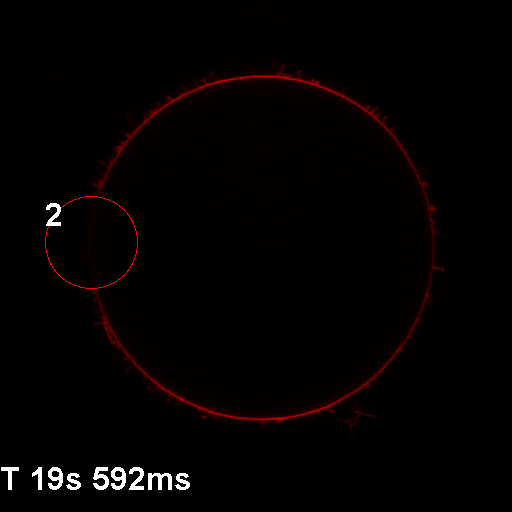

Supplement: S3 File — Zip file archive containing original photomicrographs obtained in Fluorescence Recovery After Photobleaching (FRAP) experiments. (ZIP) [file pone.0158729.s003.zip › PEO-PBD-COO- FRAP Fast/COO- frap with Freerun 15 times_C001T002.tif]

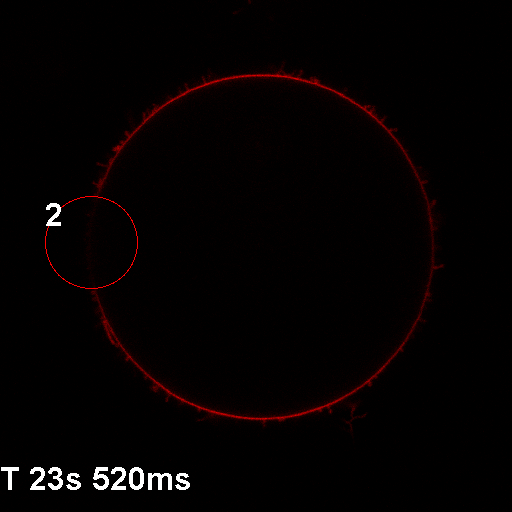

Supplement: S3 File — Zip file archive containing original photomicrographs obtained in Fluorescence Recovery After Photobleaching (FRAP) experiments. (ZIP) [file pone.0158729.s003.zip › PEO-PBD-COO- FRAP Fast/COO- frap with Freerun 15 times_C001T003.tif]

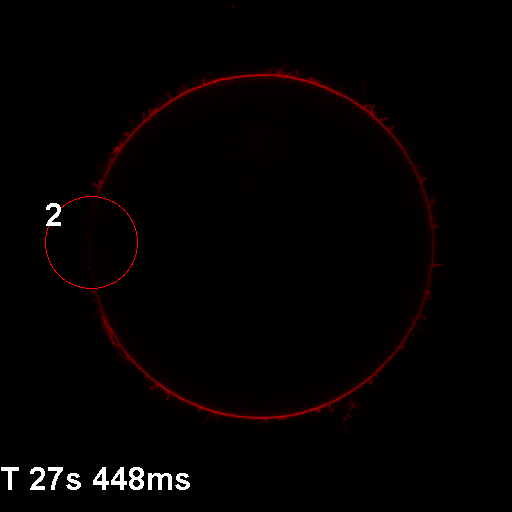

Supplement: S3 File — Zip file archive containing original photomicrographs obtained in Fluorescence Recovery After Photobleaching (FRAP) experiments. (ZIP) [file pone.0158729.s003.zip › PEO-PBD-COO- FRAP Fast/COO- frap with Freerun 15 times_C001T004.tif]

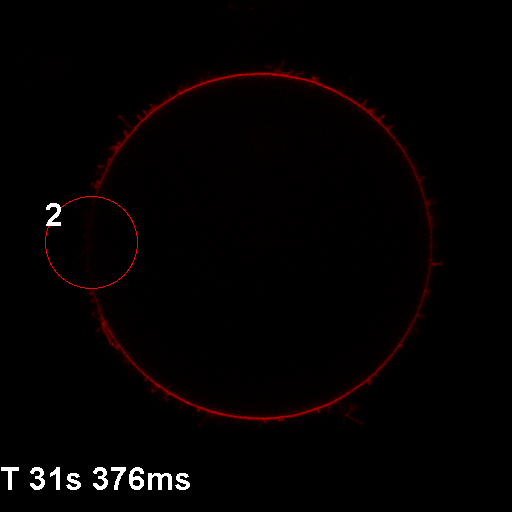

Supplement: S3 File — Zip file archive containing original photomicrographs obtained in Fluorescence Recovery After Photobleaching (FRAP) experiments. (ZIP) [file pone.0158729.s003.zip › PEO-PBD-COO- FRAP Fast/COO- frap with Freerun 15 times_C001T005.tif]

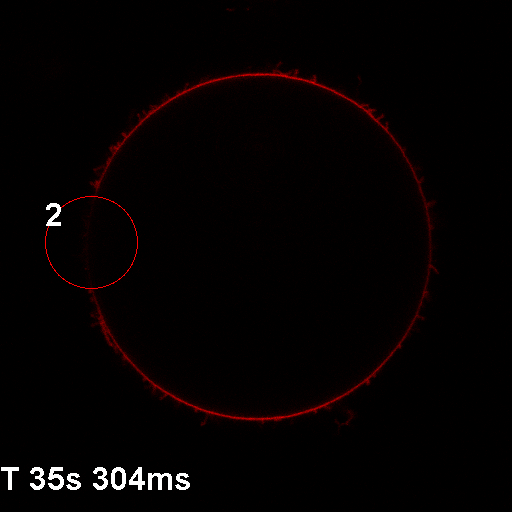

Supplement: S3 File — Zip file archive containing original photomicrographs obtained in Fluorescence Recovery After Photobleaching (FRAP) experiments. (ZIP) [file pone.0158729.s003.zip › PEO-PBD-COO- FRAP Fast/COO- frap with Freerun 15 times_C001T006.tif]

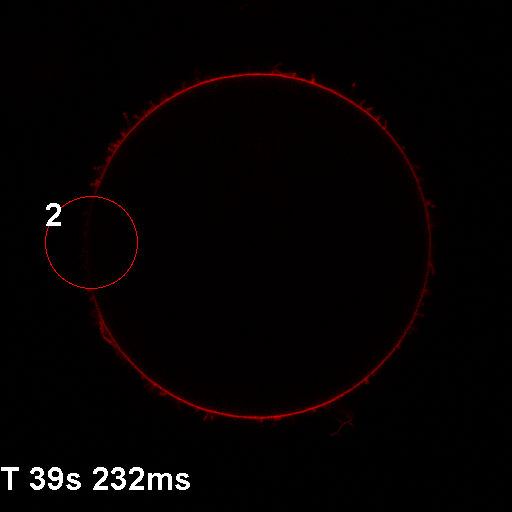

Supplement: S3 File — Zip file archive containing original photomicrographs obtained in Fluorescence Recovery After Photobleaching (FRAP) experiments. (ZIP) [file pone.0158729.s003.zip › PEO-PBD-COO- FRAP Fast/COO- frap with Freerun 15 times_C001T007.tif]

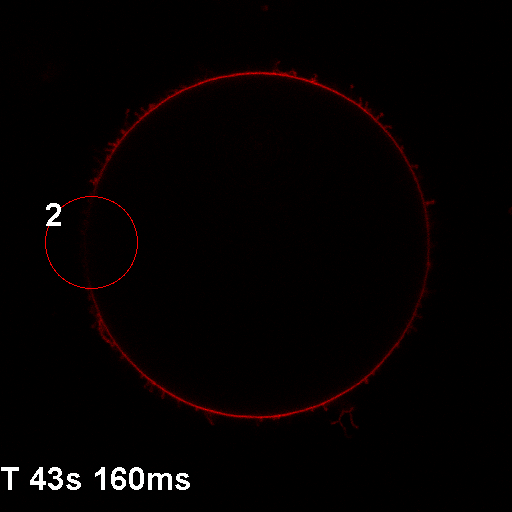

Supplement: S3 File — Zip file archive containing original photomicrographs obtained in Fluorescence Recovery After Photobleaching (FRAP) experiments. (ZIP) [file pone.0158729.s003.zip › PEO-PBD-COO- FRAP Fast/COO- frap with Freerun 15 times_C001T008.tif]

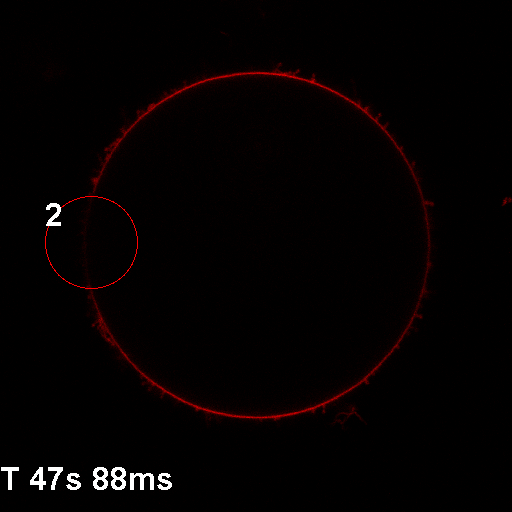

Supplement: S3 File — Zip file archive containing original photomicrographs obtained in Fluorescence Recovery After Photobleaching (FRAP) experiments. (ZIP) [file pone.0158729.s003.zip › PEO-PBD-COO- FRAP Fast/COO- frap with Freerun 15 times_C001T009.tif]

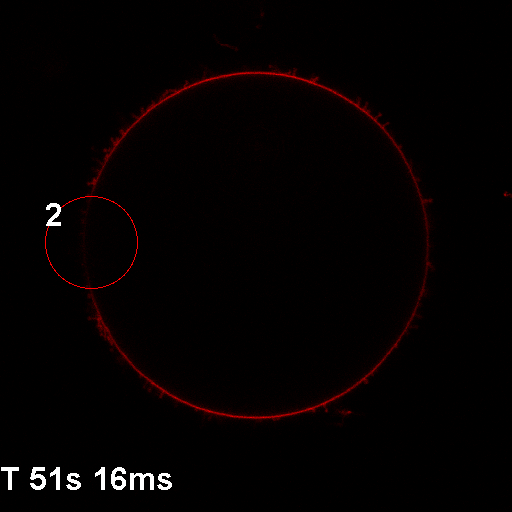

Supplement: S3 File — Zip file archive containing original photomicrographs obtained in Fluorescence Recovery After Photobleaching (FRAP) experiments. (ZIP) [file pone.0158729.s003.zip › PEO-PBD-COO- FRAP Fast/COO- frap with Freerun 15 times_C001T010.tif]

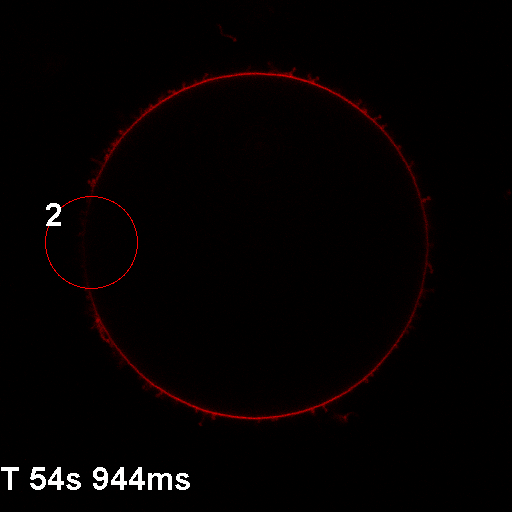

Supplement: S3 File — Zip file archive containing original photomicrographs obtained in Fluorescence Recovery After Photobleaching (FRAP) experiments. (ZIP) [file pone.0158729.s003.zip › PEO-PBD-COO- FRAP Fast/COO- frap with Freerun 15 times_C001T011.tif]

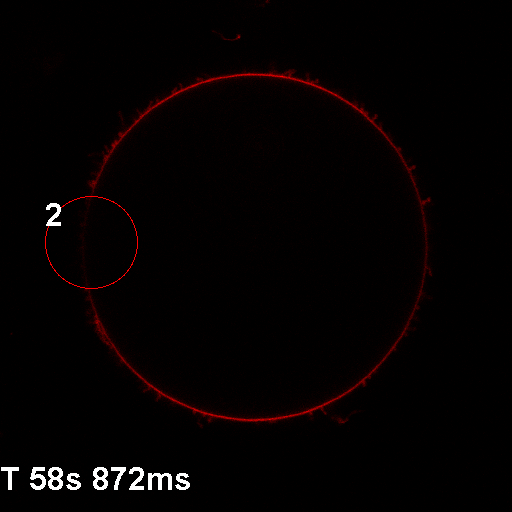

Supplement: S3 File — Zip file archive containing original photomicrographs obtained in Fluorescence Recovery After Photobleaching (FRAP) experiments. (ZIP) [file pone.0158729.s003.zip › PEO-PBD-COO- FRAP Fast/COO- frap with Freerun 15 times_C001T012.tif]

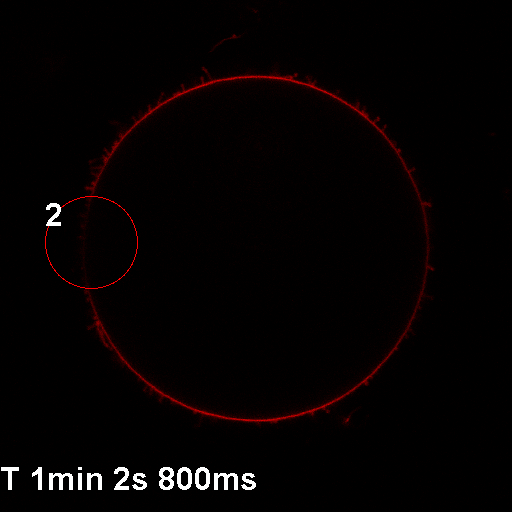

Supplement: S3 File — Zip file archive containing original photomicrographs obtained in Fluorescence Recovery After Photobleaching (FRAP) experiments. (ZIP) [file pone.0158729.s003.zip › PEO-PBD-COO- FRAP Fast/COO- frap with Freerun 15 times_C001T013.tif]

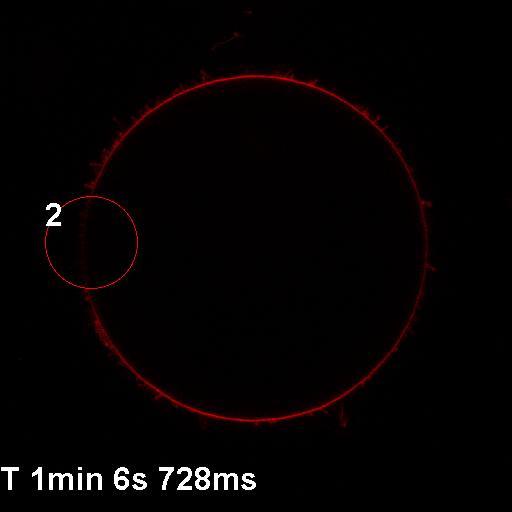

Supplement: S3 File — Zip file archive containing original photomicrographs obtained in Fluorescence Recovery After Photobleaching (FRAP) experiments. (ZIP) [file pone.0158729.s003.zip › PEO-PBD-COO- FRAP Fast/COO- frap with Freerun 15 times_C001T014.tif]

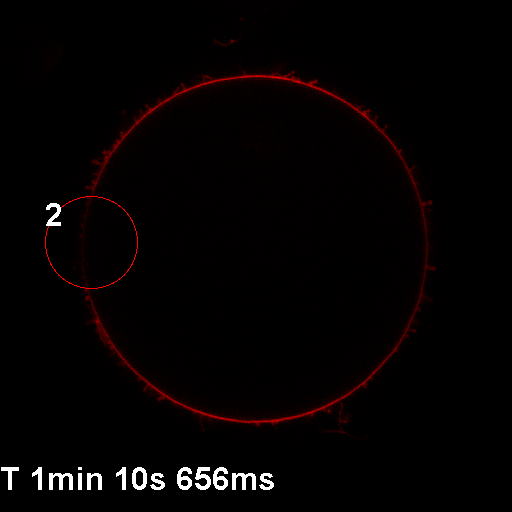

Supplement: S3 File — Zip file archive containing original photomicrographs obtained in Fluorescence Recovery After Photobleaching (FRAP) experiments. (ZIP) [file pone.0158729.s003.zip › PEO-PBD-COO- FRAP Fast/COO- frap with Freerun 15 times_C001T015.tif]

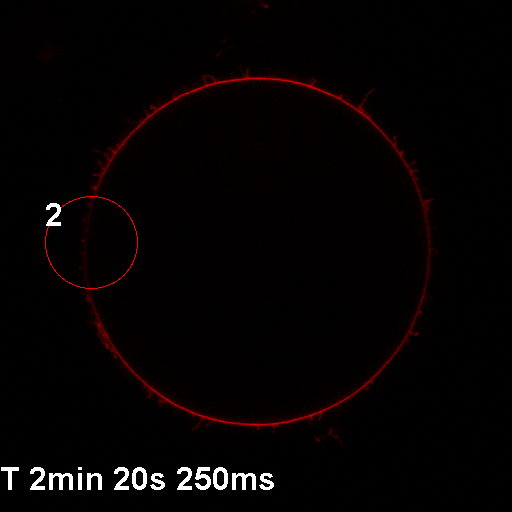

Supplement: S3 File — Zip file archive containing original photomicrographs obtained in Fluorescence Recovery After Photobleaching (FRAP) experiments. (ZIP) [file pone.0158729.s003.zip › PEO-PBD-COO- FRAP Fast/COO- frap with Freerun 15 times_C001T016.tif]

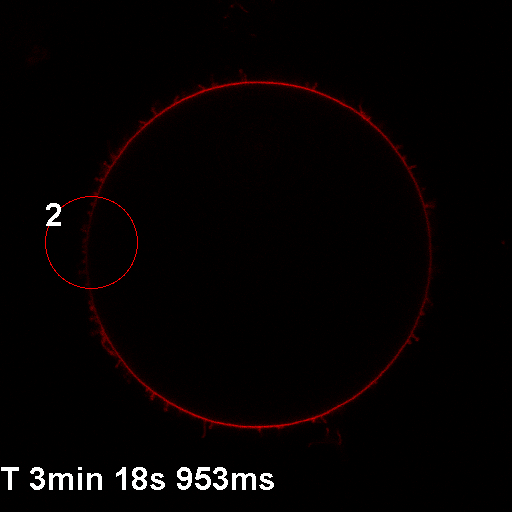

Supplement: S3 File — Zip file archive containing original photomicrographs obtained in Fluorescence Recovery After Photobleaching (FRAP) experiments. (ZIP) [file pone.0158729.s003.zip › PEO-PBD-COO- FRAP Fast/COO- frap with Freerun 15 times_C001T017.tif]

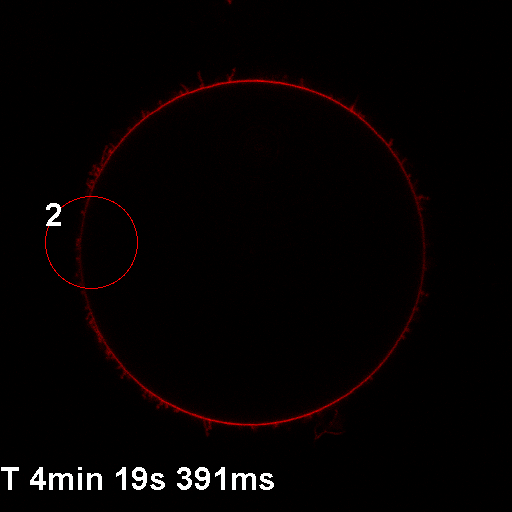

Supplement: S3 File — Zip file archive containing original photomicrographs obtained in Fluorescence Recovery After Photobleaching (FRAP) experiments. (ZIP) [file pone.0158729.s003.zip › PEO-PBD-COO- FRAP Fast/COO- frap with Freerun 15 times_C001T018.tif]

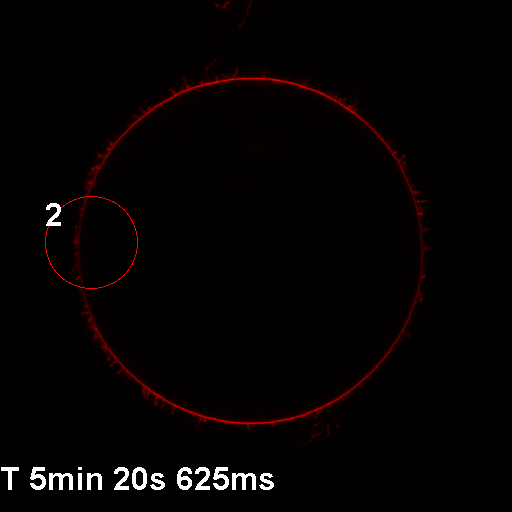

Supplement: S3 File — Zip file archive containing original photomicrographs obtained in Fluorescence Recovery After Photobleaching (FRAP) experiments. (ZIP) [file pone.0158729.s003.zip › PEO-PBD-COO- FRAP Fast/COO- frap with Freerun 15 times_C001T019.tif]

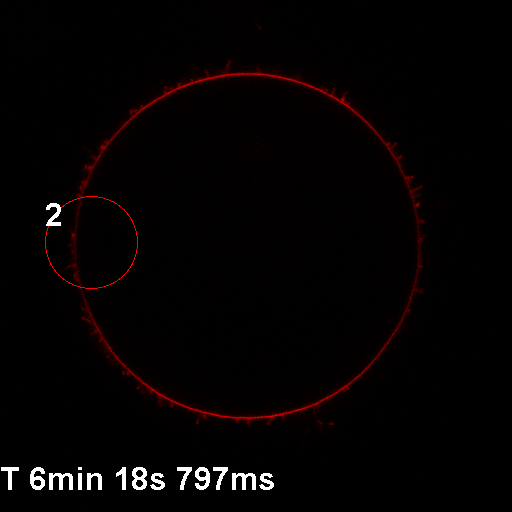

Supplement: S3 File — Zip file archive containing original photomicrographs obtained in Fluorescence Recovery After Photobleaching (FRAP) experiments. (ZIP) [file pone.0158729.s003.zip › PEO-PBD-COO- FRAP Fast/COO- frap with Freerun 15 times_C001T020.tif]

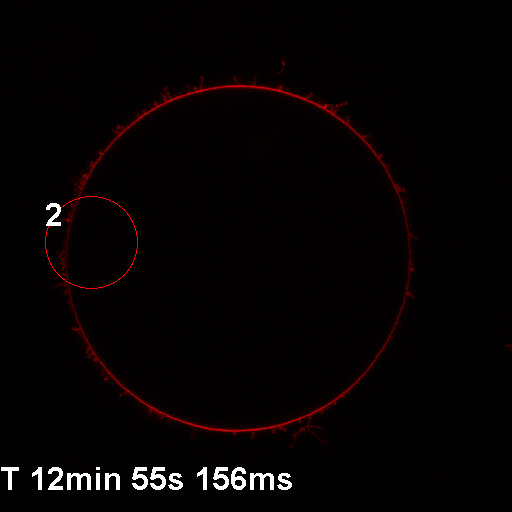

Supplement: S3 File — Zip file archive containing original photomicrographs obtained in Fluorescence Recovery After Photobleaching (FRAP) experiments. (ZIP) [file pone.0158729.s003.zip › PEO-PBD-COO- FRAP Fast/COO- frap with Freerun 15 times_C001T021.tif]

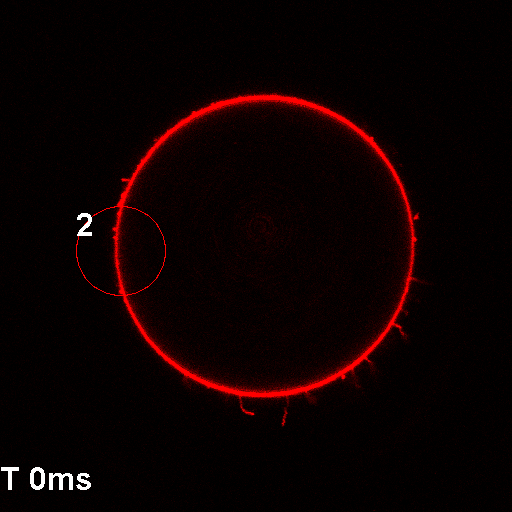

Supplement: S3 File — Zip file archive containing original photomicrographs obtained in Fluorescence Recovery After Photobleaching (FRAP) experiments. (ZIP) [file pone.0158729.s003.zip › PEO-PBD-COO- FRAP Slow/COO- frap normal 20 min img002_C001T001.tif]

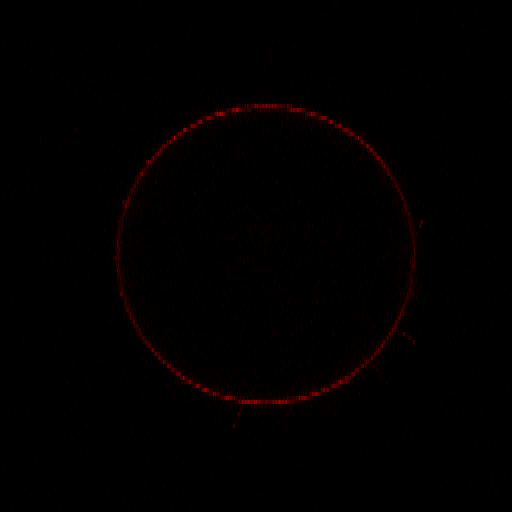

Supplement: S3 File — Zip file archive containing original photomicrographs obtained in Fluorescence Recovery After Photobleaching (FRAP) experiments. (ZIP) [file pone.0158729.s003.zip › PEO-PBD-COO- FRAP Slow/COO- frap normal 20 min img002_C001T001-R001.tif]

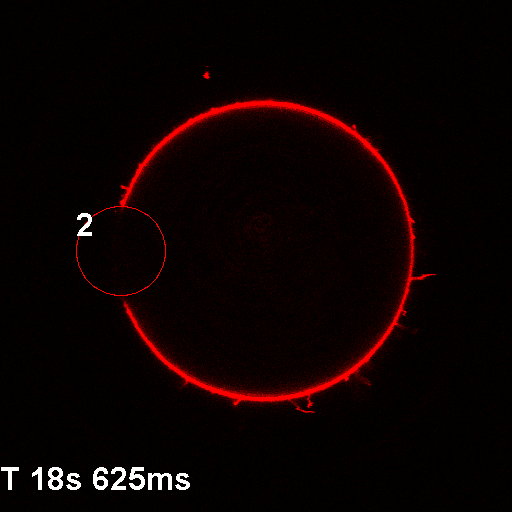

Supplement: S3 File — Zip file archive containing original photomicrographs obtained in Fluorescence Recovery After Photobleaching (FRAP) experiments. (ZIP) [file pone.0158729.s003.zip › PEO-PBD-COO- FRAP Slow/COO- frap normal 20 min img002_C001T002.tif]

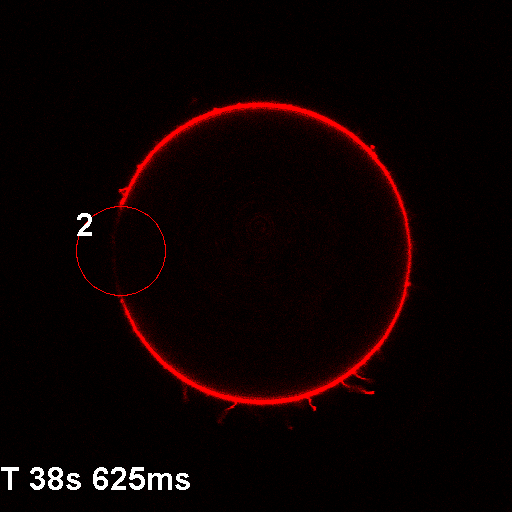

Supplement: S3 File — Zip file archive containing original photomicrographs obtained in Fluorescence Recovery After Photobleaching (FRAP) experiments. (ZIP) [file pone.0158729.s003.zip › PEO-PBD-COO- FRAP Slow/COO- frap normal 20 min img002_C001T003.tif]

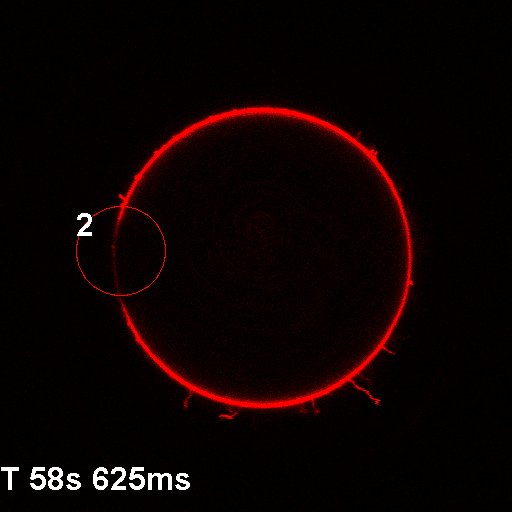

Supplement: S3 File — Zip file archive containing original photomicrographs obtained in Fluorescence Recovery After Photobleaching (FRAP) experiments. (ZIP) [file pone.0158729.s003.zip › PEO-PBD-COO- FRAP Slow/COO- frap normal 20 min img002_C001T004.tif]

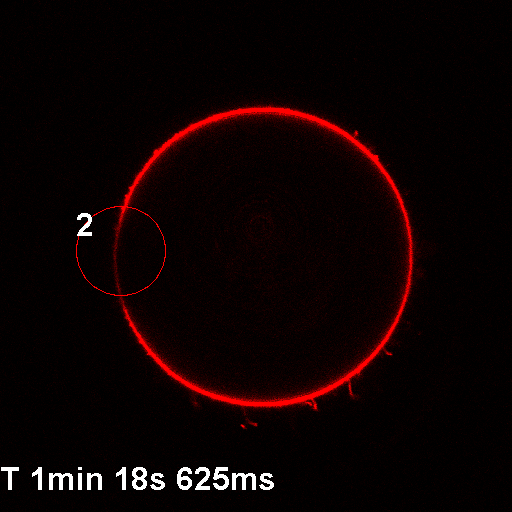

Supplement: S3 File — Zip file archive containing original photomicrographs obtained in Fluorescence Recovery After Photobleaching (FRAP) experiments. (ZIP) [file pone.0158729.s003.zip › PEO-PBD-COO- FRAP Slow/COO- frap normal 20 min img002_C001T005.tif]

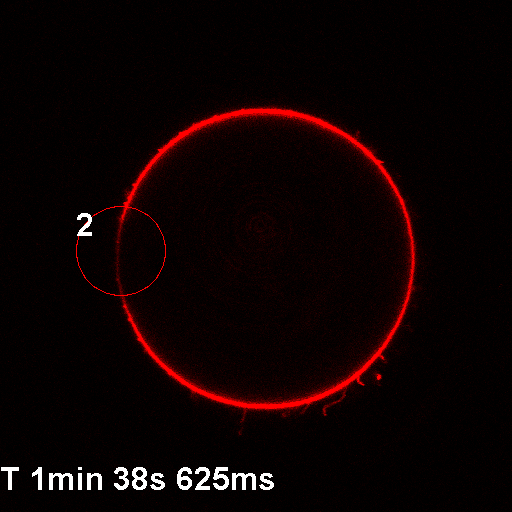

Supplement: S3 File — Zip file archive containing original photomicrographs obtained in Fluorescence Recovery After Photobleaching (FRAP) experiments. (ZIP) [file pone.0158729.s003.zip › PEO-PBD-COO- FRAP Slow/COO- frap normal 20 min img002_C001T006.tif]

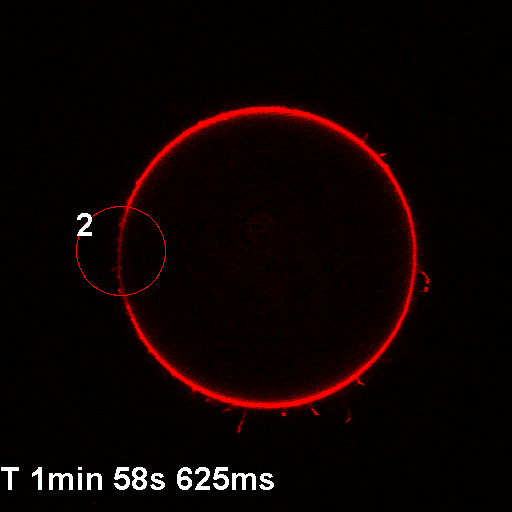

Supplement: S3 File — Zip file archive containing original photomicrographs obtained in Fluorescence Recovery After Photobleaching (FRAP) experiments. (ZIP) [file pone.0158729.s003.zip › PEO-PBD-COO- FRAP Slow/COO- frap normal 20 min img002_C001T007.tif]

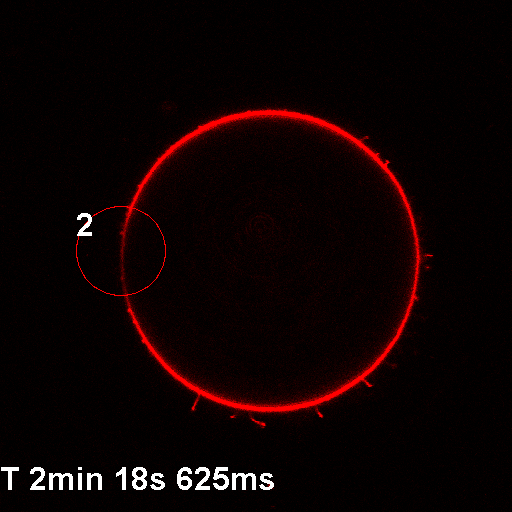

Supplement: S3 File — Zip file archive containing original photomicrographs obtained in Fluorescence Recovery After Photobleaching (FRAP) experiments. (ZIP) [file pone.0158729.s003.zip › PEO-PBD-COO- FRAP Slow/COO- frap normal 20 min img002_C001T008.tif]

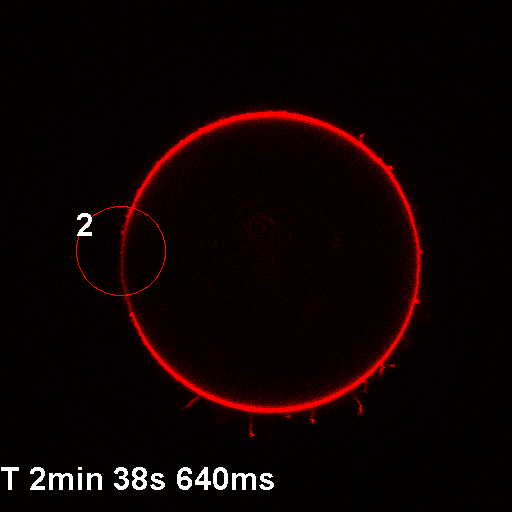

Supplement: S3 File — Zip file archive containing original photomicrographs obtained in Fluorescence Recovery After Photobleaching (FRAP) experiments. (ZIP) [file pone.0158729.s003.zip › PEO-PBD-COO- FRAP Slow/COO- frap normal 20 min img002_C001T009.tif]

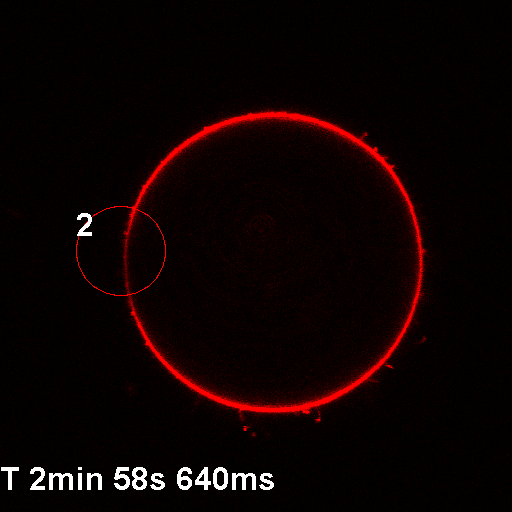

Supplement: S3 File — Zip file archive containing original photomicrographs obtained in Fluorescence Recovery After Photobleaching (FRAP) experiments. (ZIP) [file pone.0158729.s003.zip › PEO-PBD-COO- FRAP Slow/COO- frap normal 20 min img002_C001T010.tif]

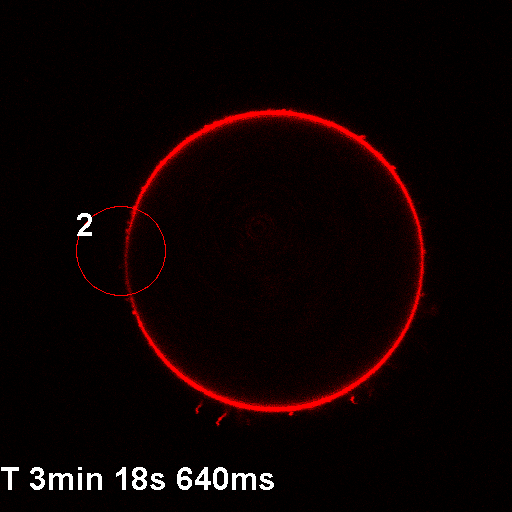

Supplement: S3 File — Zip file archive containing original photomicrographs obtained in Fluorescence Recovery After Photobleaching (FRAP) experiments. (ZIP) [file pone.0158729.s003.zip › PEO-PBD-COO- FRAP Slow/COO- frap normal 20 min img002_C001T011.tif]

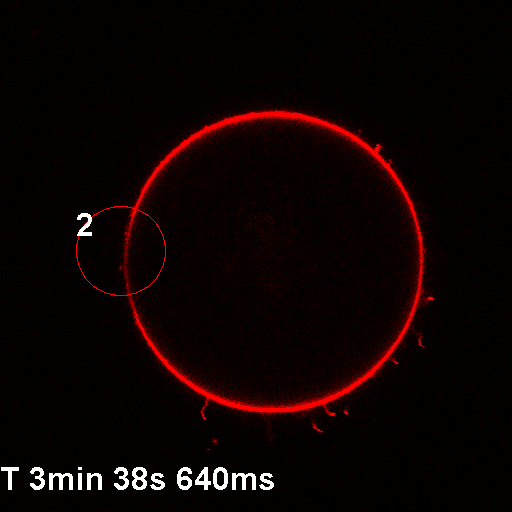

Supplement: S3 File — Zip file archive containing original photomicrographs obtained in Fluorescence Recovery After Photobleaching (FRAP) experiments. (ZIP) [file pone.0158729.s003.zip › PEO-PBD-COO- FRAP Slow/COO- frap normal 20 min img002_C001T012.tif]

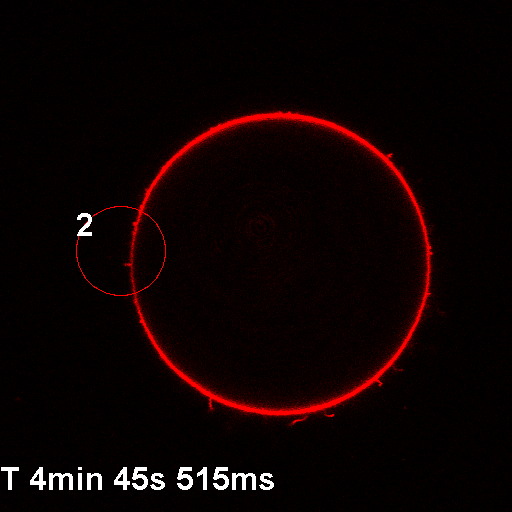

Supplement: S3 File — Zip file archive containing original photomicrographs obtained in Fluorescence Recovery After Photobleaching (FRAP) experiments. (ZIP) [file pone.0158729.s003.zip › PEO-PBD-COO- FRAP Slow/COO- frap normal 20 min img002_C001T013.tif]

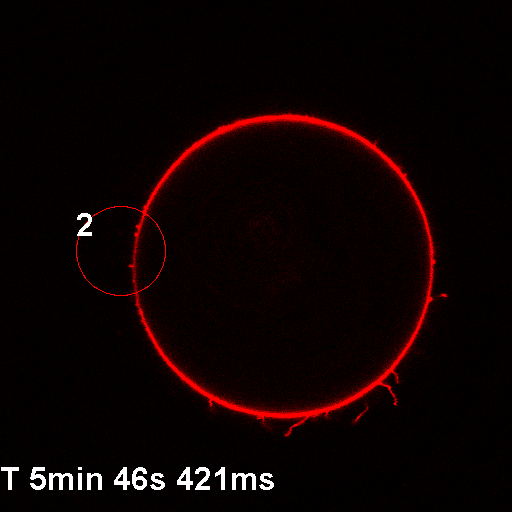

Supplement: S3 File — Zip file archive containing original photomicrographs obtained in Fluorescence Recovery After Photobleaching (FRAP) experiments. (ZIP) [file pone.0158729.s003.zip › PEO-PBD-COO- FRAP Slow/COO- frap normal 20 min img002_C001T014.tif]

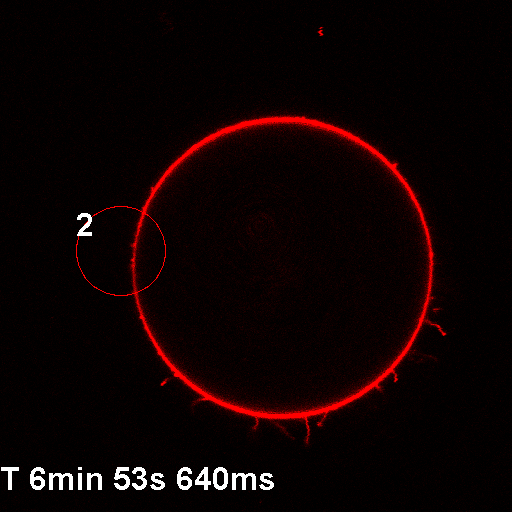

Supplement: S3 File — Zip file archive containing original photomicrographs obtained in Fluorescence Recovery After Photobleaching (FRAP) experiments. (ZIP) [file pone.0158729.s003.zip › PEO-PBD-COO- FRAP Slow/COO- frap normal 20 min img002_C001T015.tif]

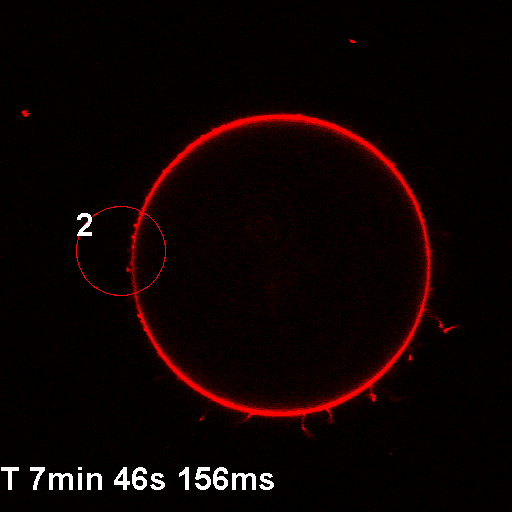

Supplement: S3 File — Zip file archive containing original photomicrographs obtained in Fluorescence Recovery After Photobleaching (FRAP) experiments. (ZIP) [file pone.0158729.s003.zip › PEO-PBD-COO- FRAP Slow/COO- frap normal 20 min img002_C001T016.tif]

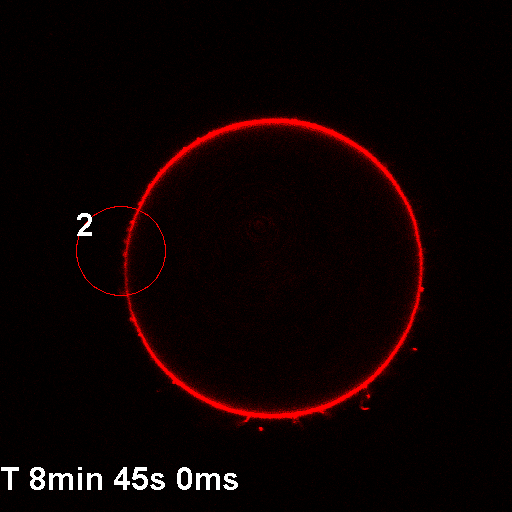

Supplement: S3 File — Zip file archive containing original photomicrographs obtained in Fluorescence Recovery After Photobleaching (FRAP) experiments. (ZIP) [file pone.0158729.s003.zip › PEO-PBD-COO- FRAP Slow/COO- frap normal 20 min img002_C001T017.tif]

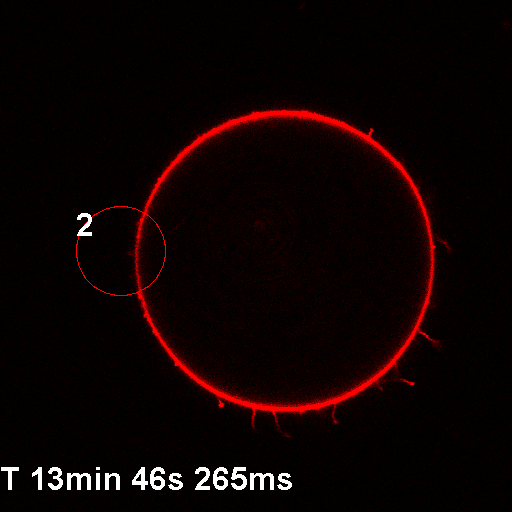

Supplement: S3 File — Zip file archive containing original photomicrographs obtained in Fluorescence Recovery After Photobleaching (FRAP) experiments. (ZIP) [file pone.0158729.s003.zip › PEO-PBD-COO- FRAP Slow/COO- frap normal 20 min img002_C001T018.tif]

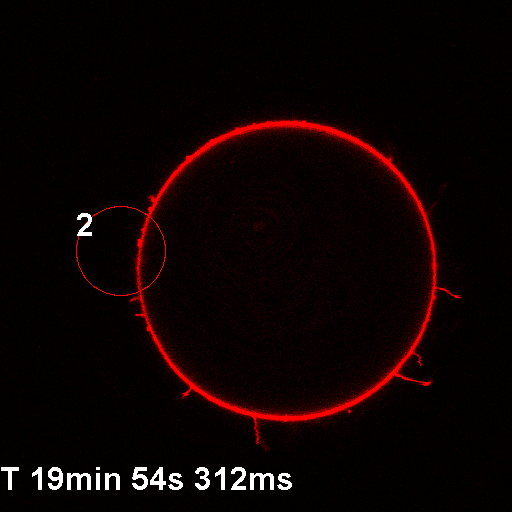

Supplement: S3 File — Zip file archive containing original photomicrographs obtained in Fluorescence Recovery After Photobleaching (FRAP) experiments. (ZIP) [file pone.0158729.s003.zip › PEO-PBD-COO- FRAP Slow/COO- frap normal 20 min img002_C001T019.tif]

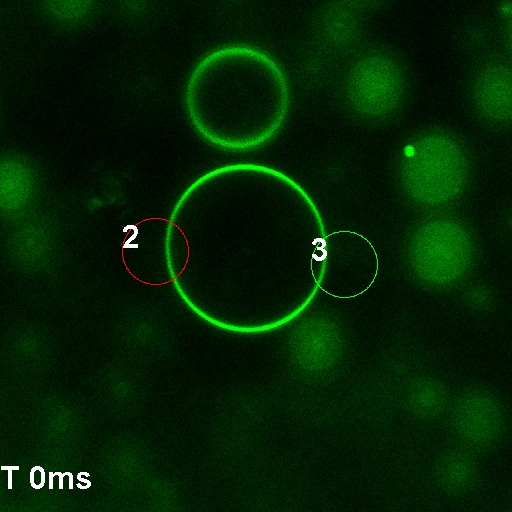

Supplement: S3 File — Zip file archive containing original photomicrographs obtained in Fluorescence Recovery After Photobleaching (FRAP) experiments. (ZIP) [file pone.0158729.s003.zip › PEO-PBD-NH2+ FRAP Fast/NH2 On Surface Free Run 15 activation 10frame 20us_pix_C001T001.jpg]

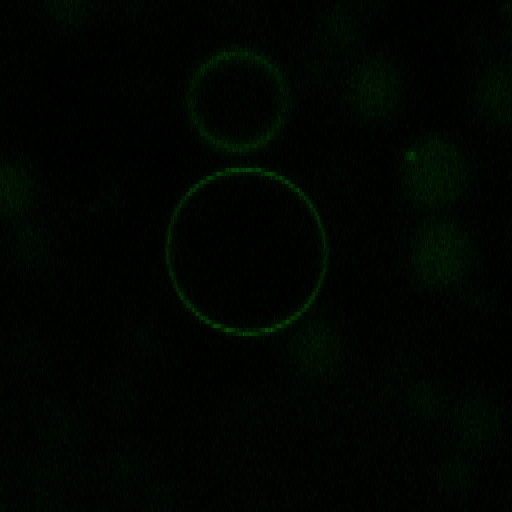

Supplement: S3 File — Zip file archive containing original photomicrographs obtained in Fluorescence Recovery After Photobleaching (FRAP) experiments. (ZIP) [file pone.0158729.s003.zip › PEO-PBD-NH2+ FRAP Fast/NH2 On Surface Free Run 15 activation 10frame 20us_pix_C001T001-R001.jpg]

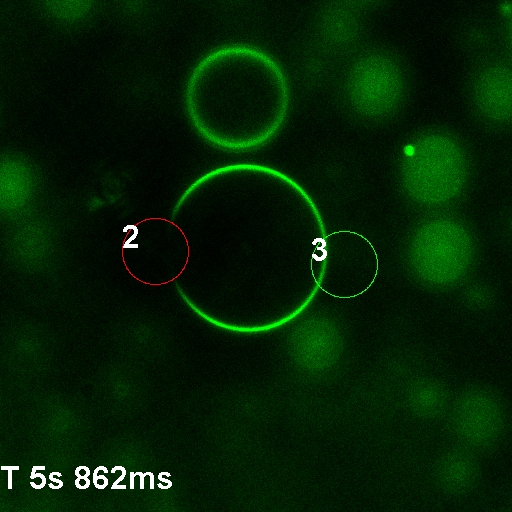

Supplement: S3 File — Zip file archive containing original photomicrographs obtained in Fluorescence Recovery After Photobleaching (FRAP) experiments. (ZIP) [file pone.0158729.s003.zip › PEO-PBD-NH2+ FRAP Fast/NH2 On Surface Free Run 15 activation 10frame 20us_pix_C001T002.jpg]

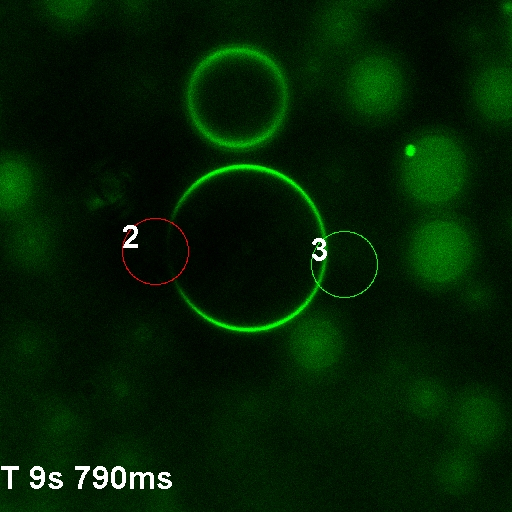

Supplement: S3 File — Zip file archive containing original photomicrographs obtained in Fluorescence Recovery After Photobleaching (FRAP) experiments. (ZIP) [file pone.0158729.s003.zip › PEO-PBD-NH2+ FRAP Fast/NH2 On Surface Free Run 15 activation 10frame 20us_pix_C001T003.jpg]

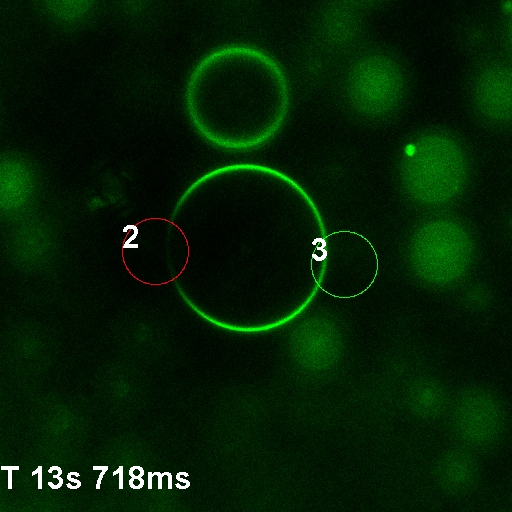

Supplement: S3 File — Zip file archive containing original photomicrographs obtained in Fluorescence Recovery After Photobleaching (FRAP) experiments. (ZIP) [file pone.0158729.s003.zip › PEO-PBD-NH2+ FRAP Fast/NH2 On Surface Free Run 15 activation 10frame 20us_pix_C001T004.jpg]

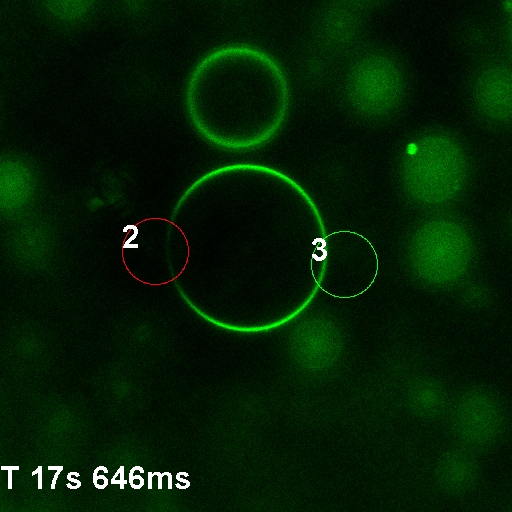

Supplement: S3 File — Zip file archive containing original photomicrographs obtained in Fluorescence Recovery After Photobleaching (FRAP) experiments. (ZIP) [file pone.0158729.s003.zip › PEO-PBD-NH2+ FRAP Fast/NH2 On Surface Free Run 15 activation 10frame 20us_pix_C001T005.jpg]

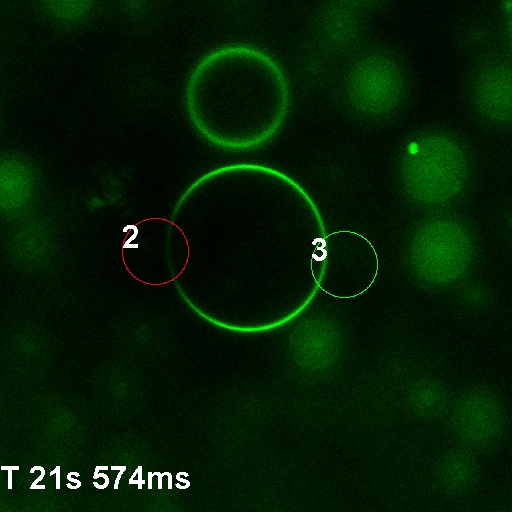

Supplement: S3 File — Zip file archive containing original photomicrographs obtained in Fluorescence Recovery After Photobleaching (FRAP) experiments. (ZIP) [file pone.0158729.s003.zip › PEO-PBD-NH2+ FRAP Fast/NH2 On Surface Free Run 15 activation 10frame 20us_pix_C001T006.jpg]

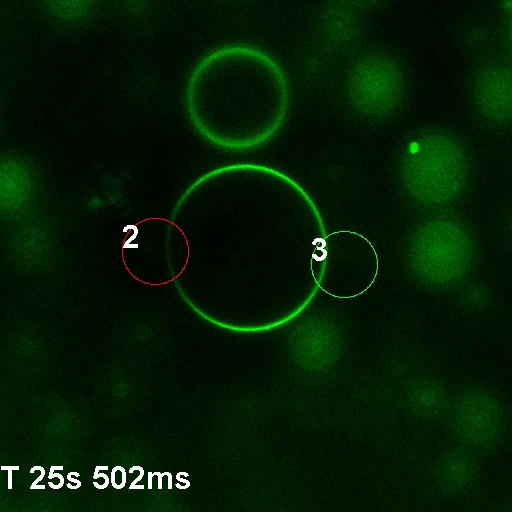

Supplement: S3 File — Zip file archive containing original photomicrographs obtained in Fluorescence Recovery After Photobleaching (FRAP) experiments. (ZIP) [file pone.0158729.s003.zip › PEO-PBD-NH2+ FRAP Fast/NH2 On Surface Free Run 15 activation 10frame 20us_pix_C001T007.jpg]

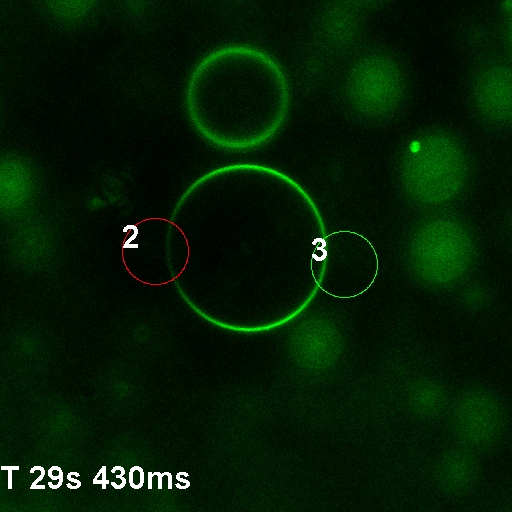

Supplement: S3 File — Zip file archive containing original photomicrographs obtained in Fluorescence Recovery After Photobleaching (FRAP) experiments. (ZIP) [file pone.0158729.s003.zip › PEO-PBD-NH2+ FRAP Fast/NH2 On Surface Free Run 15 activation 10frame 20us_pix_C001T008.jpg]

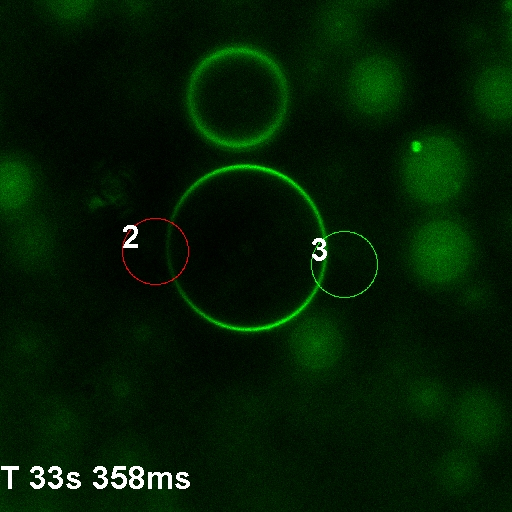

Supplement: S3 File — Zip file archive containing original photomicrographs obtained in Fluorescence Recovery After Photobleaching (FRAP) experiments. (ZIP) [file pone.0158729.s003.zip › PEO-PBD-NH2+ FRAP Fast/NH2 On Surface Free Run 15 activation 10frame 20us_pix_C001T009.jpg]

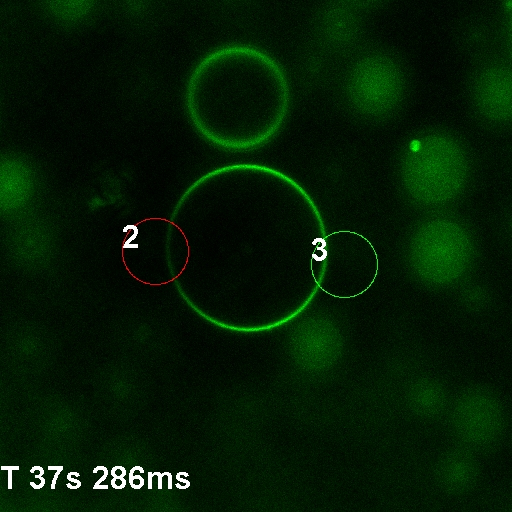

Supplement: S3 File — Zip file archive containing original photomicrographs obtained in Fluorescence Recovery After Photobleaching (FRAP) experiments. (ZIP) [file pone.0158729.s003.zip › PEO-PBD-NH2+ FRAP Fast/NH2 On Surface Free Run 15 activation 10frame 20us_pix_C001T010.jpg]

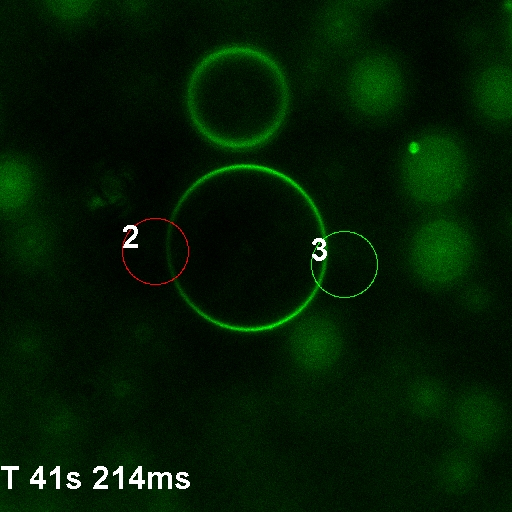

Supplement: S3 File — Zip file archive containing original photomicrographs obtained in Fluorescence Recovery After Photobleaching (FRAP) experiments. (ZIP) [file pone.0158729.s003.zip › PEO-PBD-NH2+ FRAP Fast/NH2 On Surface Free Run 15 activation 10frame 20us_pix_C001T011.jpg]

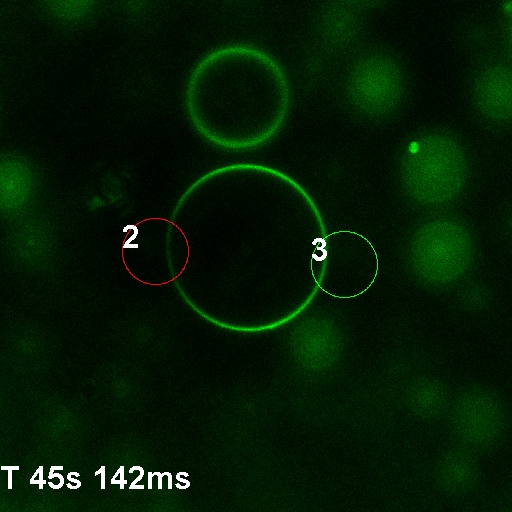

Supplement: S3 File — Zip file archive containing original photomicrographs obtained in Fluorescence Recovery After Photobleaching (FRAP) experiments. (ZIP) [file pone.0158729.s003.zip › PEO-PBD-NH2+ FRAP Fast/NH2 On Surface Free Run 15 activation 10frame 20us_pix_C001T012.jpg]

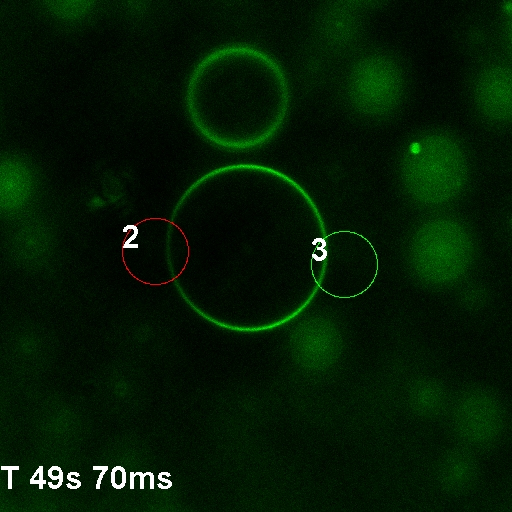

Supplement: S3 File — Zip file archive containing original photomicrographs obtained in Fluorescence Recovery After Photobleaching (FRAP) experiments. (ZIP) [file pone.0158729.s003.zip › PEO-PBD-NH2+ FRAP Fast/NH2 On Surface Free Run 15 activation 10frame 20us_pix_C001T013.jpg]

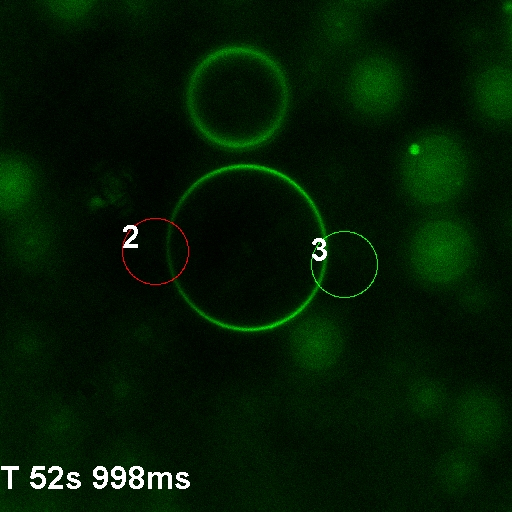

Supplement: S3 File — Zip file archive containing original photomicrographs obtained in Fluorescence Recovery After Photobleaching (FRAP) experiments. (ZIP) [file pone.0158729.s003.zip › PEO-PBD-NH2+ FRAP Fast/NH2 On Surface Free Run 15 activation 10frame 20us_pix_C001T014.jpg]

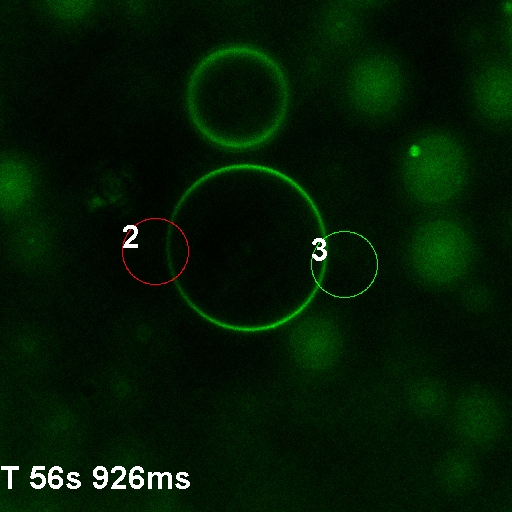

Supplement: S3 File — Zip file archive containing original photomicrographs obtained in Fluorescence Recovery After Photobleaching (FRAP) experiments. (ZIP) [file pone.0158729.s003.zip › PEO-PBD-NH2+ FRAP Fast/NH2 On Surface Free Run 15 activation 10frame 20us_pix_C001T015.jpg]
