# Supplementary figures and images for: The Role of Membrane Fluidization in the Gel-Assisted Formation of Giant Polymersomes (part 3 of 3)
Source: PLoS One. 2016 Jul 13;11(7):e0158729. doi: 10.1371/journal.pone.0158729 (PMC4943728; doi:10.1371/journal.pone.0158729)

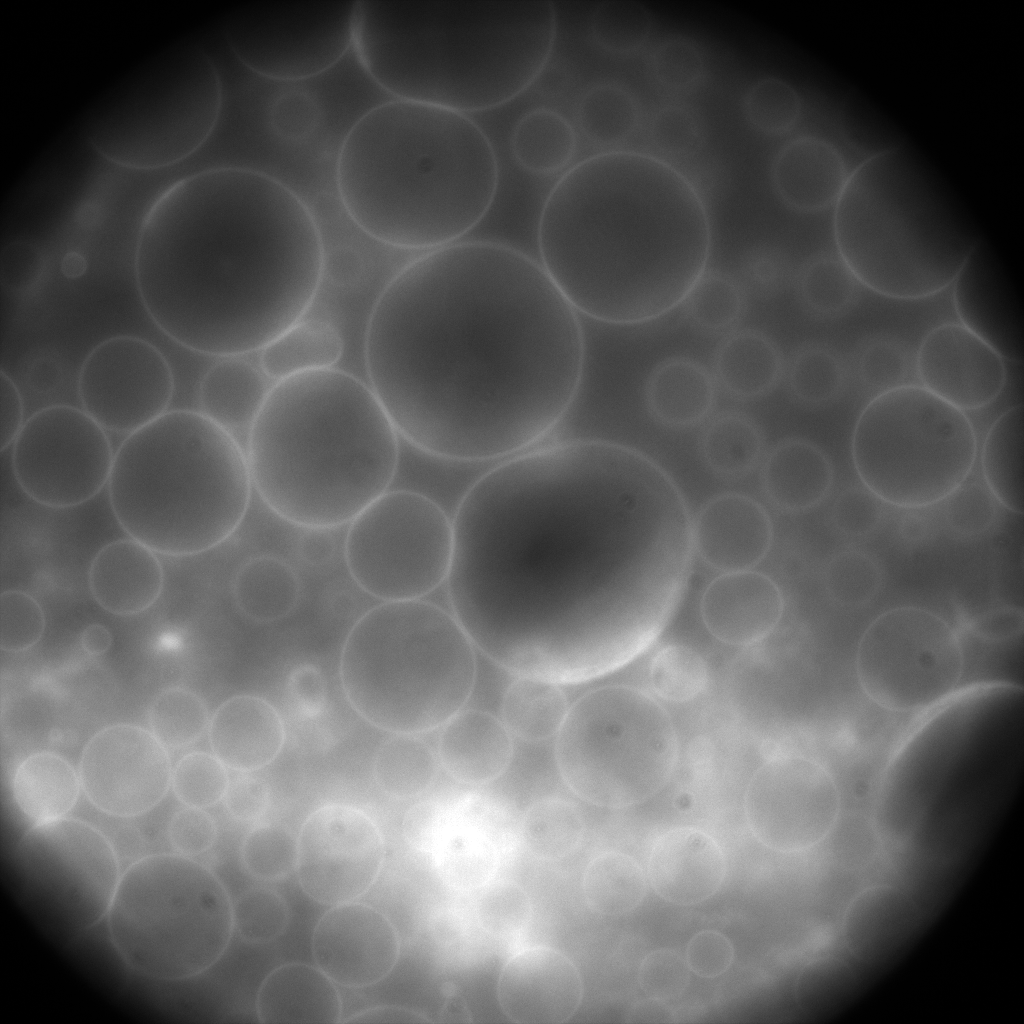

Supplement: S11 File — Zip file archive containing original photomicrographs of polymersomes formed following rehydration at 60°C. (ZIP) [file pone.0158729.s011.zip › 60C/Image_4098_20150311_174332.tif]

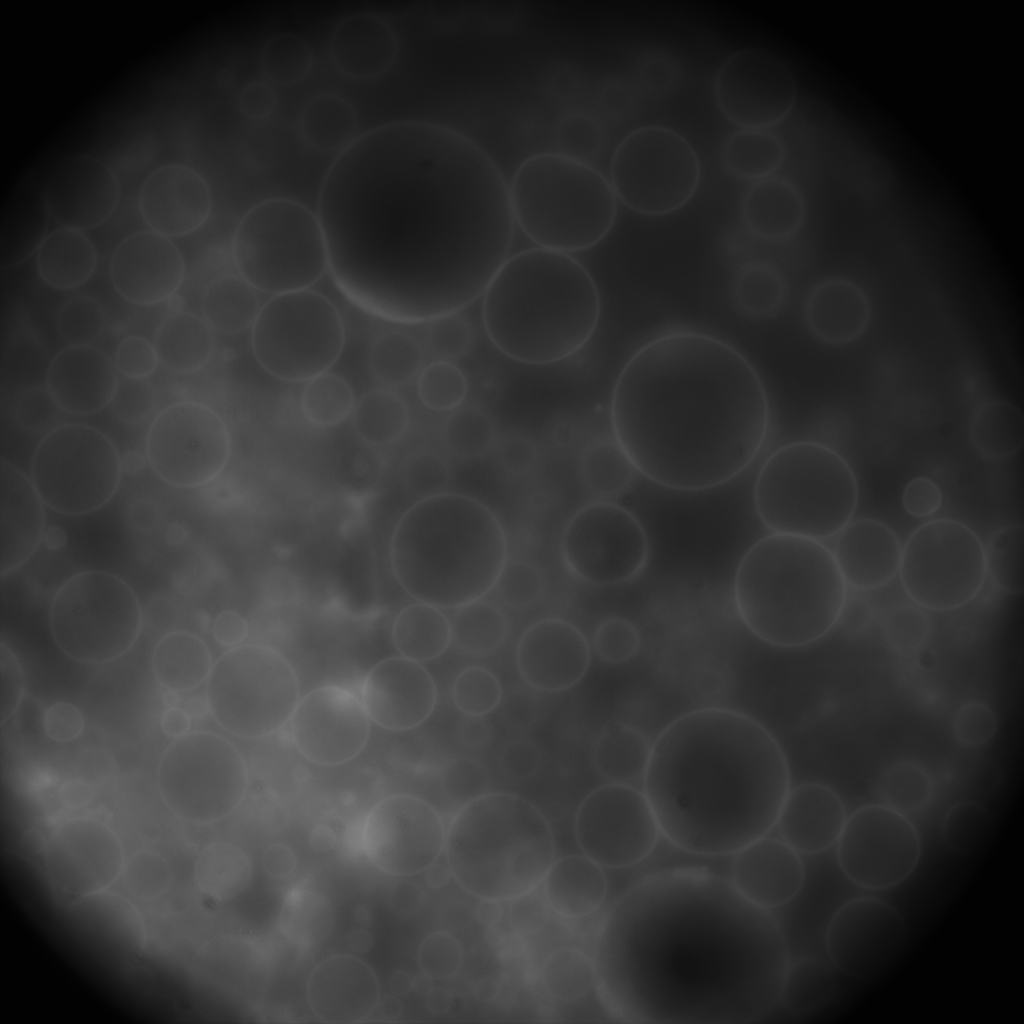

Supplement: S11 File — Zip file archive containing original photomicrographs of polymersomes formed following rehydration at 60°C. (ZIP) [file pone.0158729.s011.zip › 60C/Image_4099_20150311_174452.tif]

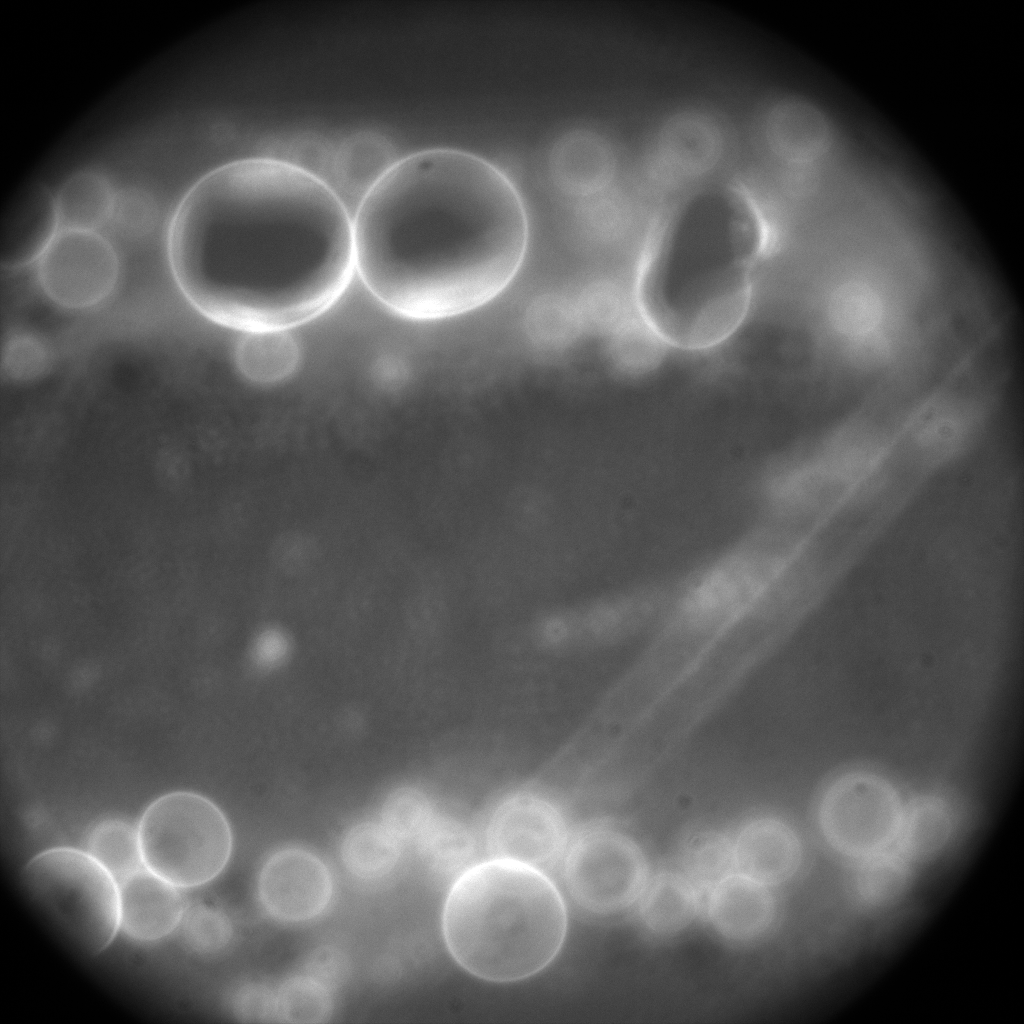

Supplement: S12 File — Zip file archive containing original photomicrographs of polymersomes formed following rehydration at 70°C. (ZIP) [file pone.0158729.s012.zip › 70C/Image_4101_20150311_180531.tif]

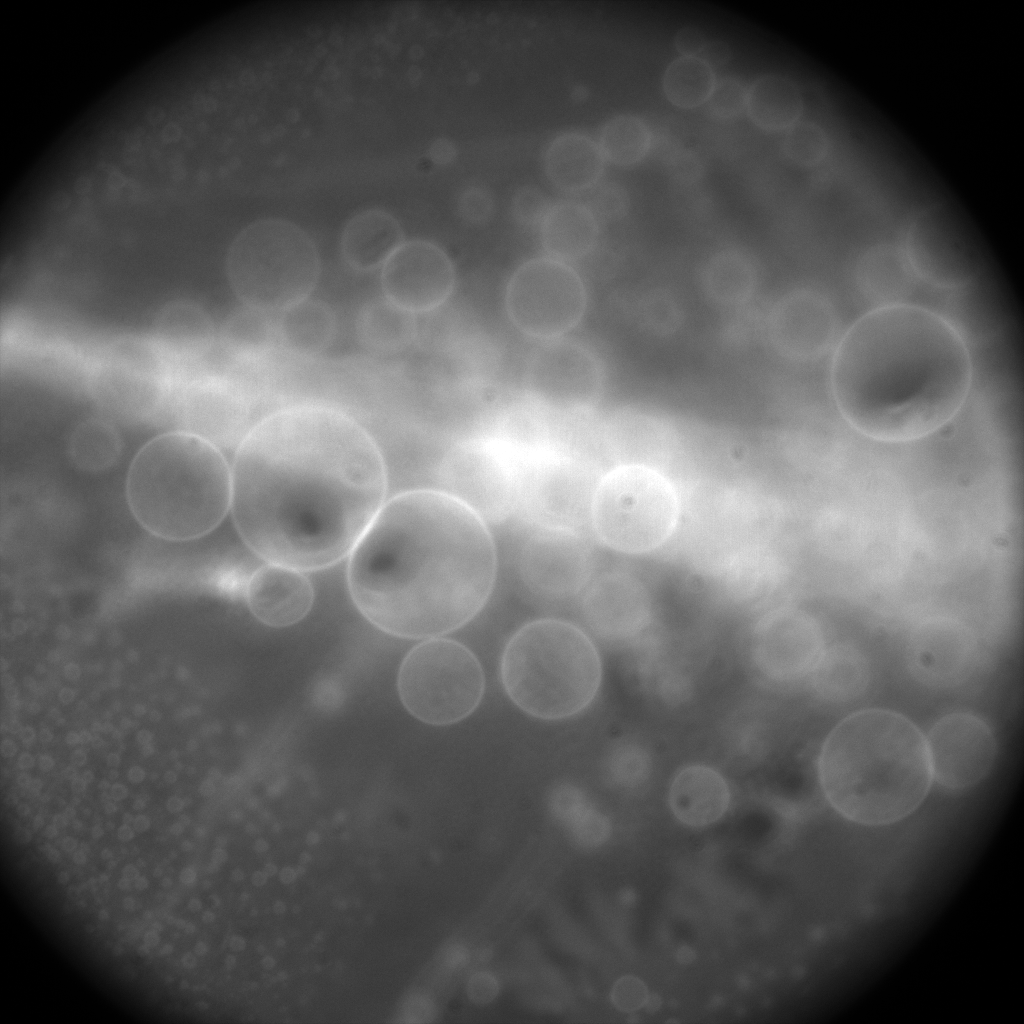

Supplement: S12 File — Zip file archive containing original photomicrographs of polymersomes formed following rehydration at 70°C. (ZIP) [file pone.0158729.s012.zip › 70C/Image_4103_20150311_180636.tif]

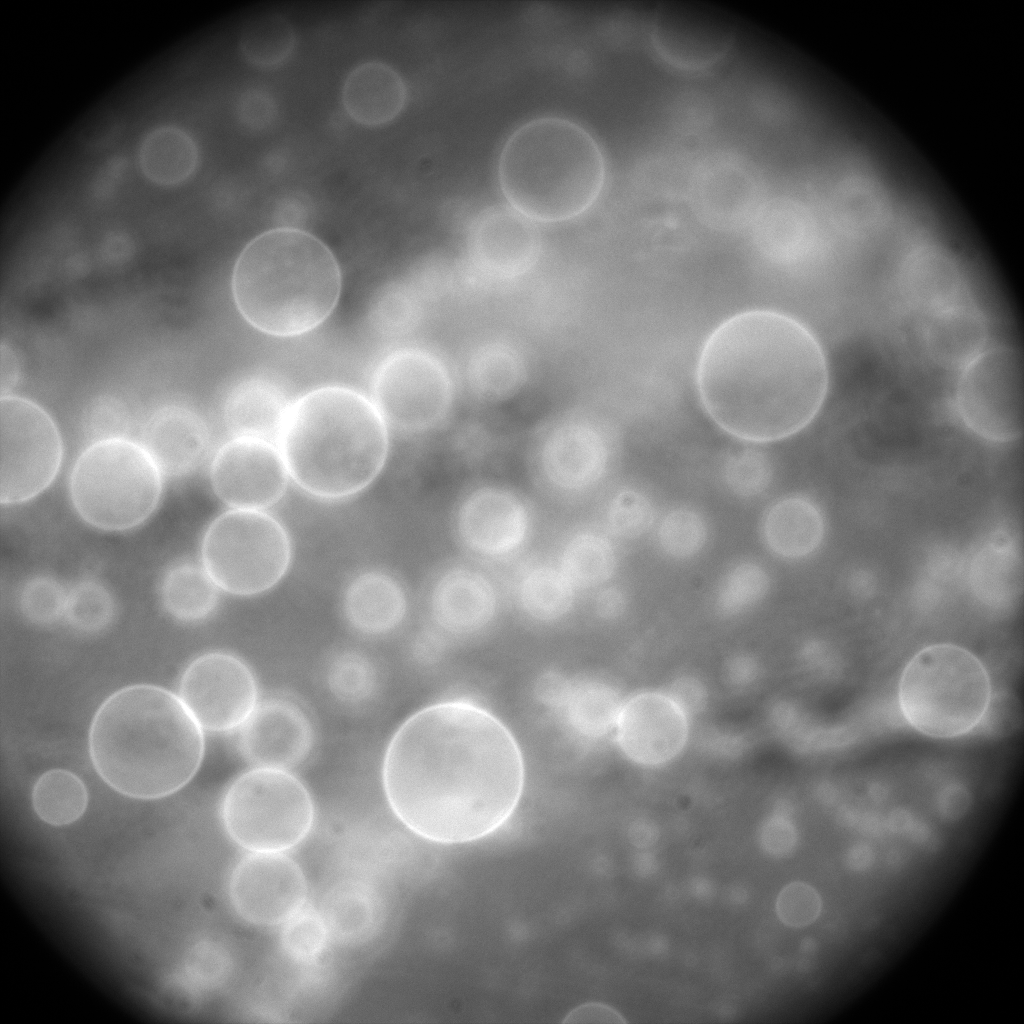

Supplement: S12 File — Zip file archive containing original photomicrographs of polymersomes formed following rehydration at 70°C. (ZIP) [file pone.0158729.s012.zip › 70C/Image_4105_20150311_180653.tif]

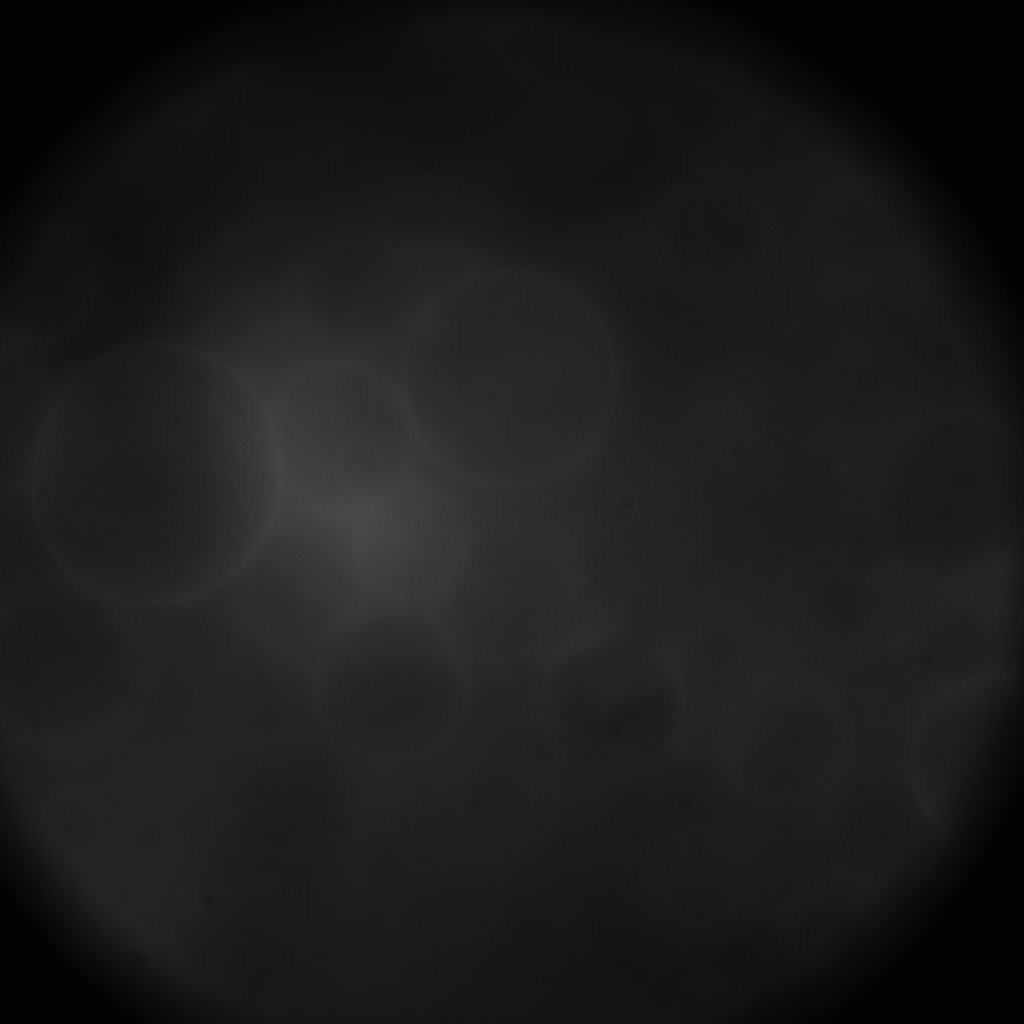

Supplement: S12 File — Zip file archive containing original photomicrographs of polymersomes formed following rehydration at 70°C. (ZIP) [file pone.0158729.s012.zip › 70C/Image_4108_20150311_180742.tif]

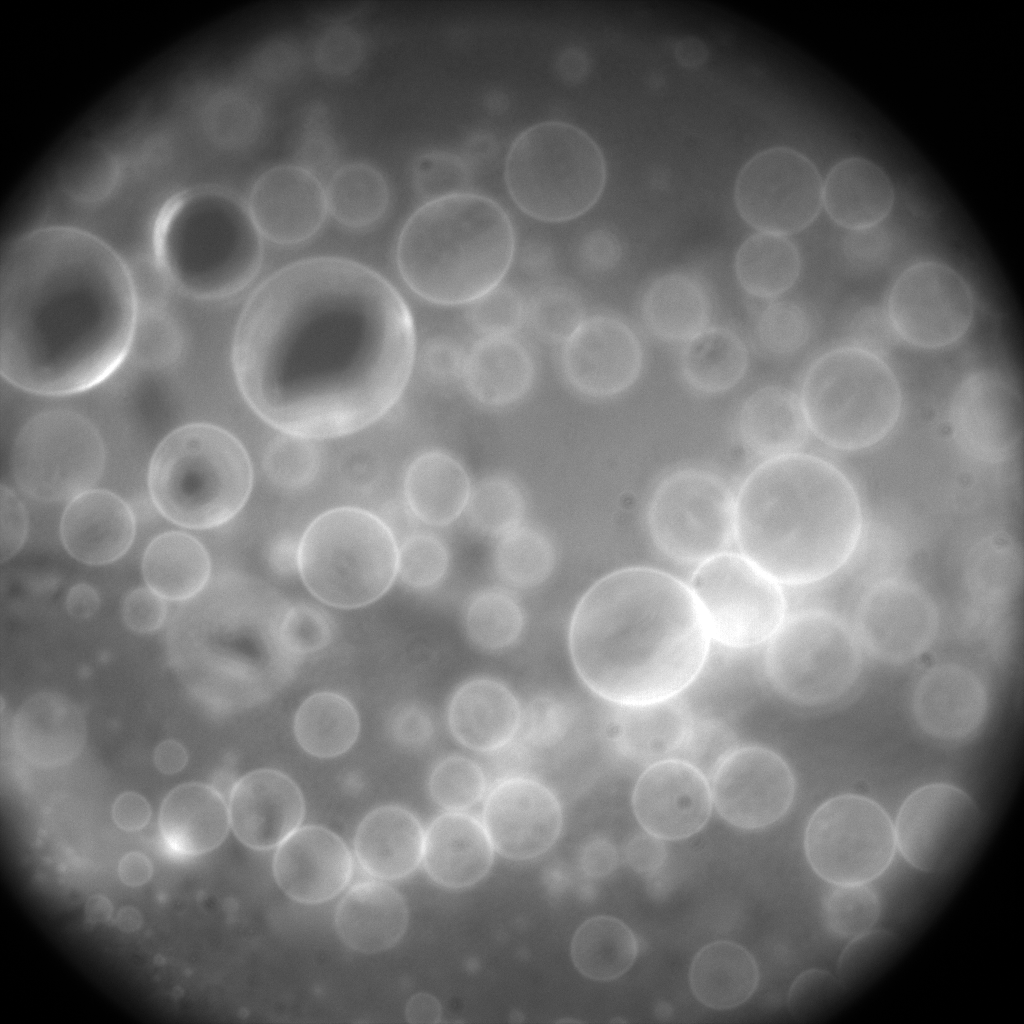

Supplement: S12 File — Zip file archive containing original photomicrographs of polymersomes formed following rehydration at 70°C. (ZIP) [file pone.0158729.s012.zip › 70C/Image_4110_20150311_180800.tif]

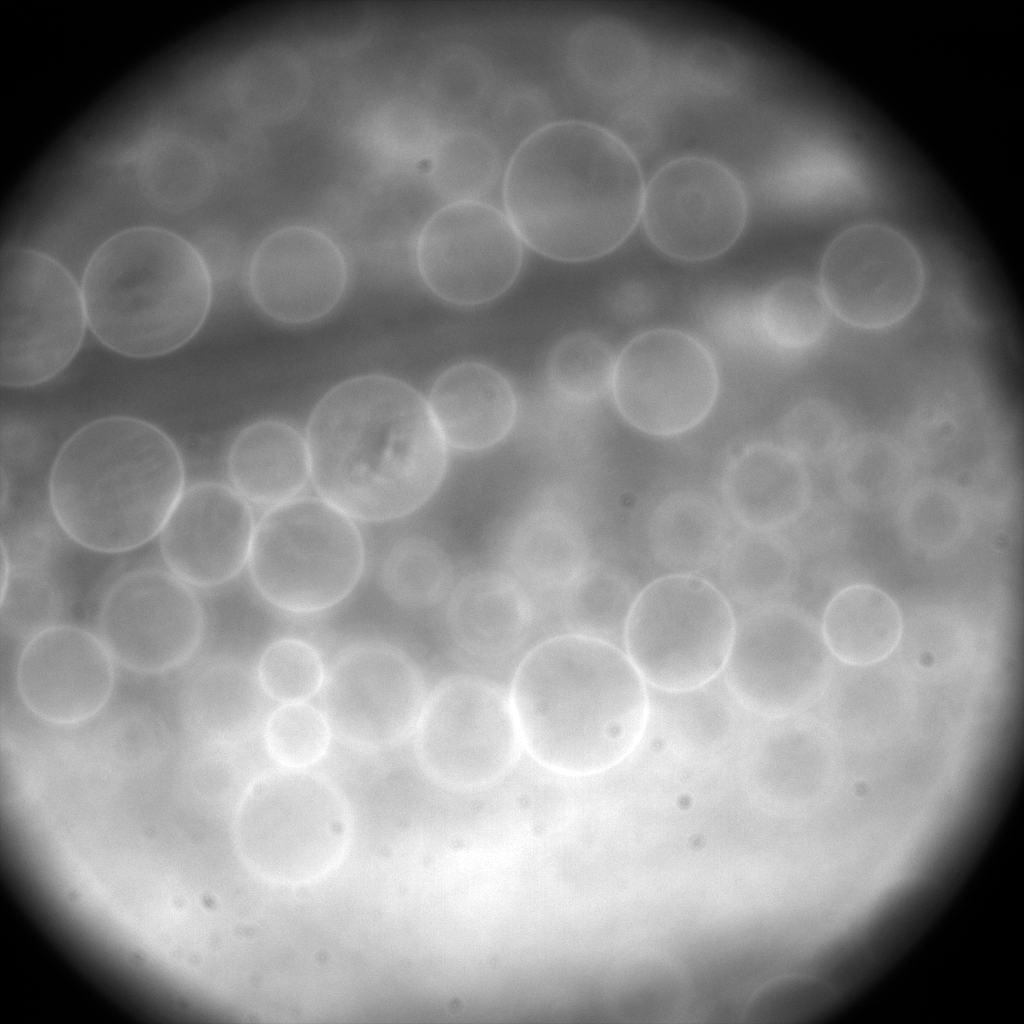

Supplement: S12 File — Zip file archive containing original photomicrographs of polymersomes formed following rehydration at 70°C. (ZIP) [file pone.0158729.s012.zip › 70C/Image_4112_20150311_180854.tif]

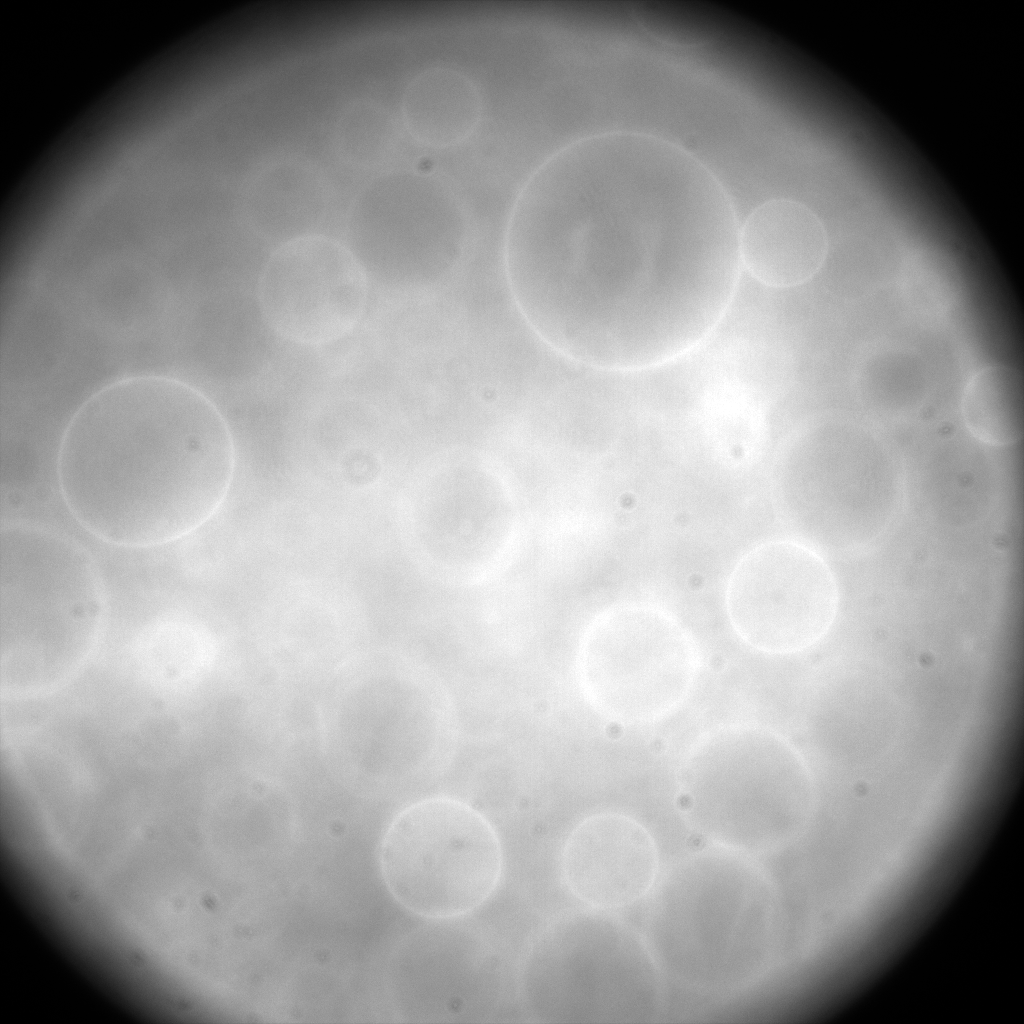

Supplement: S12 File — Zip file archive containing original photomicrographs of polymersomes formed following rehydration at 70°C. (ZIP) [file pone.0158729.s012.zip › 70C/Image_4114_20150311_180927.tif]

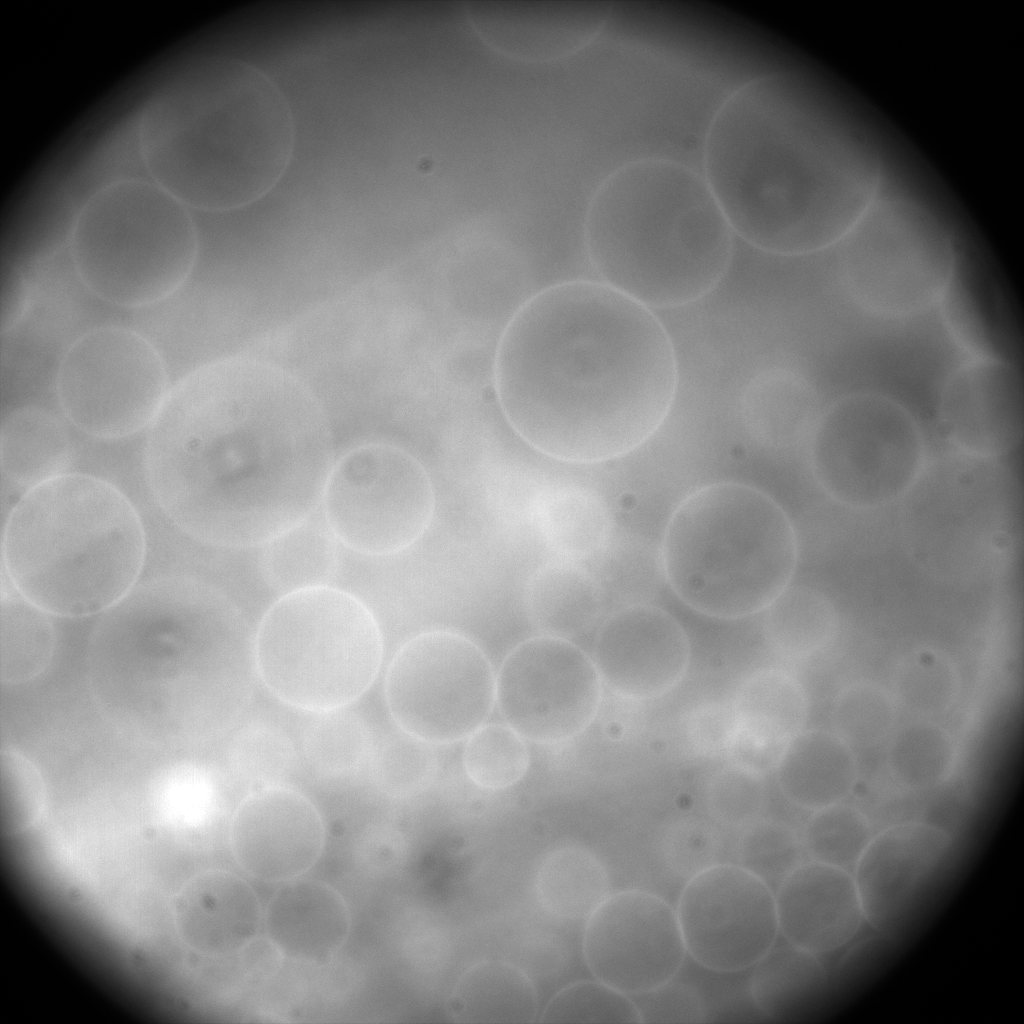

Supplement: S12 File — Zip file archive containing original photomicrographs of polymersomes formed following rehydration at 70°C. (ZIP) [file pone.0158729.s012.zip › 70C/Image_4116_20150311_181032.tif]

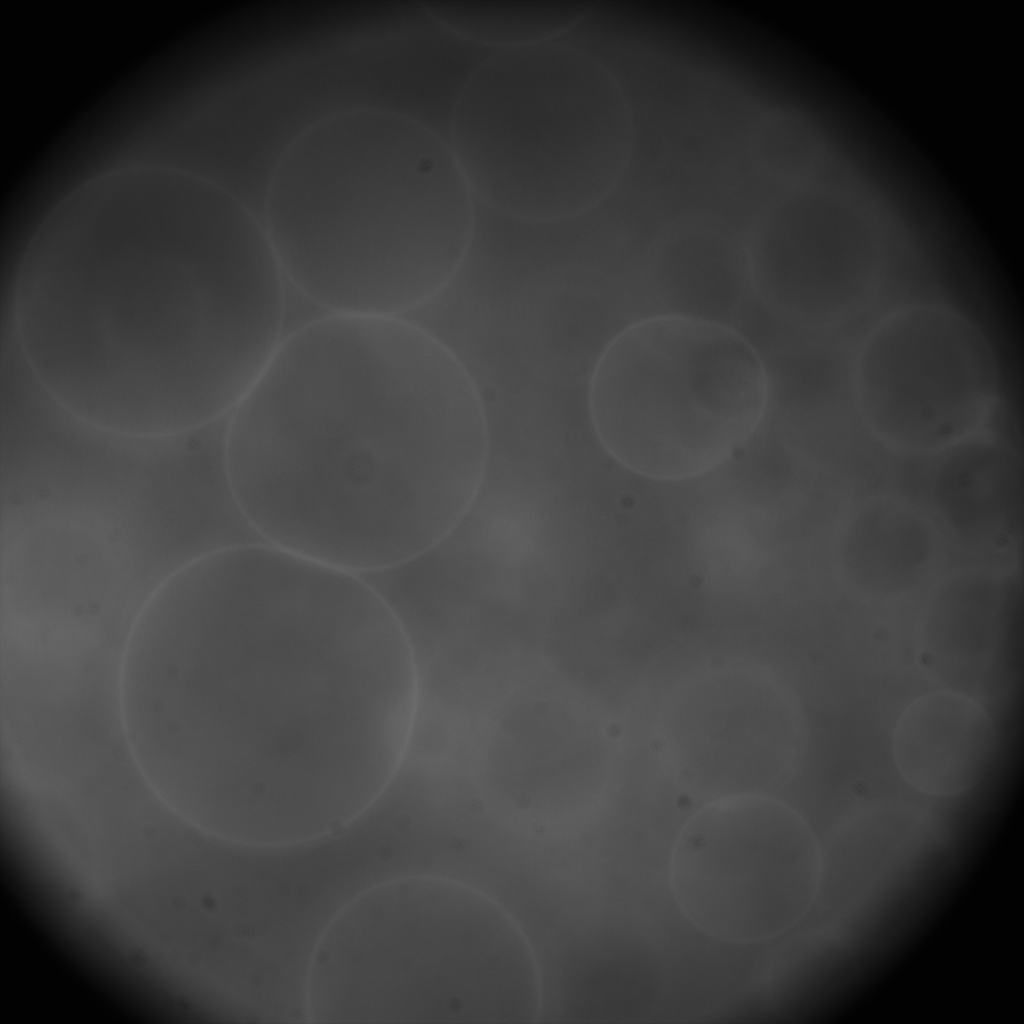

Supplement: S12 File — Zip file archive containing original photomicrographs of polymersomes formed following rehydration at 70°C. (ZIP) [file pone.0158729.s012.zip › 70C/Image_4118_20150311_181145.tif]

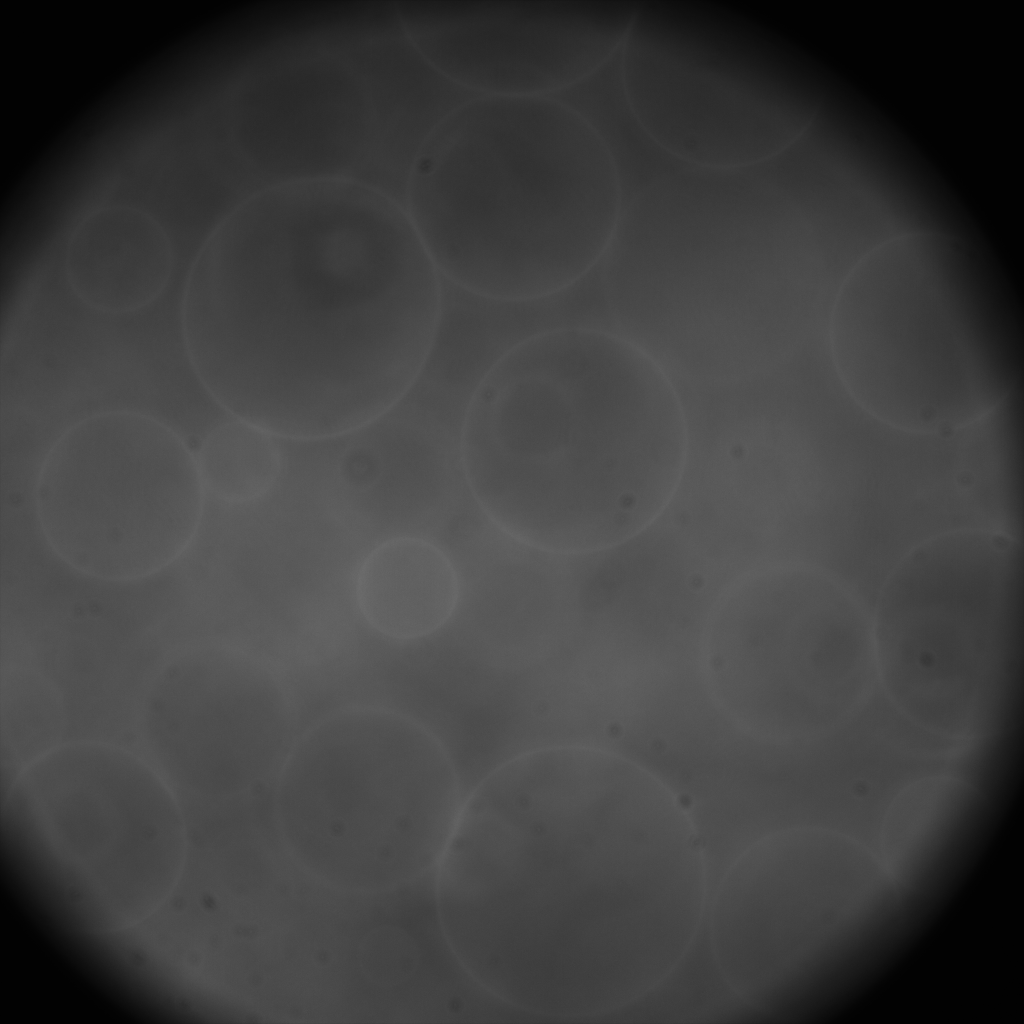

Supplement: S12 File — Zip file archive containing original photomicrographs of polymersomes formed following rehydration at 70°C. (ZIP) [file pone.0158729.s012.zip › 70C/Image_4120_20150311_181159.tif]
